# Sulfonium Salts as Leaving Groups for Aromatic Labelling of Drug-like Small Molecules with Fluorine-18

**DOI:** 10.1038/srep09941

**Published:** 2015-04-21

**Authors:** Kerstin Sander, Thibault Gendron, Elena Yiannaki, Klaudia Cybulska, Tammy L. Kalber, Mark F. Lythgoe, Erik Årstad

**Affiliations:** 1 Institute of Nuclear Medicine – Radiochemistry, University College London, 235 Euston Road (T-5), London NW1 2BU, UK; 2 Department of Chemistry, University College London, 20 Gordon Street, London WC1H 0AJ, UK; 3 Division of Biosciences, University College London, Gower Street, London WC1E 6BT, UK; 4 Centre for Advanced Biomedical Imaging, University College London, 72 Huntley Street, London WC1E 6DD, UK

## Abstract

Positron emission tomography (PET) is unique in that it allows quantification of biochemical processes *in vivo*, but difficulties with preparing suitably labelled radiotracers limit its scientific and diagnostic applications. Aromatic [^18^F]fluorination of drug-like small molecules is particularly challenging as their functional group compositions often impair the labelling efficiency. Herein, we report a new strategy for incorporation of ^18^F into highly functionalized aromatic compounds using sulfonium salts as leaving groups. The method is compatible with pharmacologically relevant functional groups, including aliphatic amines and basic heterocycles. Activated substrates react with [^18^F]fluoride at room temperature, and with heating the reaction proceeds in the presence of hydrogen bond donors. Furthermore, the use of electron rich spectator ligands allows efficient and regioselective [^18^F]fluorination of non-activated aromatic moieties. The method provides a broadly applicable route for ^18^F labelling of biologically active small molecules, and offers immediate practical benefits for drug discovery and imaging with PET.

Positron emission tomography (PET) is a highly sensitive medical imaging technique that relies on the use of radioactive tracers for quantification of biochemical processes *in vivo*. While several radionuclides are suitable for PET, fluorine-18 (half-life 110 min) is by far the most widely used due to its near ideal decay properties for imaging, small atomic size and ability to form stable C-F bonds. Fluorine-18 is typically produced by bombardment of ^18^O enriched water ([^18^O]H_2_O) with a beam of protons, which provides [^18^F]fluoride in high yield and with high specific activity through the ^18^O(p,n)^18^F nuclear reaction. However, labelling of drug-like small molecules is challenging as there are few positions available for incorporation of ^18^F, and the presence of hydrogen bond donating and accepting groups often impairs the reactivity. With the restrains imposed by the half-life of ^18^F, which makes multi-step radiochemical routes impractical, and the limitations of protection-deprotection strategies, the drug-like chemical space that is accessible for labelling remains marginal. It is therefore a pressing need to develop new strategies for incorporation of ^18^F that combine broad substrate scope with high functional group tolerance, in particular to basic aliphatic moieties, in order to expand the arsenal of PET tracers that can be made available for clinical applications.

Aromatic [^18^F]fluorination is attractive as the steric and electronic effects of substituting a hydrogen atom with fluorine are subtle, yet it can improve the metabolic stability of the parent compound^1^,^2^. Nucleophilic aromatic substitution of nitro, trimethylammonium and halide leaving groups with [^18^F]fluoride is the most widely used approach. However, the reaction is limited to activated substrates, harsh conditions are typically required, and purification of the resulting tracers can be difficult^3^,^4^. Extensive efforts have been made to overcome these limitations, and recent progress in fluorine chemistry has greatly expanded the repertoire of reactions available for [^18^F]fluorination^5^. This includes the use of alternative leaving groups such as aromatic sulfonium salts and sulfoxides^6^,^7^, as well as transition metal and hypervalent iodine complexes^8^. The development of palladium mediated fluorination was a major conceptual breakthrough as it allows labelling of a wide range of aromatic moieties, including electron rich arenes, with [^18^F]fluoride^9^,^10^. Yet, applications of the method have been hampered by the reliance on air sensitive palladium complexes that are demanding to synthesize, and that must be prepared freshly before labelling. Arylnickel complexes were reported to overcome some of these shortcomings, but have only been demonstrated to work with analytical levels of [^18^F]fluoride (<20 MBq)^11^. Recently, pinacol derived aryl boronate esters were shown to undergo efficient ^18^F labelling in the presence of a commercially available copper complex (Cu(OTf)_2_(py)_4_,), which makes this approach more accessible, although the high concentration of precursors used in the reactions pose a number of practical challenges^12^. Following two decades of optimisation, several hypervalent iodine complexes have emerged as attractive alternatives to transition metal based labelling methods as they allow [^18^F]fluorination of a broad scope of aromatic moieties using conventional reaction conditions^13^,^14^,^15^. Despite these remarkable developments, hypervalent iodine, as well as transition metal complexes, have so far only been applied to ‘late-stage' [^18^F]fluorination of fully protected substrates, such as amino acids, or compounds that lack reactive functional groups, for instance [^18^F]fluoroestrone and the TSPO ligand [^18^F]DAA1106^5^. Direct aromatic [^18^F]fluorination of non-activated compounds containing aliphatic amines or other similarly basic moieties, has yet to be demonstrated. This is a fundamental limitation for applications to PET, as the majority of drugs contain basic moieties, very frequently tertiary aliphatic amines, which cannot be masked with protection-deprotection strategies^16^.

Recently, we aimed to design ^18^F labelled tracers for imaging of transport mechanisms at the blood-brain barrier, using fragments from archetypical centrally-acting drugs such as haloperidol, terfenadine and ritanserin (Figure 1). Due to the poor compatibility of the available labelling methods with the structural properties of these drugs, in particular the shared piperidine scaffold, sulfonium salts were explored as alternative leaving groups for [^18^F]fluorination. Herein, we report the development and applications of a highly efficient and versatile method for aromatic labelling of drug-like molecules based on the reaction of functionalized triarylsulfonium salts with [^18^F]fluoride.

## Results

### Synthesis of functionalized sulfonium salts as labelling precursors

**Figure 1:**
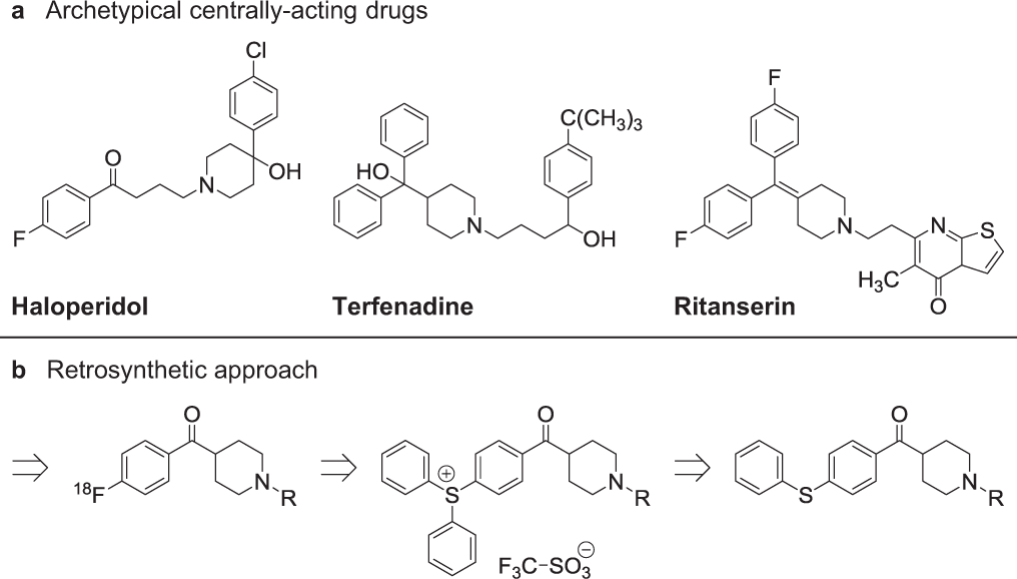
Approach to labelling of drug-like molecules. (**a**) Structures of archetypical centrally-acting drugs. (**b**) Retrosynthetic approach to [^18^F]fluorinated aryl ketone building blocks.

**Figure 2:**
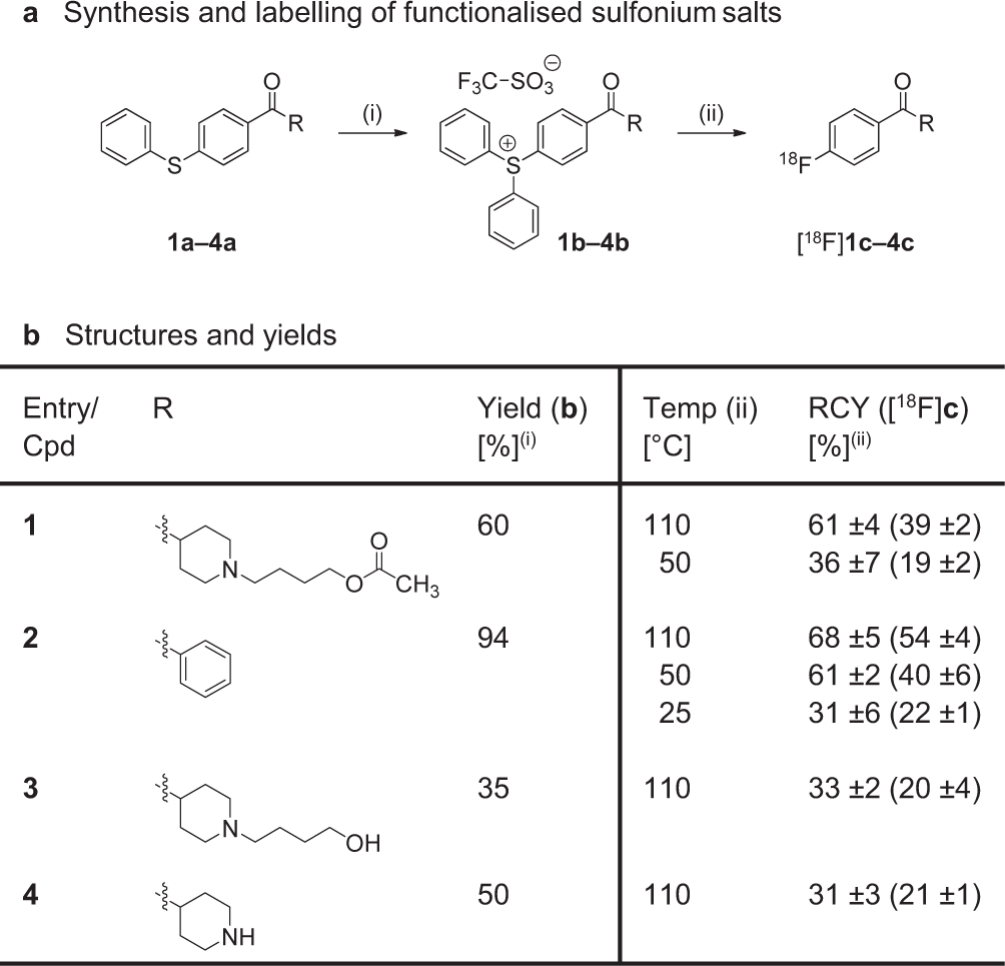
Synthesis of the triarylsulfonium salts 1b–4b and the corresponding radioactive products [^18^F]1c–4c. (**a**) Reagents and conditions: (i) TFSA (entries 1,3,4), diphenyliodonium triflate, Cu(II) benzoate, chlorobenzene, 1 h, 125°C; (ii) [^18^F]F−, KHCO_3_, K_222_, DMSO, 15 min. (**b**) Structures and yields: (i) isolated as triarylsulfonium triflate salts; non-optimized yields of a single reaction; (ii) analytical (and decay-corrected isolated) radiochemical yields (RCY); experiments were performed in triplicate (±SD).

Triarylsulfonium salts have been reported to allow highly efficient labelling of [^18^F]fluorobenzene and activated aryls, but as few methods are available to prepare the required precursors, practical examples have so far been limited to simple aromatic systems bearing unreactive functional groups^7^. In our case, employment of a ketone building block (*cf.* Figure 1) precluded the use of organometallic reagents^17^,^18^,^19^, and attempted reaction of the thioether **1a** with diaryliodonium triflate under copper(II) catalysis^20^ failed to provide the desired sulfonium salt **1b** (Figure 2). As aliphatic amines coordinate strongly with copper(II) ions, and the resulting complexes can undergo an intramolecular redox reaction to give copper(I)^21^, we attempted to mask the piperidine moiety by protonation with trifluoromethanesulfonic acid (TFSA). Gratifyingly, the corresponding TFSA salt of **1a** reacted cleanly with diaryliodonium triflate (125°C, 1 h) to give the desired sulfonium salt **1b** in 60% yield (Figure 2). The compound proved highly stable and could readily be purified by column chromatography on silica. To enable labelling, the base was liberated after purification by extraction with aqueous sodium hydroxide (2 M).

### Radiolabelling of functionalized sulfonium salts

**Figure 3:**
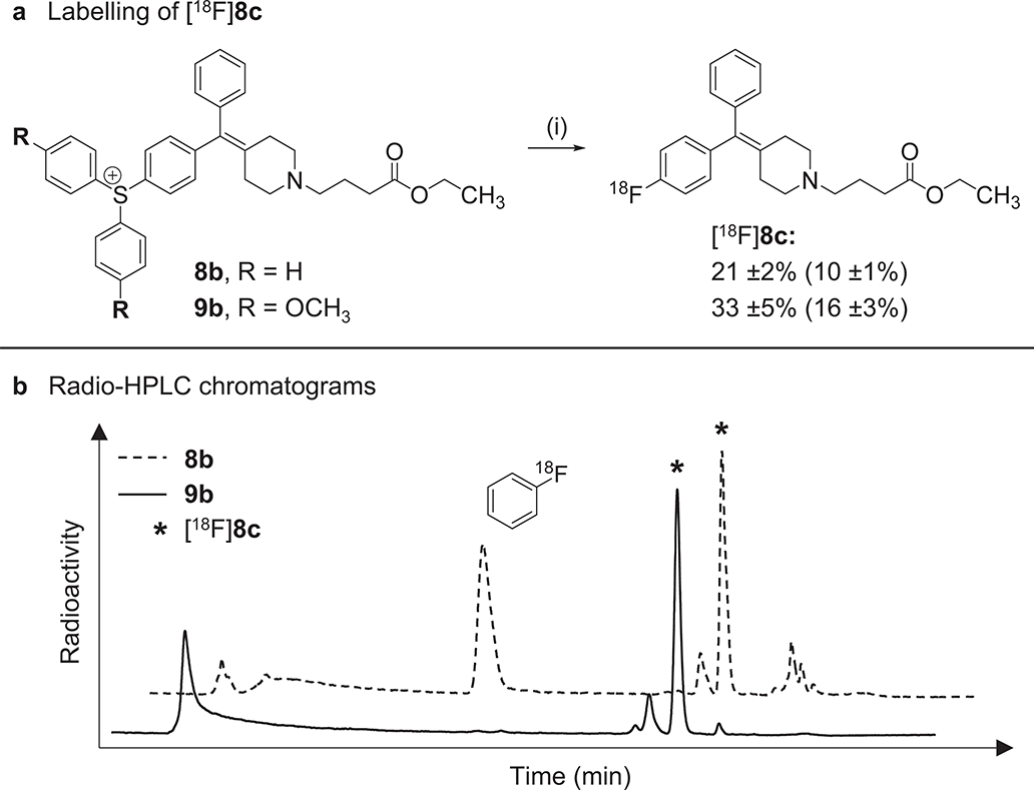
Labelling of [^18^F]8c. (**a**) Reagents and conditions: (i) [^18^F]F−, KHCO_3_, K_222_, DMSO, 15 min, 110°C; analytical (and decay-corrected isolated) radiochemical yields; experiments were performed in triplicates (±SD). (**b**) Radio-HPLC chromatograms of the reaction mixture when using **8b** (dotted line) or **9b** (full line) as labelling precursor.

**Figure 4:**
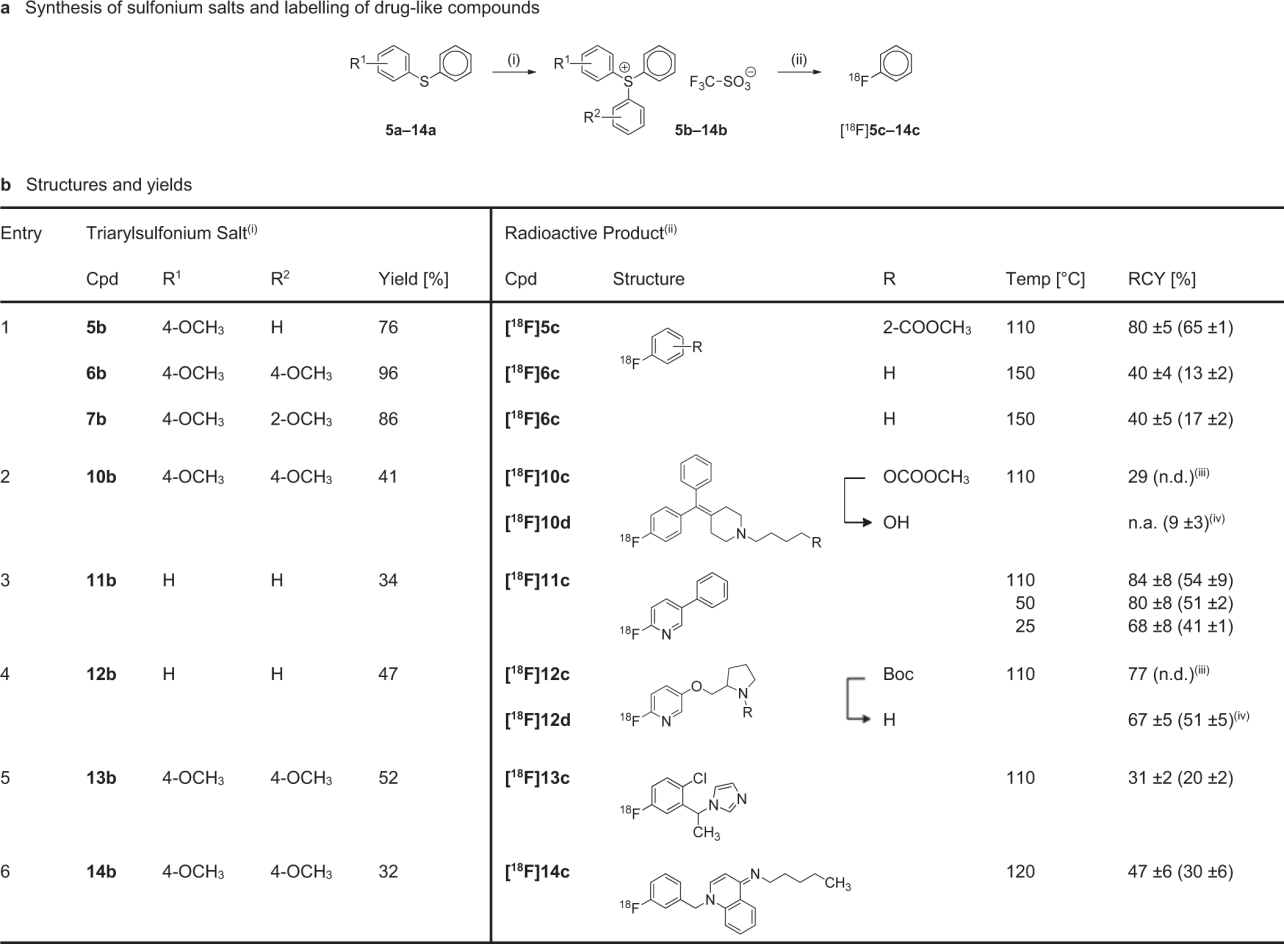
Synthesis of the functionalized triarylsulfonium salts 5b–14b and labelling of the corresponding radioactive products [^18^F]5c–14c. (**a**) Reagents and conditions: (i) TFSA (entries 2,5,6), diaryliodonium triflate, Cu(II) benzoate, chlorobenzene, 1–2 h, 125°C; (ii) [^18^F]F−, KHCO_3_, K_222_, DMSO, 15 min. (**b**) Structures and yields: (i) isolated as triflate salts; non-optimized yields of a single reaction; (ii) analytical (and decay-corrected isolated) radiochemical yields (RCY); experiments were performed in triplicate (±SD); (iii) analytical RCY of a single experiment; (iv) RCY after two steps.

Screening of solvents and bases commonly used for nucleophilic substitution reactions with [^18^F]fluoride revealed dimethyl sulfoxide (DMSO) and potassium bicarbonate to be optimal for labelling. Under these conditions (5 mg precursor, 0.5 ml DMSO, 110°C, 15 min), the sulfonium salt **1b** reacted with [^18^F]fluoride to give [^18^F]**1c** in 61% radiochemical yield (RCY) as determined by radio-HPLC (*cf.* Figure 2). The reaction also proceeded at 50°C, although in lower yield (Supporting information, Figure S14). To gain a better understanding of the impact of the temperature on the reaction, we used benzophenone **2b** as a model compound. For this substrate, labelling proceeded with comparable efficiency at 50 and 110°C, and afforded [^b18^F]**2c** in 61% and 68% RCY, respectively. Remarkably, the reaction also took place at room temperature to give [^18^F]**2c** in 31% RCY. The high reactivity prompted us to explore labelling in the presence of hydrogen bond donating groups with the aim to circumvent the need for protection-deprotection strategies. Indeed, the primary alcohol **3b** reacted smoothly with [^18^F]fluoride at 110°C to give [^18^F]**3c** in 33% RCY. Similarly, the secondary amine [^18^F]**4c** was obtained in 31% RCY using the sulfonium salt **4b** as the precursor. While nucleophilic [^18^F]fluorination has been reported to occur in the presence of unprotected alcohols, this is the first demonstration that labelling of small molecules with [^18^F]fluoride can be achieved in useful RCY in the presence of secondary amines and primary alcohols^5^,^8^,^22^. Encouraged by the unique reactivity of the sulfonium salts, we investigated the possibility of labelling drug-like target compounds directly (*cf.* Figure 1). Reaction of the sulfonium salt **8b**, decorated with an electron neutral piperidin-4-ylidene moiety, with [^18^F]fluoride resulted in formation of [^18^F]fluorobenzene as the major radiochemical product (28% RCY), but nevertheless provided the desired product [^18^F]**8c** in 21% RCY (Figure 3). In order to improve the regioselectivity of the reaction we opted to design triarylsulfonium salts with electron-rich aromatic ligands. In accordance with published results^7^, and as demonstrated by the clean reaction of the asymmetric sulfonium salt **5b** to [^18^F]2-fluorobenzoate (80% RCY), [^18^F]fluoride preferentially reacts at the most electron deficient of the three sulfonium-substituted carbons (Figure 4b, Entry 1). Further evaluation of sulfonium salts bearing two anisole groups (**6b** and **7b**) showed identical labelling efficiency for *ortho*- and *para*-methoxy substituents, with formation of [^18^F]fluorobenzene in 40% RCY. For practical reasons, and to aid characterization by NMR, *para*-methoxy residues were chosen as spectator ligands, and the sulfonium salt **9b** was prepared accordingly. In contrast to the parent compound **8b**, the *para*-methoxy functionalized precursor **9b** reacted regioselectively and provided [^18^F]**8c** with increased RCY (33%) (Figure 3). To the best of our knowledge, this is the first example of direct aromatic [^18^F]fluorination of a non-activated substrate in the presence of a non-protected aliphatic amine.

### Applications to PET

**Figure 5:**
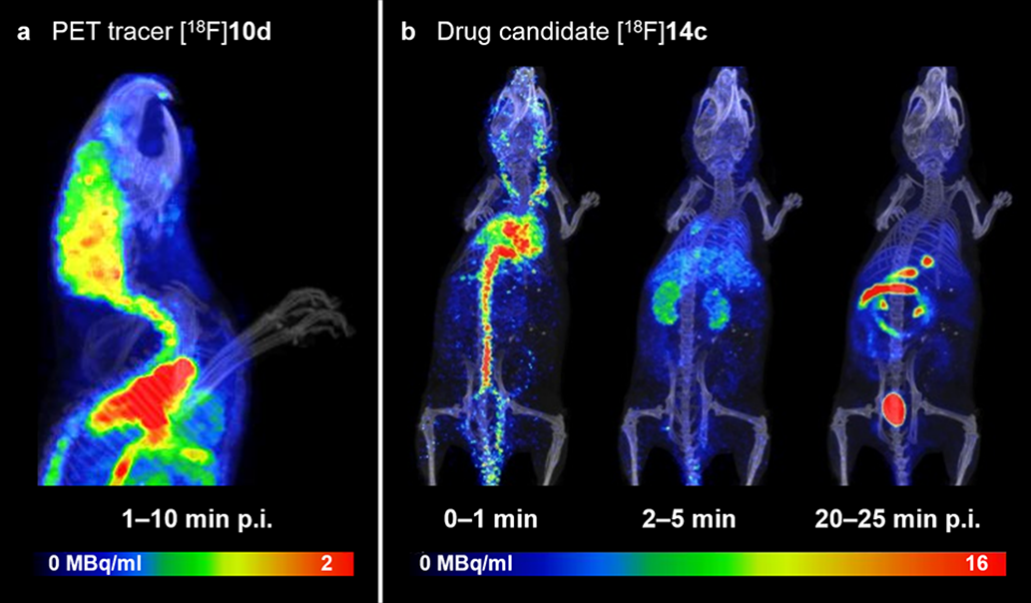
Nano-PET/CT imaging in mice. (**a**) Peak brain uptake of the PET tracer [^18^F]**10d**. (**b**) Pharmacokinetic profile of the drug candidate [^18^F]**14c**.

With this new labelling strategy in hand we prepared several derivatives of [^18^F]**8c**, and following biological evaluation, [^18^F]**10d** was identified as a lead compound (details to be reported elsewhere). The tracer exhibits an exceptionally high brain uptake with peak brain-to-blood ratios approaching 10: 1 in rodents (Figure 5). However, the lack of an activating electron withdrawing group in compound [^18^F]**10d**, and the presence of the primary alcohol, proved detrimental to the labelling efficiency (< 5% RCY). The alcohol was therefore protected as an acetate, and treatment of the corresponding sulfonium salt precursor **10b** with [^18^F]fluoride, followed by ester hydrolysis, afforded the target compound in 9% decay-corrected isolated RCY (Figure 4b, Entry 2). The total synthesis time was 120 min, including formulation. When starting with 2 GBq, tracer [^18^F]**10d** was obtained with a specific activity of 4.0 GBq/μmol.

The scope of the labelling reaction was further evaluated using heteroaromatic compounds (Figure 4b, Entries 3–6). The highly activated diphenyl(pyridin-2-yl)sulfonium salt **11b** reacted readily with [^18^F]fluoride at room temperature to give [^18^F]**11c** in 68% analytical RCY, and with moderate heating (50°C) the RCY reached 80% (*cf.* Supporting information, Figure S14). To demonstrate practical applicability, the sulfonium salt **12b** was used to prepare the nicotinic acetylcholine receptor ligand [^18^F]**12d** as a representative example of a class of 2-fluoropyridine containing radiotracers, such as [^18^F]flubatine or [^18^F]nifene, that currently are in use for imaging of neurodegenerative diseases^23^,^24^. In this instance, the high polarity of the corresponding deprotected sulfonium salt of **12b** made purification difficult, and the Boc group was therefore left intact for the labelling reaction. Treatment of **12b** with [^18^F]fluoride, and subsequent deprotection, afforded [^18^F]**12d** in 51% decay-corrected isolated RCY. Imidazole is an important motif for PET chemistry as it mediates binding to an array of enzymes and receptors. As an example of this class of compounds, we labelled the benzylic 1*H*-imidazole [^18^F]**13c** (31% RCY), a structural analogue of [^11^C]metomidate, which has shown promise as a tracer for imaging of adrenal lesions^25^. Finally, we prepared the iminodihydroquinoline [^18^F]**14c**, a [^18^F]fluorinated analogue of the potassium channel blocker CP-339,818^26^, with the goal to characterise the pharmacokinetic properties of the compound by dynamic PET imaging. Under optimized reaction conditions (120°C, 15 min) the target compound was obtained in 30% decay-corrected isolated RCY. Intriguingly, the pharmacokinetic profile of [^18^F]**14c**, as determined by PET (Figure 5), closely resembled that of a previously reported [^125^I]iodinated derivative^27^. The high labelling efficiency achieved for the electron neutral benzylic group opens up the prospect of using ^18^F as a scouting radionuclide to characterize the pharmacokinetic properties of lead compounds during the early stages of drug development. The omnipresence of benzylic groups, and other electron neutral aromatic and heteroaromatic moieties, in drug-like small molecules makes this a widely applicable labelling strategy.

## Discussion

We have developed practical synthetic protocols that allow sulfonium salts to be incorporated as leaving groups for [^18^F]fluorination of drug-like molecules. Importantly, the method is fully compatible with Lewis bases, including aliphatic amines, imines and basic heterocycles, as well as other common functional groups, such as alcohols, aldehydes, ketones, and esters. However, the synthetic route is not without limitations. Diaryliodonium salts can react with a number of nucleophiles, and competing arylation of heteroatoms can prevent sulfonium salt formation^28^,^29^. *N*-arylation can also give rise to permanently charged side products that make purification of the target compounds impractical. While protonation of basic functional groups conveniently masks nucleophilic nitrogens, this is not always a feasible strategy, and for conjugated systems, protonation can abolish the reactivity of the diaryl thioether.

Triarylsulfonium salts have high thermal and chemical stability, yet they exhibit a remarkable reactivity with fluoride. Conveniently, [^18^F]fluorination can be carried out under conventional radiochemical conditions that are routinely used for GMP compliant automated tracer preparation. Labelling of activated substrates proceeds at room temperature, and with heating the reaction occurs in the presence of unprotected alcohols and secondary amines. Furthermore, the use of electron rich spectator ligands allows regioselective [^18^F]fluorination of non-activated aromatic moieties, including electron neutral benzylic groups. Importantly, Lewis bases, including tertiary amines, are well tolerated in the reaction. However, hydrogen bond donors reduce the labelling efficiency, and in the case of non-activated substrates, they need to be masked with protecting groups. Nonetheless, the ability to label non-activated aromatic groups with ^18^F in the presence of basic moieties provides, for the first time, a method that is broadly applicable to drug-like compounds, and hence opens up extensive pharmacological space for the design of small molecule PET tracers.

In conclusion, we have developed a novel strategy for aromatic [^18^F]fluorination of drug-like molecules using sulfonium salts as leaving groups. The method allows efficient labelling of electron neutral and electron deficient arenes in the presence of basic functional groups and heterocyclic moieties. This provides, for the first time, a broadly applicable and practical method for aromatic [^18^F]fluorination of drug-like molecules and offers immediate benefits for applications to drug discovery and medical imaging with PET.

## Methods

Synthetic procedures and full analytical characterisation of all labelling precursors, non-labelled reference compounds and [^18^F]labelled compounds can be found in the supplementary information.

### Synthesis of triarylsulfonium salts – example 1b

To a solution of the thioether **1a** (100 mg, 0.24 mmol = 1 equiv.) in chlorobenzene (1 ml) were added TFSA (1 equiv.), diphenyliodonium triflate (1 equiv.) and copper(II) benzoate hydrate (5%) and the mixture was heated at 125°C for 1 h. After cooling, the resulting brown oil was washed with diethyl ether (3 × 5 ml). The product was purified by column chromatography (DCM: methanol = 10: 0 to 9: 1). The isolated product was dissolved in DCM (5 ml) and washed with aqueous sodium hydroxide (2 M; 5 ml) and a saturated solution of sodium triflate (5 ml). The organic phase was dried (magnesium sulfate), filtered and concentrated to give **1b** as a colourless oil (90 mg, 60%).

### Labelling of triarylsulfonium salts – example [^18^F]1c

[^18^F]Fluoride in [^18^O]H_2_O (50–200 MBq) was trapped on a Sep-Pak® QMA cartridge and released with a solution (0.5 ml) of Kryptofix 222 (30 mM) and potassium bicarbonate (30 mM) in acetonitrile: water (85: 15). The solvent was removed by heating at 90°C under a stream of nitrogen, and [^18^F]fluoride was dried by azeotropic distillation with acetonitrile (2 × 0.5 ml; 90°C). The reaction vial was capped, a solution of the sulfonium salt **1b** (5 mg) in DMSO (0.5 ml) was added, and the mixture was stirred at 110°C for 15 min. After cooling, water (1.5 ml) was added and the reaction mixture was purified by HPLC to give [^18^F]**1c** in 39 ±2% decay-corrected isolated RCY. Analytical RCYs (61 ±4%) were measured with radio-HPLC.

### Determination of RCY and specific activity (*cf.* Figures 2 and 4)

The entire quenched reaction mixture was used to determine analytical and decay-corrected isolated RCY, and no corrections were made to account for losses during the preparative procedure. Decay-corrected isolated RCY were calculated by relating the amount of isolated radioactive product to the initial amount of [^18^F]fluoride in [^18^O]H_2_O. Analytical RCY were determined by integrating the area under the curve in the preparative radio-HPLC chromatogram. The specific activity was calculated from the total area under the curve in the UV chromatogram that overlapped with the peak of the radioactive target compound.

### Animal experiments

Animal experiments were performed in accordance with the United Kingdom Home Office's Animals (Scientific Procedures) Act 1986 and were approved by the University College London Animal Ethics Committee.

## Author Contributions

KS and EÅ have conceptualised the study and have written the manuscript. KS has carried out synthetic chemistry and radiochemistry, while TG, EY and KC have supported the work with preparation of precursors for labelling and reference compounds. *In vivo* experiments have been performed by KS, TG and TLK. MFL is co-I on awarded grants and has provided preclinical imaging facilities. EÅ is PI on awarded grants and has supervised the work.

## Supplementary Material

Supplementary Information Supplementary information
