# Supplementary material for: Sulfonium Salts as Leaving Groups for Aromatic Labelling of Drug-like Small Molecules with Fluorine-18
Source: Sci Rep. 2015 Apr 21;5:9941. doi: 10.1038/srep09941 (PMC4404714; doi:10.1038/srep09941)

Supplementary information

Sulfonium salts as leaving groups for aromatic labelling of drug-like small molecules with fluorine-18

Kerstin Sander[1], Thibault Gendron[1], Elena Yiannaki[2], Klaudia Cybulska[3], Tammy L. Kalber[4], Mark F. Lythgoe[4] & Erik Årstad[1,2,*]

1) Institute of Nuclear Medicine – Radiochemistry, University College London, 235 Euston Road (T-5), London NW1 2BU, UK
2) Department of Chemistry, University College London, 20 Gordon Street, London WC1H 0AJ, UK
3) Division of Biosciences, University College London, Gower Street, London WC1E 6BT, UK
4) Centre for Advanced Biomedical Imaging, University College London, 72 Huntley Street, London WC1E 6DD, UK

* Email: e.arstad@ucl.ac.uk

Contents

page

1) General considerations *S2*
   a. Abbreviations
   b. Equipment
2) Synthesis and analytical characterization of sulfonium salts *S3*
   a. General procedure for the preparation of sulfonium salts
   b. Compounds **1b**, **3b**, **4b**, and **8b**
   c. Compounds **2b** and **5b**–**7b**
   d. Compounds **9b** and **10b**
   e. Compound **11b**
   f. Compound **12b**
   g. Compound **13b**
   h. Compound **14b**
3) Synthesis and analytical characterization of fluorinated reference compounds *S21*
   a. Compounds **1c**, **3c**, **4c**, **8c**, and **10d**
   b. Compounds **11c**, **12d** and **14c**
   c. Compound **13c**
4) Radiosynthesis of fluorine-18 labelled compounds *S28*
   a. General procedure for labelling of sulfonium salts with fluorine-18
   b. Preparation of compounds **[^{18}F]1c**–**[^{18}F]8c** and **[^{18}F]11c**–**[^{18}F]13c**
   c. Preparation of compounds **[^{18}F]10d** and **[^{18}F]14c** for preclinical studies
   d. Labelling of **[^{18}F]1c** and **[^{18}F]11c** at 50 °C
5) Animal experiments *S36*
6) Appendix: ^{1}H and ^{13}C NMR spectra *S37*

1. General considerations

a. Abbreviations

Bis((2-diphenylphosphino)phenyl) ether – DPEPhos; dichloromethane – DCM; *N*,*N*-dimethylformamide – DMF; dimethyl sulfoxide – DMSO; pentyl – pent; phenyl – ph; positron emission tomography – PET; pyridine – pyr; pyrrolidine – pyrr; quinoline – quin; radiochemical yield – RCY; trifluoromethanesulfonic acid – TFSA; trifluoroacetic acid – TFA; tetrahydrofuran – THF.

Petrol refers to the distillation fraction of petroleum ether with a boiling point ranging from 40–60 °C.

b. Equipment

Reagents were purchased from Sigma-Aldrich, Acros Organics or Fluorochem and were used without further purification. Purification of non-labelled compounds by column chromatography was performed on silica unless otherwise specified. For characterization of non-labelled compounds, ^{1}H and ^{13}C NMR spectra were recorded at room temperature unless otherwise specified. The respective instruments, Bruker Avance 400, 500 or 600 were operated at a frequency of 400, 500 or 600 MHz for ^{1}H and 125 or 150 MHz for ^{13}C, respectively. Proton decoupled ^{19}F NMR spectra were recorded on a Bruker Avance 300 instrument at a frequency of 282 MHz. All spectra were internally referenced to the respective deuterated solvents. Chemical shifts are reported in ppm and coupling constants (J) are given in Hertz (Hz). Full NMR assignment was performed with the aid of multidimensional and long range experiments. High resolution mass data were recorded on either a Thermo Finnigan MAT900xp (CI, EI) or a MALDI microMX (TOF) mass spectrometer. Melting points were taken on a Gallenkamp heating block and are uncorrected.

All labelling reactions were performed manually using [^{18}F]fluoride in [^{18}O]H_2O. Radio-HPLC was performed with an Agilent 1200 HPLC system equipped with a 1200 Series Diode Array Detector and a GABI Star NaI(Tl) scintillation detector. The system was used for purification as well as characterization of radiotracers.

2. Synthesis and analytical characterization of sulfonium salts

a. General procedure for the preparation of sulfonium salts

The thioether was dissolved in chlorobenzene. For compounds **1b**, **3b**, **4b**, **8b**, **9b**, **10b**, **13b**, and **14b**, TFSA was added to form the respective ammonium salts *in situ*. After addition of diaryliodonium trifluoromethanesulfonate and copper(II) benzoate dihydrate, the resulting mixture was heated at 125 °C for 1–2 h, and then allowed to reach ambient temperature. The reaction mixture was diluted with diethyl ether (5 ml), which resulted in formation of a brown oil. The solvents were decanted, and the brown oil was washed by addition of diethyl ether (2 × 5 ml) under vigorous stirring, and the crude product was purified by column chromatography to give the target compound. To liberate the base from ammonium salts, the product was dissolved in DCM (5 ml), and washed with aqueous NaOH (2 M; 5 ml), followed by a saturated solution of sodium trifluoromethanesulfonate (5 ml). The organic phase was dried ($MgSO_4$), filtered and concentrated under reduced pressure.

b. Compounds 1b, 3b, 4b, and 8b

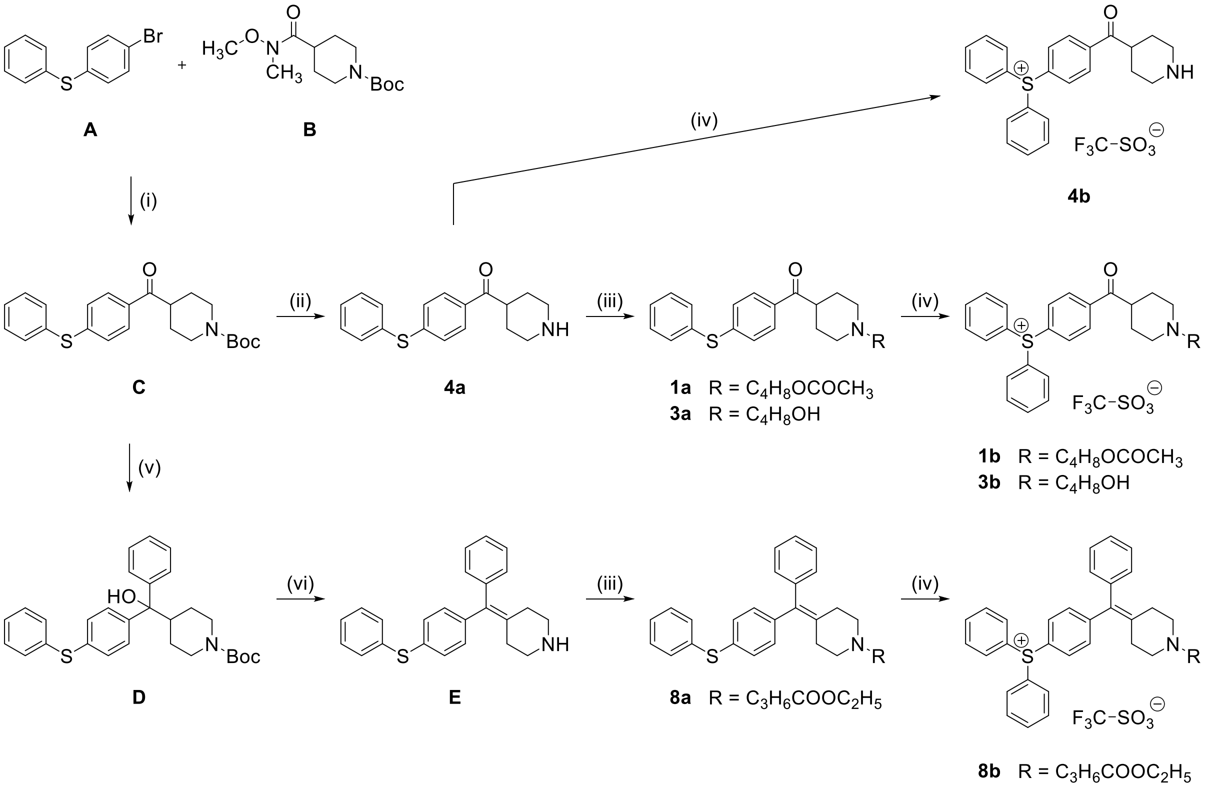

Scheme S1. Synthesis of the sulfonium salts **1b**, **3b**, **4b**, and **8b**

Reagents and conditions: (i) 1. *n*-BuLi, THF, –78 °C, 30 min; 2. **B**, THF, –78 °C – 0 °C, 30 min; (ii) HCl, THF, rt, 12 h; (iii) alkyl bromide, K_2CO_3, acetone, 60 °C – rt, 15 h; (iv) $(Ph)_2I^+ \cdot CF_3O_3S^-$, Cu(II) benzoate, TFSA, chlorobenzene, 125 °C, 1 h; (v) PhMgBr, THF, 0 °C – rt, 3 h; (vi) TFA, DCM, 0 °C – rt, 2 h.

(4-Bromophenyl)(phenyl)sulfane (A). Under inert atmosphere, a mixture of 1-bromo-4-iodobenzene (2.8 g, 10 mmol), thiophenol (1.24 ml, 12 mmol), potassium hydroxide (1.4 g, 25 mmol), copper(I) iodide (0.1 g, 0.5 mmol), and sarcosine (0.18 g, 2 mmol) in anhydrous dioxane (15 ml) was heated to 100 °C for 24 h. After cooling, the crude mixture was filtered over a plug of silica and the products were eluted with ethyl acetate. Purification by column chromatography (petrol) gave the product as a colourless oil (2.5 g, 94%). ^{1}H NMR (600 MHz, $CDCl_3$): δ 7.41 (d, J = 8.5 Hz, 2H, ph'-2,6*H*), 7.36 (d, J = 7.1 Hz, 2H, ph-2,6*H*), 7.33 (t, J = 7.4

Hz, 2H, ph-3,5*H*), 7.28 (t, J = 7.1 Hz, 1H, ph-4*H*), 7.18 (d, J = 8.5 Hz, 2H, ph'-3,5*H*); ^{13}C NMR (150 MHz, $CDCl_3$): δ 136.6 (ph-1*C*), 134.9 (ph'-1*C*), 132.4 (ph'-3,5*C*), 132.1 (ph-2,6*C*), 131.7 (ph'-2,6*C*), 129.5 (ph-3,5*C*), 127.7 (ph-4*C*), 121.0 (ph'-4*C*); HRMS (m/z): $[M]^+$ calcd. for $C_{12}H_9BrS$, 263.9603; found, 263.9598.

***tert*-Butyl 4-(methoxy(methyl)carbamoyl)piperidine-1-carboxylate (B).** Piperidine-1,4-dicarboxylic acid 1-*tert*-butyl ester 4-ethyl ester (3.5 g, 13.6 mmol) and *O*,*N*-dimethyl-hydroxylamine hydrochloride (2 g, 20.4 mmol) were dissolved in THF (20 ml). Under inert atmosphere and ice cooling, a solution of isopropylmagnesium chloride in THF (2 M; 20 ml, 40.8 mmol) was added dropwise. The mixture was stirred at room temperature for 5 h, subsequently quenched with a saturated solution of ammonium chloride and extracted with ethyl acetate. The organic phase was dried ($MgSO_4$) and concentrated under reduced pressure to give the product as a colorless oil (3.3 g, 89%). ^{1}H NMR (600 MHz, DMSO-d_6): δ 3.94 (br s, 2H, pip-2,6H^{eq}), 3.68 (s, 3H, OCH_3), 3.08 (s, 3H, NCH_3), 2.85–2.73 (m, 3H, pip-4*H*, pip-2,6H^{ax}), 1.63 (d, J = 12.1 Hz, 2H, pip-3,5H^{eq}), 1.39-1.33 (m, 11H, C(CH_3)$_3$, pip-3,5H^{ax}); ^{13}C NMR (150 MHz, DMSO-d_6): δ 174.7 (*C*=O^{amide}), 153.8 (*C*=O^{boc}), 78.6 (*C*(CH_3)$_3$), 61.5 (OCH_3), 43.3/42.3 (pip-2,6*C*), 37.0 (pip-4*C*), 31.8 (NCH_3), 28.1 (C(CH_3)$_3$), 27.8 (pip-3,5*C*); HRMS (m/z): $[M+H]^+$ calcd. for $C_{13}H_{24}O_4N_2$, 273.1814; found, 273.1817.

***tert*-Butyl 4-(4-(phenylthio)benzoyl)piperidine-1-carboxylate (C).** A solution of compound **A** (Scheme S1; 0.25 g, 0.94 mmol) in THF (0.5 ml) was cooled to –78 °C, and *n*-butyl lithium (2.5 M in hexane; 0.34 ml, 0.86 mmol) was added dropwise. After stirring for 30 min at –78 °C, a solution of compound **B** (Scheme S1; 0.23 g, 0.86 mmol) in THF (0.5 ml) was added. The mixture was allowed to come to 0 °C during 1 h and was subsequently quenched with a saturated solution of ammonium chloride. The crude product was extracted with ethyl acetate and purified by column chromatography (petrol: ethyl acetate = 10: 0 → 9: 1). The product was isolated as a white solid (0.1 g, 29%). Mp: 89–90 °C; ^{1}H NMR (600 MHz, DMSO-d_6): δ 7.92 (d, J = 8.6 Hz, 2H, ph-2,6*H*), 7.53-7.47 (m, 5H, ph'-2–6*H*), 7.25 (d, J = 8.5 Hz, 2H, ph-3,5*H*), 3.95 (br s, 2H, pip-2,6H^{eq}), 3.57–3.52 (m, 1H, pip-4*H*), 2.88 (br s, 2H, pip-2,6H^{ax}), 1.73 (d, J = 12.5 Hz, 2H, pip-3,5H^{eq}), 1.39–1.36 (m, 11H, C(CH_3)$_3$, pip-3,5H^{ax}); ^{13}C NMR (150 MHz, DMSO-d_6): δ 201.0 (*C*=O^{ketone}), 153.9 (*C*=O^{boc}), 143.7 (ph-4*C*), 133.6 (ph'-2,6*C*), 133.0 (ph'-1*C*), 131.2 (ph-1*C*), 130.1 (ph'-3,5*C*), 129.3 (ph-2,6*C*), 129.2 (ph'-4*C*), 127.4 (ph-3,5*C*), 78.7 (*C*(CH_3)$_3$), 43.3/42.3 (pip-2,6*C*), 42.2 (pip-4*C*), 28.2 (pip-3,5*C*), 28.1 (C(CH_3)$_3$); HRMS (m/z): $[M+Na]^+$ calcd. for $C_{23}H_{27}NO_3S$, 420.1609; found, 420.1606.

(4-(Phenylthio)phenyl)(piperidin-4-yl)methanone hydrochloride (4a). To a solution of compound **C** (Scheme S1; 0.72 g, 1.8 mmol) in THF (3.0 ml) was added HCl in dioxane (4 M; 1 ml). The solution was stirred for 12 h at room temperature. The resulting white solid was filtered off, washed with diethyl ether and dried *in vacuo* (0.48 g, 79%). Mp: 219–220 °C; ^{1}H NMR (600 MHz, DMSO-d_6): δ 9.18/8.87 (s/s, 1H/1H, NH_2^+), 7.94 (d, J = 8.5 Hz, 2H, ph-2,6*H*), 7.54–7.48 (m, 5H, ph'-2–6*H*), 7.26 (d, J = 8.5 Hz, 2H, ph-3,5*H*), 3.71–3.66 (m, 1H, pip-4*H*), 3.27 (d, J = 12.5 Hz, 2H, pip-2,6H^{eq}), 3.02–2.96 (m, 2H, pip-2,6H^{ax}), 1.90 (d, J = 12.5 Hz, 2H, pip-3,5H^{eq}), 1.78–1.71 (m, 2H, pip-3,5H^{ax}); ^{13}C NMR (150 MHz, DMSO-d_6): δ 200.2 (*C*=O), 144.1 (ph-4*C*), 133.7 (ph'-2,6*C*), 132.5 (ph-1*C*), 131.0 (ph'-1*C*), 130.2 (ph'-3,5*C*), 129.35 (ph-2,6*C*), 129.3 (ph'-4*C*), 127.3 (ph-3,5*C*), 42.4 (pip-2,6*C*), 40.1 (pip-4*C*), 25.0 (pip-3,5*C*); HRMS (m/z): $[M]^+$ calcd. for $C_{18}H_{19}NOS$, 297.1182; found, 297.1185.

4-(4-(4-(Phenylthio)benzoyl)piperidin-1-yl)butyl acetate (1a). Under inert atmosphere, compound **4a** (Scheme S1; 0.45 g, 1.3 mmol), 4-bromobutyl acetate (0.4 ml, 2.6 mmol) and potassium carbonate (0.72 g, 5.2 mmol) were dissolved in anhydrous acetone (20 ml). After heating at 60 °C for 3 h the mixture was stirred at room temperature for 12 h. The inorganic compounds were filtered off and the crude product purified by column chromatography (DCM:

methanol = 98: 2). The product was isolated as a colourless oil (0.3 g, 56%). ^{1}H NMR (600 MHz, DMSO-d_6): δ 7.89 (d, J = 8.5 Hz, 2H, ph-2,6*H*), 7.53–7.46 (m, 5H, ph'-2–6*H*), 7.25 (d, J = 8.5 Hz, 2H, ph-3,5*H*), 3.99 (t, J = 6.6 Hz, 2H, but-1H_2), 3.31–3.27 (m, 1H, pip-4*H*), 2.87 (d, J = 10.3 Hz, pip-2,6H^{eq}), 2.28 (br s, 2H, but-4H_2), 2.00–1.99 (m, 5H, COCH_3, pip-2,6H^{ax}), 1.71 (d, J = 12.5 Hz, 2H, pip-3,5H^{eq}), 1.58–1.49 (m, 4H, but-2H_2, pip-3,5H^{ax}), 1.48–1.43 (m, 2H, but-3H_2); ^{13}C NMR (150 MHz, DMSO-d_6): δ 201.6 (*C*=O^{ketone}), 170.5 (*C*=O^{acetyl}), 143.4 (ph-4*C*), 133.6 (ph'-2,6*C*), 133.2 (ph-1*C*), 131.3 (ph'-1*C*), 130.1 (ph'-3,5*C*), 129.2 (ph-2,6*C*), 129.16 (ph'-4*C*), 127.5 (ph-3,5*C*), 63.8 (but-1*C*), 57.6 (but-4*C*), 52.6 (pip-2,6*C*), 42.7 (pip-4*C*), 28.5 (but-2*C*), 26.2 (pip-3,5*C*), 22.8 (but-3*C*), 20.8 (CO*C*H_3); HRMS (m/z): $[M+H]^+$ calcd. for $C_{24}H_{29}NO_3S$, 412.1946; found, 412.1954).

(4-(1-(4-Acetoxybutyl)piperidine-4-carbonyl)phenyl)diphenylsulfonium trifluoromethanesulfonate (1b). Following the general method for preparation of sulfonium salts (page S3), compound **1a** (Scheme S1; 100 mg, 0.24 mmol), chlorobenzene (1 ml), TFSA (0.02 ml, 0.24 mmol), diphenyliodonium trifluoromethanesulfonate (100 mg, 0.24 mmol) and copper(II) benzoate dihydrate (4 mg, 0.012 mmol) were reacted at 125 °C for 1 h. Treatment with diethyl ether, column chromatography (DCM: methanol = 10: 0 → 9: 1) and extractive workup yielded the product as a colourless oil (90 mg, 60%). ^{1}H NMR (600 MHz, DMSO-d_6): δ 8.26 (d, J = 8.6 Hz, 2H, ph'-3,5*H*), 7.92 (d, J = 8.6 Hz, 2H, ph'-2,6*H*), 7.89–7.86 (m, 6H, ph-2,4,6*H*), 7.80 (t, J = 7.9 Hz, 4H, ph-3,5*H*), 4.00 (t, J = 6.5 Hz, 2H, but-4H_2), 3.46 (br s, 1H, pip-4*H*), 2.98 (br s, 2H, pip-2,6H^{eq}), 2.43 (br s, 2H, but-1H_2), 2.20 (br s, 2H, pip-2,6H^{ax}), 2.00 (s, 3H, COCH_3), 1.81 (d, J = 12.2 Hz, 2H, pip-3,5H^{eq}), 1.60–1.50 (m, 6H, but-2,3H_2, pip-3,5H^{ax}); ^{13}C NMR (150 MHz, DMSO-d_6): δ 201.7 (*C*=O^{ketone}), 170.5 (*C*=O^{acetyl}), 139.6 (ph'-1*C*), 134.6 (ph'-3,5*C*), 131.7 (ph-4*C*), 131.6 (ph-3,5*C*), 131.5 (ph-2,6*C*), 130.5 (ph'-2,6*C*), 129.9 (ph'-4*C*), 124.9 (ph-1*C*), 120.7 (q, $^{1}J_{C,F}$ = 321.6, *C*F_3), 63.7 (but-4*C*), 57.1 (but-1*C*), 52.2 (pip-2,6*C*), 42.8 (pip-4*C*), 27.7 (but-3*C*), 26.0 (pip-3,5*C*), 22.4 (but-2*C*), 20.8 (CO*C*H_3); ^{19}F NMR (282 MHz, DMSO-d_6): δ –78.20 (CF_3); HRMS (m/z): $[M]^+$ calcd. for $C_{30}H_{34}NO_3S$, 488.2259; found, 488.2274.

(1-(4-Hydroxybutyl)piperidin-4-yl)(4-(phenylthio)phenyl)methanone (3a). Under inert atmosphere, a solution of compound **4a** (Scheme S1; 0.13 g, 0.4 mmol), 4-bromobutan-1-ol (0.12 g, 0.8 mmol) and potassium carbonate (0.21 g, 1.6 mmol) in anhydrous acetonitrile (10 ml) was heated at 60 °C for 5 h. After cooling, the inorganic compounds were filtered off and the crude product was purified by column chromatography (DCM: methanol = 100: 0 → 95: 5). The product was isolated as a colourless oil (0.1 g, 67%). ^{1}H NMR (600 MHz, $CDCl_3$): δ 7.77 (d, J = 8.5 Hz, 2H, ph-2,6*H*), 7.49–7.47 (m, 2H, ph'-2,6*H*), 7.41–7.38 (m, 3H, ph'-3–5*H*), 7.19 (d, J = 8.6 Hz, 2H, ph-3,5*H*), 3.56 (t, J = 4.6 Hz, 2H, but-4CH_2), 3.23–3.18 (m, 1H, pip-4*H*), 3.02 (d, J = 11.4 Hz, 2H, pip-2,6H^{eq}), 2.42 (t, J = 5.1 Hz, 2H, but-1H_2), 2.20 (br s, 2H, pip-2,6H^{ax}), 1.89–1.83 (m, 4H, pip-3,5H_2), 1.67–1.66 (m, 4H, but-2,3H_2); ^{13}C NMR (150 MHz, $CDCl_3$): δ 201.2 (*C*=O), 145.0 (ph'-4*C*), 134.1 (ph'-2,6*C*), 133.3 (ph'-1*C*), 132.0 (ph-1*C*), 129.8 (ph'-3,5*C*), 129.0 (ph'-4*C*), 128.95 (ph-2,6*C*), 127.6 (ph-3,5*C*), 62.7 (but-4*C*), 58.7 (but-1*C*), 52.9 (pip-2,6*C*), 43.0 (pip-4*C*), 32.6 (but-3*C*), 28.1 (pip-3,5*C*), 25.4 (but-2*C*); HRMS (m/z): $[M+H]^+$ calcd. for $C_{22}H_{27}NO_2S$, 370.1841; found, 370.1841.

(4-(1-(4-Hydroxybutyl)piperidine-4-carbonyl)phenyl)diphenylsulfonium trifluoromethanesulfonate (3b). Following the general method for preparation of sulfonium salts (page S3), compound **3a** (Scheme S1; 70 mg, 0.19 mmol), chlorobenzene (1 ml), TFSA (17.0 µl, 0.19 mmol), diphenyliodonium trifluoromethanesulfonate (74 mg, 0.19 mmol) and copper(II) benzoate dihydrate (3.1 mg, 0.01 mmol) were reacted at 125 °C for 1 h. Treatment with diethyl ether, column chromatography (DCM: methanol = 10: 0 → 8: 2) and extractive workup yielded the product as a light brown oil (40 mg, 35%). ^{1}H NMR (600 MHz, $CDCl_3$): δ

8.19 (d, J = 8.6 Hz, 2H, ph'-2,6*H*), 7.92 (d, J = 8.7 Hz, 2H, ph'-3,5*H*), 7.80 (t, J = 7.3 Hz, 2H, ph-4*H*), 7.77 (d, J = 7.6 Hz, 4H, ph-2,6*H*), 7.73 (t, J = 7.8 Hz, 4H, ph-3,5*H*), 3.56 (br s, 2H, but-4H_2), 3.31–3.26 (m, 1H, pip-4*H*), 3.05 (d, J = 11.2, 2H, pip-2,6H^{eq}), 2.44 (br s, 2H, but-1H_2), 2.21 (t, J = 11.4 Hz, 2H, pip-2,6H^{ax}), 1.92–1.83 (m, 4H, pip-3,5H_2), 1.68 (br s, 4H, but-2,3H_2); ^{13}C NMR (150 MHz, $CDCl_3$): δ 200.7 (*C*=O), 140.7 (ph'-1*C*), 135.1 (ph-4*C*), 132.0 (ph-3,5*C*), 131.9 (ph'-2.6*C*), 131.5 (ph-2,6*C*), 130.0 (ph'-3,5*C*), 128.7 (ph'-4*C*), 123.9 (ph-1*C*), 120.9 (q, $^1J_{C,F}$ = 320.8 Hz, CF_3), 62.8 (but-4*C*), 58.7 (but-1*C*), 52.8 (pip-2,6*C*), 44.0 (pip-4*C*), 32.7 (but-3*C*), 27.9 (pip-3,5*C*), 25.6 (but-2*C*); ^{19}F NMR (282 MHz, $CDCl_3$): δ –78.1 (CF_3).

Diphenyl(4-(piperidine-4-carbonyl)phenyl)sulfonium trifluoromethanesulfonate (4b). Following the general method for preparation of sulfonium salts (page S3), compound **4a** (Scheme S1; 0.11 g, 0.38 mmol), chlorobenzene (1 ml), TFSA (34.0 µl, 0.38 mmol), diphenyliodonium trifluoromethanesulfonate (0.16 g, 0.38 mmol) and copper(II) benzoate dihydrate (6 mg, 0.018 mmol) were reacted at 125 °C for 1 h. Treatment with diethyl ether, column chromatography (DCM: methanol = 10: 0 → 9.5: 0.5) and extractive workup yielded the product as a light brown oil (0.05 g, 23%). ^{1}H NMR (600 MHz, DMSO-d_6): δ 8.27 (d, J = 8.6 Hz, 2H, ph'-2,6*H*), 7.93 (d, J = 8.7 Hz, 2H, ph'-3,5*H*), 7.90–7.86 (m, 6H, ph-2,4,6*H*), 7.80 (t, J = 7.9 Hz, 4H, ph-3,5*H*), 3.61 (t, J = 11.3 Hz, 1H, pip-4*H*), 3.11 (d, J = 12.5 Hz, 2H, pip-2,6H^{eq}), 2.76 (td, J = 12.3/2.1 Hz, 2H, pip-2,6H^{ax}), 1.79 (d, J = 12.4 Hz, 2H pip-3,5H^{eq}), 1.52 (qd, J = 12.5/3.2 Hz, 2H, pip-3,5H^{ax}); ^{13}C NMR (150 MHz, DMSO-d_6): δ 201.4 (*C*=O), 139.4 (ph'-4*C*), 134.6 (ph-4*C*), 131.7 (ph'-3,5*C*), 131.6 (ph-2,6*C*), 131.5 (ph-3,5*C*), 130.5 (ph'-2,6*C*), 129.9 (ph'-1*C*), 124.9 (ph-1*C*), 120.7 (q, $^1J_{C,F}$ = 323.4 Hz, CF_3), 44.3 (pip-2,6*C*), 42.6 (pip-4*C*), 27.4 (pip-3,5*C*); ^{19}F NMR (282 MHz, DMSO-d_6): δ –77.77 (CF_3); HRMS (m/z): $[M]^+$ calcd. for $C_{24}H_{24}NOS$, 374.1579; found, 374.1572.

***tert*-Butyl 4-(hydroxy(phenyl)(4-(phenylthio)phenyl)methyl)piperidine-1-carboxylate (D).** A solution of compound **C** (Scheme S1; 0.4 g, 1.0 mmol) in THF (2 ml) was added dropwise to a solution of phenylmagnesium bromide (1 M in THF; 2 ml, 2.0 mmol) kept at 0 °C. The mixture was allowed to come to ambient temperature and was subsequently heated at 60 °C for 3 h. After cooling, the reaction mixture was quenched with brine, extracted with ethyl acetate and purified by column chromatography (petrol: ethyl acetate = 10: 0 → 8: 2) to afford the product as a white solid (0.24 g, 50%). Mp: 151–152 °C; ^{1}H NMR (600 MHz, DMSO-d_6): δ 7.52–7.50 (m, 4H, ph-2,6*H*, ph'-3,5*H*), 7.36–7.34 (m, 2H, ph-3,5*H*), 7.29–7.26 (m, 5H, ph''-2,3,5,6*H*, ph-4*H*), 7.23 (d, J = 8.5 Hz, 2H, ph'-2,6*H*), 7.14 (t, J = 7.3 Hz, 1H, ph''-4*H*), 5.38 (s, 1H, CO*H*), 3.94 (br s, 2H, pip-2,6H^{eq}), 2.72–2.61 (m, 3H, pip-4*H*, pip-2,6H^{ax}), 1.36 (s, 9H, $C(CH_3)_3$), 1.30–1.22 (m, 4H, pip-3,5H_2); ^{13}C NMR (150 MHz, DMSO-d_6): δ 153.7 (*C*=O), 146.7 (ph-1*C*, ph'-1*C*), 134.9 (ph''-1*C*), 131.9 (ph'-4*C*), 130.6 (ph''-2,6*C*), 130.4 (ph'-2,6*C*), 129.6 (ph''-3,5*C*), 128.0 (ph''-3,5*C*), 127.4 (ph''-4*C*), 127.1 (ph'-3,5*C*), 126.1 (ph-4*C*), 125.7 (ph-2,6*C*), 78.5/78.4 ($C(CH_3)_3$, *C*OH), 44.0 (pip-2,6*C*), 43.2 (pip-4*C*), 28.1 ($C(CH_3)_3$), 26.0 (pip-3,5*C*); HRMS (m/z): $[M]^+$ calcd. for $C_{29}H_{33}NO_3S$, 498.2079; found, 498.2078.

4-(Phenyl(4-(phenylthio)phenyl)methylene)piperidine (E). Compound **D** (Scheme S1; 1.64 g, 3.4 mmol) was dissolved in DCM (20 ml). TFA (2 ml) was added and the solution was stirred 1 h at room temperature. The mixture was made basic by addition of aqueous NaOH (2M) and the organic phase was separated. The resulting crude product was purified by column chromatography (DCM: methanol = 10: 0 → 8: 2) to give the product as a colourless oil (0.63 g, 53%). ^{1}H NMR (600 MHz, DMSO-d_6): δ 8.9 (br s, 1H, N*H*), 7.41–7.32 (m, 7H, ph''-2–6*H*, ph-3,5*H*), 7.27–7.23 (m, 3H, ph-4*H*, ph'-3,5*H*), 7.14–7.12 (m, 4H, ph'-2,6*H*, ph-2,6*H*), 3.11–3.08 (m, 4H, pip-2,6H_2), 2.45–2.40 (m, 4H, pip-3,5H_2); ^{13}C NMR (150 MHz, DMSO-d_6): δ 141.0 (ph-1*C*), 140.1 (ph'-1*C*), 136.9 (*C*=C^{pip}), 134.0 (ph''-1*C*), 133.8 (ph'-4*C*), 131.4 (ph''-2,6*C*), 130.7 (C=C^{pip}), 130.4 (ph'-3,5*C*), 129.8 (ph-3,5*C*), 129.7 (ph''-3,5*C*), 129.3 (ph-2,6*C*),

128.4 (ph'-2,6*C*), 127.9 (ph''-4*C*), 127.0 (ph-4*C*), 44.4 (pip-2,6*C*), 28.1 (pip-3,5*C*); HRMS (m/z): $[M+H]^+$ calcd. for $C_{24}H_{23}NS$, 358.1624; found, 358.1627.

Ethyl 4-(4-(phenyl(4-(phenylthio)phenyl)methylene)piperidin-1-yl)butanoate (8a). Under inert atmosphere, compound **E** (Scheme S1; 0.18 g, 0.5 mmol), 4-bromo-butyric acid ethyl ester (0.14 ml, 1.0 mmol) and potassium carbonate (0.28 g, 2.0 mmol) were dissolved in anhydrous acetone (10 ml). After heating at 60 °C for 3 h the mixture was stirred at room temperature for a further 12 h. The inorganic compounds were filtered off and the crude product was purified by column chromatography (DCM: methanol = 100: 0 → 98: 2). The product was isolated as a colourless oil (0.17 g, 71%). ^{1}H NMR (600 MHz, DMSO-d_6): δ 7.39–7.30 (m, 7H, ph-3,5*H*, ph''-2-6*H*), 7.24–7.20 (m, 3H, ph'-3,5*H*, ph-4*H*), 7.08–7.06 (m, 4H, ph-2,6*H*, ph'-2,6*H*), 4.03 (q, J = 7.1 Hz, 2H, ethyl-CH_2), 2.40–2.37 (m, 4H, pip-2,6H_2), 2.30–2.25 (m, 4H, but-2,4H_2), 2.25–2.12 (m, 4H, pip-3,5H_2), 1.66 (q, J = 7.2 Hz, 2H, but-3H_2), 1.16 (t, J = 7.1 Hz, 3H, ethyl-CH_3); ^{13}C NMR (150 MHz, DMSO-d_6): δ 172.9 (*C*=O), 141.8 (ph-1*C*), 141.1 (ph'-1*C*), 136.2 (*C*=C^{pip}), 134.4 (ph''-1*C*), 134.2 (ph'-4*C*), 132.9 (C=C^{pip}), 131.1 (ph''-2,6*C*), 130.6 (ph'-3,5*C*), 130.1 (ph-3,5*C*), 129.7 (ph''-3,5*C*), 129.5 (ph-2,6*C*), 128.3 (ph'-2,6*C*), 127.6 (ph''-4*C*), 126.6 (ph-4*C*), 59.7 (ethyl-*C*H_2), 56.8 (but-4*C*), 54.6 (pip-2,6*C*), 31.6 (pip-3,5*C*), 31.3 (but-2*C*), 22.0 (but-3*C*), 14.2 (ethyl-*C*H_3); HRMS (m/z): $[M+H]^+$ calcd. for $C_{30}H_{33}NO_2S$, 472.2305; found, 472.2299.

(4-((1-(4-Ethoxy-4-oxobutyl)piperidin-4-ylidene)(phenyl)methyl)phenyl)diphenyl-sulfonium trifluoromethanesulfonate (8b). Following the general method for preparation of sulfonium salts (page S3), compound **8a** (Scheme S1; 150 mg, 0.32 mmol), chlorobenzene (1.5 ml), TFSA (25.5 µl, 0.32 mmol), diphenyliodonium trifluoromethanesulfonate (126 mg, 0.32 mmol) and copper(II) benzoate dihydrate (5.1 mg, 0.015 mmol) were reacted at 125 °C for 1 h. Treatment with diethyl ether, column chromatography (DCM: methanol = 98: 2 → 95: 5) and extractive workup yielded the product as a yellow oil (77 mg, 35%). ^{1}H NMR (400 MHz, DMSO-d_6, 128 °C): δ 7.90–7.77 (m, 12H, ph-2–6*H*, ph'-3,5*H*), 7.57 (d, J = 8.7 Hz, 2H, ph'-2,6*H*), 7.38 (t, J = 7.3 Hz, 2H, ph''-3,5*H*), 7.30 (t, J = 7.3 Hz, 2H, ph'-4*H*), 7.19 (d, J = 7.0 Hz, 2H, ph''-2,6*H*), 4.13 (q, J = 7.0 Hz, 2H, ethyl-CH_2), 2.96 (br s, 4H, pip-2,6H_2), 2.84 (br s, 4H, pip-3,5H_2), 2.45 (t, J = 5.0 Hz, 2H, but-1H_2), 2.39 (t, J = 7.2 Hz, 2H, but-3H_2), 1.89 (t, J = 7.0 Hz, 2H, but-2H_2), 1.23 (t, J = 7.1 Hz, 3H, ethyl-CH_3); ^{13}C NMR (150 MHz, DMSO-d_6): δ 172.7 (*C*=O), 147.8 (ph''-1*C*), 140.6 (ph'-4*C*), 135.2 (*C*=C^{pip}), 134.4 (ph-4*C*), 132.1 (ph'-3,5*C*), 131.8 (C=C^{pip}), 131.4 (ph-3,5*C*), 131.3 (ph-2,6*C*), 131.28 (ph'-2,6*C*), 130.7 (ph'-1*C*), 129.5 (ph''-2,6*C*), 128.6 (ph''-3,5*C*), 127.1 (ph''-4*C*), 125.3 (ph-1*C*), 120.7 (q, $^1J_{C,F}$ = 323.5 Hz, CF_3), 59.9 (ethyl-*C*H_2), 56.7 (but-1*C*), 54.4 (pip-2,6*C*), 31.5 (pip-3,5*C*), 30.6 (but-3*C*), 21.9 (but-2*C*), 14.2 (ethyl-*C*H_3); ^{19}F NMR (282 MHz, DMSO-d_6): δ –78.05 (CF_3); HRMS (m/z): $[M]^+$ calcd. for $C_{36}H_{38}NO_2S$, 548.2618; found, 548.2605.

c. Compounds 2b and 5b–7b

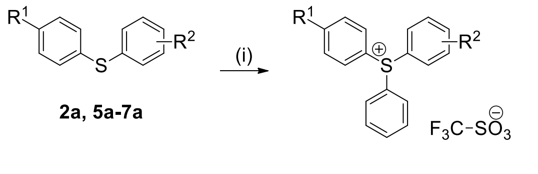

2a, 5a-7a

2b, 5b-7b

2a, 2b R^1 = H, R^2 = *p*-COC_6H_5
5a, 5b R^1 = *p*-OCH_3, R^2 = *o*-$COOCH_3$
6a, 6b R^1 = *p*-OCH_3, R^2 = *p*-OCH_3
7a, 7b R^1 = *p*-OCH_3, R^2 = *o*-OCH_3

Scheme S2. Synthesis of the sulfonium salts **2b** and **5b–7b**

Reagents and conditions: (i) $(Ph)_2I^+ \cdot CF_3O_3S^-$, Cu(II) benzoate, chlorobenzene, 125 °C, 1 h.

Phenyl(4-(phenylthio)phenyl)methanone (2a). Under inert atmosphere, tris(dibenzylideneacetone)dipalladium(0) (73 mg, 1%) and DPEPhos (86 mg, 2%) were

suspended in toluene (64 ml) and stirred for 10 min at room temperature. 4-Bromobenzophenone (2.1 g, 8 mmol), thiophenol (0.82 ml, 8 mmol) and potassium *tert*-butoxide (1 g, 8.8 mmol) were added and the solution was heated to 120 °C for 3 h. After cooling, the reaction mixture was filtered over a plug of Celite®, the filtrate washed with aqueous NaOH (2 M) and concentrated to dryness. Purification by column chromatography (petrol: ethyl acetate = 10: 0 → 95: 5) afforded the product as a white solid (2.1 g, 89%). Mp: 66–68 °C; ^{1}H NMR (600 MHz, $CDCl_3$): δ 7.77 (d, J = 7.9 Hz, 2H, ph-2,6*H*), 7.70 (d, J = 8.3 Hz, 2H, ph'-2,6*H*), 7.58 (t, J = 7.5 Hz, 1H, ph-4*H*), 7.54–7.52 (m, 2H, ph''-3,5*H*), 7.47 (t, J = 7.8 Hz, 2H, ph-3,5*H*), 7.43–7.38 (m, 3H, ph''-2,4,6*H*), 7.25 (d, J = 8.4 Hz, 2H, ph'-3,5*H*); ^{13}C NMR (150 MHz, $CDCl_3$): δ 195.9 (C=O), 144.4 (ph'-4*C*), 137.7 (ph'-1*C*), 134.9 (ph-1*C*), 134.0 (ph''-3,5*C*), 132.5 (ph-4*C*), 132.3 (ph''-1*C*), 130.9 (ph'-2,6*C*), 130.0 (ph-2,6*C*), 129.8 (ph''-2,6*C*), 128.9 (ph''-4*C*), 128.4 (ph-3,5*C*), 127.4 (ph'-3,5*C*); HRMS (m/z): $[M]^+$ calcd. for $C_{19}H_{14}SO$, 290.0765; found, 290.0761.

(4-Benzoylphenyl)diphenylsulfonium trifluoromethanesulfonate (2b). Following the general method for preparation of sulfonium salts (page S3), compound **2a** (Scheme S2; 0.2 g, 1.6 mmol), chlorobenzene (2 ml), diphenyliodonium trifluoromethanesulfonate (0.69 g, 1.6 mmol) and copper(II) benzoate dihydrate (26 mg, 0.08 mmol) were reacted at 125 °C for 2 h. Treatment with diethyl ether followed by column chromatography (DCM: methanol = 10: 0 → 9.5: 0.5) yielded the product as a brown solid (0.3 g, 94%). Mp: 37–40 °C; ^{1}H NMR (600 MHz, DMSO-d_6): δ 8.04 (d, J = 8.5 Hz, 2H, ph'-3,5*H*), 7.95 (d, J = 8.5 Hz, 2H, ph'-2,6*H*), 7.91–7.88 (m, 6H, ph''-3–5*H*), 7.83–7.80 (m, 6H, ph''-2,6*H*, ph-2,6*H*), 7.75 (t, J = 7.6 Hz, 1H, ph-4*H*), 7.60 (t, J = 7.7 Hz, 2H, ph-3,5*H*); ^{13}C NMR (150 MHz, DMSO-d_6): δ 194.5 (*C*=O), 141.8 (ph'-1*C*), 135.7 (ph-1*C*), 134.6 (ph''-4*C*), 133.8 (ph-4*C*), 131.7 (ph''-3,5*C*), 131.6 (ph'-3,5*C*), 131.5 (ph''-2,6*C*), 131.4 (ph'-2,6*C*), 130.0 (ph-2,6*C*), 129.1 (ph'-4*C*), 128.9 (ph-3,5*C*), 124.9 (ph''-1*C*), 120.7 (q, $^1J_{C,F}$ = 324.2 Hz, CF_3); ^{19}F NMR (282 MHz, DMSO-d_6): δ –77.76 (CF_3); HRMS (m/z): $[M]^+$ calcd. for $C_{25}H_{19}OS$, 367.1156; found, 367.1157.

Methyl 2-((4-methoxyphenyl)thio)benzoate (5a). Under inert atmosphere, tris(dibenzylideneacetone)dipalladium(0) (35 mg, 0.04 mmol) and 1,1'-bis(diphenyl-phosphino)ferrocene (42 mg, 0.08 mmol) were dissolved in toluene (5 ml). Methyl 2-iodobenzoate (0.56 ml, 3.8 mmol), triethylamine (0.58 ml, 4.2 mmol) and 4-methoxythiophenol (0.47 ml, 3.8 mmol) were subsequently added and the mixture was heated to 110 °C for 16 h. After cooling, the reaction was quenched with water and extracted with ethyl acetate. The organic phase was dried ($MgSO_4$), filtered, concentrated under reduced pressure and purified by column chromatography (petrol: ethyl acetate = 100: 0 → 95: 5). The resulting product was triturated with petrol to afford a white solid (0.64 g, 62%). Mp: 82–83 °C; ^{1}H NMR (600 MHz, DMSO-d_6): δ 7.91 (dd, J = 7.9/1.7 Hz, 1H, ph-6*H*), 7.48 (d, J = 8.6 Hz, 2H, ph'-2,6*H*), 7.38 (td, J = 7.7/1.6 Hz, 1H, ph-4*H*), 7.19 (td, J = 7.5/1.2 Hz, 1H, ph-5*H*), 7.08 (d, J = 8.6 Hz, 2H, ph'-3,5*H*), 6.68 (d, J = 7.9 Hz, 1H, ph-3*H*), 3.87 (s, 3H, $COOCH_3$), 3.82 (s, 3H, OCH_3); ^{13}C NMR (150 MHz, DMSO-d_6): δ 166.0 (*C*=O), 160.5 (ph'-4*C*), 143.4 (ph-2*C*), 137.5 (ph'-2,6*C*), 132.8 (ph-4*C*), 130.8 (ph-6*C*), 126.3 (ph-3*C*), 125.8 (ph'-1*C*), 124.4 (ph-5*C*), 121.6 (ph-1*C*), 115.8 (ph'-3,5*C*), 55.4 (OCH_3), 52.3 ($COOCH_3$); HRMS (m/z): $[M]^+$ calcd. for $C_{15}H_{14}O_3S$, 274.0664; found, 274.0663.

(2-(Methoxycarbonyl)phenyl)(4-methoxyphenyl)(phenyl)sulfonium trifluoromethane-sulfonate (5b). Following the general method for preparation of sulfonium salts (page S3), compound **5a** (Scheme S2; 100 mg, 0.36 mmol), chlorobenzene (1 ml), diphenyliodonium trifluoromethanesulfonate (156 mg, 0.36 mmol) and copper(II) benzoate dihydrate (5.9 mg, 0.018 mmol) were reacted at 125 °C for 1 h. Treatment with diethyl ether followed by column chromatography (DCM: methanol = 10: 0 → 9: 1) yielded the product as a colourless oil (136

mg, 76%). ^{1}H NMR (600 MHz, DMSO-d_6): δ 8.36–8.33 (m, 1H, ph''-3*H*), 8.07–7.97 (m, 2H, ph''-4,5*H*), 7.82 (t, J = 7.4 Hz, 1H, ph-4*H*), 7.75 (t, J = 7.8 Hz, 2H, ph-3,5*H*), 7.71–7.68 (m, 4H, ph-2,6*H*, ph'-2,6*H*), 7.33–7.29 (m, 3H, ph'-3,5*H*, ph''-6*H*), 3.88 (s, 3H, COOCH_3), 3.88 (s, 3H, OCH_3); ^{13}C NMR (150 MHz, DMSO-d_6): δ 164.8 (*C*=O), 163.7 (ph'-4*C*), 135.5/134.1 (ph''-4,5*C*), 134.0 (ph'-2,6*C*), 133.8 (ph-4*C*), 132.6 (ph''-3*C*), 131.7 (ph''-6*C*), 131.3 (ph-3,5*C*), 130.7 (ph-2,6*C*), 130.0/127.6 (ph''-1,2*C*), 126.5 (ph-1*C*), 120.7 (q, $^1J_{C,F}$ = 323.0 Hz, CF_3), 116.9 (ph'-3,5*C*), 114.2 (ph'-1*C*), 56.1 (O*C*H$_3$), 53.6 (COO*C*H$_3$); ^{19}F NMR (282 MHz, DMSO-d_6): δ –77.77 (CF_3); HRMS (m/z): $[M]^+$ calcd. for $C_{21}H_{19}O_3S$, 351.1055; found, 351.1052.

4,4'-Dimethoxy diphenyl sulfide (6a). 4-Iodoanisole (0.47 g, 2 mmol), Xantphos (0.06 g, 5%), triethylamine (0.56 ml, 4 mmol) and dioxane (5 ml) were mixed under inert atmosphere. The resulting solution was evacuated and subsequently back flushed with argon, and this procedure was repeated twice. Tris(dibenzylideneacetone)dipalladium(0) (0.05 g, 2.5%) and 4-methoxythiophenol (0.25 ml, 2 mmol) were added, and the resulting brown mixture was heated to 130 °C for 16 h. After cooling, the reaction mixture was diluted with diethyl ether and washed with aqueous NaOH (2 M) and HCl (2 M). The organic phase was dried ($MgSO_4$), filtered, evaporated and purified by column chromatography (petrol: ethyl acetate = 97.5: 2.5 → 92.5: 7.5). The product was isolated as a white solid (0.31 g, 63%). Mp: 45–47 °C; ^{1}H NMR (600 MHz, $CDCl_3$): δ 7.28 (d, J = 8.7 Hz, 4H, ph-2,6*H*), 6.84 (d, J = 8.7 Hz, 4H, ph-3,5*H*), 3.79 (s, 6H, OCH_3); ^{13}C NMR (150 MHz, $CDCl_3$): δ 159.1 (ph-4*C*), 132.9 (ph-2,6*C*), 127.5 (ph-1*C*), 114.9 (ph-3,5*C*), 55.5 (O*C*H$_3$); HRMS (m/z): $[M+H]^+$ calcd. for $C_{14}H_{14}O_2S$, 247.0793; found, 247.0791.

Bis(4-methoxyphenyl)(phenyl)sulfonium trifluoromethanesulfonate (6b). Following the general method for preparation of sulfonium salts (page S3), compound **6a** (Scheme S2; 0.2 g, 0.81 mmol), chlorobenzene (2 ml), diphenyliodonium trifluoromethanesulfonate (0.46 g, 1.06 mmol) and copper(II) benzoate dihydrate (12 mg, 0.04 mmol) were reacted at 125 °C for 1 h. Treatment with diethyl ether followed by column chromatography (DCM: methanol = 10: 0 → 9: 1) yielded the product as an off-white solid (0.37 g, 96%). Mp: 105–107 °C; ^{1}H NMR (600 MHz, $CDCl_3$): δ 7.72–7.64 (m, 7H, ph-3–5*H*, ph'-2,6*H*), 7.59 (d, J = 7.9 Hz, 2H, ph-2,6*H*), 7.17 (d, J = 9.1 Hz, 4H, ph'-3,5*H*), 3.89 (s, 6H, O*C*H$_3$); ^{13}C NMR (150 MHz, $CDCl_3$) *δ*: 164.7 (ph'-4*C*), 134.1 (ph-4*C*), 133.3 (ph'-2,6*C*), 131.5 (ph-3,5*C*), 130.2 (ph-2,6*C*), 126.5 (ph-1*C*), 121.0 (q, $^1J_{C,F}$ = 320.0 Hz, CF_3), 117.4 (ph'-3,5*C*), 114.1 (ph'-1*C*), 56.3 (O*C*H$_3$); ^{19}F NMR (282 MHz, DMSO-d_6): δ –78.16 (CF_3); HRMS (m/z): $[M]^+$ calcd. for $C_{20}H_{19}O_2S$, 323.1106; found, 323.1103.

(2-Methoxyphenyl)(4-methoxyphenyl)sulfane (7a). Under inert atmosphere, tris(dibenzylideneacetone)dipalladium(0) (10 mg, 0.01 mmol) and DPEPhos (11 mg, 0.02 mmol) were suspended in toluene (5 ml) and stirred for 5 min at room temperature. 2-Iodoanisole (0.13 ml, 1 mmol), 4-methoxythiophenol (0.12 ml, 1 mmol) and potassium *tert*-butoxide (0.12 g, 1.1 mmol) dissolved in toluene (3 ml) were subsequently added. The brown solution was heated to 110 °C for 2 h. After cooling, the reaction mixture was filtered over a plug of Celite® and the filtrate was concentrated to dryness. The resulting orange oil was purified by column chromatography (petrol: DCM = 80: 20 → 55: 45) to afford the product as a colorless oil (0.24 g, 96%). ^{1}H NMR (600 MHz, $CDCl_3$): δ 7.44 (d, J = 8.8 Hz, 2H, ph-2,6*H*), 7.13 (ddd, J = 8.1/7.3/1.8 Hz, 1H, ph'-4*H*), 6.93 (d, J = 9.0 Hz, 2H, ph-3,5*H*), 6.86 (dd, J = 8.2/1.1 Hz, 1H, ph'-3*H*), 6.81 (td, J = 7.5/1.1 Hz, 1H, ph'-5*H*), 6.77 (dd, J = 7.7/1.8 Hz, 1H, ph'-6*H*), 3.91 (s, 3H, 2-OCH_3), 3.84 (s, 3H, 4-OCH_3); ^{13}C NMR (150 MHz, $CDCl_3$): δ 160.1 (ph-4*C*), 155.7 (ph'-2*C*), 136.3 (ph-2,6*C*), 128.1 (ph'-6*C*), 127.5 (ph-1*C*), 126.7 (ph'-4*C*),

122.8 (ph'-1*C*), 121.3 (ph'-5*C*), 115.2 (ph-3,5*C*), 110.4 (ph'-3*C*), 56.0 (2-O*C*H3), 55.5 (4-O*C*H3); HRMS (m/z): $[M]^+$ calcd. for $C_{14}H_{14}O_2S$, 246.0715; found, 246.0710.

(2-Methoxyphenyl)(4-methoxyphenyl)(phenyl)sulfonium trifluoromethanesulfonate (7b). Following the general method for preparation of sulfonium salts (page S3), compound **7a** (Scheme S2; 0.18 g, 0.73 mmol), chlorobenzene (2 ml), diphenyliodonium trifluoromethanesulfonate (0.41 g, 0.95 mmol) and copper(II) benzoate dihydrate (15 mg, 0.05 mmol) were reacted at 125 °C for 1 h. Treatment with diethyl ether followed by column chromatography (DCM: methanol = 10: 0 → 9: 1) yielded the product as a colourless oil (0.3 g, 86%). ^{1}H NMR (600 MHz, DMSO-*d*6): δ 7.86–7.83 (m, 2H, ph-4*H*, ph''-4*H*), 7.78–7.73 (m, 6H, ph-2,3,5,6*H*, ph'-2,6*H*), 7.48 (d, J = 7.9 Hz, 1H, ph''-3*H*), 7.33 (d, J = 9.1 Hz, 2H, ph'-3,5*H*), 7.29 (t, J = 7.7 Hz, 1H, ph''-5*H*), 7.02 (dd, J = 8.0/1.5 Hz, 1H, ph''-6*H*), 3.89 (s, 3H, 2-OC*H3*), 3.88 (s, 3H, 4-OC*H3*); ^{13}C NMR (150 MHz, DMSO-*d*6): δ 163.9 (ph'-4*C*), 157.5 (ph''-2*C*), 136.3 (ph''-4*C*), 134.1 (ph-4*C*), 133.8 (ph'-2,6*C*), 131.4 (ph-3,5*C*), 130.6 (ph-2,6*C*), 130.4 (ph''-6*C*), 124.8 (ph-1*C*), 123.0 (ph''-5*C*), 120.7 (q, $^1J_{C,F}$ = 321.0 Hz, *C*F3), 117.1 (ph'-3,5*C*), 114.0 (ph''-3*C*), 112.8 (ph''-1*C*), 112.4 (ph'-1*C*), 57.4 (2-O*C*H3), 56.2 (4-O*C*H3); ^{19}F NMR (282 MHz, DMSO-*d*6): δ –77.77 (C*F3*); HRMS (m/z): $[M]^+$ calcd. for $C_{20}H_{19}O_2S$, 323.1106; found, 323.1115.

d. Compounds 9b and 10b

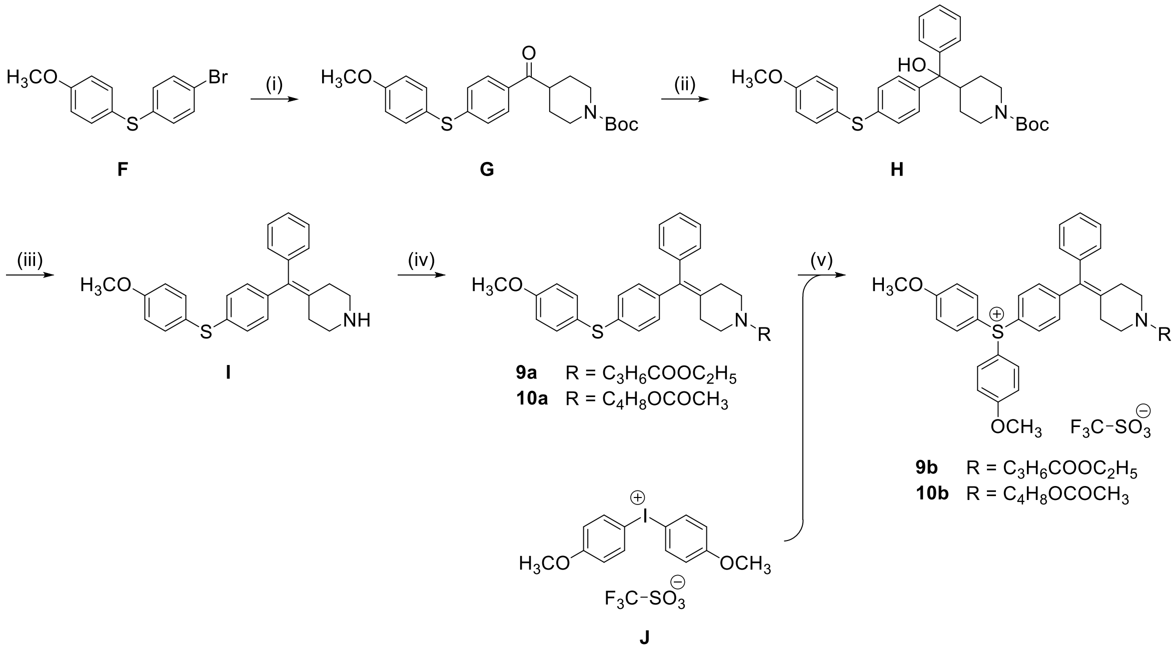

Scheme S3. Synthesis of the sulfonium salts **9b** and **10b**

Reagents and conditions: (i) 1. *n*-BuLi, THF, –78 °C, 30 min; 2. **B**, THF, –78 °C – 0 °C, 30 min; (ii) PhMgBr, THF, 0 °C – rt, 3 h; (iii) TFA, DCM, 0 °C – rt, 2 h; (iv) alkyl bromide, K_2CO_3, acetone, 60 °C – rt, 15 h; (v) Cu(II) benzoate, TFSA, chlorobenzene, 125 °C, 1 h.

(4-Bromophenyl)(4-methoxyphenyl)sulfane (F). Under inert atmosphere, a solution of 1-bromo-4-iodobenzene (5.6 g, 20 mmol), 4-methoxybenzenethiol (3 ml, 24 mmol), potassium hydroxide (2.8 g, 50 mmol), copper(I) iodide (0.2 g, 1 mmol), and sarcosine (0.36 g, 4 mmol) in anhydrous dioxane (30 ml) was heated to 100 °C for 24 h. After cooling, the crude product was filtered over a plug of silica and eluted with ethyl acetate. Trituration of the resulting oil with petrol led to precipitation of the product as a white solid (2.9 g, 49%). ^{1}H NMR (600 MHz, CDCl3): δ 7.41 (d, J = 8.7 Hz, 2H, ph'-2,6*H*), 7.33 (d, J = 8.5 Hz, 2H, ph-2,6*H*), 7.00 (d, J = 8.5, 2H, ph-3,5*H*), 6.91 (d, J = 8.7 Hz, 2H, ph'-3,5*H*), 3.83 (s, 3H, OC*H3*); ^{13}C NMR (150 MHz,

$CDCl_3$): δ 160.2 (ph'-4*C*), 138.3 (ph-4*C*), 135.8 (ph'-2,6*C*), 132.0 (ph-2,6*C*), 129.5 (ph-3,5*C*), 123.6 (ph'-1*C*), 119.5 (ph-1*C*), 115.3 (ph'-3,5*C*), 55.5 (O*C*H_3); HRMS (m/z): $[M]^+$ calcd. for $C_{13}H_{11}BrOS$, 293.9714; found, 293.9709.

***tert*-Butyl 4-(4-((4-methoxyphenyl)thio)benzoyl)piperidine-1-carboxylate (G).** A solution of compound **F** (Scheme S3; 2.34 g, 7.9 mmol) in THF (5 ml) was cooled to –78 °C and *n*-butyl lithium (2.5 M in hexane; 2.9 ml, 7.2 mmol) was added dropwise. After stirring for 30 minutes at –78 °C, a solution of compound **B** (Scheme S1; 1.96 g, 7.2 mmol) in THF (5 ml) was added. The mixture was allowed to come to 0 °C during 1 h. It was subsequently quenched with a saturated solution of ammonium chloride, extracted with ethyl acetate and purified by column chromatography (petrol: ethyl acetate = 9: 1 → 7: 3) to afford the product as a colourless oil (1.5 g, 50%). ^1H NMR (600 MHz, DMSO-d_6): δ 7.88 (d, J = 8.5 Hz, 2H, ph-2,6*H*), 7.51 (d, J = 8.8 Hz, 2H, ph'-2,6*H*), 7.12 (d, J = 8.5 Hz, 2H, ph-3,5*H*), 7.1 (d, J = 8.7 Hz, 2H, ph'-3,5*H*), 3.95 (br s, 2H, pip-2,6H^{eq}), 3.82 (s, 3H, OCH_3), 3.55–3.50 (m, 1H, pip-4*H*), 2.86 (br s, 2H, pip-2,6H^{ax}), 1.71 (d, J = 12.8 Hz, 2H, pip-3,5H^{eq}), 1.39–1.33 (m, 11H, C(CH_3)$_3$, pip-3,5H^{ax}); ^{13}C NMR (150 MHz, DMSO-d_6): δ 200.9 (*C*=O^{ketone}), 160.5 (ph'-4*C*), 153.9 (*C*=O^{boc}), 145.9 (ph-4*C*), 136.8 (ph'-2,6*C*), 132.3 (ph-1*C*), 129.1 (ph-2,6*C*), 125.7 (ph-3,5*C*), 120.2 (ph'-1*C*), 115.8 (ph'-3,5*C*), 78.7 (*C*(CH_3)$_3$), 55.5 (O*C*H_3), 43.3/42.4 (pip-2,6*C*), 42.2 (pip-4*C*), 28.2 (pip-3,5*C*), 28.1 (C(*C*H_3)$_3$); HRMS (m/z): $[M]^+$ calcd. for $C_{24}H_{29}NO_4S$, 427.1817; found, 427.1810.

***tert*-Butyl 4-(hydroxy(4-((4-methoxyphenyl)thio)phenyl)(phenyl)methyl)piperidine-1-carboxylate (H).** A solution of compound **G** (Scheme S3; 1.1 g, 2.6 mmol) in THF (5 ml) was added dropwise to a solution of phenylmagnesium bromide (1 M in THF; 5.1 ml, 5.1 mmol) kept at 0 °C. The mixture was allowed to come to room temperature and subsequently heated at 60 °C for 3 h. After cooling, the reaction was quenched with brine. The product was extracted with ethyl acetate, purified by column chromatography (DCM: methanol = 10: 0 → 9.9: 0.1) and isolated as a colourless oil (0.8 g, 62%). ^1H NMR (600 MHz, DMSO-d_6): δ 7.48 (d, J = 7.7 Hz, 2H, ph-2,6*H*), 7.43 (d, J = 8.4 Hz, 2H, ph'-2,6*H*), 7.39 (d, J = 8.6 Hz, 2H, ph''-2,6*H*), 7.25 (t, J = 7.7 Hz, 2H, ph-3,5*H*), 7.12 (t, J = 7.3 Hz, 1H, ph-4*H*), 7.03 (d, J = 8.5 Hz, 2H, ph'-3,5*H*), 6.98 (d, J = 8.7 Hz, 2H, ph''-3,5*H*), 5.31 (s, 1H, CO*H*), 3.93 (br s, 2H, pip-2,6H^{eq}), 3.76 (s, 3H, OCH_3), 2.70–2.61 (m, 3H, pip-4*H*, pip-2,6H^{ax}), 1.36 (s, 9H, C(CH_3)$_3$), 1.29–1.23 (m, 4H, pip-3,5H_2); ^{13}C NMR (150 MHz, DMSO-d_6): δ 159.6 (ph''-4*C*), 153.7 (*C*=O), 146.8 (ph-1*C*), 145.3 (ph'-1*C*), 135.2 (ph''-2,6*C*), 135.0 (ph'-4*C*), 127.9 (ph-3,5*C*), 127.5 (ph'-3,5*C*), 126.7 (ph'-2,6*C*), 126.0 (ph-4*C*), 125.6 (ph-2,6*C*), 123.2 (ph''-1*C*), 115.4 (ph''-3,5*C*), 78.4/78.4 (*C*OH, *C*(CH_3)$_3$), 55.3 (O*C*H_3), 44.0/43.1 (pip-2,6*C*, pip-4*C*), 28.1 (C(*C*H_3)$_3$), 26.1 (pip-3,5*C*); HRMS (m/z): $[M]^+$ calcd. for $C_{30}H_{35}NO_4S$, 505.2287; found, 505.2281.

4-((4-((4-Methoxyphenyl)thio)phenyl)(phenyl)methylene)piperidine (I). Compound **H** (Scheme S3; 0.8 g, 1.6 mmol) was dissolved in DCM (20 ml). TFA (1 ml) was added and the solution was stirred for 1 h at room temperature. The mixture was made basic by addition of aqueous NaOH (2 M), and the organic phase was dried ($MgSO_4$) and concentrated under reduced pressure. The product was obtained as a light yellow oil (0.54 g, 87%). ^1H NMR (600 MHz, DMSO-d_6): δ 7.42 (d, J = 8.6 Hz, 2H, ph''-2,6*H*), 7.29 (t, J = 7.6 Hz, 2H, ph-3,5*H*), 7.19 (t, J = 7.4 Hz, 1H, ph-4*H*), 7.06–7.03 (m, 4H, ph-2,6*H*, ph'-2,6*H*), 7.01–6.99 (m, 4H, ph'-3,5*H*, ph''-3,5*H*), 3.77 (s, 3H, OCH_3), 2.70 (br s, 4H, pip-2,6H_2), 2.14–2.11 (m, 4H, pip-3,5H_2); ^{13}C NMR (150 MHz, DMSO-d_6): δ 159.8 (ph''-4*C*), 142.1 (ph-1*C*), 140.0 (ph'-1*C*), 137.2 (*C*=C^{pip}), 135.8 (ph'-4*C*), 135.6 (ph''-2,6*C*), 133.8 (C=C^{pip}), 130.4 (ph'-3,5*C*), 129.5 (ph-2,6*C*), 128.2 (ph-3,5*C*), 127.2 (ph'-2,6*C*), 126.4 (ph-4*C*), 122.7 (ph''-1*C*), 115.4 (ph''-3,5*C*), 55.3 (O*C*H_3), 48.1 (pip-2,6*C*), 33.2 (pip-3,5*C*); HRMS (m/z): $[M]^+$ calcd. for $C_{25}H_{25}NOS$, 387.1657; found, 387.1641.

Ethyl 4-(4-((4-((4-methoxyphenyl)thio)phenyl)(phenyl)methylene)piperidin-1-yl)butanoate (9a). Under inert atmosphere, compound **I** (Scheme S3; 0.14 g, 0.36 mmol), ethyl 4-bromobutanoate (0.1 ml, 0.72 mmol) and potassium carbonate (0.2 g, 1.44 mmol) were dissolved in anhydrous acetone (10 ml). After heating at 60 °C for 3 h the mixture was stirred at room temperature for a further 12 h. The inorganic compounds were filtered off and the crude mixture purified by column chromatography (DCM: methanol = 100: 0 → 98: 2). The product was isolated as a colourless oil (0.09 g, 50%). ^{1}H NMR (600 MHz, DMSO-d_6): δ 7.42 (d, J = 8.5 Hz, 2H, ph''-2,6*H*), 7.29 (t, J = 7.5 Hz, 2H, ph-3,5*H*), 7.20 (t, J = 7.3 Hz, 1H, ph-4*H*), 7.06–7.03 (m, 4H, ph-2,6*H*, ph'-2,6*H*), 7.01–6.98 (m, 4H, ph'-3,5*H*, ph''-3,5*H*), 4.03 (q, J = 7.1 Hz, 2H, ethyl-C*H*$_2$), 3.77 (s, 3H, OC*H*$_3$), 2.37 (br s, 4H, pip-2,6*H*$_2$), 2.29–2.25 (m, 4H, but-2,4*H*$_2$), 2.22–2.19 (m, 4H, pip-3,5*H*$_2$), 1.68–1.64 (m, 2H, but-3*H*$_2$), 1.16 (t, J = 7.1 Hz, 3H, ethyl-C*H*$_3$); ^{13}C NMR (150 MHz, DMSO-d_6): δ 172.9 (*C*=O), 159.8 (ph''-4*C*), 141.9 (ph-1*C*), 139.8 (ph'-1*C*), 136.0 (ph'-4*C*), 135.95 (*C*=C^{pip}), 135.7 (ph''-2,6*C*), 134.3 (C=*C*^{pip}), 130.3 (ph'-3,5*C*), 129.5 (ph-2,6*C*), 128.2 (ph-3,5*C*), 127.2 (ph'-2,6*C*), 126.5 (ph-4*C*), 122.6 (ph''-1*C*), 115.4 (ph''-3,5*C*), 59.7 (ethyl-*C*H$_2$), 56.8 (but-4*C*), 55.3 (O*C*H$_3$), 54.6 (pip-2,6*C*), 31.6 (pip-3,5*C*), 31.3 (but-2*C*), 22.0 (but-3*C*), 14.2 (ethyl-*C*H$_3$); HRMS (m/z): [M+H]$^+$ calcd. for $C_{31}H_{35}NO_3S$, 502.2416; found, 502.2410.

Bis(4-methoxyphenyl)iodonium trifluoromethanesulfonate (J). To a solution of iodine (0.52 g, 2.0 mmol) and 3-chloroperbenzoic acid (0.8 g, 4.6 mmol) in DCM (15 ml) were added anisole (0.68 g, 6.2 mmol) and *para*-toluenesulfonic acid monohydrate (1.2 g, 6.3 mmol), and the mixture was stirred for 15 min at 40 °C. The red solution was cooled to 0 °C (ice bath) and trifluoromethanesulfonic acid (0.35 ml, 4 mmol) was added. The red slurry was stirred for 1 h at room temperature, diluted with DCM and purified by column chromatography (DCM: diethyl ether = 2: 1, DCM, DCM: methanol = 96: 4). The product was obtained as a light brown oil (1.2 g, 80%). ^{1}H NMR (600 MHz, DMSO-d_6): δ 8.12 (d, J = 9.0 Hz, 4H, ph-2,6*H*), 7.06 (d, J = 9.0 Hz, 4H, ph-3,5*H*), 3.79 (s, 6H, OC*H*$_3$); ^{13}C NMR (150 MHz, DMSO-d_6): δ 161.8 (ph-4*C*), 136.9 (ph-2,6*C*), 120.7 (q, $^{1}J_{C,F}$ = 321.3 Hz, *C*F$_3$), 117.4 (ph-3,5*C*), 106.2 (ph-1*C*), 55.7 (O*C*H$_3$); ^{19}F NMR (282 MHz, DMSO-d_6): δ –78.20 (C*F*$_3$); HRMS (m/z): [M]$^+$ calcd. for $C_{14}H_{14}IO_2$, 341.0039; found, 341.0031.

(4-((1-(4-Ethoxy-4-oxobutyl)piperidin-4-ylidene)(phenyl)methyl)phenyl)bis(4-methoxyphenyl)sulfonium trifluoromethanesulfonate (9b). Following the general method for preparation of sulfonium salts (page S3), compound **9a** (Scheme S3; 90 mg, 0.18 mmol), chlorobenzene (1.0 ml), TFSA (16 µl, 0.18 mmol), compound **J** (Scheme S3; 90 mg, 0.18 mmol) and copper(II) benzoate dihydrate (3 mg, 0.01 mmol) were reacted at 125 °C for 1 h. Treatment with diethyl ether, column chromatography (DCM: methanol = 10: 0 → 9: 1) and extractive workup yielded the product as a light yellow oil (50 mg, 37%). ^{1}H NMR (600 MHz, DMSO-d_6): δ 7.78 (d, J = 9.1 Hz, 4H, ph-2,6*H*), 7.64 (d, J = 8.1 Hz, 2H, ph'-3,5*H*), 7.46 (d, J = 8.1 Hz, 2H, ph'-2,6*H*), 7.35 (t, J = 7.4 Hz, 2H, ph''-3,5*H*), 7.31 (d, J = 9.1 Hz, 4H, ph-3,5*H*), 7.26 (t, J = 7.1 Hz, 1H, ph''-4*H*), 7.12 (d, J = 7.1 Hz, 2H, ph''-2,6*H*), 4.04 (q, J = 7.1 Hz, 2H, ethyl-C*H*$_2$), 3.87 (s, 6H, OC*H*$_3$), 2.41–2.24 (m, 12H, pip-2,3,5,6*H*$_2$, but-1,3*H*$_2$), 1.68 (br s, 2H, but-2*H*$_2$), 1.17 (t, J = 7.1 Hz, 3H, ethyl-C*H*$_3$); ^{13}C NMR (150 MHz, DMSO-d_6): δ 172.9 (*C*=O), 163.8 (ph-4*C*), 147.3 (ph''-1*C*), 141.0 (ph'-4*C*), 138.3 (*C*=C^{pip}), 133.4 (ph-2,6*C*), 133.2 (C=*C*^{pip}), 131.9 (ph'-3,5*C*), 130.2 (ph'-2,6*C*), 129.5 (ph''-2,6*C*), 128.5 (ph''-3,5*C*), 127.0 (ph''-4*C*), 124.6 (ph'-1*C*), 120.7 (q, $^{1}J_{C,F}$ = 322.8 Hz, *C*F$_3$), 116.9 (ph-3,5*C*), 115.2 (ph-1*C*), 59.8 (ethyl-*C*H$_2$), 56.7 (but-1*C*), 56.2 (OC*H*$_3$), 54.4 (pip-2,6*C*), 31.8 (but-3*C*), 31.3 (pip-3,5*C*), 22.0 (but-2*C*), 14.2 (ethyl-*C*H$_3$). ^{19}F NMR (282 MHz, DMSO-d_6): δ –78.05 (C*F*$_3$); HRMS (m/z): [M]$^+$ calcd. for $C_{38}H_{42}NO_4S$, 608.2662; found, 608.2683.

4-(4-((4-((4-Methoxyphenyl)thio)phenyl)(phenyl)methylene)piperidin-1-yl)butyl acetate (10a). Under inert atmosphere, compound **I** (Scheme S3; 0.54 g, 1.4 mmol), 4-bromobutyl acetate (0.35 ml, 2.8 mmol) and potassium carbonate (0.77 g, 5.6 mmol) were dissolved in anhydrous acetone (10 ml). After heating at 60 °C for 3 h, the mixture was stirred at room temperature for a further 12 h. The inorganic compounds were filtered off and the crude mixture purified by column chromatography (DCM: methanol = 100: 0 → 95: 5) to afford the product as a colourless oil (0.48 g, 69%). ^{1}H NMR (600 MHz, DMSO-d_6): δ 7.42 (d, J = 8.7 Hz, 2H, ph''-2,6*H*), 7.29 (t, J = 7.6 Hz, 2H, ph-3,5*H*), 7.21 (t, J = 7.4 Hz, 1H, ph-4*H*), 7.06–7.03 (m, 4H, ph-2,6*H*, ph'-2,6*H*), 7.01–6.99 (m, 4H, ph'-3,5*H*, ph''-3,5*H*), 3.99 (q, J = 6.6 Hz, 2H, but-1H_2), 3.77 (s, 3H, OCH_3), 2.37 (br s, 4H, pip-2,6H_2), 2.27 (t, J = 6.9 Hz, 2H, but-4H_2), 2.23–2.20 (m, 4H, pip-3,5H_2), 1.98 (s, 3H, COCH_3), 1.59–1.53 (m, 2H, but-2H_2), 1.47–1.42 (m, 2H, but-3H_2); ^{13}C NMR (150 MHz, DMSO-d_6): δ 170.5 (*C*=O), 159.8 (ph''-4*C*), 141.9 (ph-1*C*), 139.8 (ph'-1*C*), 136.0 (ph'-4*C*), 135.7 (ph''-2,6*C*), 134.3 (C=C^{pip}), 130.3 (ph'-3,5*C*), 129.5 (ph-2,6*C*), 128.2 (ph-3,5*C*), 127.2 (ph'-2,6*C*), 126.5 (ph-4*C*), 122.6 (ph''-1*C*), 115.4 (ph''-3,5*C*), 63.8 (but-1*C*), 57.2 (but-4*C*), 55.3 (O*C*H3), 54.6 (pip-2,6*C*), 31.3 (pip-3,5*C*), 26.2 (but-2*C*), 22.9 (but-3*C*), 20.8 (CO*C*H3), n.d. (*C*=C^{pip}); HRMS (m/z): $[M+H]^+$ calcd. for $C_{31}H_{35}NO_3S$, 502.2416; found, 502.2402.

(4-((1-(4-Acetoxybutyl)piperidin-4-ylidene)(phenyl)methyl)phenyl)bis(4-methoxyphenyl)sulfonium trifluoromethanesulfonate (10b). Following the general method for preparation of sulfonium salts (page S3), compound **10a** (Scheme S3; 64 mg, 0.13 mmol), chlorobenzene (0.5 ml), TFSA (11.5 µl, 0.13 mmol), compound **J** (Scheme S3; 64 mg, 0.13 mmol) and copper(II) benzoate dihydrate (2 mg, 0.007 mmol) were reacted at 125 °C for 1 h. Treatment with diethyl ether, column chromatography (DCM: methanol = 10: 0 → 9: 1) and extractive workup yielded the product as a light yellow oil (41 mg, 41%). ^{1}H NMR (600 MHz, DMSO-d_6): δ 7.78 (d, J = 9.1 Hz, 4H, ph-2,6*H*), 7.64 (d, J = 8.3 Hz, 2H, ph'-3,5*H*), 7.46 (d, J = 8.4 Hz, 2H, ph'-2,6*H*), 7.35 (t, J = 7.5 Hz, 2H, ph''-3,5*H*), 7.31 (d, J = 9.1 Hz, 4H, ph-3,5*H*), 7.26 (t, J = 7.3 Hz, 1H, ph''-4*H*), 7.11 (d, J = 7.3 Hz, 2H, ph''-2,6*H*), 4.00 (t, J = 6.5 Hz, 2H, but-4H_2), 3.87 (s, 6H, OCH_3), 2.41 (br s, 4H, pip-2,6H_2), 2.28–2.25 (m, 6H, pip-3,5H_2, but-1H_2), 1.99 (s, 3H, COCH_3), 1.57 (br s, 2H, but-3H_2), 1.46 (br s, 2H, but-2H_2); ^{13}C NMR (150 MHz, DMSO-d_6): δ 170.5 (*C*=O), 163.7 (ph-4*C*), 147.4 (ph''-1*C*), 141.0 (ph'-4*C*), 138.3 (*C*=C^{pip}), 133.4 (ph-2,6*C*), 133.2 (C=C^{pip}), 131.9 (ph'-3,5*C*), 130.2 (ph'-2,6*C*), 129.5 (ph''-2,6*C*), 128.5 (ph''-3,5*C*), 127.0 (ph''-4*C*), 124.5 (ph'-1*C*), 120.7 (q, $^{1}J_{C,F}$ = 323.0 Hz, *C*F3), 116.9 (ph-3,5*C*), 115.2 (ph-1*C*), 63.7 (but-4*C*), 57.1 (but-1*C*), 56.2 (O*C*H_3), 54.5 (pip-2,6*C*), 31.3 (pip-3,5*C*), 26.2 (but-3*C*), 22.9 (but-2*C*), 20.8 (CO*C*H3); ^{19}F NMR (282 MHz, DMSO-d_6): δ –78.05 (CF_3); HRMS (m/z): $[M]^+$ calcd. for $C_{38}H_{42}NO_4S$, 608.2835; found, 608.2829.

e. Compound 11b

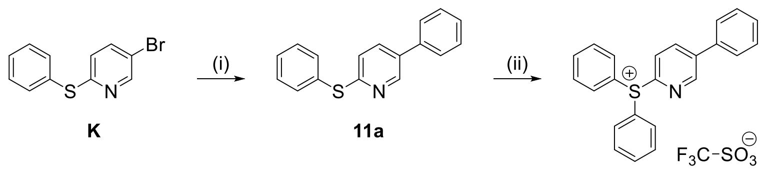

Scheme S4. Synthesis of the sulfonium salt **11b**

Reagents and conditions: (i) $PhB(OH)_2$, $Pd(PPh_3)_4$, Na_2CO_3, H_2O, CH_3CN, 90 °C, 3 h; (ii) $(Ph)_2I^+ \cdot CF_3O_3S^-$, Cu(II) benzoate, chlorobenzene, 125 °C, 2 h.

5-Bromo-2-(phenylthio)pyridine (K). Under inert atmosphere, a suspension of 5-bromo-2-iodopyridine (2 g, 7.0 mmol), thiophenol (0.8 ml, 7.75 mmol), copper(I) iodide (65 mg, 0.35

mmol), sarcosine (126 mg, 1.4 mmol) and potassium hydroxide (0.87 g, 15.5 mmol) in DMF (3 ml) was heated at 110 °C for 12 h. After cooling, the reaction mixture was diluted with ethyl acetate and filtered over a plug of silica. The filtrate was concentrated under reduced pressure and purified by column chromatography (petrol: ethyl acetate = 10: 0 → 95: 5) to afford the product as a colourless oil (1.4 g, 77%). ^{1}H NMR (600 MHz, $CDCl_3$): δ 8.47 (d, J = 2.4 Hz, 1H, pyr-6*H*), 7.59–7.57 (m, 2H, ph-3,5*H*), 7.55 (dd, J = 8.6/2.3 Hz, 1H, pyr-4*H*), 7.45–7.42 (m, 3H, ph-2,4,6*H*), 6.77 (d, J = 8.6 Hz, 1H, pyr-3*H*); ^{13}C NMR (150 MHz, $CDCl_3$): δ 160.5 (pyr-2*C*), 150.5 (pyr-6*C*), 139.3 (pyr-4*C*), 135.2 (ph-3,5*C*), 130.6 (ph-1*C*), 129.9 (ph-2,6*C*), 129.6 (ph-4*C*), 122.6 (pyr-3*C*), 116.6 (pyr-5*C*); HRMS (m/z): $[M+H]^+$ calcd. for $C_{11}H_8BrNS$, 265.9639; found, 265.9630.

5-Phenyl-2-(phenylthio)pyridine (11a). Compound **K** (Scheme S4; 0.53 g, 2 mmol) and phenylboronic acid (0.24 g, 2 mmol) were dissolved in a mixture of acetonitrile (10 ml) and aqueous sodium carbonate (0.4 M; 10 ml). Tetrakis(triphenylphosphine)palladium(0) (0.12 g, 0.1 mmol) was subsequently added, and the mixture was heated at 90 °C for 3 h. After cooling, the organic solvent was removed under reduced pressure. The aqueous solution was diluted with water and extracted with DCM. The organic phase was dried ($MgSO_4$), filtered and concentrated to dryness. The crude mixture was purified by column chromatography (petrol: ethyl acetate = 10: 0 → 95: 5) to afford the product as a colourless oil (0.32 g, 61%). ^{1}H NMR (600 MHz, $CDCl_3$): δ 8.67 (d, J = 2.3 Hz, 1H, pyr-6*H*), 7.66 (dd, J = 8.3/2.3 Hz, 1H, pyr-4*H*), 7.64–7.62 (m, 2H, ph-3,5*H*), 7.52 (d, J = 7.2 Hz, 2H, ph'-2,6*H*), 7.47–7.42 (m, 5H, ph-2,4,6*H*, ph'-3,5*H*), 7.38 (t, J = 7.3 Hz, 1H, ph'-4*H*), 6.97 (d, J = 8.3 Hz, 1H, pyr-3*H*); ^{13}C NMR (150 MHz, $CDCl_3$): δ 160.4 (pyr-2*C*), 148.1 (pyr-6*C*), 137.5 (ph'-1*C*), 135.3 (pyr-4*C*), 135.1 (ph-3,5*C*), 133.2 (pyr-5*C*), 131.2 (ph-1*C*), 129.8 (ph-2,6*C*), 129.3 (ph-4*C*), 129.2 (ph'-3,5*C*), 128.1 (ph'-4*C*), 127.0 (ph'-2,6*C*), 121.5 (pyr-3*C*); HRMS (m/z): $[M+H]^+$ calcd. for $C_{17}H_{13}NS$, 264.0847; found, 264.0851.

Diphenyl(5-phenylpyridin-2-yl)sulfonium trifluoromethanesulfonate (11b). Following the general method for preparation of sulfonium salts (page S3), compound **11a** (Scheme S4; 0.1 g, 0.38 mmol), chlorobenzene (1 ml), diphenyliodonium trifluoromethanesulfonate (0.16 g, 0.38 mmol) and copper(II) benzoate dihydrate (6 mg, 0.02 mmol) were reacted at 125 °C for 1 h. Treatment with diethyl ether followed by column chromatography (DCM: methanol = 10: 0 → 9.5: 0.5) yielded the product as a colourless oil (0.06 g, 34%). ^{1}H NMR (600 MHz, $CDCl_3$): δ 8.94 (d, J = 2.6 Hz, 1H, pyr-6*H*), 8.58 (d, J = 8.5 Hz, 1H, pyr-3*H*), 8.25 (dd, J = 8.2/2.3 Hz, 1H, pyr-4*H*), 7.97 (d, J = 8.0 Hz, 4H, ph-2,6*H*), 7.74 (t, J = 7.4 Hz, 2H, ph-4*H*), 7.67 (t, J = 7.7 Hz, 4H, ph-3,5*H*), 7.60 (d, J = 6.7 Hz, 2H, ph'-2,6*H*), 7.54–7.48 (m, 3H, ph'-3–5*H*); ^{13}C NMR (150 MHz, $CDCl_3$): δ 150.8 (pyr-6*C*), 144.6 (pyr-2*C*), 141.8 (ph'-1*C*), 138.4 (pyr-4*C*), 135.0 (pyr-5*C*), 134.7 (ph-4*C*), 131.9 (ph-2,6*C*), 131.4 (ph-3,5*C*), 130.6 (pyr-3*C*), 130.1 (ph'-4*C*), 129.7 (ph'-3,5*C*), 127.7 (ph'-2,6*C*), 125.1 (ph-1*C*), 121.0 (q, $^{1}J_{C,F}$ = 320.4 Hz, *C*F_3); ^{19}F NMR (282 MHz, DMSO-d_6): δ –70.61 (CF_3); HRMS (m/z): $[M+H]^+$ calcd. for $C_{23}H_{18}NS$, 340.1160; found, 340.1162.

f. Compound 12b

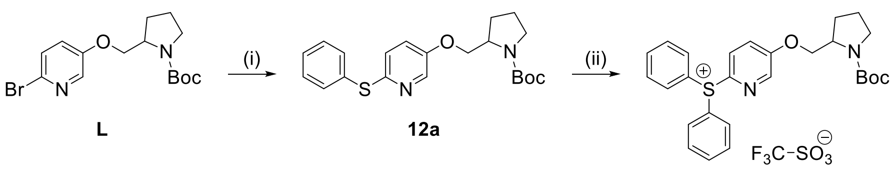

Scheme S5. Synthesis of the sulfonium salt **12b**

Reagents and conditions: (i) Thiophenol, $Pd_2(dba)_3$, DPEPhos, ^{t}ButOK, toluene, 120 °C, 2 h; (ii) $(Ph)_2I^+ \cdot CF_3SO_3^-$, Cu(II) benzoate, chlorobenzene, 125 °C, 2 h.

***tert*-Butyl 2-(((6-bromopyridin-3-yl)oxy)methyl)pyrrolidine-1-carboxylate (L).** Under inert atmosphere and under cooling (–10 °C), diisopropyl azodicarboxylate (1.47 ml, 7.5 mmol) was added dropwise to a solution of triphenylphosphine (2 g, 7.5 mmol) in THF (15 ml). After stirring at –10 °C for 30 min, a solution of Boc-L-prolinol (1 g, 5 mmol) and 2-bromo-5-hydroxypyridine (1.3 g, 7.5 mmol) in THF (5 ml) was added dropwise, and the resulting solution was stirred at –10 °C for 1 h, at 0 °C for 6 h and at room temperature for 12 h. The mixture was concentrated under reduced pressure and treated with a mixture of petrol and diethyl ether (1: 1). A precipitate formed and was subsequently removed by filtration. The filtrate was concentrated *in vacuo* and purified by column chromatography (petrol: ethyl acetate = 93: 7 → 8: 2) to afford the product as a colourless oil (1.5 g, 84%). ^{1}H NMR (400 MHz, DMSO-d_6, 80 °C): δ 8.13 (d, J = 3.2 Hz, 1H, pyr-2*H*), 7.50 (d, J = 8.6 Hz, 1H, pyr-5*H*), 7.39 (dd, J = 8.8/3.2 Hz, 1H, pyr-4*H*), 4.18–4.01 (m, 3H, pyrr-2*H*, OCH_2), 3.37–3.24 (m, 2H, pyrr-5H_2), 2.06–1.77 (m, 4H, pyrr-3,4H_2), 1.41 (s, 9H, C(CH_3)$_3$); ^{13}C NMR, *major rotational isomer* (150 MHz, DMSO-d_6): δ 154.8 (pyr-3*C*), *153.8* (*C*=O), *138.0* (pyr-2*C*), *131.3* (pyr-6*C*), 128.3 (pyr-5*C*), *125.5* (pyr-4*C*), *78.7* (*C*(CH$_3$)$_3$), *68.4* (O*C*H$_2$), *55.4* (pyrr-2*C*), *46.6* (pyrr-5*C*), *28.1* (C(*C*H$_3$)$_3$), *27.6* (pyrr-3*C*), *23.3* (pyrr-4*C*); ^{13}C NMR, *minor rotational isomer* (150 MHz, DMSO-d_6): δ 154.8 (pyr-3*C*), *153.4* (*C*=O), *137.9* (pyr-2*C*), *131.4* (pyr-6*C*), 128.3 (pyr-5*C*), *125.6* (pyr-4*C*), *78.8* (*C*(CH$_3$)$_3$), *69.1* (O*C*H$_2$), *55.3* (pyrr-2*C*), *46.3* (pyrr-5*C*), *28.3* (pyrr-3*C*), *28.09* (C(*C*H$_3$)$_3$), *22.4* (pyrr-4*C*); HRMS (m/z): [M+H]$^+$ calcd. for $C_{15}H_{21}BrN_2O_3$, 357.0814; found, 357.0816.

***tert*-Butyl 2-(((6-(phenylthio)pyridin-3-yl)oxy)methyl)pyrrolidine-1-carboxylate (12a).** Under inert atmosphere, tris(dibenzylideneacetone)dipalladium(0) (13 mg, 0.014 mmol) and DPEPhos (15 mg, 0.027 mmol) were dissolved in toluene (11 ml) and the resulting solution was stirred for 10 min at room temperature. Compound **L** (Scheme S5; 0.49 g, 1.36 mmol), thiophenol (0.15 ml, 1.36 mmol) and potassium *tert*-butoxide (0.17 g, 1.54 mmol) were subsequently added and the mixture was heated at 120 °C for 2 h. After cooling, the mixture was passed through a pad of Celite®. The filtrate was washed with aqueous NaOH (2 M), dried ($MgSO_4$), filtered, and concentrated under reduced pressure. The resulting crude product was purified by column chromatography (petrol: ethyl acetate = 9: 1 → 8: 2). The product was obtained as a yellow oil (0.48 g, 92%). ^{1}H NMR (400 MHz, DMSO-d_6, 80 °C): δ 8.22 (d, J = 3.1 Hz, 1H, pyr-2*H*), 7.47–7.34 (m, 6H, ph-2–6*H*, pyr-4*H*), 7.15 (d, J = 8.7 Hz, 1H, pyr-5*H*), 4.16–4.01 (m, 3H, pyrr-2*H*, OCH_2), 3.37–3.23 (m, 2H, pyrr-5H_2), 2.04–1.78 (m, 4H, pyrr-3,4H_2), 1.41 (s, 9H, C(CH_3)$_3$); ^{13}C NMR, *major rotational isomer* (150 MHz, DMSO-d_6): δ 153.8 (pyr-3*C*), 153.4 (*C*=O), *148.9* (pyr-6*C*), *137.9* (pyr-2*C*), *132.7* (ph-1*C*), 129.7 (ph-2,3,5,6*C*), 128.3 (pyr-5*C*), 124.3 (ph-4*C*), *123.3* (pyr-4*C*), *78.7* (*C*(CH$_3$)$_3$), *68.2* (O*C*H$_2$), *55.5* (pyrr-2*C*), *46.6* (pyrr-5*C*), *28.2* (C(*C*H$_3$)$_3$), *27.6* (pyrr-3*C*), *23.3* (pyrr-4*C*); ^{13}C NMR, *minor rotational isomer* (150 MHz, DMSO-d_6): δ 153.8 (pyr-3*C*), 153.4 (*C*=O), *149.0* (pyr-6*C*), *137.86* (pyr-2*C*), *132.8* (ph-1*C*), 129.7 (ph-2,3,5,6*C*), 128.3 (pyr-5*C*), 124.3 (ph-4*C*), *123.4*

(pyr-4*C*), *78.8* (*C*(CH_3)$_3$), *68.9* (O*C*H_2), *55.4* (pyrr-2*C*), *46.3* (pyrr-5*C*), *28.3* (pyrr-3*C*), *28.1* (C(*C*H_3)$_3$), *22.4* (pyrr-4*C*); HRMS (m/z): $[M]^+$ calcd. for $C_{21}H_{26}N_2O_3S$, 386.1664; found, 386.1663.

(5-((1-(*tert*-Butoxycarbonyl)pyrrolidin-2-yl)methoxy)pyridin-2-yl)diphenylsulfonium trifluoromethanesulfonate (12b). Following the general method for preparation of sulfonium salts (page S3), compound **12a** (Scheme S5; 0.1 g, 0.25 mmol), chlorobenzene (1 ml), diphenyliodonium trifluoromethanesulfonate (0.14 g, 0.33 mmol) and copper(II) benzoate dihydrate (4 mg, 0.013 mmol) were reacted at 125 °C for 2 h. Treatment with diethyl ether followed by column chromatography (DCM: methanol = 10: 0 → 9.5: 0.5) yielded the product as an orange solid (72 mg, 47%). Mp: 45–47 °C; ^{1}H NMR (400 MHz, DMSO-d_6, 80 °C): δ 8.59 (d, J = 2.9 Hz, 1H, pyr-6*H*), 8.16 (d, J = 8.9 Hz, 1H, pyr-3*H*), 7.88–7.74 (m, 11H, ph-2–6*H*, pyr-4*H*), 4.32–4.07 (m, 3H, pyrr-2*H*, OC*H*$_2$), 3.39–3.25 (m, 2H, pyrr-5*H*$_2$), 2.08–1.80 (m, 4H, pyrr-3,4*H*$_2$), 1.40 (s, 9H, C(C*H*$_3$)$_3$); ^{13}C NMR, *major rotational isomer* (150 MHz, DMSO-d_6): δ 158.2 (pyr-5*C*), *153.9* (*C*=O), 141.6 (pyr-6*C*), *135.8* (pyr-2*C*), 134.2 (ph-4*C*), 131.5 (ph-3,5*C*), 131.1 (ph-2,6*C*), *130.8* (pyr-4*C*), 125.8 (ph-1*C*), 123.7 (pyr-3*C*), 120.7 (q, $^1J_{C,F}$ = 323.0 Hz, *C*F_3), *78.8* (*C*(CH_3)$_3$), *68.9* (O*C*H_2), 55.2 (pyrr-2*C*), *46.6* (pyrr-5*C*), *28.1* (C(*C*H_3)$_3$), *27.6* (pyrr-3*C*), *23.3* (pyrr-4*C*); ^{13}C NMR, *minor rotational isomer* (150 MHz, DMSO-d_6): δ 158.2 (pyr-5*C*), *153.4* (*C*=O), 141.6 (pyr-6*C*), *135.85* (pyr-2*C*), 134.2 (ph-4*C*), 131.5 (ph-3,5*C*), 131.1 (ph-2,6*C*), *130.9* (pyr-4*C*), 125.8 (ph-1*C*), 123.7 (pyr-3*C*), 120.7 (q, $^1J_{C,F}$ = 323.0 Hz, *C*F_3), *78.9* (*C*(CH_3)$_3$), *69.7* (O*C*H_2), 55.2 (pyrr-2*C*), *46.3* (pyrr-5*C*), *28.3* (pyrr-3*C*), *28.1* (C(*C*H_3)$_3$), *22.4* (pyrr-4*C*); ^{19}F NMR (282 MHz, DMSO-d_6): δ –78.8 (C*F*$_3$); HRMS (m/z): $[M]^+$ calcd. for $C_{27}H_{31}N_2O_3S$, 463.2055; found, 463.2058.

g. Compound 13b

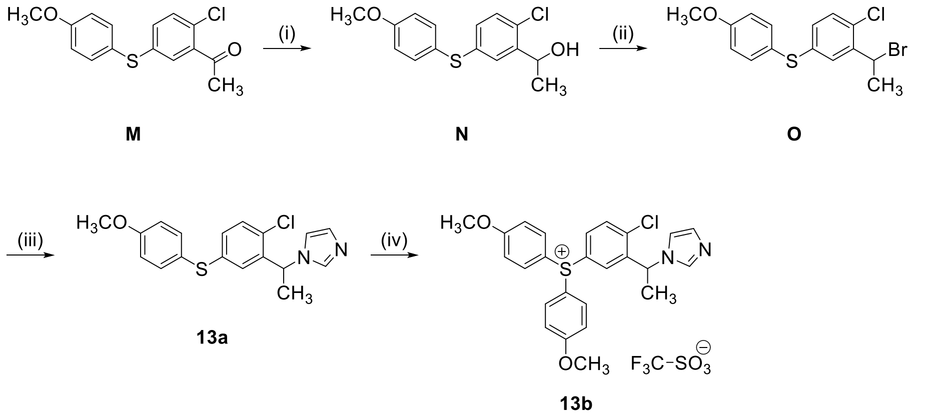

Scheme S6. Synthesis of the sulfonium salt **13b**

Reagents and conditions: (i) $NaBH_4$, methanol, 0 °C – rt, 2 h; (ii) PBr_3, DCM, 0 °C – rt, 6 h; (iii) NaH, imidazole, DMF, rt, 16 h; (iv) **J**, Cu(II) benzoate, TFSA, chlorobenzene, 125 °C, 12 h.

1-(2-Chloro-5-((4-methoxyphenyl)thio)phenyl)ethan-1-one (M). To a solution of 1-(5-bromo-2-chlorophenyl)ethan-1-one (0.2 ml, 2.0 mmol) and *N*,*N*-diisopropylethylamine (0.7 ml, 4.0 mmol) in anhydrous dioxane (8 ml) were added under inert atmosphere tris(dibenzylideneacetone)dipalladium(0) (46 mg, 0.05 mmol), Xantphos (58 mg, 0.1 mmol) and 4-methoxythiophenol (0.25 ml, 2.0 mmol), and the mixture was heated at 100 °C for 16 h. After cooling, the mixture was diluted with ethyl acetate and washed with aqueous NaOH (2 M). The organic phase was dried ($MgSO_4$), filtered and concentrated under reduced pressure. The crude product was purified by column chromatography (toluene: DCM = 9: 1). The product was isolated as a colourless oil (2.1 g, 82%). ^{1}H NMR (600 MHz, $CDCl_3$): δ 7.42 (d, J = 8.6 Hz, 2H, ph'-2,6*H*), 7.26 (d, J = 2.2 Hz, 1H, ph-6*H*), 7.24 (d, J = 8.5 Hz, 1H, ph-3*H*), 7.09 (dd,

J = 8.5/2.2 Hz, 1H, ph-4*H*), 6.92 (d, J = 8.6 Hz, 2H, ph'-3,5*H*), 3.84 (s, 3H, OC*H*3), 2.58 (s, 3H, COC*H*3); ^{13}C NMR (150 MHz, $CDCl_3$): δ 200.2 (*C*=O), 160.5 (ph'-4*C*), 139.5 (ph-1*C*), 139.1 (ph-5*C*), 136.2 (ph'-2,6*C*), 131.1 (ph-3*C*), 130.7 (ph-4*C*), 128.2 (ph-2*C*), 127.9 (ph-6*C*), 122.5 (ph'-1*C*), 115.5 (ph'-3,5*C*), 55.5 (O*C*H3), 30.8 (CO*C*H3); HRMS (m/z): $[M+H]^+$ calcd. for $C_{15}H_{13}ClO_2S$, 293.0403; found, 293.0401.

1-(2-Chloro-5-((4-methoxyphenyl)thio)phenyl)ethan-1-ol (N). A solution of sodium borohydride (0.47 g, 12.3 mmol) in methanol (15 ml) was cooled to 0 °C, and a solution of compound **M** (Scheme S6; 1.8 g, 6.15 mmol) in methanol (15 ml) was added dropwise. The reaction mixture was stirred at room temperature for 2 h and subsequently concentrated to dryness. The crude product was dissolved in DCM and the solution was extracted with aqueous HCl (2 M) and water. The organic phase was dried ($MgSO_4$), filtered and concentrated *in vacuo* to afford the product as a colourless oil (1.8 g, 87%). ^{1}H NMR (600 MHz, $CDCl_3$): δ 7.45 (d, J = 2.5 Hz, 1H, ph-6*H*), 7.41 (d, J = 8.8 Hz, 2H, ph'-2,6*H*), 7.16 (d, J = 8.4 Hz, 1H, ph-3*H*), 6.92–6.89 (m, 3H, ph-4*H*, ph'-3,5*H*), 5.21 (q, J = 6.7 Hz, 1H, C*H*CH3), 3.83 (s, 3H, OC*H*3), 1.85 (br s, 1H, O*H*), 1.44 (d, J = 6.5 Hz, 3H, CHC*H*3); ^{13}C NMR (150 MHz, $CDCl_3$): δ 160.2 (ph'-4*C*), 143.6 (ph-1*C*), 138.4 (ph-5*C*), 135.7 (ph'-2,6*C*), 130.0 (ph-3*C*), 129.1 (ph-2*C*), 127.9 (ph-4*C*), 126.0 (ph-6*C*), 123.7 (ph'-1*C*), 115.2 (ph'-3,5*C*), 67.1 (*C*HCH3), 55.5 (O*C*H3), 23.5 (CH*C*H3); HRMS (m/z): $[M+H]^+$ calcd. for $C_{15}H_{15}ClO_2S$, 295.0560; found, 295.0554.

(3-(1-Bromoethyl)-4-chlorophenyl)(4-methoxyphenyl)sulfane (O). Under inert atmosphere, a solution of phosphorous tribromide (1.92 ml, 20.4 mmol) in anhydrous DCM (5 ml) was cooled to 0 °C, and a solution of compound **N** (Scheme S6; 1.5 g, 5.09 mmol) in DCM (5 ml) was added dropwise. The reaction mixture was allowed to come to room temperature and was stirred at room temperature for 5 h. The reaction was quenched with a solution of potassium carbonate (5%) and extracted with DCM. The organic phase was washed with water, dried ($MgSO_4$), filtered and concentrated under reduced pressure to give the product as a yellow oil (1.15 g, 63%). ^{1}H NMR (600 MHz, $CDCl_3$): δ 7.44 (d, J = 2.3 Hz, 1H, ph'-2*H*), 7.42 (d, J = 8.5 Hz, 2H, ph-2,6*H*), 7.18 (d, J = 8.5 Hz, 1H, ph'-5*H*), 6.94–6.90 (m, 3H, ph'-6*H*, ph-3,5*H*), 5.53 (q, J = 6.9 Hz, 1H, C*H*CH3), 3.84 (s, 3H, OC*H*3), 1.95 (d, J = 6.9 Hz, 3H, CHC*H*3); ^{13}C NMR (150 MHz, $CDCl_3$): δ 160.3 (ph-4*C*), 140.7 (ph'-3*C*), 138.8 (ph'-1*C*), 135.9 (ph-2,6*C*), 130.2 (ph'-5*C*), 129.9 (ph'-4*C*), 128.6 (ph'-6*C*), 127.2 (ph'-2*C*), 123.2 (ph-1*C*), 115.3 (ph-3,5*C*), 55.5 (O*C*H3), 44.1 (*C*HCH3), 25.7 (CH*C*H3); HRMS (m/z): $[M+H]^+$ calcd. for $C_{15}H_{14}BrClOS$, 356.9716; found, 356.9713.

1-(1-(2-Chloro-5-((4-methoxyphenyl)thio)phenyl)ethyl)-1*H*-imidazole (13a). To a solution of sodium hydride (60% dispersion in mineral oil; 0.2 g, 5.04 mmol) in anhydrous DMF (7.5 ml) was added a solution of imidazole (0.26 g, 3.78 mmol) in DMF (7.5 ml). The resulting suspension was stirred at room temperature for 1 h. A solution of compound **O** (Scheme S6; 0.9 g, 2.52 mmol) in DMF (10 ml) was subsequently added dropwise and the reaction was stirred at room temperature for 15 h. The reaction mixture was quenched with water and the product was extracted with DCM. The organic phase was washed with water, dried ($MgSO_4$), filtered, concentrated and purified by column chromatography (ethyl acetate) to afford the product as a colourless oil (0.6 g, 70%). ^{1}H NMR (600 MHz, $CDCl_3$): δ 7.56 (s, 1H, im-2*H*), 7.35 (d, J = 8.9 Hz, 2H, ph'-2,6*H*), 7.21 (d, J = 8.4 Hz, 1H, ph-3*H*), 7.04 (s, 1H, im-5*H*), 6.93–6.89 (m, 3H, ph-4*H*, ph'-3,5*H*), 6.87 (s, 1H, im-4*H*), 6.73 (d, J = 2.3 Hz, 1H, ph-6*H*), 5.71 (q, J = 7.0 Hz, 1H, C*H*CH3), 3.85 (s, 3H, OC*H*3), 1.77 (d, J = 7.1 Hz, 3H, CHC*H*3); ^{13}C NMR (150 MHz, $CDCl_3$): δ 160.5 (ph'-4*C*), 139.7 (ph-1*C*), 139.5 (ph-5*C*), 136.2 (im-2*C*, ph'-2,6*C*), 130.4 (ph-3*C*), 129.34 (ph-2*C*), 129.31 (im-4*C*), 128.0 (ph-4*C*), 125.3 (ph-6*C*), 122.5 (ph'-1*C*), 118.0 (im-5*C*), 115.4 (ph'-3,5*C*), 55.6 (O*C*H3), 53.4 (*C*HCH3), 20.7 (CH*C*H3); HRMS (m/z): $[M+H]^+$ calcd. for $C_{18}H_{17}ClN_2OS$, 345.0828; found, 345.0824.

(3-(1-(1*H*-Imidazol-1-yl)ethyl)-4-chlorophenyl)bis(4-methoxyphenyl)sulfonium trifluoromethanesulfonate (13b). Following the general method for preparation of sulfonium salts (page S3), compound **13a** (Scheme S6; 180 mg, 0.53 mmol), chlorobenzene (1.5 ml), TFSA (47 µl, 0.53 mmol), compound **J** (Scheme S3; 260 mg, 0.53 mmol) and copper(II) benzoate dihydrate (8 mg, 0.027 mmol) were reacted at 125 °C for 12 h. Treatment with diethyl ether, column chromatography (DCM: methanol = 97.5: 2.5 → 95: 5) and extractive workup yielded the product as a colourless oil (165 mg, 52%). ^{1}H NMR (600 MHz, DMSO-*d*$_6$): δ 7.89 (d, J = 8.7 Hz, 1H, ph'-5*H*), 7.74–7.72 (m, 5H, im-2*H*, ph-2,6*H*), 7.62 (dd, J = 8.7/2.5 Hz, 1H, ph'-6*H*), 7.37 (d, J = 2.5 Hz, 1H, ph'-2*H*), 7.28 (dd, J = 9.0/2.2 Hz, 4H, ph-3,5*H*), 7.17 (s, 1H, im-5*H*), 6.85 (s, 1H, im-4*H*), 5.89 (q, J = 7.1 Hz, 1H, C*H*CH$_3$), 3.89 (s, 6H, OC*H*$_3$), 1.76 (d, J = 7.0 Hz, 3H, CHC*H*$_3$); ^{13}C NMR (150 MHz, DMSO-*d*$_6$): δ 163.8 (ph-4*C*), 142.2 (ph'-3*C*), 137.4 (ph'-4*C*), 136.7 (im-2*C*), 133.4 (ph-2,6*C*), 132.5 (ph'-5*C*), 131.0 (ph'-6*C*), 129.1 (ph'-2*C*), 128.8 (im-4*C*), 126.8 (ph'-1*C*), 120.7 (q, $^1J_{C,F}$ = 322.4, *C*F$_3$), 118.2 (im-5*C*), 116.9 (ph-3,5*C*), 114.9 (ph-1*C*), 56.2 (O*C*H$_3$), 52.3 (*C*HCH$_3$), 20.0 (CH*C*H$_3$); ^{19}F NMR (282 MHz, DMSO-*d*$_6$): δ –77.77 (C*F*$_3$); HRMS (m/z): [M]$^+$ calcd. for $C_{25}H_{24}ClN_2O_2S$, 451.1247; found, 451.1242.

h. Compound 14b

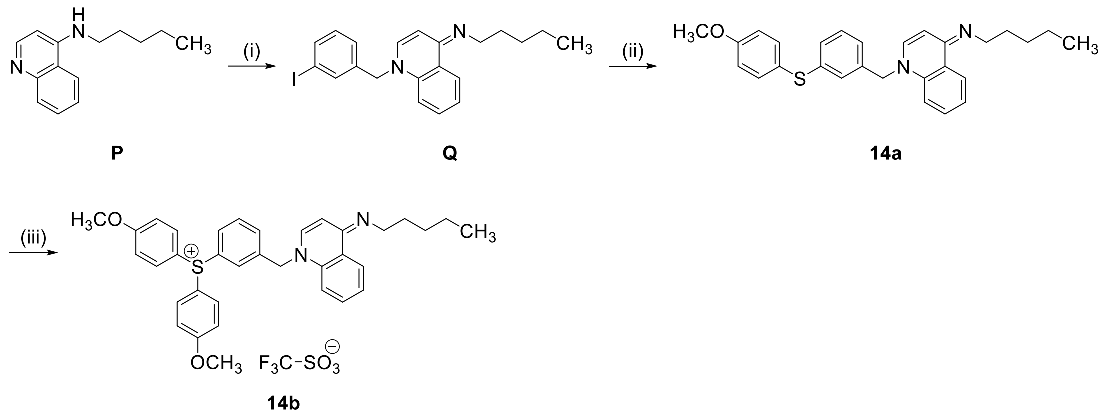

Scheme S7. Synthesis of the sulfonium salt **14b**

Reagents and conditions: (i) 1-(Bromomethyl)-3-iodobenzene, NaI, acetone, 60 °C, 18 h; (ii) 4-methoxythiophenol, Pd$_2$(dba)$_3$, DPEPhos, ^{t}ButOK, toluene, 100 °C, 3 h; (iii) **J**, Cu(II) benzoate, TFSA, chlorobenzene, 125 °C, 2.5 h.

***N*-Pentylquinolin-4-amine (P).** A solution of 4-chloroquinoline (1.0 g, 6.11 mmol) in amylamine (3.9 ml, 33.62 mmol) was heated at 110 °C for 24 h under inert atmosphere. After cooling, the solution was transferred to an ice/water mixture (~50 ml) while stirring vigorously. The precipitate formed was filtered off, washed with water and pentane, and dried to afford the product as an off white solid (1.15 g, 88%). Mp: 102–103 °C; ^{1}H NMR (500 MHz, CDCl$_3$): δ 8.56 (d, J = 5.4 Hz, 1H, quin-2*H*), 7.98 (d, J = 8.5 Hz, 1H, quin-5*H*), 7.73 (d, J = 8.2 Hz, 1H, quin-8*H*), 7.62 (ddd, J = 8.3/7.0/1.2 Hz, 1H, quin-7*H*), 7.41 (ddd, J = 8.2/7.0/1.2 Hz, 1H, quin-6*H*), 6.43 (d, J = 5.4 Hz, 1H, quin-3*H*), 5.00 (br s, 1H, N*H*), 3.33–3.30 (m, 2H, pent-1*H*$_2$), 1.77 (q, J = 7.3 Hz, 2H, pent-2*H*$_2$), 1.49–1.38 (m, 4H, pent-3,4*H*$_2$), 0.95 (t, J = 7.1 Hz, 3H, pent-5C*H*$_3$); ^{13}C NMR (125 MHz, CDCl$_3$): δ 151.2 (quin-2*C*), 149.8 (quin-4*C*), 148.5 (quin-8a*C*), 130.1 (quin-5*C*), 129.1 (quin-7*C*), 124.7 (quin-6*C*), 119.3 (quin-8*C*), 118.8 (quin-4a*C*), 98.9 (quin-3*C*), 43.4 (pent-1*C*), 29.4 (pent-3*C*), 28.8 (pent-2*C*), 22.6 (pent-4*C*), 14.2 (pent-5*C*); HRMS (m/z): [M]$^+$ calcd. for $C_{14}H_{18}N_2$, 214.1465; found, 214.1471.

1-(3-Iodobenzyl)-*N*-pentylquinolin-4(1*H*)-imine (Q). To a solution of compound **P** (Scheme S7; 0.50 g, 2.33 mmol) in acetone (10 ml) were added 1-(bromomethyl)-3-iodobenzene (0.76 g, 2.66 mmol) and sodium iodide (0.39 g, 2.57 mmol), and the mixture was heated at 60 °C for

18 h. After cooling, the mixture was poured on ice and stirred vigorously for 15 min. The precipitate formed was filtered off and washed with water and petrol. The precipitate was re-dissolved in ethanol (5 ml) and potassium *tert*-butoxide (0.39 g, 3.47 mmol) was added. The solution was stirred at room temperature for 15 min and subsequently extracted with DCM. The separated organic phase was dried ($MgSO_4$), filtered and concentrated to dryness to afford the product as a pale yellow solid (0.79 g, 79%). Mp: 118–119 °C; ^{1}H NMR (500 MHz, $CDCl_3$): δ 8.55 (d, J = 7.5 Hz, 1H, quin-5*H*), 7.61 (d, J = 7.9 Hz, 1H, ph-4*H*), 7.54 (s, 1H, ph-2*H*), 7.34 (t, J = 7.7 Hz, 1H, quin-7*H*), 7.20 (t, J = 7.5 Hz, 1H, quin-6*H*), 7.11–7.09 (m, 2H, quin-2*H*, ph-6*H*), 7.04 (t, J = 7.7 Hz, 1H, ph-5*H*), 6.96 (d, J = 8.3 Hz, 1H, quin-8*H*), 6.08 (d, J = 7.9 Hz, 1H, quin-3*H*), 5.05 (s, 2H, CH_2), 3.37 (t, J = 7.5 Hz, 2H, pent-1H_2), 1.76 (q, J = 7.4 Hz, 2H, pent-2H_2), 1.47–1.36 (m, 4H, pent-3,4H_2), 0.93 (t, J = 7.2 Hz, 3H, pent-5CH_3); ^{13}C NMR (125 MHz, $CDCl_3$): δ 154.6 (quin-4*C*), 139.5 (quin-2*C*), 138.6/138.4 (quin-8a*C*, ph-1*C*), 137.2 (ph-4*C*), 135.1 (ph-2*C*), 130.9 (ph-5*C*), 130.6 (quin-7*C*), 125.8 (quin-5*C*), 125.4 (quin-4a*C*), 125.4 (ph-6*C*), 123.8 (quin-6*C*), 115.1 (quin-8*C*), 99.2 (quin-3*C*), 95.1 (ph-3*C*), 55.0 (*C*H_2), 49.8 (pent-1*C*), 30.8 (pent-2*C*), 30.2 (pent-3*C*), 22.9 (pent-4*C*), 14.4 (pent-5*C*); HRMS (m/z): $[M]^+$ calcd. for $C_{21}H_{23}IN_2$, 430.0906; found, 430.0900.

1-(3-((4-Methoxyphenyl)thio)benzyl)-*N*-pentylquinolin-4(1*H*)-imine (14a). Under inert atmosphere, tris(dibenzylideneacetone)dipalladium(0) (19 mg, 0.02 mmol) and DPEPhos (23 mg, 0.04 mmol) were dissolved in toluene (35 ml) and the resulting solution was stirred under for 10 min at room temperature. Compound **Q** (Scheme S7; 0.89 g, 2.07 mmol), 4-methoxythiophenol (0.25 ml, 2.07 mmol) and potassium *tert*-butoxide (0.28 g, 2.49 mmol) were subsequently added and the mixture was heated at 100 °C for 3 h. After cooling, the mixture was passed through a pad of Celite®, the filtrate was concentrated under reduced pressure and purified by column chromatography (ethyl acetate: methanol = 100: 0 → 85: 15; solid phase: basic aluminium oxide) to afford the product as a viscous yellow oil (0.57 g, 63%). ^{1}H NMR (600 MHz, $CDCl_3$): δ 9.22 (dd, J = 8.3/1.2 Hz, 1H, quin-5*H*), 7.99 (d, J = 7.6 Hz, 1H, quin-2*H*), 7.61–7.55 (m, 2H, quin-6,7*H*), 7.33–7.30 (m, 3H, quin-8*H*, ph'-2,6*H*), 7.18 (t, J = 7.8 Hz, 1H, ph-5*H*), 7.02 (d, J = 8.3 Hz, 1H, ph-4*H*), 6.84 (d, J = 8.9 Hz, 2H, ph'-3,5*H*), 6.82 (d, J = 7.8 Hz, 1H, ph-6*H*), 6.77 (s, 1H, ph-2*H*), 6.32 (d, J = 7.7 Hz, 1H, quin-3*H*), 5.40 (s, 2H, CH_2), 3.83 (s, 3H, OCH_3), 3.51 (t, J = 7.4 Hz, 2H, pent-1H_2), 1.76 (q, J = 7.4 Hz, 2H, pent-2H_2), 1.41–1.34 (m, 4H, pent-3,4H_2), 0.91 (t, J = 7.1 Hz, 3H, pent-5CH_3); ^{13}C NMR (125 MHz, $CDCl_3$): δ 160.5 (ph'-4*C*), 156.3 (quin-4*C*), 141.5 (quin-8a*C*), 137.7 (ph-1*C*), 136.3 (ph'-2,6*C*), 134.9 (ph-3*C*), 133.6 (quin-7*C*), 130.0 (ph-5*C*), 127.8 (quin-5*C*), 127.3 (ph-4*C*), 126.8 (quin-6*C*), 124.5 (ph-2*C*), 123.1 (ph-6*C*), 122.2 (ph'-1*C*), 119.5 (quin-4a*C*), 116.6 (quin-8*C*), 115.3 (ph'-3,5*C*), 97.8 (quin-3*C*), 57.2 (*C*H_2), 55.6 (O*C*H_3), 44.5 (pent-1*C*), 29.4 (pent-3*C*), 27.9 (pent-2*C*), 22.6 (pent-4*C*), 14.2 (pent-5*C*); HRMS (m/z): $[M]^+$ calcd. for $C_{28}H_{30}N_2OS$, 442.2079; found, 442.2081.

Bis(4-methoxyphenyl)(3-((4-(pentylimino)quinolin-1(4*H*)-yl)methyl)phenyl)sulfonium trifluoromethanesulfonate (14b). Following the general method for preparation of sulfonium salts (page S3), compound **14a** (Scheme S7; 220 mg, 0.5 mmol), chlorobenzene (2.5 ml), TFSA (44 µl, 0.5 mmol), compound **j** (200 mg, 0.4 mmol) and copper(II) benzoate dihydrate (13 mg, 0.04 mmol) were reacted at 125 °C for 2.5 h. Treatment with diethyl ether, column chromatography (chloroform: methanol = 10: 0 → 9: 1) and extractive workup yielded the product as a colourless oil (90 mg, 32%). ^{1}H NMR (600 MHz, $CDCl_3$): δ 8.47 (dd, J = 8.1/1.4 Hz, 1H, quin-5*H*), 7.57 (t, J = 7.9 Hz, 1H, ph-5*H*), 7.53 (d, J = 9.2 Hz, 4H, ph'-2,6*H*), 7.51 (d, J = 8.0 Hz, 1H, ph-6*H*), 7.45 (d, J = 8.0 Hz, 1H, ph-4*H*), 7.33–7.29 (m, 2H, quin-2,7*H*), 7.26 (s, 1H, ph-2*H*), 7.19–7.16 (m, 1H, quin-6*H*), 7.09 (d, J = 9.0 Hz, 4H, ph'-3,5*H*), 6.91 (d, J = 8.3 Hz, 1H, quin-8*H*), 6.02 (d, J = 8.1 Hz, 1H, quin-3*H*), 5.29 (s, 2H, CH_2), 3.89 (s, 6H, OCH_3), 3.33 (t, J = 7.4 Hz, 2H, pent-1H_2), 1.74 (q, J = 7.4 Hz, 2H, pent-2H_2), 1.46–1.36 (m, 4H, pent-

3,4*H_2*), 0.93 (t, J = 7.1 Hz, 3H, pent-C*H_3*); ^{13}C NMR (125 MHz, $CDCl_3$): δ 164.7 (ph'-4*C*), 154.3 (quin-4*C*), 141.1 (quin-8a*C*), 140.2 (quin-2*C*), 138.0 (ph-3*C*), 133.2 (ph'-2,6*C*), 131.84/131.82 (ph-5,6*C*), 130.6 (quin-7*C*), 129.0 (ph-4*C*), 127.9 (ph-2*C*), 127.1 (ph-1*C*), 125.7 (quin-5*C*), 125.2 (quin-4a*C*), 123.8 (quin-6*C*), 121.0 (q, $^1J_{C,F}$ = 320.0 Hz, *C*F_3), 117.4 (ph'-3,5*C*), 115.2 (quin-8*C*), 113.6 (ph'-1*C*), 99.4 (quin-3*C*), 56.3 (O*C*H_3), 54.5 (*C*H_2), 49.6 (pent-1*C*), 30.7 (pent-2*C*), 30.2 (pent-3*C*), 22.9 (pent-4*C*), 14.3 (pent-5*C*); ^{19}F NMR (282 MHz, $CDCl_3$): δ –78.2 (C*F_3*); HRMS (m/z): $[M]^+$ calcd. for $C_{35}H_{37}N_2O_2S$, 549.2576; found, 549.2571.

3. Synthesis and Analytical Characterization of Fluorinated Reference Compounds

a. Compounds 1c, 3c, 4c, 8c, and 10d

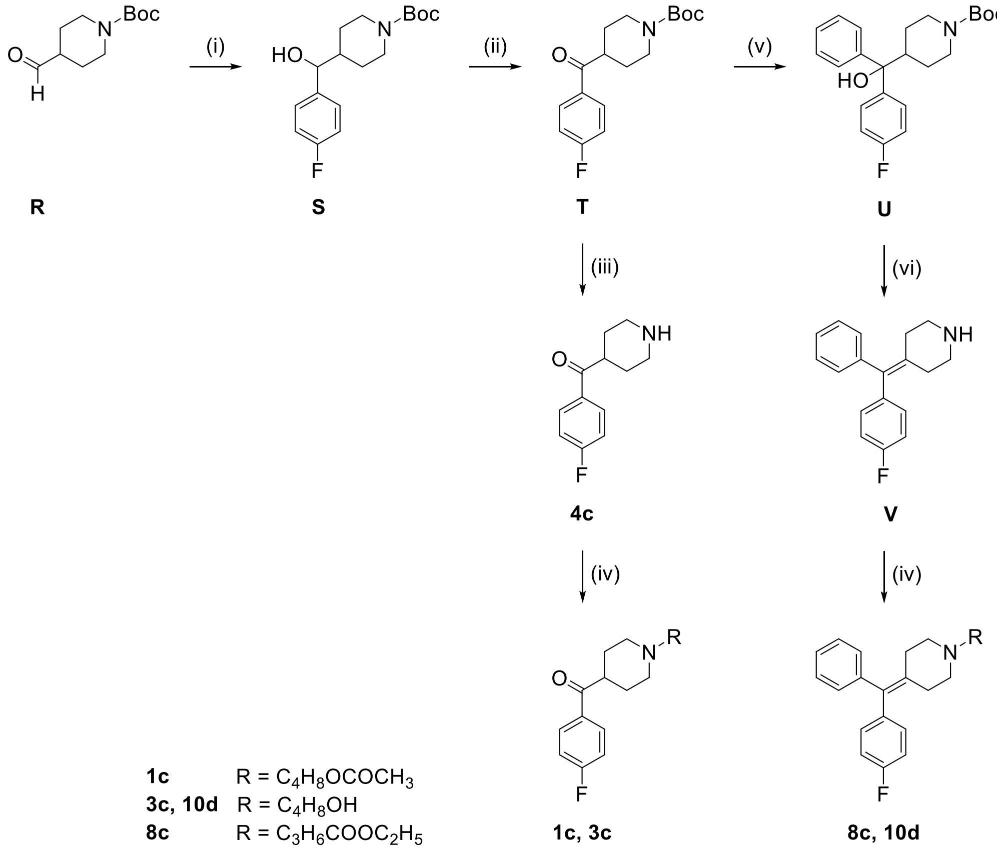

Scheme S8. Synthesis of the fluorinated compounds **1c**, **3c**, **4c**, **8c**, and **10d**

Reagents and conditions: (i) 4F-PhMgBr, THF, 0 – 60 °C, 2 h; (ii) Dess-Martin periodinane, DCM, rt, 2 h; (iii) HCl, dioxane, THF, rt, 12 h; (iv) alkyl bromide, K_2CO_3, acetone, 60 °C – rt, 15 h; (v) PhMgBr, THF, 0 – 60 °C, 2 h; (vi) TFA, DCM, 0 °C – rt, 2 h.

***tert*-Butyl 4-formylpiperidine-1-carboxylate (R).** Under inert atmosphere, *tert*-butyl 4-(hydroxymethyl)piperidine-1-carboxylate (1.0 g, 4.65 mmol), pyridinium chlorochromate (1.5 g, 7.0 mmol) and Celite® (1.5 g) were suspended in DCM (10 ml) and stirred at room temperature for 3 h. During this time the mixture turned from orange to brown. It was diluted with diethyl ether and filtered through a plug of Celite®. The filtrate was concentrated to dryness and purified by column chromatography (petrol: ethyl acetate = 10: 1 → 5: 1) to afford the product as a colourless oil (0.59 g, 59%). ^{1}H NMR (600 MHz, $CDCl_3$): δ 9.63 (s, 1H, C*H*O), 3.95 (br s, 2H, pip-2,6H^{eq}), 2.90 (t, J = 11.5 Hz, 2H, pip-2,6H^{ax}), 2.41–2.37 (m, 1H, pip-4*H*), 1.85 (d, J = 11.1 Hz, 2H, pip-3,5H^{eq}), 1.55–1.49 (m, 2H, pip-3,5H^{ax}), 1.43 (s, 9H, C(CH_3)$_3$); ^{13}C NMR (150 MHz, $CDCl_3$): δ 203.1 (*C*HO), 154.8 (*C*=O), 79.9 (*C*(CH_3)$_3$), 48.1 (pip-2,6*C*), 42.9 (pip-4*C*), 28.5 (C(*C*H_3)$_3$), 25.3 (pip-3,5*C*); HRMS (m/z): $[M+H]^+$ calcd. for $C_{11}H_{19}NO_3$, 214.1443; found, 214.1438.

***tert*-Butyl 4-((4-fluorophenyl)(hydroxy)methyl)piperidine-1-carboxylate (S).** A solution of 4-fluorophenylmagnesium bromide in THF (1 M, 9.2 ml) was cooled to 0 °C, and compound **R** (Scheme S8; 1.0 g, 4.6 mmol) dissolved in THF (5 ml) was added dropwise. The mixture was allowed to come to room temperature and subsequently heated to 60 °C for 2 h. Under cooling (ice bath), the reaction was quenched with brine. The mixture was concentrated and extracted with ethyl acetate. Purification by column chromatography (petrol: ethyl acetate = 8:

1 → 2: 1) gave the product as a colourless oil (0.75 g, 54%). ^{1}H NMR (600 MHz, DMSO-*d*$_6$): δ 7.30 (d, J = 8.5 Hz, 2H, ph-2,6*H*), 7.12 (d, J = 8.9 Hz, 2H, ph-3,5*H*), 5.27 (d, J = 4.4 Hz, 1H, CHO*H*), 4.28 (d, J = 6.5 Hz, 1H, C*H*OH), 3.98–3.85 (m, 2H, pip-2,6H^{eq}), 2.65–2.50 (m, 2H, pip-2,6H^{ax}), 1.72/1.19 (d/d, J = 13.0/11.0 Hz, 1H/1H, pip-3,5H^{eq}), 1.61–1.55 (m, 1H, pip-4*H*), 1.36 (s, 9H, C(CH_3)$_3$), 1.08–1.00 (m, 2H, pip-3,5H^{ax}); ^{13}C NMR (150 MHz, DMSO-*d*$_6$): δ 161.1 (d, $^{1}J_{C,F}$ = 240.5 Hz, ph-4*C*), 153.8 (*C*=O), 140.7 (d, $^{4}J_{C,F}$ = 2.7 Hz, ph-1*C*), 128.3 (d, $^{3}J_{C,F}$ = 7.8 Hz, ph-2,6*C*), 114.5 (d, $^{2}J_{C,F}$ = 20.9 Hz, ph-3,5*C*), 78.4 (*C*(CH$_3$)$_3$), 75.4 (*C*HOH), 43.9/42.9 (pip-2,6*C*), 43.2 (pip-4*C*), 28.2/27.6 (pip-3,5*C*), 28.1 (C(*C*H$_3$)$_3$); ^{19}F NMR (282 MHz, DMSO-*d*$_6$): δ –116.74 (ph-*F*); HRMS (m/z): [M+H]$^+$ calcd. for $C_{17}H_{24}FNO_3$, 310.1819; found, 310.1820.

***tert*-Butyl 4-(4-fluorobenzoyl)piperidine-1-carboxylate (T).** To a solution of compound **S** (Scheme S8; 0.75 g, 2.4 mmol) in DCM (10 ml) was added 1,1,1-triacetoxy-1,1-dihydro-1,2-benziodoxol-3(1*H*)-one (Dess-Martin periodinane; 1 g, 2.4 mmol), the suspension was stirred at room temperature for 2 h and subsequently concentrated to dryness. The crude mixture was dissolved in ethyl acetate and washed with a saturated solution of sodium bicarbonate and brine. The organic extract was purified by column chromatography (petrol: ethyl acetate = 9: 1 → 7: 1) to afford the product as a white solid (0.64 g, 87%). Mp: 89 °C; ^{1}H NMR (600 MHz, DMSO-*d*$_6$): δ 8.08 (dd, J = 8.8/5.6 Hz, 2H, ph-2,6*H*), 7.36 (t, J = 8.8 Hz, 2H, ph-3,5*H*), 3.97 (br s, 2H, pip-2,6H^{eq}), 3.65–3.60 (m, 1H, pip-4*H*), 2.90 (br s, 2H, pip-2,6H^{ax}), 1.75 (d, J = 12.2 Hz, 2H, pip-3,5H^{eq}), 1.40 (m, 11H, C(CH_3)$_3$, pip-3,5H^{ax}); ^{13}C NMR (150 MHz, DMSO-*d*$_6$): δ 200.7 (*C*=O^{ketone}), 165.1 (d, $^{1}J_{C,F}$ = 250.4 Hz, ph-4*C*), 153.9 (*C*=O^{boc}), 132.2 (d, $^{4}J_{C,F}$ = 2.9 Hz, ph-1*C*), 131.3 (d, $^{3}J_{C,F}$ = 9.5 Hz, ph-2,6*C*), 115.9 (d, $^{2}J_{C,F}$ = 21.6 Hz, ph-3,5*C*), 78.7 (*C*(CH$_3$)$_3$), 43.3/42.3 (pip-2,6*C*), 42.3 (pip-4*C*), 28.2 (pip-3,5*C*), 28.1 (C(*C*H$_3$)$_3$); ^{19}F NMR (282 MHz, DMSO-*d*$_6$): δ –106.45 (ph-*F*); HRMS (m/z): [M+H]$^+$ calcd. for $C_{17}H_{22}FNO_3$, 308.1662; found, 308.1665.

(4-Fluorophenyl)(piperidin-4-yl)methanone (4c). To a solution of compound **T** (Scheme S8; 0.63 g, 2 mmol) in THF (2.0 ml) was added HCl in dioxane (4 M, 1 ml). The solution was stirred at room temperature for 12 h. An extractive workup using aqueous NaOH (2 M) and DCM gave the product as a white solid (0.25 g, 61%). An analytical sample was crystallized as HCl salt. Mp: 200–202 °C (carbonization); ^{1}H NMR (600 MHz, DMSO-*d*$_6$): δ 9.31/9.01 (s/s, 1H/1H, NH_2^+), 8.11 (dd, J = 8.8/5.5 Hz, 2H, ph-2,6*H*), 7.39 (t, J = 8.8 Hz, 2H, ph-3,5*H*), 3.79–3.75 (m, 1H, pip-4*H*), 3.28 (d, J = 12.4 Hz, 2H, pip-2,6H^{eq}), 3.01 (q, J = 10.0 Hz, 2H, pip-2,6H^{ax}), 1.92 (d, J = 12.5 Hz, 2H, pip-3,5H^{eq}), 1.81–1.74 (m, 2H, pip-3,5H^{ax}); ^{13}C NMR (150 MHz, DMSO-*d*$_6$): δ 199.9 (*C*=O), 165.2 (d, $^{1}J_{C,F}$ = 250.7 Hz, ph-4*C*), 131.8 (d, $^{4}J_{C,F}$ = 2.7 Hz, ph-1*C*), 131.5 (d, $^{3}J_{C,F}$ = 9.5 Hz, ph-2,6*C*), 116.0 (d, $^{2}J_{C,F}$ = 21.8 Hz, ph-3,5*C*), 42.3 (pip-2,6*C*), 40.0 (pip-4*C*), 25.0 (pip-3,5*C*); ^{19}F NMR (282 MHz, DMSO-*d*$_6$): δ –106.01 (ph-*F*); HRMS (m/z): [M+H]$^+$ calcd. for $C_{12}H_{14}FNO$, 208.1138; found, 208.1141.

4-(4-(4-Fluorobenzoyl)piperidin-1-yl)butyl acetate (1c). Under inert atmosphere, compound **4c** (Scheme S8; 0.25 g, 1.2 mmol), 4-bromobutyl acetate (0.36 ml, 2.4 mmol) and potassium carbonate (0.5 g, 3.6 mmol) were dissolved in anhydrous acetone (10 ml). After heating at 60 °C for 3 h the mixture was stirred at room temperature for further 12 h. The inorganic compounds were filtered off and the filtrate purified by column chromatography (DCM: methanol = 100: 0 → 95: 5). The product was isolated as a colourless oil (0.32 g, 82%). ^{1}H NMR (600 MHz, DMSO-*d*$_6$): δ 8.05 (dd, J = 8.8/5.6 Hz, 2H, ph-2,6*H*), 7.35 (t, J = 8.8 Hz, 2H, ph-3,5*H*), 4.00 (t, J = 6.6 Hz, 2H, but-1H_2), 3.38 (t, J = 3.7 Hz, 1H, pip-4*H*), 2.87 (d, J = 10.9 Hz, 2H, pip-2,6H^{eq}), 2.29 (t, J = 6.9 Hz, 2H, but-4H_2), 2.02 (t, J = 10.9 Hz, 2H, pip-2,6H^{ax}), 1.99 (s, 3H, COCH_3), 1.73 (d, J = 12.8 Hz, 2H, pip-3,5H^{eq}), 1.59–1.51 (m, 4H, but-2H_2, pip-3,5H^{ax}), 1.46 (q, J = 7.3 Hz, 2H, but-3H_2); ^{13}C NMR (150 MHz, DMSO-*d*$_6$): δ 201.2 (*C*=O^{ketone}),

170.5 (C=O^{acetyl}), 165.0 (d, $^1J_{C,F}$ = 250.1 Hz, ph-4*C*), 132.4 (d, $^4J_{C,F}$ = 2.7 Hz, ph-1*C*), 131.2 (d, $^3J_{C,F}$ = 9.3 Hz, ph-2,6*C*), 115.9 (d, $^2J_{C,F}$ = 21.6 Hz, ph-3,5*C*), 63.8 (but-1*C*), 57.6 (but-4*C*), 52.6 (pip-2,6*C*), 42.8 (pip-4*C*), 28.5 (but-2*C*), 26.2 (pip-3,5*C*), 22.8 (but-3*C*), 20.8 (COCH$_3$); ^{19}F NMR (282 MHz, DMSO-d_6): δ –106.73 (ph-*F*); HRMS (m/z): [M]$^+$ calcd. for $C_{18}H_{24}FNO_3$, 321.1735; found, 321.1739.

(4-Fluorophenyl)(1-(4-hydroxybutyl)piperidin-4-yl)methanone (3c). Under inert atmosphere, compound **4c** (Scheme S8; 0.25 g, 1.2 mmol), 4-bromobutan-1-ol (0.37 g, 2.4 mmol), and potassium carbonate (0.5 g, 3.6 mmol) were suspended in acetone (10 ml). After heating at 60 °C for 3 h the mixture was stirred at room temperature for 12 h. After cooling, the inorganic compounds were filtered off, and the filtrate was purified by column chromatography (1% triethylamine; DCM: methanol = 98: 2 → 9: 1) to afford the product as a yellow oil (0.13 g, 38%). ^{1}H NMR (600 MHz, DMSO-d_6): δ 8.05 (dd, J = 8.7/5.6 Hz, 2H, ph-2,6*H*), 7.35 (t, J = 8.8 Hz, 2H, ph-3,5*H*), 4.62 (br s, 1H, O*H*), 3.39–3.36 (m, 3H, but-4H_2, pip-4*H*), 2.89 (d, J = 11.8 Hz, 2H, pip-2,6H^{eq}), 2.28 (t, J = 7.0 Hz, 2H, but-1H_2), 2.03 (t, J = 11.3 Hz, 2H, pip-2,6H^{ax}), 1.74 (d, J = 13.5 Hz, 2H, pip-3,5H^{eq}), 1.58–1.51 (m, 2H, pip-3,5H^{ax}), 1.48–1.39 (m, 4H, but-2,3H_2); ^{13}C NMR (150 MHz, DMSO-d_6): δ 201.2 (C=O), 165.0 (d, $^1J_{C,F}$ = 251.5 Hz, ph-4*C*), 132.4 (d, $^4J_{C,F}$ = 2.6 Hz, ph-1*C*), 131.2 (d, $^3J_{C,F}$ = 9.5 Hz, ph-2,6*C*), 115.9 (d, $^2J_{C,F}$ = 21.6 Hz, ph-3,5*C*), 60.8 (but-4*C*), 58.1 (but-1*C*), 52.6 (pip-2,6*C*), 42.8 (pip-4*C*), 30.7 (but-3*C*), 28.5 (pip-3,5*C*), 23.3 (but-2*C*); ^{19}F NMR (282 MHz, DMSO-d_6): δ –106.67 (ph-*F*); HRMS (m/z): [M+H]$^+$ calcd. for $C_{16}H_{22}FNO_2$, 280.1713; found, 280.1716.

***tert*-Butyl 4-((4-fluorophenyl)(hydroxy)(phenyl)methyl)piperidine-1-carboxylate (U).** Compound **T** (Scheme S8; 0.78 g, 2.8 mmol) dissolved THF (5 ml) was added dropwise to a solution of phenylmagnesium bromide in THF (1 M, 5.6 ml) kept at 0 °C. The reaction was allowed to come to room temperature and was subsequently heated to 60 °C for 2 h. After cooling, it was quenched with brine and concentrated. The resulting aqueous solution was extracted with ethyl acetate. The organic phase was dried ($MgSO_4$), filtered, concentrated and crystallized from ethanol to give the product as a white solid (0.56 g, 54%). Mp: 155–156 °C; ^{1}H NMR (500 MHz, $CDCl_3$): δ 7.45–7.40 (m, 4H, ph-2,6*H*, ph'-2,6*H*), 7.31 (t, J = 7.8 Hz, 2H, ph-3,5*H*), 7.21 (t, J = 8.8 Hz, 1H, ph-4*H*), 6.98 (t, J = 8.7 Hz, 2H, ph'-3,5*H*), 4.14 (d, J = 12.8 Hz, 2H, pip-2,6H^{eq}), 2.70 (tt, J = 13.0/3.0 Hz, 2H, pip-2,6H^{ax}), 2.51 (tt, J = 11.9/3.2 Hz, 1H, pip-4*H*), 2.08 (br s, 1H, O*H*), 1.55–1.43 (m, 11H, pip-3,5H^{eq}, C(CH_3)$_3$), 1.35–1.24 (m, 2H, pip-3,5H^{ax}); ^{13}C NMR (125 MHz, $CDCl_3$): δ 161.6 (d, $^1J_{C,F}$ = 244.4 Hz, ph'-4*C*), 154.8 (C=O), 145.5 (ph-1*C*), 141.4 (d, $^4J_{C,F}$ = 3.0 Hz, ph'-1*C*), 128.5 (ph-3,5*C*), 127.6 (d, $^3J_{C,F}$ = 8.0 Hz, ph'-2,6*C*), 127.0 (ph-4*C*), 125.8 (ph-2,6*C*), 115.1 (d, $^2J_{C,F}$ = 51.3 Hz, ph'-3,5*C*), 79.5 (*C*OH), 68.0 (C(CH$_3$)$_3$), 44.5 (pip-2,6*C*), 44.1 (pip-4*C*), 28.5 (C(CH$_3$)$_3$), 26.5 (pip-3,5*C*); ^{19}F NMR (282 MHz, DMSO-d_6): δ –116.82 (ph-*F*); HRMS (m/z): [M+H]$^+$ calcd. for $C_{23}H_{28}FNO_3$, 386.2132; found, 386.2133.

4-((4-Fluorophenyl)(phenyl)methylene)piperidine (V). Compound **U** (Scheme S8; 1.18 g, 3.1 mmol) was dissolved in DCM (15 ml). TFA (2 ml) was added and the solution was stirred 2 h at room temperature. The mixture was made basic by addition of aqueous NaOH (2 M) and the organic phase was dried and concentrated under reduced pressure giving the product as colourless oil (0.68 g, 82%). ^{1}H NMR (600 MHz, DMSO-d_6): δ 7.30 (t, J = 7.6 Hz, 2H, ph-3,5*H*), 7.21 (t, J = 7.4 Hz, 1H, ph-4*H*), 7.14–7.06 (m, 6H, ph-2,6*H*, ph'-2,3,5,6*H*), 2.73 (q, J = 5.1 Hz, 4H, pip-2,6H_2), 2.15 (t, J = 5.1 Hz, 4H, pip-3,5H_2); ^{13}C NMR (150 MHz, DMSO-d_6): δ 160.7 (d, $^1J_{C,F}$ = 242.4 Hz, ph'-4*C*), 142.1 (ph-1*C*), 138.5 (d, $^4J_{C,F}$ = 3.2 Hz, ph'-1*C*), 137.0 (C=C^{pip}), 133.5 (C=C^{pip}), 131.3 (d, $^3J_{C,F}$ = 8.0 Hz, ph'-2,6*C*), 129.4 (ph-3,5*C*), 128.2 (ph-2,6*C*), 126.4 (ph-4*C*), 114.9 (d, $^2J_{C,F}$ = 21.2 Hz, ph'-3,5*C*), 48.0 (pip-2,6*C*), 33.1 (pip-3,5*C*); ^{19}F NMR

(282 MHz, DMSO-d_6): δ –116.33 (ph'-*F*); HRMS (m/z): $[M+H]^+$ calcd. for $C_{18}H_{18}FN$, 268.1496; found, 268.1489.

Ethyl 4-(4-((4-Fluorophenyl)(phenyl)methylene)piperidin-1-yl)butanoate (8c). Under inert atmosphere, compound **V** (Scheme S8; 0.33 g, 1.2 mmol), 4-bromo-butyric acid ethyl ester (0.35 ml, 2.4 mmol) and potassium carbonate (0.67 g, 4.9 mmol) were dissolved in anhydrous acetone (20 ml). After heating at 60 °C for 3 h the mixture was stirred at room temperature for further 12 h. The inorganic compounds were filtered off, and the filtrate purified by column chromatography (DCM: methanol = 100: 0 → 98: 2) to afford the product as a colourless oil (0.31 g, 68%). ^{1}H NMR (600 MHz, DMSO-d_6): δ 7.31 (t, J = 7.7 Hz, 2H, ph-3,5*H*), 7.22 (t, J = 7.4 Hz, 1H, ph-4*H*), 7.14–7.07 (m, 6H, ph-2,6*H*, ph'-2,3,5,6*H*), 4.03 (q, J = 7.1 Hz, 2H, ethyl-CH_2), 2.38 (br s, 4H, pip-2,6H_2), 2.30–2.26 (m, 4H, but-2,4H_2), 2.22 (t, J = 5.3 Hz, 4H, pip-3,5H_2), 1.67 (q, J = 7.2 Hz, 2H, but-3H_2), 1.17 (t, J = 7.1 Hz, 3H, ethyl-CH_3); ^{13}C NMR (150 MHz, DMSO-d_6): δ 172.9 (*C*=O), 160.8 (d, $^1J_{C,F}$ = 241.8 Hz, ph'-4*C*), 142.0 (ph-1*C*), 138.4 (d, $^4J_{C,F}$ = 2.7 Hz, ph'-1*C*), 136.0 (*C*=C^{pip}), 134.0 (C=*C*^{pip}), 131.3 (d, $^3J_{C,F}$ = 8.0 Hz, ph'-2,6*C*), 129.4 (ph-3,5*C*), 128.2 (ph-2,6*C*), 126.5 (ph-4*C*), 115.0 (d, $^2J_{C,F}$ = 21.2 Hz, ph'-3,5*C*), 59.7 (ethyl-*C*H$_2$), 56.8 (but-4*C*), 54.6 (pip-2,6*C*), 31.6 (but-2*C*), 31.3 (pip-3,5*C*), 22.0 (but-3*C*), 14.2 (ethyl-*C*H$_3$); ^{19}F NMR (282 MHz, DMSO-d_6): δ –116.56 (ph'-*F*); HRMS (m/z): $[M+H]^+$ calcd. for $C_{24}H_{28}FNO_2$, 382.2177; found, 382.2187.

4-(4-((4-Fluorophenyl)(phenyl)methylene)piperidin-1-yl)butan-1-ol (10d). Under inert atmosphere, compound **V** (Scheme S8; 162.5 mg, 0.6 mmol), 4-bromo-1-butanol (0.1 ml, 1.2 mmol) and potassium carbonate (0.34 g, 2.5 mmol) were dissolved in anhydrous acetone (10 ml). After heating at 60 °C for 3 h the mixture was stirred at room temperature for further 12 h. The inorganic compounds were filtered off and the filtrate purified by column chromatography (DCM: methanol = 100: 0 → 95: 5) to afford the product as a colourless oil (102 mg, 51%). ^{1}H NMR (600 MHz, DMSO-d_6): δ 7.31 (t, J = 7.6 Hz, 2H, ph-3,5*H*), 7.22 (t, J = 7.4 Hz, 1H, ph-4*H*), 7.15–7.08 (m, 6H, ph-2,6*H*, ph'-2,3,5,6*H*), 4.57 (br s, 1H, O*H*), 3.38 (t, J = 6.2 Hz, 2H, but-1H_2), 2.41 (br s, 4H, pip-2,6H_2), 2.28 (t, J = 6.5 Hz, 2H, but-4H_2), 2.24 (t, J = 5.3 Hz, 4H, pip-3,5H_2), 1.47–1.39 (m, 4H, but-2,3H_2); ^{13}C NMR (150 MHz, DMSO-d_6): δ 160.8 (d, $^1J_{C,F}$ = 241.8 Hz, ph'-4*C*), 142.0 (ph-1*C*), 138.4 (d, $^4J_{C,F}$ = 3.2 Hz, ph'-1*C*), 135.9 (*C*=C^{pip}), 134.0 (C=*C*^{pip}), 131.3 (d, $^3J_{C,F}$ = 7.8 Hz, ph'-2,6*C*), 129.4 (ph-3,5*C*), 128.2 (ph-2,6*C*), 126.5 (ph-4*C*), 115.0 (d, $^2J_{C,F}$ = 21.0 Hz, ph'-3,5*C*), 60.7 (but-1*C*), 57.6 (but-4*C*), 54.6 (pip-2,6*C*), 31.2 (pip-3,5*C*), 30.7 (but-2*C*), 23.3 (but-3*C*); ^{19}F NMR (282 MHz, DMSO-d_6): δ –116.53 (ph'-*F*); HRMS (m/z): $[M+H]^+$ calcd. for $C_{22}H_{26}FNO$, 340.2071; found, 340.2075.

b. Compounds 11c, 12d and 14c

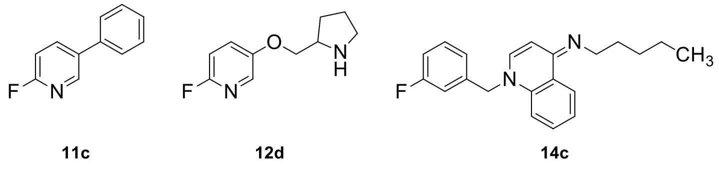

11c **12d** **14c**

Figure S1. Structures of the fluorinated compounds **11c**, **12d** and **14c**

2-Fluoro-5-phenylpyridine (11c). 2-Fluoro-5-bromopyridine (0.1 ml, 1 mmol) and phenylboronic acid (0.12 g, 1 mmol) were dissolved in a mixture of acetonitrile (5 ml) and sodium carbonate solution (0.4 M; 5 ml). Tetrakis(triphenylphosphine)palladium(0) (60 mg, 0.05 mmol) was added and the mixture was heated at 90 °C for 3 hours. After cooling, the organic solvent was removed under reduced pressure, the remaining aqueous solution diluted with water and the mixture extracted with DCM. The organic phase was dried ($MgSO_4$),

filtered, concentrated under reduced pressure and purified by column chromatography (petrol: ethyl acetate = 100: 0 → 85: 15) to afford the product as a colourless oil (0.16 g, 91%). ^{1}H NMR (600 MHz, $CDCl_3$,): δ 8.42 (d, J = 2.5 Hz, 1H, pyr-6*H*), 7.97 (td, J = 8.0/2.6 Hz, 1H, pyr-4*H*), 7.53 (d, J = 7.2 Hz, 2H, ph-2,6*H*), 7.48 (t, J = 7.7 Hz, 2H, ph-3,5*H*), 7.41 (t, J = 7.4 Hz, 1H, ph-4*H*), 7.01 (dd, J = 8.4/3.1 Hz, 1H, pyr-3*H*); ^{13}C NMR (150 MHz, $CDCl_3$): δ 163.2 (d, $^1J_{C,F}$ = 240.0 Hz, pyr-2*C*), 146.0 (d, $^3J_{C,F}$ = 14.8 Hz, pyr-6*C*), 139.9 (d, $^3J_{C,F}$ = 7.5 Hz, pyr-4*C*), 136.8 (ph-1*C*), 135.0 (d, $^4J_{C,F}$ = 4.3 Hz, pyr-5*C*), 129.3 (ph-3,5*C*), 128.3 (ph-4*C*), 127.2 (ph-2,6*C*), 109.6 (d, $^2J_{C,F}$ = 38.2 Hz, pyr-3*C*); ^{19}F NMR (282 Hz, $CDCl_3$): δ –70.60 (pyr-*F*); HRMS (m/z): $[M]^+$ calcd. for $C_{11}H_8FN$, 173.0635; found, 173.0637.

2-Fluoro-5-(pyrrolidin-2-ylmethoxy)pyridine (12d). A solution of triphenylphosphine (0.79 g, 3 mmol) in THF (6 ml) was cooled to –10 °C. Diisopropyl azodicarboxylate (0.59 ml, 3 mmol) was added dropwise, and the resulting suspension was stirred for 30 minutes at –10 °C. Boc-L-prolinol (0.4 g, 2 mmol) and 6-fluoro-pyridin-3-ol (0.34 g, 3 mmol) dissolved in THF (2 ml) were added. The reaction was stirred for 1 hour at –10 °C, for 6 hours at 0 °C, and for 20 hours at room temperature. The organic solvents were removed under reduced pressure and the residue taken up in a mixture of diethyl ether and pentane (1: 1; 10 ml) in order to precipitate triphenylphosphine oxide under cooling (–78 °C), which was subsequently removed by filtration. The filtrate was concentrated *in vacuo* and the crude product purified by column chromatography (petrol: ethyl acetate = 93: 7 → 8: 2) to afford a colourless oil (0.43 g, 73%). The oil (0.1 g, 0.34 mmol) was taken up in DCM (2 ml) and TFA (2 ml) was added. The solution was stirred for 2.5 hours at room temperature, subsequently diluted with DCM and washed with aqueous NaOH (3 M). The separated organic solution was dried ($MgSO_4$), filtered and concentrated under reduced pressure. The resulting yellow oil was purified by column chromatography (DCM: methanol (containing 1 M ammonia) = 92.5: 7.5 → 85: 15) to afford the product as a yellow oil (64 mg, quantitative conversion). ^{1}H NMR (600 MHz, DMSO-*d*$_6$): δ 7.88 (s, 1H, pyr-6*H*), 7.61–7.58 (m, 1H, pyr-4*H*), 7.10 (dd, J = 9.0/3.5 Hz, 1H, pyr-3*H*), 3.89–3.85 (m, 2H, C*H*$_2$), 3.39 (q, J = 6.7 Hz, 1H, pyrr-2C*H*), 2.84–2.78 (m, 2H, pyrr-5*H*$_2$), 1.87–1.81 (m, 1H, pyrr-3*H*), 1.73–1.59 (m, 2H, pyrr-4*H*$_2$), 1.46–1.41 (m, 1H, pyrr-3*H*); ^{13}C NMR (150 MHz, DMSO-*d*$_6$): δ 157.1 (d, $^1J_{C,F}$ = 227.6 Hz, pyr-2*C*), 153.4 (d, $^4J_{C,F}$ = 3.3 Hz, pyr-5*C*), 131.1 (d, $^3J_{C,F}$ = 15.3 Hz, pyr-6*C*), 128.2 (d, $^3J_{C,F}$ = 8.7 Hz, pyr-4*C*), 109.7 (d, $^2J_{C,F}$ = 40.5 Hz, pyr-3*C*), 72.6 (*C*H_2), 56.5 (pyrr-2*C*H), 46.0 (pyrr-5*C*H_2), 28.1 (pyrr-3*C*H_2), 25.0 (pyrr-4*C*H_2); ^{19}F NMR (282 MHz, DMSO-*d*$_6$): δ –78.7 (pyr-*F*); HRMS (m/z): $[M+H]^+$ calcd. for $C_{10}H_{13}FN_2O$, 197.1090; found, 197.1090.

***N*-(1-(3-Fluorobenzyl)quinolin-4(1*H*)-ylidene)pentan-1-amine (14c).** To a solution of compound **P** (Scheme S7; 0.86 g, 4.0 mmol) in acetone (15 ml), 3-fluorobenzyl bromide (0.54 ml, 4.4 mmol) and sodium iodide (0.78 g, 4.79 mmol) were added and the mixture was heated at 60 °C for 18 h. After cooling, DCM was added and the resulting mixture was washed with water. The organic phase was dried ($MgSO_4$), filtered and concentrated under reduced pressure. The resulting residue was triturated with petrol to afford an off-white precipitate, which was filtered off. The solid was dissolved in ethanol (20 ml), and potassium *tert*-butoxide (0.49 g, 4.4 mmol) was added. The resulting solution was stirred at room temperature for 15 min, subsequently diluted with DCM and washed with water. The organic phase was dried ($MgSO_4$), filtered and evaporated to dryness. Re-crystallization with pentane and diethyl ether afforded the product as a pale yellow solid (1.28 g, 71%). Mp: 64–65 °C; ^{1}H NMR (600 MHz, $CDCl_3$): δ 8.50 (d, J = 8.1 Hz, 1H, quin-5*H*), 7.32–7.27 (m, 2H, quin-7*H*, ph-5*H*), 7.17 (t, J = 7.6 Hz, 1H, quin-6*H*), 7.00 (d, J = 8.5 Hz, 1H, quin-3*H*), 6.98–6.95 (m, 2H, ph-4,6*H*), 6.93 (d, J = 8.3 Hz, 1H, quin-8*H*), 6.87 (d, J = 9.5 Hz, 1H, ph-2*H*), 6.05 (d, J = 8.0 Hz, 1H, quin-2*H*), 5.05 (s, 2H, *CH*$_2$), 3.36 (t, J = 7.6 Hz, 2H, pent-1*H*$_2$), 1.76 (q, J = 7.5 Hz, 2H, pent-2*H*$_2$), 1.47–1.37 (m, 4H, pent-3,4*H*$_2$), 0.94 (t, J = 7.1 Hz, 3H, pent-5C*H*$_3$); ^{13}C NMR (150 MHz, $CDCl_3$): δ 163.3 (d,

$^{1}J_{C,F}$ = 249.0 Hz, ph-3*C*), 154.4 (quin-4*C*), 139.1 (d, $^{3}J_{C,F}$ = 6.7 Hz, ph-1*C*), 139.0 (quin-3*C*), 138.5 (quin-8a*C*), 130.8 (d, $^{3}J_{C,F}$ = 8.8 Hz, ph-5*C*), 130.2 (quin-7*C*), 126.0 (quin-4a*C*), 125.7 (quin-5*C*), 123.5 (quin-6*C*), 121.7 (d, $^{4}J_{C,F}$ = 2.6 Hz, ph-6*C*), 115.0 (d, $^{2}J_{C,F}$ = 21.7 Hz, ph-4*C*), 114.9 (quin-8*C*), 113.2 (d, $^{2}J_{C,F}$ = 21.7 Hz, ph-2*C*), 99.2 (quin-2*C*), 55.1 (CH_2), 50.3 (pent-1*C*), 31.0 (pent-2*C*), 30.3 (pent-3*C*), 22.9 (pent-4*C*), 14.4 (pent-5*C*); ^{19}F NMR (282 MHz, $CDCl_3$): δ –112.13 (ph'-*F*); HRMS (m/z): $[M–H]^+$ calcd. for $C_{21}H_{23}FN_2$, 321.1767; found, 321.1764.

c. Compound 13c

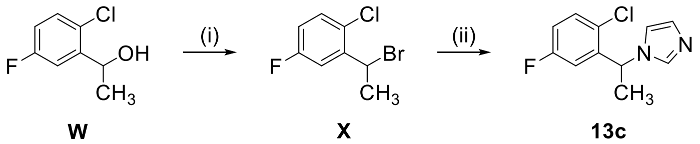

Scheme S9. Synthesis of the fluorinated compound **13c**

Reagents and conditions: (i) PBr_3, DCM, 0 °C – rt, 2 h; (ii) NaH, imidazole, DMF, rt, 16 h.

1-(2-Chloro-5-fluorophenyl)ethan-1-ol (W). A solution of 1-(2-chloro-5-fluorophenyl)ethan-1-one (2.0 g, 11.6 mmol) in methanol (25 ml) was added dropwise to a cooled (ice bath) solution of sodium borohydride (0.88 g, 23.2 mmol) in methanol (25 ml). The reaction mixture was stirred at room temperature for 2 hours. The volatiles were subsequently removed under reduced pressure and the resulting residue was suspended in water, acidified with HCl (2 M; to pH 6) and extracted with DCM. The organic phase was washed with water, dried ($MgSO_4$), filtered and concentrated *in vacuo* to afford the product as a colourless oil (1.70 g, 84%). ^{1}H NMR (500 MHz, $CDCl_3$): δ 7.34–7.26 (m, 2H, ph-3,4*H*), 6.92–6.88 (m, 1H, ph-6*H*), 5.23 (q, J = 6.2 Hz, 1H, C*H*), 2.03 (br s, 1H, O*H*), 1.47 (d, J = 6.4 Hz, 3H, CH_3); ^{13}C NMR (125 MHz, $CDCl_3$): δ 161.9 (d, $^{1}J_{C,F}$ = 246.7 Hz, ph-5*C*), 145.5 (d, $^{3}J_{C,F}$ = 6.7 Hz, ph-1*C*), 130.7 (d, $^{3}J_{C,F}$ = 7.8 Hz, ph-3*C*), 126.2 (d, $^{4}J_{C,F}$ = 2.9 Hz, ph-2*C*), 115.4 (d, $^{2}J_{C,F}$ = 23.0 Hz, ph-4*C*), 113.7 (d, $^{2}J_{C,F}$ = 23.9 Hz, ph-6*C*), 66.9 (*C*H), 23.5 (CH_3); ^{19}F NMR (282 MHz, $CDCl_3$): δ –114.29 (ph-*F*); HRMS (m/z): $[M]^+$ calcd. for C_8H_8ClFO, 174.0245; found, 174.0240.

2-(1-Bromoethyl)-1-chloro-4-fluorobenzene (X). A solution of compound **W** (Scheme S9; 1.0 g, 5.73 mmol) in anhydrous DCM (6 ml) was added dropwise to a solution of phosphorous tribromide (1.08 ml, 11.5 mmol) in DCM (6 ml) kept at 0 °C. The mixture was stirred at room temperature for 3 hours under inert atmosphere. Under cooling, the reaction was quenched with a solution of potassium carbonate (5%) and extracted with DCM. The organic phase was washed with water, dried ($MgSO_4$), filtered and concentrated under reduced pressure to afford the product as a yellow oil (1.36 g, 70%). ^{1}H NMR (600 MHz, $CDCl_3$): δ 7.36–7.31 (m, 2H, ph-3,6*H*), 6.95 (td, J = 8.1/3.0 Hz, 1H, ph-5*H*), 5.55 (qd, J = 7.0/1.5 Hz, 1H, C*H*), 2.01 (d, J = 7.0 Hz, 3H, CH_3); ^{13}C NMR (150 MHz, $CDCl_3$): δ 161.7 (d, $^{1}J_{C,F}$ = 247.2 Hz, ph-4*C*), 142.4 (d, $^{3}J_{C,F}$ = 7.5 Hz, ph-2*C*), 131.1 (d, $^{3}J_{C,F}$ = 8.1 Hz, ph-6*C*), 127.5 (d, $^{4}J_{C,F}$ = 2.9 Hz, ph-1*C*), 116.7 (d, $^{2}J_{C,F}$ = 23.0 Hz, ph-5*C*), 115.3 (d, $^{2}J_{C,F}$ = 24.1 Hz, ph-3*C*), 43.7 (*C*H), 25.8 (CH_3); ^{19}F NMR (282 MHz, $CDCl_3$): δ –113.60 (ph-*F*); HRMS (m/z): $[M+H]^+$ calcd. for C_8H_7BrClF, 236.9482; found, 236.9487.

1-(1-(2-Chloro-5-fluorophenyl)ethyl)-1*H*-imidazole (13c). A solution of imidazole (0.34 g, 4.94 mmol) in anhydrous DMF (5 ml) was added dropwise to a solution of sodium hydride (60% dispersion in mineral oil; 0.16 g, 6.6 mmol) in DMF (5 ml). The mixture was stirred for 1 hour at room temperature. Compound **X** (Scheme S9; 0.78 g, 3.3 mmol) dissolved in DMF (5 ml) was added dropwise and the mixture was stirred at room temperature for 15 h. The reaction was quenched with water and extracted with DCM. The organic solution was washed with water, dried ($MgSO_4$), filtered and concentrated under reduced pressure. The crude

product was purified by column chromatography (ethyl acetate) to afford the product as a colourless oil (0.27 g, 36%). ^{1}H NMR (600 MHz, DMSO-d_6): δ 7.64 (s, 1H, im-2*H*), 7.36 (dd, J = 8.8/5.1 Hz, 1H, ph-3*H*), 7.12 (s, 1H, im-5*H*), 6.98–6.94 (m, 2H, ph-4*H*, im-4*H*), 6.67 (dd, J = 9.2/3.0 Hz, 1H, ph-6*H*), 5.75 (q, J = 7.0 Hz, 1H, C*H*), 1.84 (d, J = 7.0 Hz, 3H, CH_3); ^{13}C NMR (150 MHz, DMSO-d_6): δ 161.9 (d, $^{1}J_{C,F}$ = 247.9 Hz, ph-5*C*), 141.5 (d, $^{3}J_{C,F}$ = 6.8 Hz, ph-1*C*), 136.3 (im-2*C*), 131.5 (d, $^{3}J_{C,F}$ = 8.1 Hz, ph-3*C*), 129.8 (im-5*C*), 127.2 (d, $^{4}J_{C,F}$ = 3.3 Hz, ph-2*C*), 118.1 (im-4*C*), 116.6 (d, $^{2}J_{C,F}$ = 22.9 Hz, ph-4*C*), 114.0 (d, $^{2}J_{C,F}$ = 24.7 Hz, ph-6*C*), 53.5 (*C*H), 20.6 (*C*H_3); ^{19}F NMR (282 MHz, DMSO-d_6): δ –112.91 (ph-*F*); HRMS (m/z): $[M+H]^+$ calcd. for $C_{11}H_{10}ClFN_2$, 225.0595; found, 225.0591.

4. Radiosynthesis of fluorine-18 labelled compounds

a. General procedure for labelling of sulfonium salts with fluorine-18

[^{18}F]Fluoride (50–200 MBq per reaction) in water was trapped on a Sep-Pak® QMA cartridge, released with a solution (0.5 ml) of Kryptofix 222 (30 mM) and potassium bicarbonate (30 mM) dissolved in acetonitrile: water (85: 15). After removing the solvent by heating at 90 °C under a stream of nitrogen, acetonitrile (0.5 ml) was added, and the distillation was continued at 90 °C. This procedure was repeated and the reaction vial was subsequently capped. The respective sulfonium trifluoromethanesulfonate precursor (5 mg) dissolved in DMSO (0.5 ml) was added and the mixture was stirred for 15 minutes at the specified temperature (*cf.* Figures 2 and 4). After cooling, the reaction was quenched with water (1.5 ml) and the mixture was purified by HPLC. The decay-corrected isolated RCY was calculated by relating the amount of isolated radioactive product to the initial amount of [^{18}F]fluoride in [^{18}O]H_2O. The analytical RCY was determined by integrating the area under the curve in the preparative radio-HPLC chromatogram. No corrections were made to account for losses during the preparative procedure.

b. Preparation of compounds [^{18}F]1c–[^{18}F]8c and [^{18}F]11c–[^{18}F]13c

[^{18}F]4-(4-(4-Fluorobenzoyl)piperidin-1-yl)butyl acetate ([^{18}F]1c). The reaction mixture was purified using a Chromolith® SemiPrep RP18-e column (100 × 10 mm) at room temperature and with a flow rate of 5 ml/min. The mobile phase consisted of water and methanol, each containing 0.5% TFA. Gradient elution starting with 10% methanol content that was increased to 55% in 12 min and further to 90% in 3 min allowed for isolation of the radioactive product. Quality control was performed on an analytical Chromolith® Performance RP-18 column (100 × 4.6 mm) at room temperature and at a flow rate of 3 ml/min. The mobile phase consisted of water and methanol, each containing 0.5% TFA. Gradient elution started with 10% methanol content that was increased to 55% in 12 min, then further to 90% in 3 min. The radiochemical purity of [^{18}F]**1c** was >98% and the identity of the radiochemical product was confirmed by co-elution with the non-radioactive analogue.

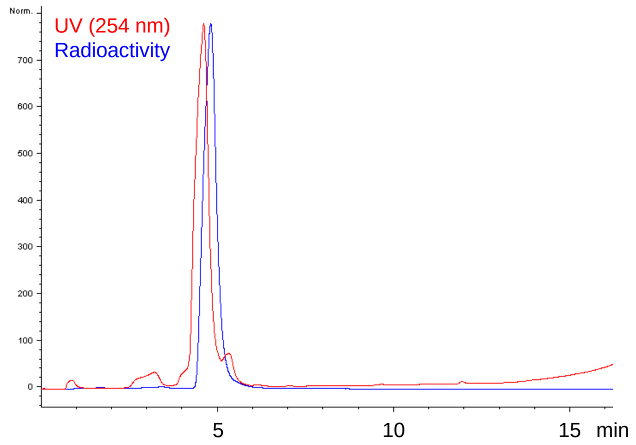

Figure S2. HPLC chromatogram of the isolated compound [^{18}F]**1c**, co-injected with the non-labelled reference **1c**.

[^{18}F]4-Fluorobenzophenone ([^{18}F]2c). The reaction mixture was purified using a Chromolith® SemiPrep RP18-e column (100 × 10 mm) at room temperature and with a flow rate of 5 ml/min. The mobile phase consisted of water and methanol, each containing 0.5% TFA. Elution starting with 10% methanol content kept constant for 5 min and then increased to 55% in 12 min and further to 90% in 3 min allowed for isolation of the radioactive product. Quality control was performed on an analytical Chromolith® Performance RP-18 column (100 × 4.6 mm) at room

temperature and at a flow rate of 3 ml/min. The mobile phase consisted of water and methanol, each containing 0.5% TFA. Gradient elution started with 10% methanol content, which was increased to 90% in 15 min. The radiochemical purity of [^{18}F]**2c** was >98% and the identity of the radiochemical product was confirmed by co-elution with the non-radioactive analogue.

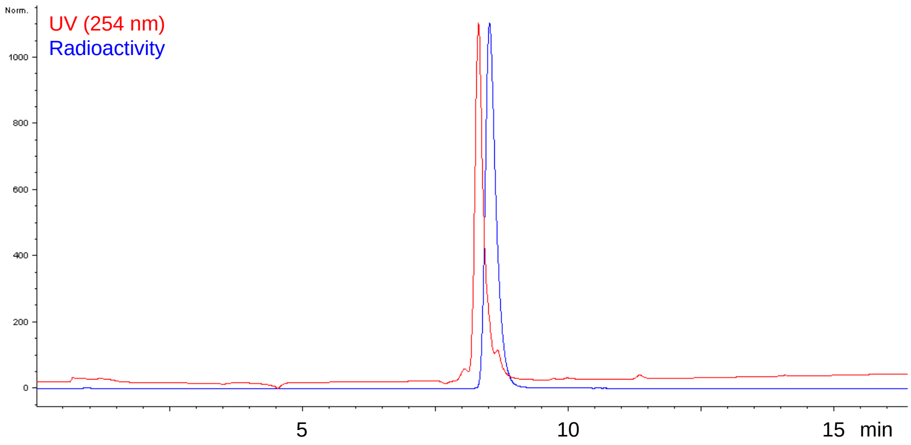

Figure S3. HPLC chromatogram of the isolated compound [^{18}F]**2c**, co-injected with 4-fluorobenzophenone.

[^{18}F](4-Fluorophenyl)(1-(4-hydroxybutyl)piperidin-4-yl)methanone ([^{18}F]3c). The reaction mixture was purified using a Chromolith® SemiPrep RP18-e column (100 × 10 mm) at room temperature and with a flow rate of 5 ml/min. The mobile phase consisted of water and methanol, each containing 0.5% TFA. Elution starting with 10% methanol content kept constant for 5 min and then increased to 55% in 12 min and further to 90% in 3 min allowed for isolation of the radioactive product. Quality control was performed on an analytical Chromolith® Performance RP-18 column (100 × 4.6 mm) at room temperature and at a flow rate of 3 ml/min. The mobile phase consisted of water and methanol, each containing 0.5% TFA. Gradient elution started with 1% methanol content, which was increased to 40% in 15 min. The radiochemical purity of [^{18}F]**3c** was >98% and the identity of the radiochemical product was confirmed by co-elution with the non-radioactive analogue.

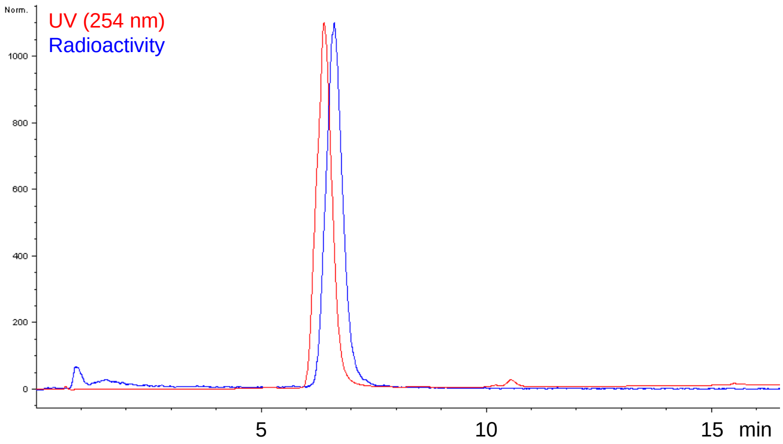

Figure S4. HPLC chromatogram of the isolated compound [^{18}F]**3c**, co-injected with the non-labelled reference **3c**.

[^{18}F](4-Fluorophenyl)(piperidin-4-yl)methanone ([^{18}F]4c). The reaction mixture was purified using a Chromolith® SemiPrep RP18-e column (100 × 10 mm) at room temperature and with a flow rate of 5 ml/min. The mobile phase consisted of water and methanol, each containing 0.5% TFA. Elution starting with 1% methanol content kept constant for 5 min, increased to 10% in 5 min, then further to 90% in 14 min, allowed for isolation of the

radioactive product. Quality control was performed on an analytical Chromolith® Performance RP-18 column (100 × 4.6 mm) at room temperature and at a flow rate of 3 ml/min. The mobile phase consisted of water and methanol, each containing 0.5% TFA. Gradient elution started with 1% methanol content, which was increased to 40% in 15 min. The radiochemical purity of [^{18}F]**4c** was >98% and the identity of the radiochemical product was confirmed by co-elution with the non-radioactive analogue.

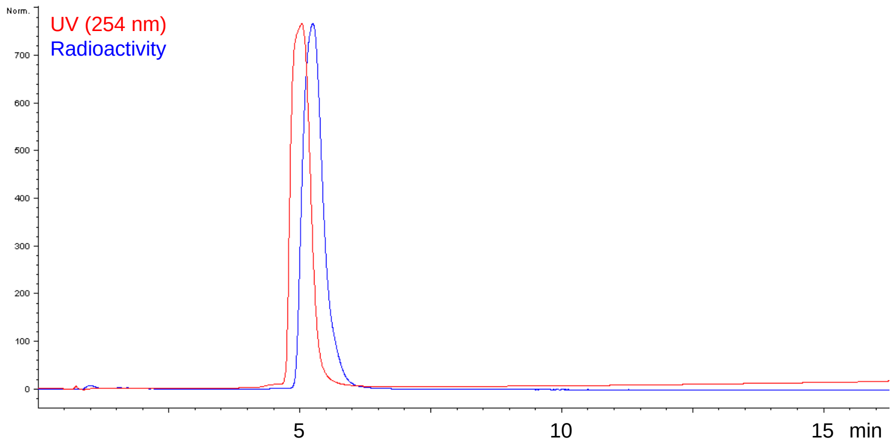

Figure S5. HPLC chromatogram of the isolated compound [^{18}F]**4c**, co-injected with the non-labelled reference **4c**.

[^{18}F]Methyl 2-fluorobenzoate ([^{18}F]5c). The reaction mixture was purified using an Agilent Zorbax® 300SB-C18 column (250 × 9.4 mm) at room temperature and with a flow rate of 3 ml/min. The mobile phase consisted of water and methanol, each containing 0.5% TFA. Elution of the radioactive product was performed with 20% methanol content for 5 min and 45% for 15 min, which was then increased to 90% over 8 minutes. Quality control was performed on an analytical Agilent Zorbax® 300SB-C18 column (150 × 4.6 mm) at room temperature and at a flow rate of 1 ml/min. The mobile phase consisted of water and methanol, each containing 0.5% TFA. Elution was performed with 20% methanol content for 5 min and 45% for 15 min, which was then increased to 90% over 10 minutes. The radiochemical purity of [^{18}F]**5c** was >98% and the identity of the radiochemical product was confirmed by co-elution with the non-radioactive analogue.

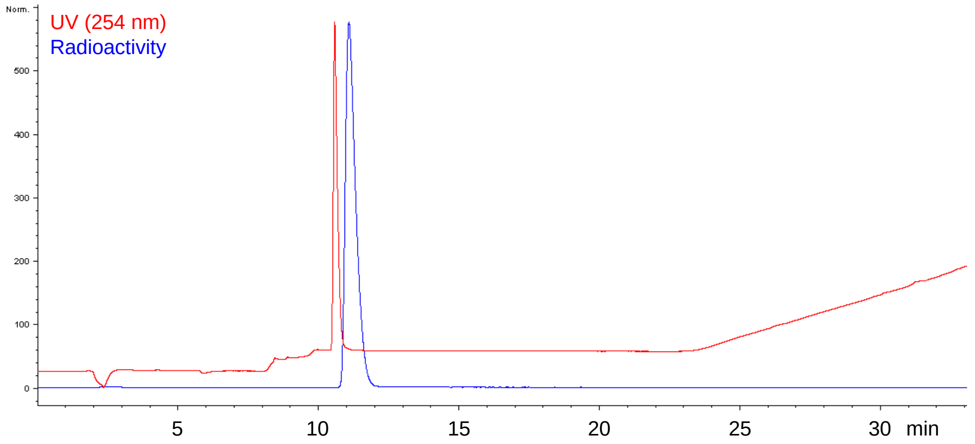

Figure S6. HPLC chromatogram of the isolated compound [^{18}F]**5c**, co-injected with methyl 2-fluorobenzoate.

[^{18}F]Fluorobenzene ([^{18}F]6c). The reaction mixture was purified using a Chromolith® SemiPrep RP18-e column (100 × 10 mm) at room temperature and with a flow rate of 5 ml/min. The mobile phase consisted of water and methanol, each containing 0.1% TFA. Elution starting

with 5% methanol content, kept constant for 5 min, then increased to 95% in 13 min, allowed for isolation of the radioactive product. The radiochemical purity of [^{18}F]**6c** was >98% and the identity of the radiochemical product was confirmed by co-elution with the non-radioactive analogue using the same HPLC method.

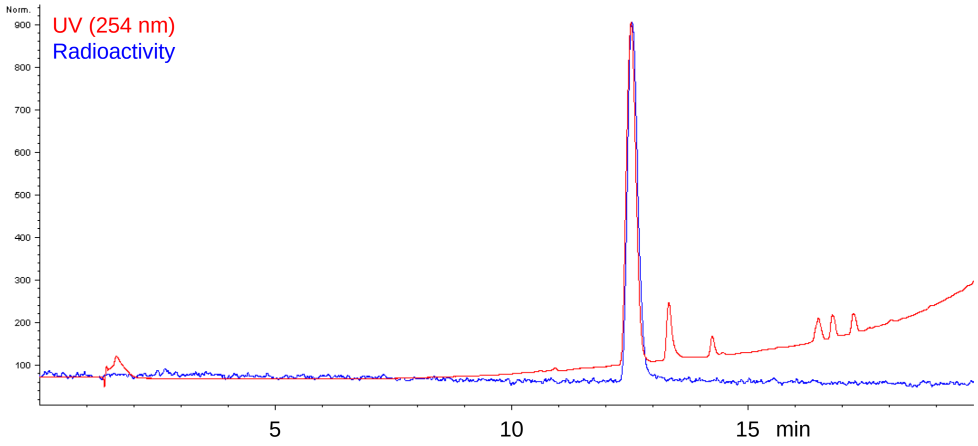

Figure S7. HPLC chromatogram of the isolated compound [^{18}F]**6c**, co-injected with fluorobenzene.

[^{18}F]Ethyl 4-(4-((4-Fluorophenyl)(phenyl)methylene)piperidin-1-yl)butanoate ([^{18}F]8c). The reaction mixture was purified using a Chromolith® SemiPrep RP18-e column (100 × 10 mm) at room temperature and with a flow rate of 5 ml/min. The mobile phase consisted of water and methanol, each containing 0.5% TFA. Gradient elution starting with 10% methanol content that was increased to 55% in 12 min and further to 90% in 3 min allowed for isolation of the radioactive product. Quality control was performed on an analytical Chromolith® Performance RP-18 column (100 × 4.6 mm) at room temperature and at a flow rate of 3 ml/min. The mobile phase consisted of water and methanol, each containing 0.5% TFA. Gradient elution started with 10% methanol content, which was increased to 55% in 12 min, then further to 90% in 3 min. The radiochemical purity of [^{18}F]**8c** was >98% and the identity of the radiochemical product was confirmed by co-elution with the non-radioactive analogue.

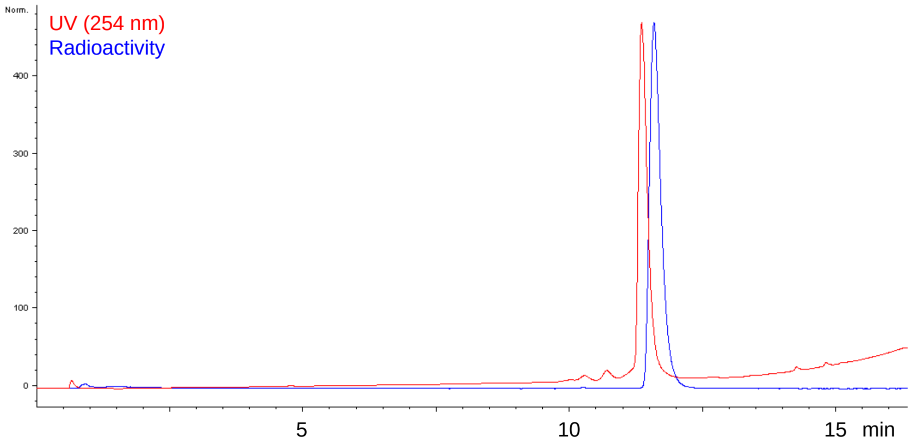

Figure S8. HPLC chromatogram of the isolated compound [^{18}F]**8c**, co-injected with the non-labelled reference **8c**.

[^{18}F]2-Fluoro-5-phenylpyridine ([^{18}F]11c). The reaction mixture was purified using a Chromolith® SemiPrep RP18-e column (100 × 10 mm) at room temperature and with a flow rate of 5 ml/min. The mobile phase consisted of water and methanol, each containing 0.5% TFA. Gradient elution starting with 25% methanol content that was increased to 65% in 12 min, then further to 90% in 2 min allowed for isolation of the radioactive product. Quality

control was performed on an analytical Chromolith® Performance RP-18 column (100 × 4.6 mm) at room temperature and at a flow rate of 3 ml/min. The mobile phase consisted of water and methanol, each containing 0.1% TFA. Gradient elution started with 10% methanol content that was increased to 70% in 15 min. The radiochemical purity of [^{18}F]**11c** was >98% and the identity of the radiochemical product was confirmed by co-elution with the non-radioactive analogue.

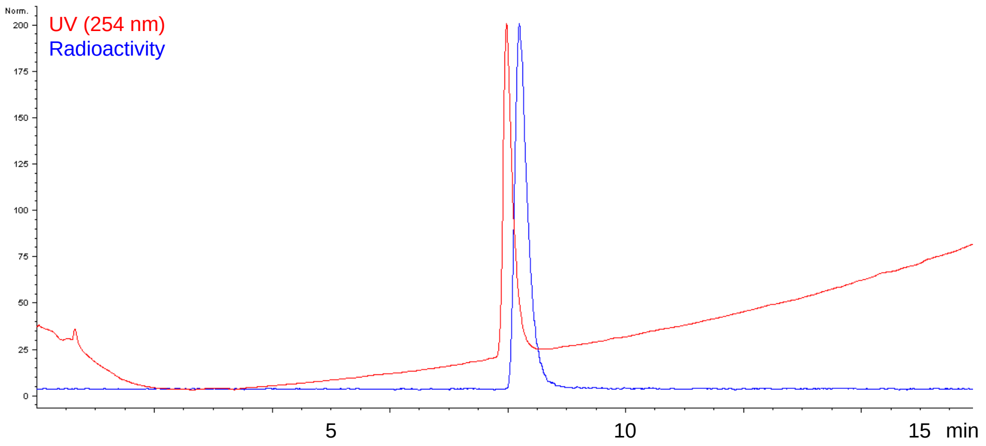

Figure S9. HPLC chromatogram of the isolated compound [^{18}F]**11c**, co-injected with the non-labelled reference **11c**.

[^{18}F]2-Fluoro-5-(pyrrolidin-2-ylmethoxy)pyridine ([^{18}F]12d). The reaction mixture was purified using an Agilent Zorbax® 300SB-C18 column (250 × 9.4 mm) at room temperature and with a flow rate of 3 ml/min. The mobile phase consisted of water and methanol, each containing 0.5% TFA. Gradient elution starting with 5% methanol content, kept constant for 5 min, then increased to 90% in 20 min, allowed for isolation of the radioactive product. Quality control was performed on an analytical Agilent Zorbax® 300SB-C18 column (150 × 4.6 mm) at room temperature and at a flow rate of 1 ml/min. The mobile phase consisted of water and methanol, each containing 0.5% TFA. Elution was performed stepwise with 20% methanol content for 5 min and 45% for 15 min. The radiochemical purity of [^{18}F]**12d** was >98% and the identity of the radiochemical product was confirmed by co-elution with the non-radioactive analogue.

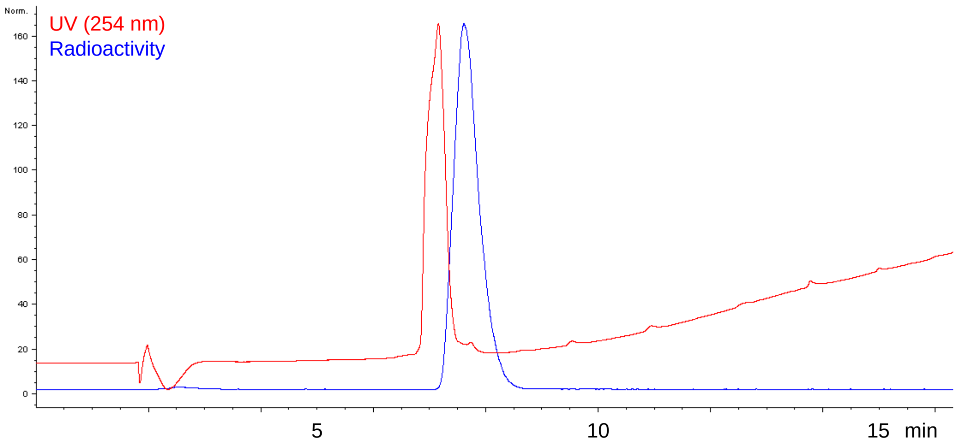

Figure S10. HPLC chromatogram of the isolated compound [^{18}F]**12d**, co-injected with the non-labelled reference **12d**.

[^{18}F]1-(1-(2-Chloro-5-fluorophenyl)ethyl)-1*H*-imidazole ([^{18}F]13c). The reaction mixture was purified using a Chromolith® SemiPrep RP18-e column (100 × 10 mm) at room

temperature and with a flow rate of 5 ml/min. The mobile phase consisted of water and methanol, each containing 0.5% TFA. Gradient elution started with 10% methanol content that was kept constant for 10 min, increased to 55% in 12 min, then further to 90% in 3 min. This system allowed for determination of analytical RCY and for isolation of the radioactive product. The radiochemical purity of [^{18}F]**13c** was >98% and the identity of the radiochemical product was confirmed by co-elution with the non-radioactive analogue using the same HPLC system.

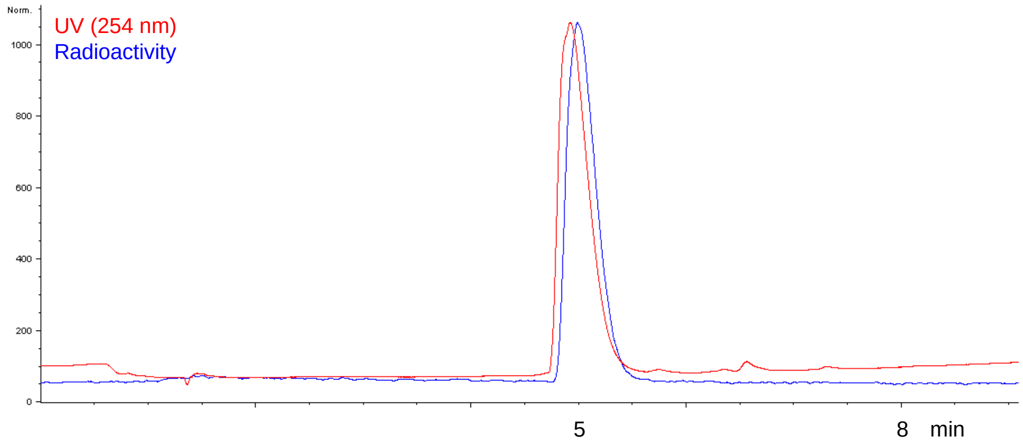

Figure S11. HPLC chromatogram of the isolated compound [^{18}F]**13c**, co-injected with the non-labelled reference **13c**.

c. Preparation of Compounds [^{18}F]10d and [^{18}F]14c for Preclinical Studies

[^{18}F]4-(4-((4-Fluorophenyl)(phenyl)methylene)piperidin-1-yl)butan-1-ol ([^{18}F]10d). [^{18}F]Fluoride (2.0 GBq) in water was trapped on a Sep-Pak® QMA cartridge, released with a solution (0.5 ml) of Kryptofix 222 (30 mM) and potassium bicarbonate (30 mM) in acetonitrile: water (85: 15). After removing the solvent by heating at 90 °C under a stream of nitrogen, acetonitrile (0.5 ml) was added, and the distillation was continued at 90 °C. This procedure was repeated and the reaction vial was subsequently capped. Compound **10b** (5 mg) dissolved in DMSO (0.5 ml) was subsequently added and the mixture was stirred at 110 °C for 15 minutes. After cooling, the reaction was quenched and further diluted with water to a volume of 20 ml. This solution was filtered over a Sep-Pak® SPE C-18 light cartridge. The labelled product was released with a solution of HCl in ethanol (1.25 M, 0.5 ml). The filtrate was heated at 90 °C for 15 minutes. After cooling, the reaction was quenched with water (1.5 ml) and purified by HPLC using a Chromolith® SemiPrep RP18-e column (100 × 10 mm) at room temperature and with a flow rate of 5 ml/min. The mobile phase consisted of water and methanol, each containing 0.5% TFA. Gradient elution starting with 10% methanol content that was increased to 55% in 12 min, then further to 90% in 3 min, allowed for isolation of the radioactive product. The obtained solution was diluted with water to a final volume of 20 ml, trapped on a Sep-Pak® SPE C-18 light cartridge, and the labelled product was released with ethanol (0.5 ml). After having reduced the volume to <0.1 ml under a stream of nitrogen, the solution was diluted with saline to give a final ethanol concentration of 5% and sterilized by filtration. Quality control was performed on a Chromolith® Performance RP18 column (100 × 4.6 mm) using water and methanol (each containing 0.5% TFA; gradient elution with a flow rate of 3 ml/min starting with 10% methanol content that was increased to 55% in 12 min and further to 90% in 3 min). The radiochemical purity of [^{18}F]**10d** was >98% and the specific activity of the tracer was 4.0 GBq/µmol. The identity of the radiochemical product was confirmed by co-elution with the non-radioactive analogue.

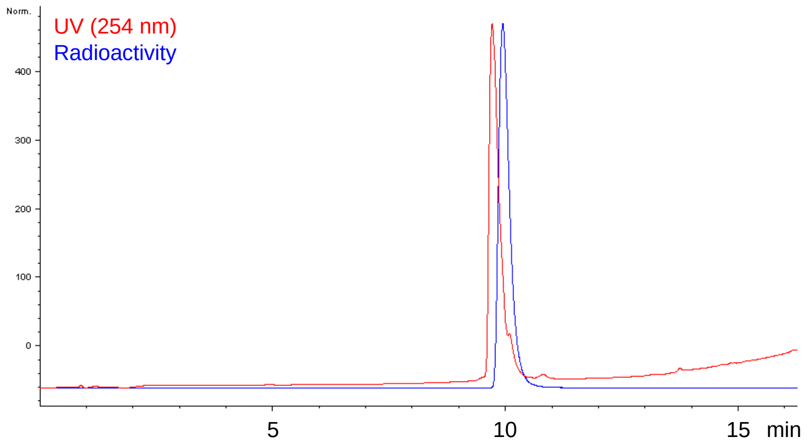

Figure S12. HPLC chromatogram of the isolated compound [18F]**10d**, co-injected with the non-labelled reference **10d**.

[18F]*N*-(1-(3-Fluorobenzyl)quinolin-4(1*H*)-ylidene)pentan-1-amine ([18F]14c). [18F]Fluoride (650 MBq) in water was trapped on a Sep-Pak® QMA cartridge, released with a solution (0.5 ml) of Kryptofix 222 (30 mM) and potassium bicarbonate (30 mM) in acetonitrile: water (85: 15). After removing the solvent by heating at 90 °C under a stream of nitrogen, acetonitrile (0.5 ml) was added, and the distillation was continued at 90 °C. This procedure was repeated and the reaction vial was subsequently capped. Compound **14b** (5 mg) dissolved in DMSO (0.5 ml) was subsequently added and the mixture was stirred at 120 °C for 15 minutes. After cooling, the reaction was quenched with water (1.5 ml) and purified by radio-HPLC using an Agilent Zorbax® 300SB-C18 column (250 × 9.4 mm) at room temperature and with a flow rate of 3 ml/min. The mobile phase consisted of water and methanol, each containing 0.5% TFA. The radioactive product was isolated using a gradient starting with 10% methanol content kept constant for 5 min and then increased to 50%. The methanol content was subsequently raised from 50 to 60% in 10 min, further to 63% in 5 min, to 70% in 5 min, and finally to 90% in 5 min. Only the most concentrated fraction of the product peak was isolated. The obtained solution was diluted with water to a final volume of 20 ml, trapped on a Sep-Pak® SPE C-18 light cartridge, and the labelled product was released with ethanol (0.5 ml). After having reduced the volume to <0.1 ml under a stream of nitrogen, the solution was diluted with saline to give a final ethanol concentration of 5% and sterilized by filtration. Quality control was performed on an analytical Agilent Zorbax® 300SB-C18 column (150 × 4.6 mm) at room temperature and with a flow rate of 1 ml/min. The mobile phase consisted of water and methanol, each containing 0.5% TFA. Gradient elution started with 30% methanol content that was kept constant for 5 min, then increased to 50%, raised to 60% in 10 min, then further to 63% in 5 min. The radiochemical purity of [18F]**14c** was >98% and the specific activity of the tracer was 2.9 GBq/µmol. The identity of the radiochemical product was confirmed by co-elution with the non-radioactive analogue.

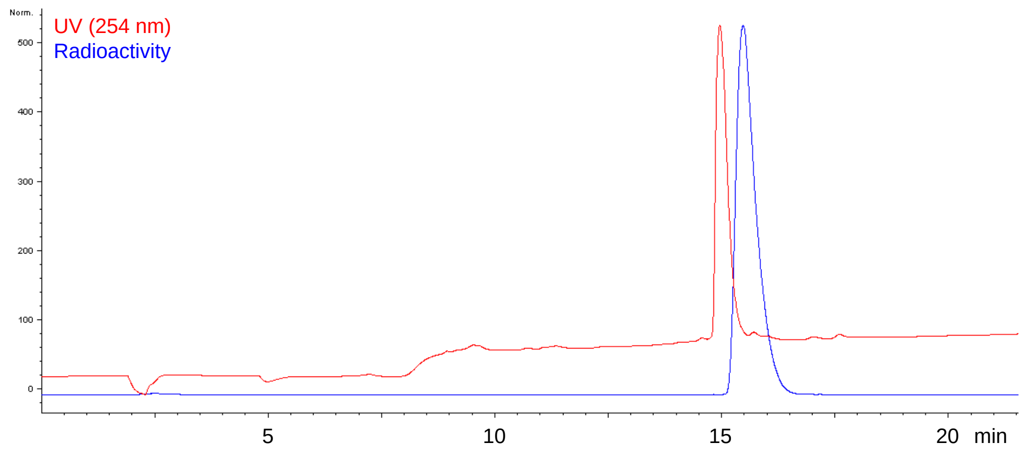

Figure S13. HPLC chromatogram of the isolated compound [^{18}F]**14c**, co-injected with the non-labelled reference **14c**.

d. Labelling of [^{18}F]1c and [^{18}F]11c at 50 °C

Labelling was performed as described above (*cf.* page S28), with stirring for 15 minutes at 50 °C. After cooling, the reaction was quenched with water (1.5 ml) and an aliquot of the crude mixture was analysed by radio-HPLC.

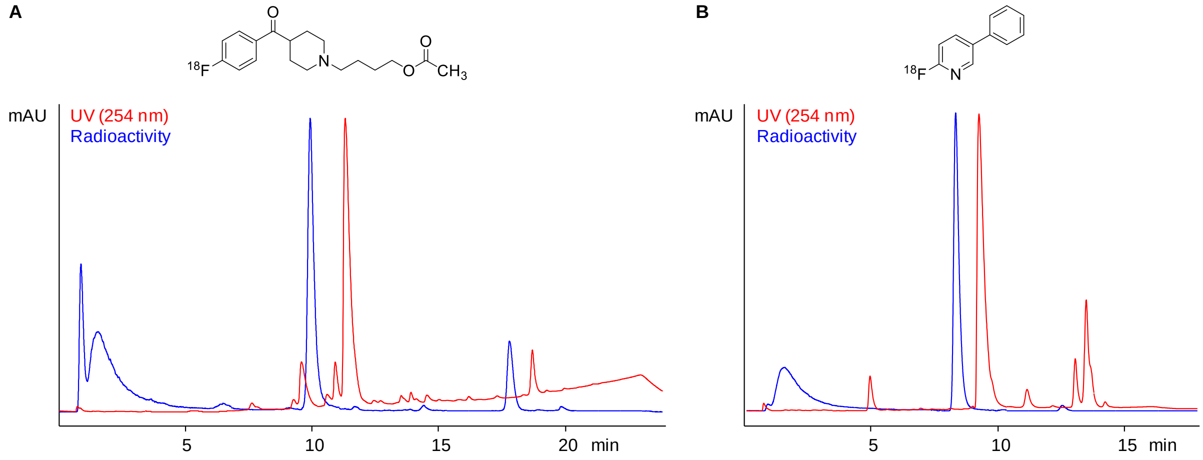

Figure S14. Radio-HPLC chromatograms of labelling reactions at 50 °C giving compounds [^{18}F]**1c** (A) and [^{18}F]**11c** (B).

Reagents and conditions: [^{18}F]F^-, $KHCO_3$, K_{222}, DMSO, 15 min, 50 °C.

5. Animal experiments

All animal work was performed in compliance with the United Kingdom Home Office's Animals (Scientific Procedures) Act 1986 and with approval of the University College London (UCL) Animal Ethics Committee.

Wild-type albino mice (FVB or Balb/C, Charles River Laboratories, UK) were allowed to acclimatize for at least one week at the animal facilities at the UCL Centre for Advanced Biomedical Imaging, and they were given food and water *ad libitum*. Dynamic PET imaging was performed using a nanoScan® PET-CT system manufactured by Mediso (Medical Imaging Systems, Budapest, Hungary). Mice were anaesthetized with isoflurane (2% in oxygen) and placed on the preheated bed of the scanner (set at 38 °C). The respective radiotracer (5–10 MBq in 100–250 µl saline solution) was injected into the tail vein via intravenous cannulation. After injection, the catheter was carefully removed. Breathing rate and body temperature of the animals were closely monitored during the dynamic PET scans and, if necessary, the isoflurane dose was adjusted. Scans were recorded over two hours, and the animals were subsequently sacrificed by cervical dislocation. Quantification of tissue uptake was carried out using the software package VivoQuant 1.23 (inviCRO, Boston, USA).

6. Appendix: ^{1}H and ^{13}C NMR spectra

(4-Bromophenyl)(phenyl)sulfane (A)

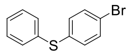

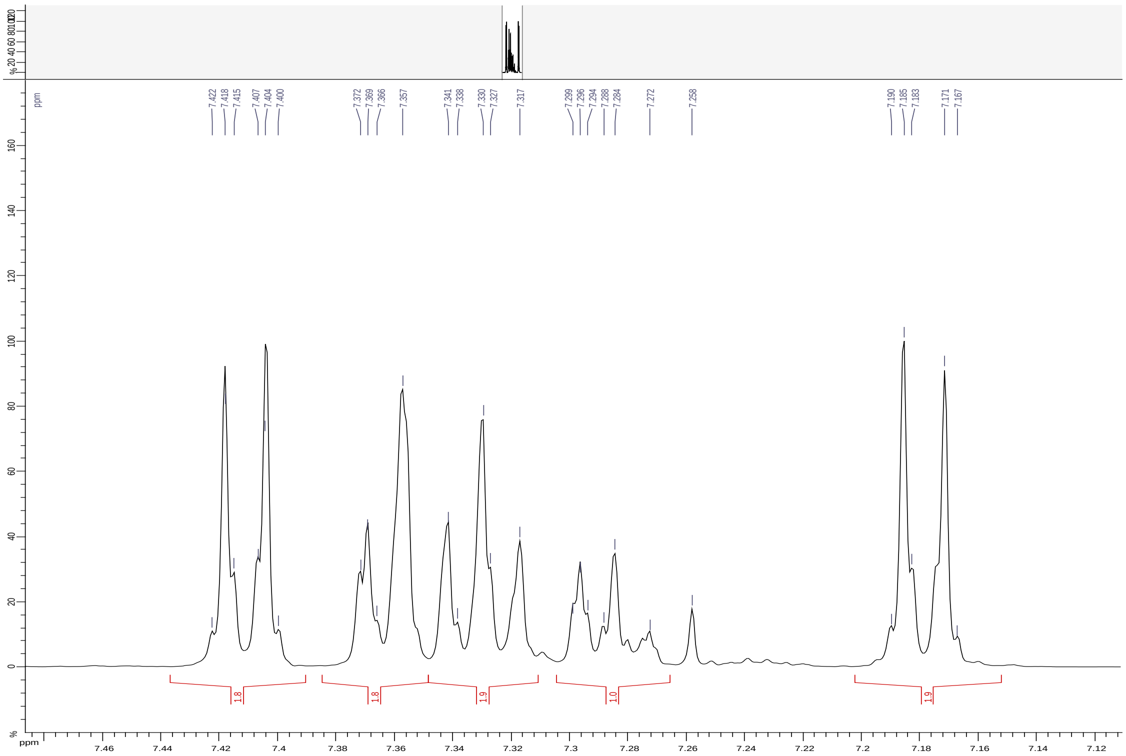

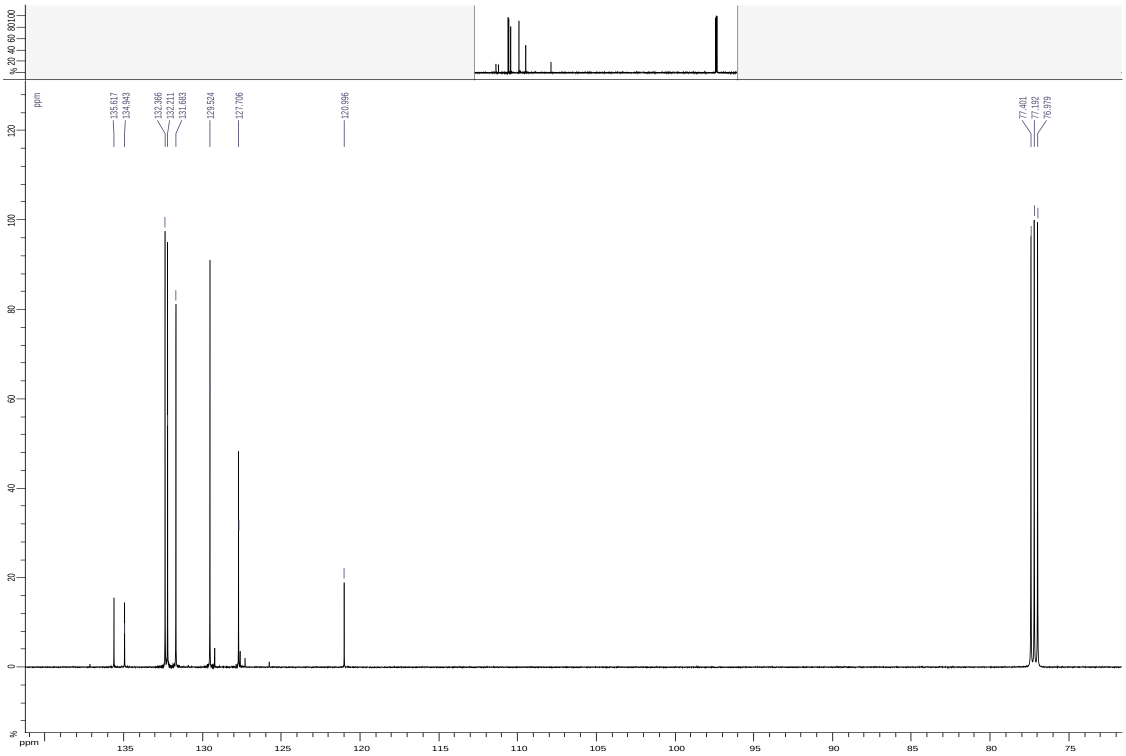

tert-Butyl 4-(methoxy(methyl)carbamoyl)piperidine-1-carboxylate (B)

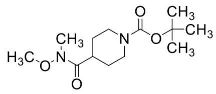

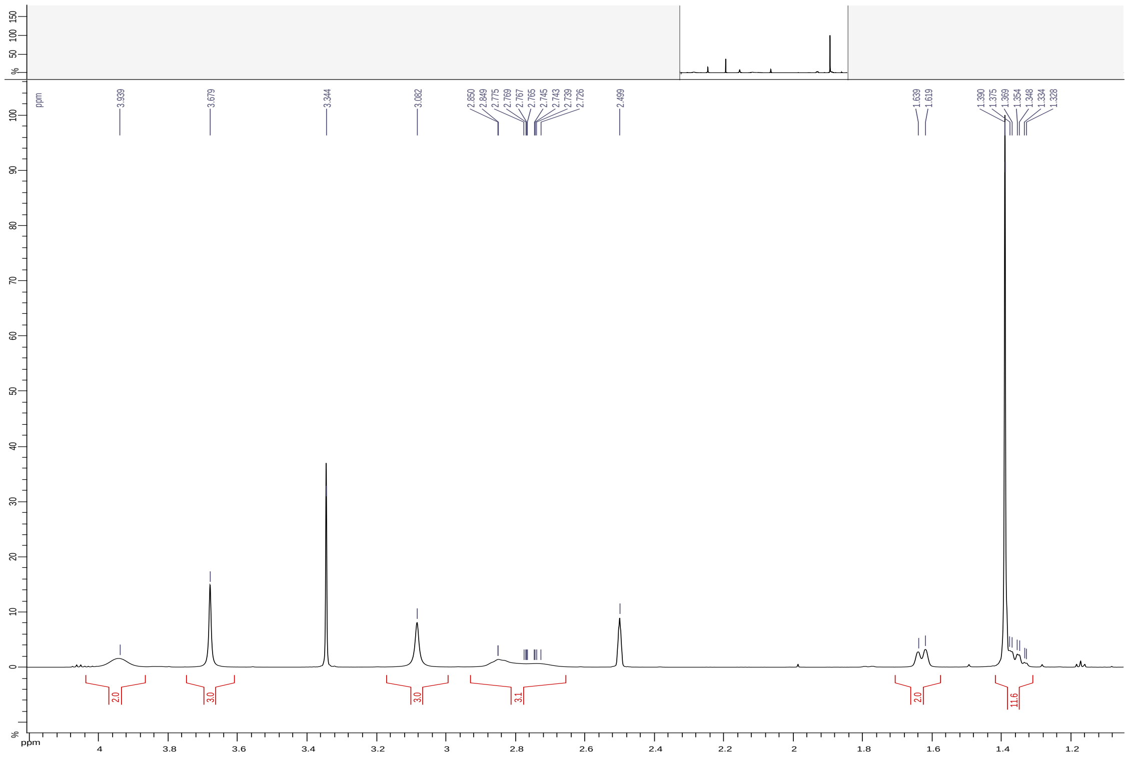

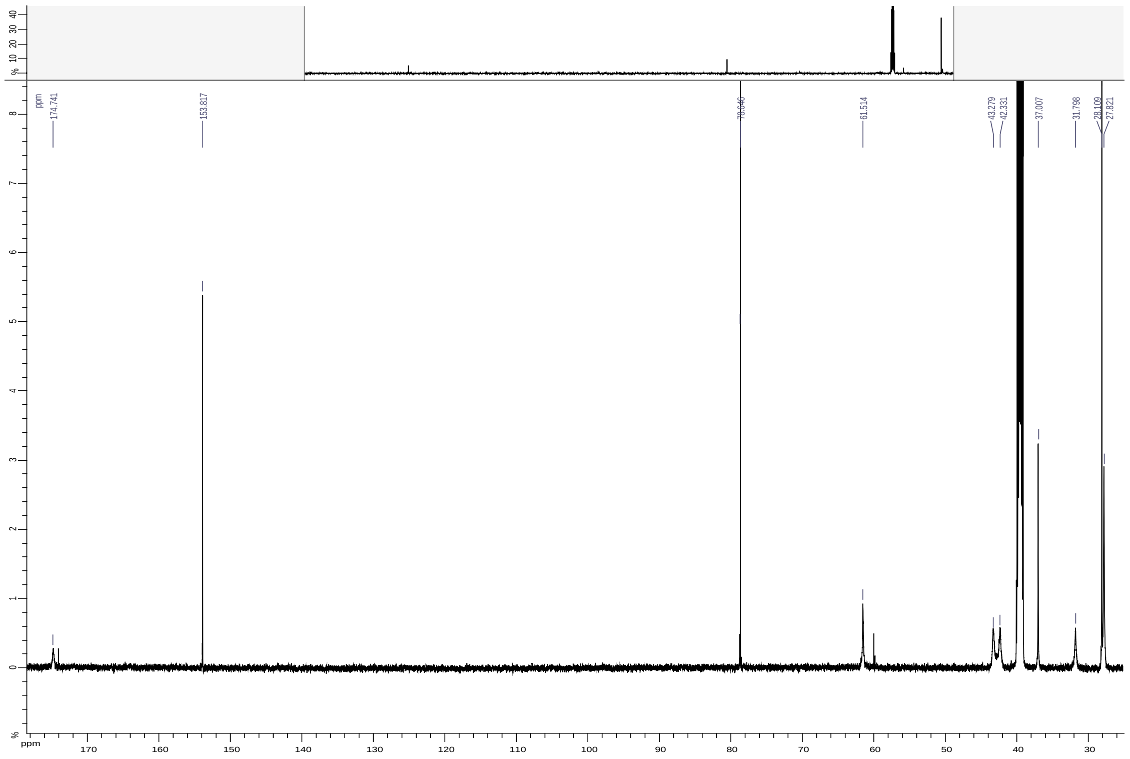

tert-Butyl 4-(4-(phenylthio)benzoyl)piperidine-1-carboxylate (C)

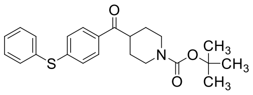

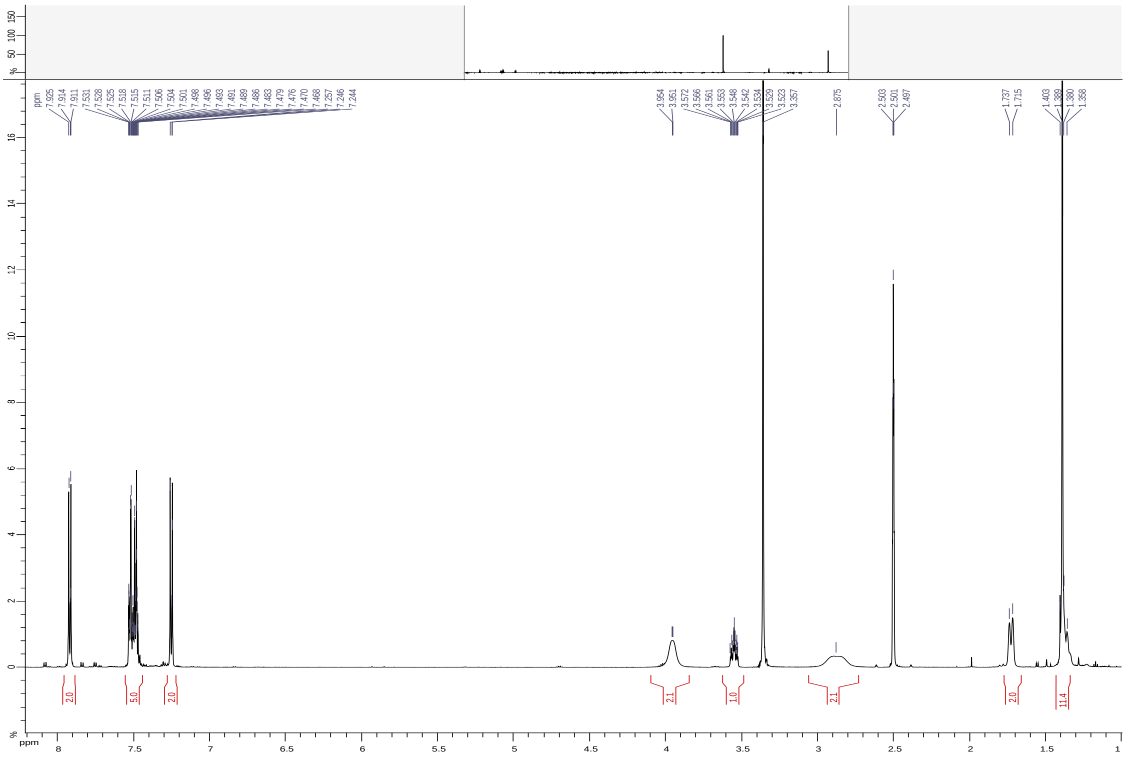

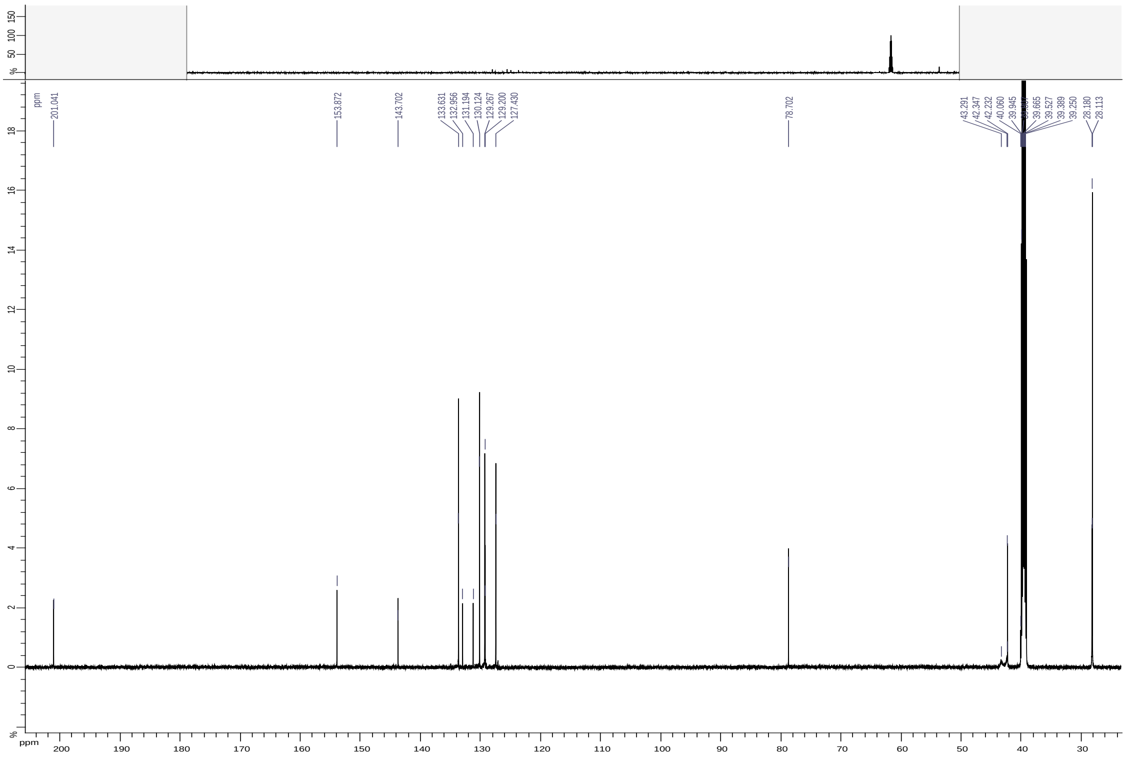

(4-(Phenylthio)phenyl)(piperidin-4-yl)methanone hydrochloride (4a)

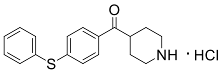

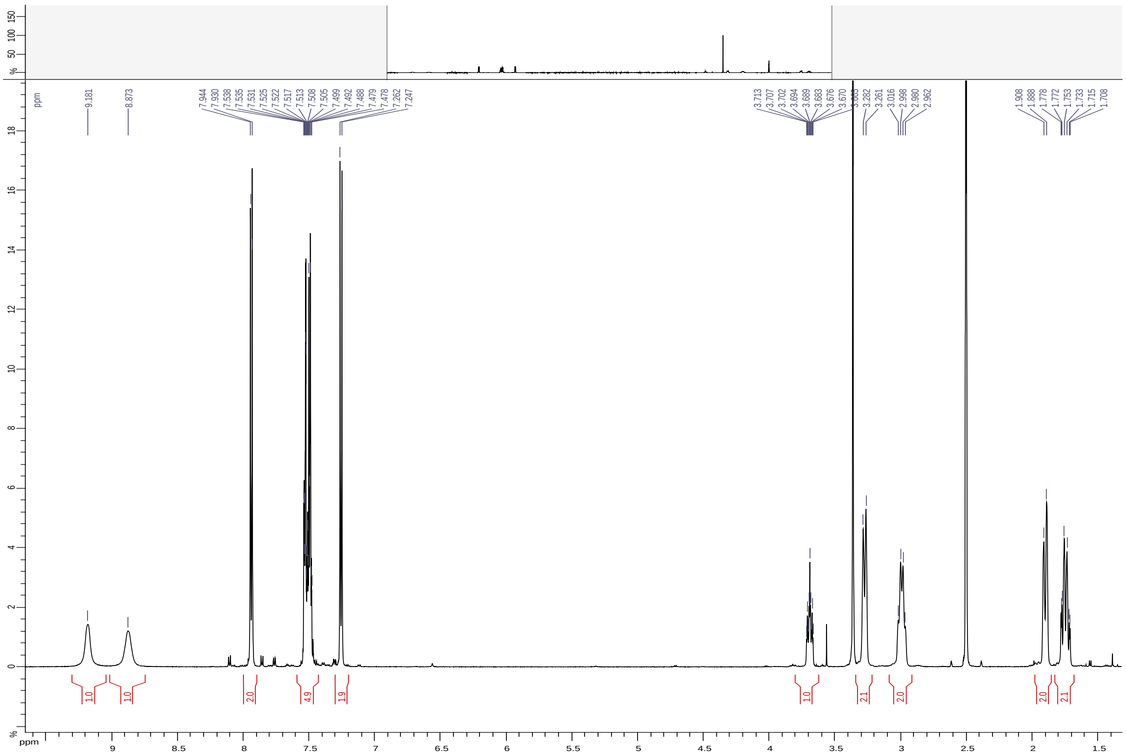

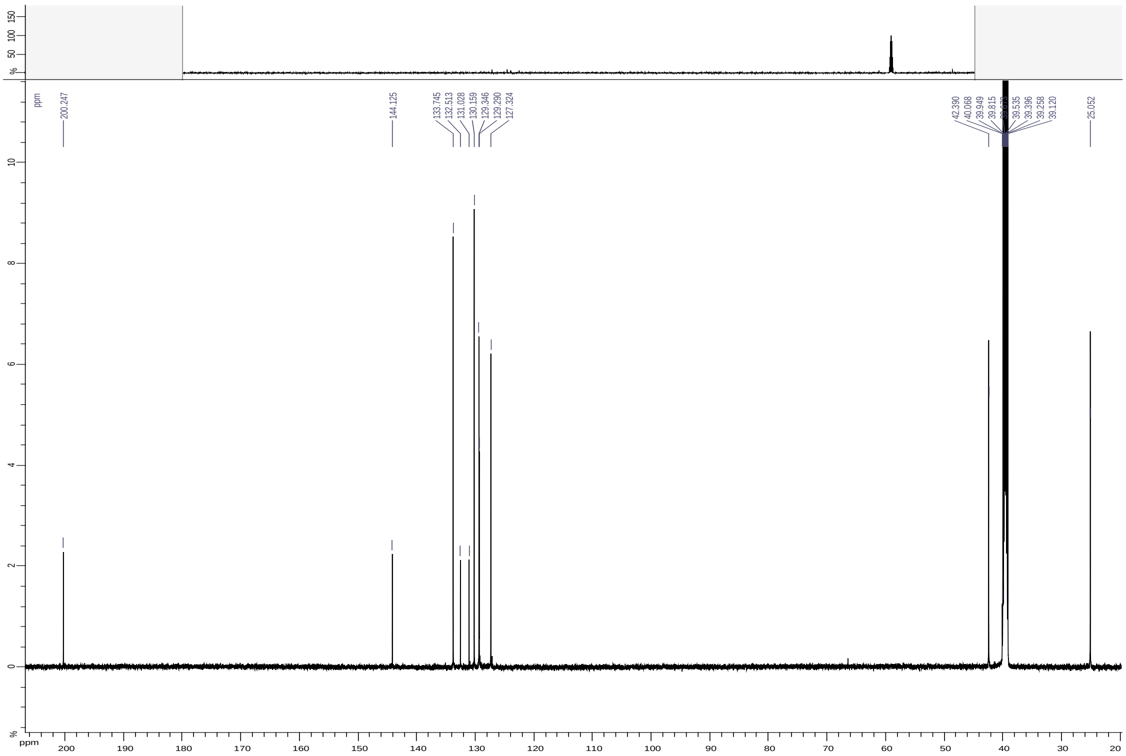

4-(4-(4-(Phenylthio)benzoyl)piperidin-1-yl)butyl acetate (1a)

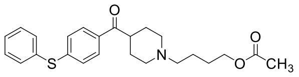

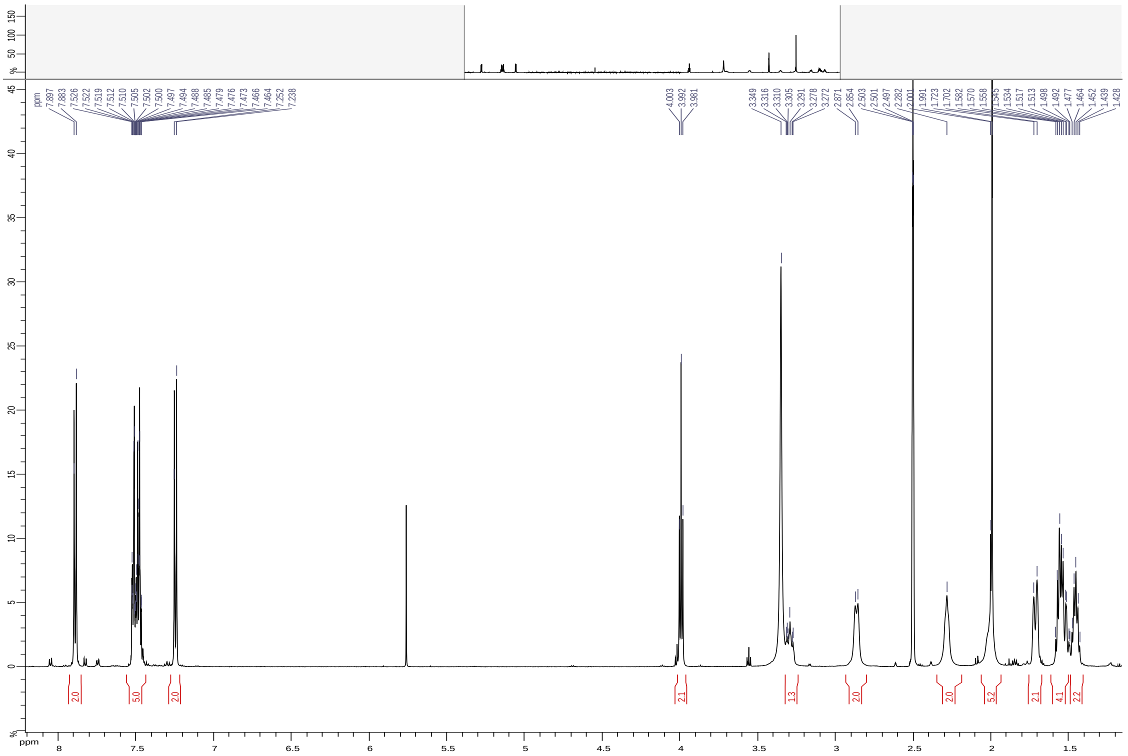

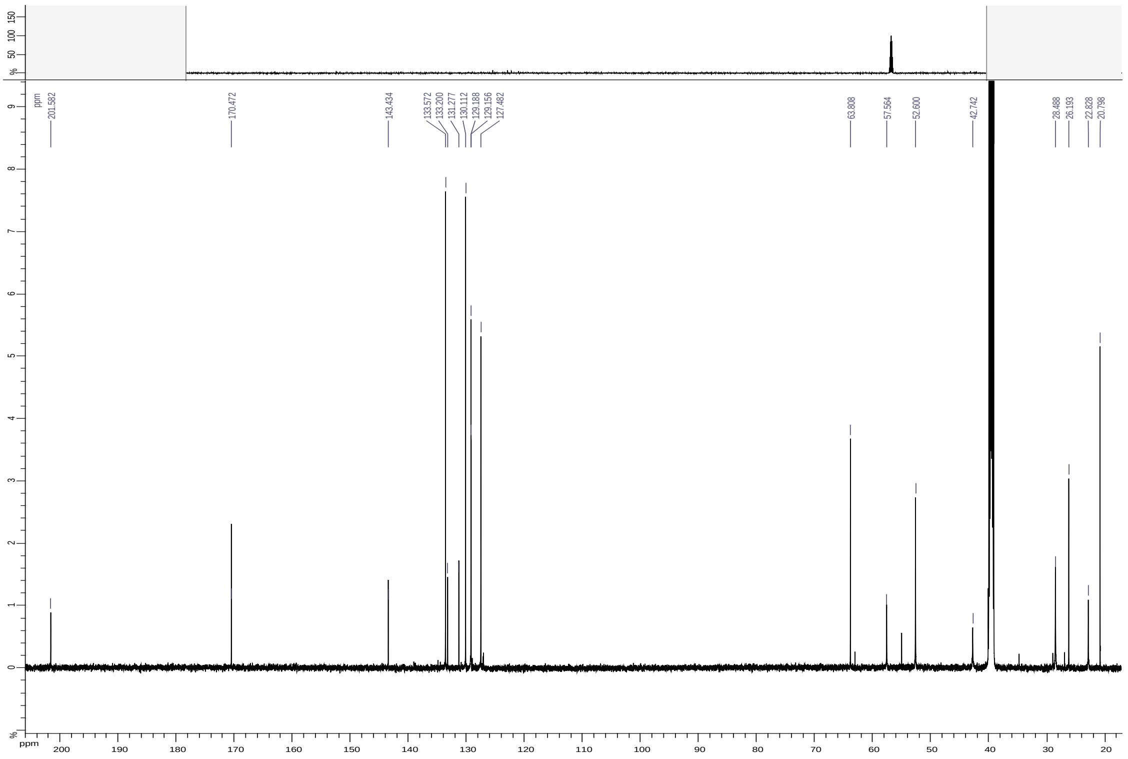

(4-(1-(4-Acetoxybutyl)piperidine-4-carbonyl)phenyl)diphenylsulfonium trifluoromethanesulfonate (1b)

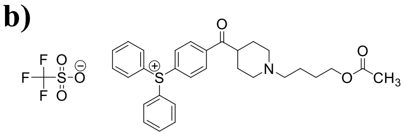

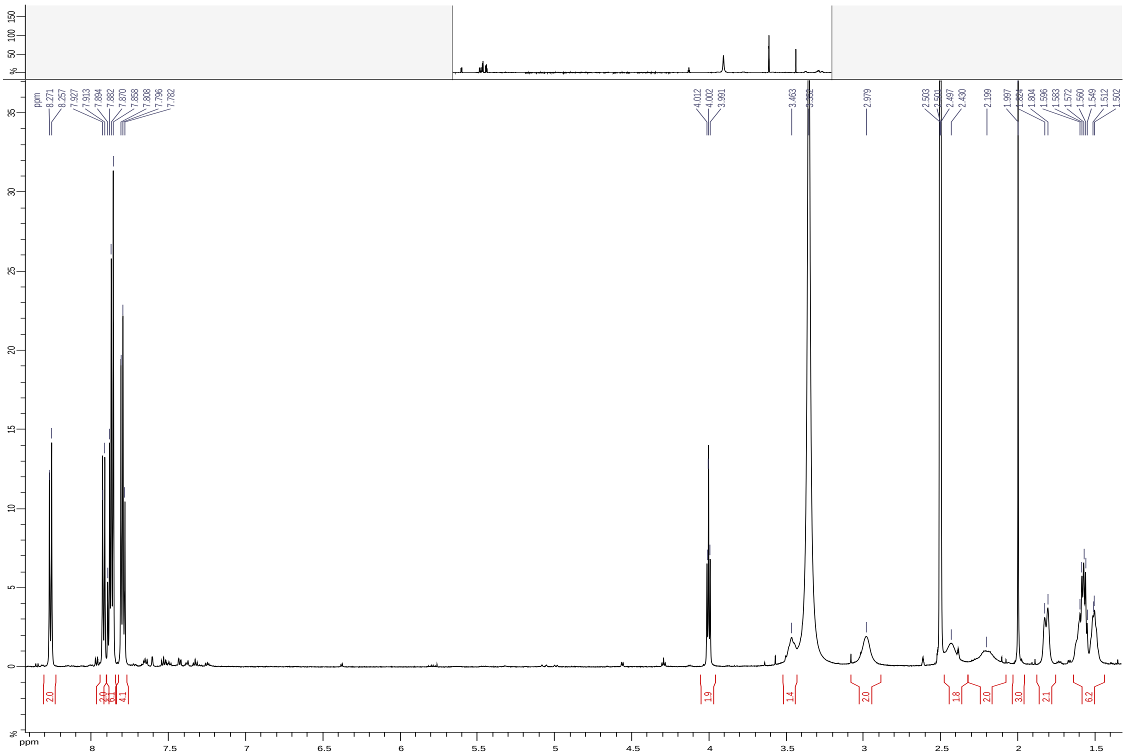

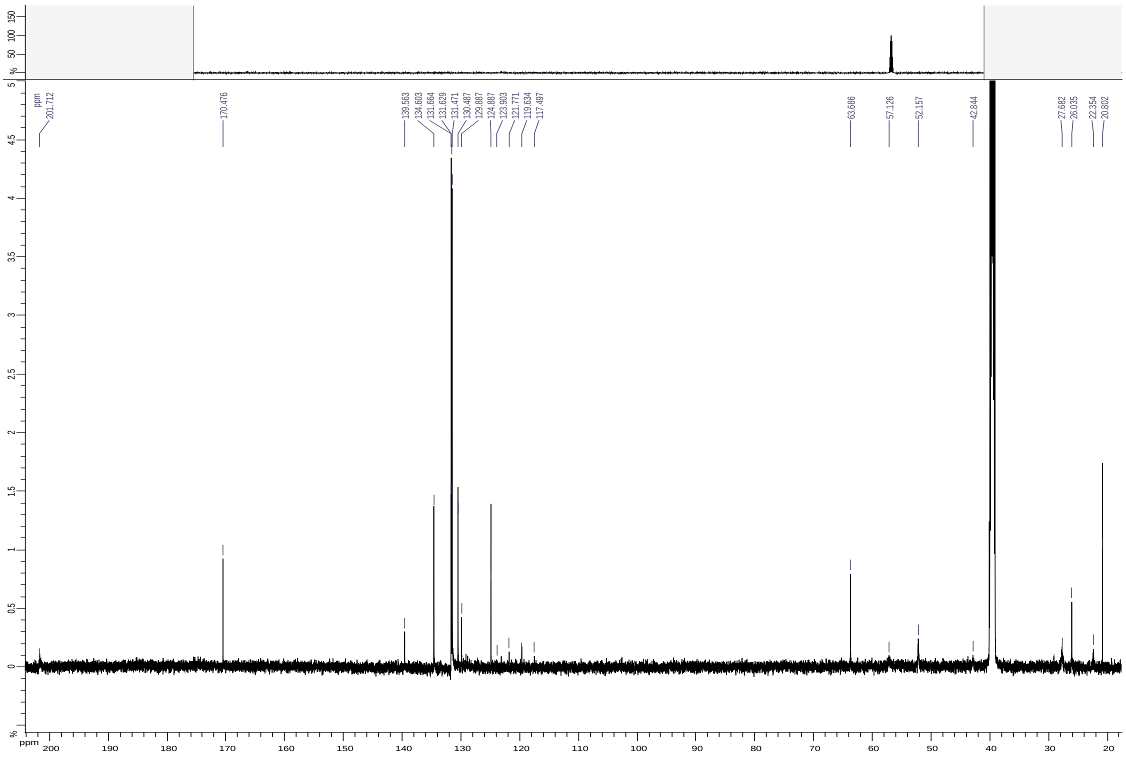

(1-(4-Hydroxybutyl)piperidin-4-yl)(4-(phenylthio)phenyl)methanone (3a)

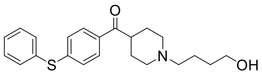

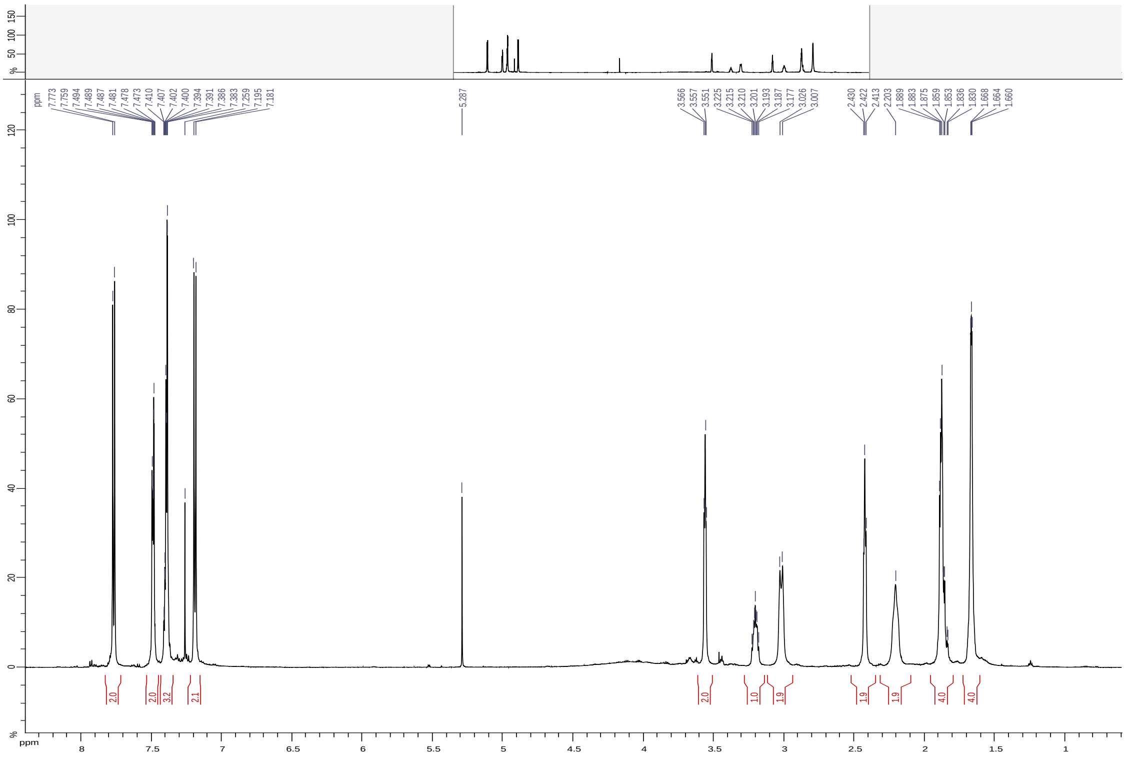

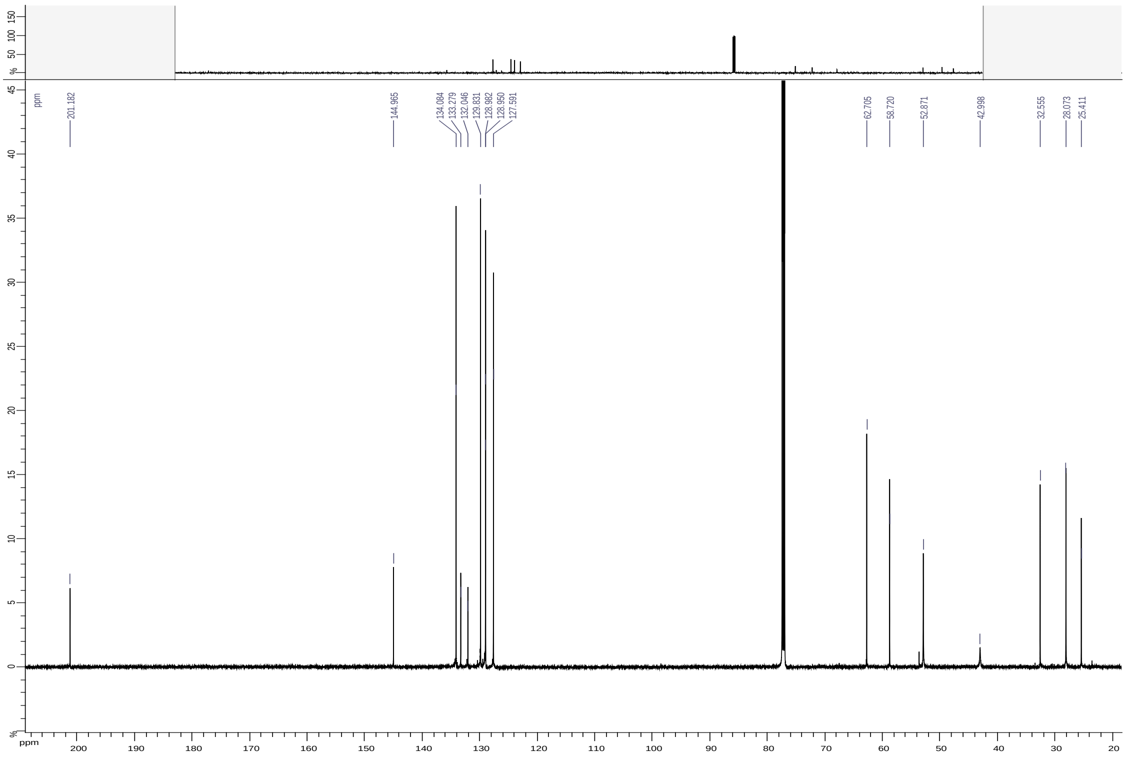

(4-(1-(4-Hydroxybutyl)piperidine-4-carbonyl)phenyl)diphenylsulfonium trifluoromethanesulfonate (3b)

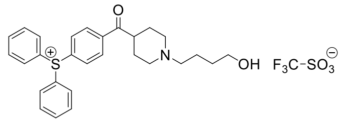

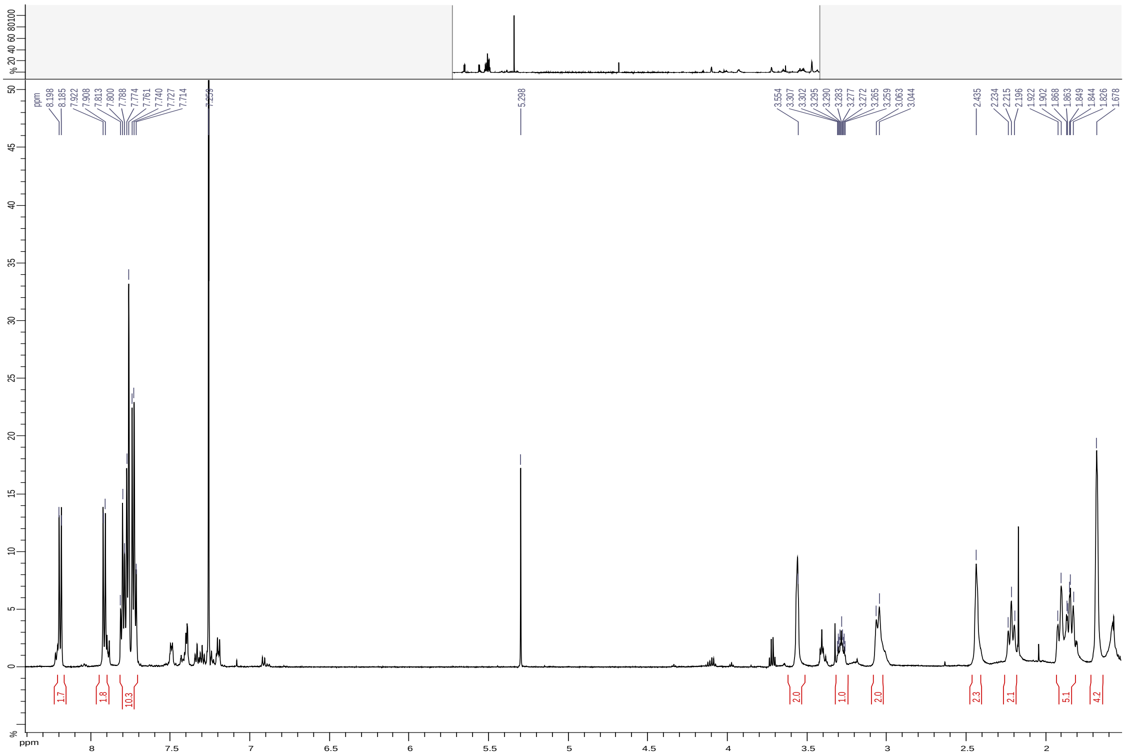

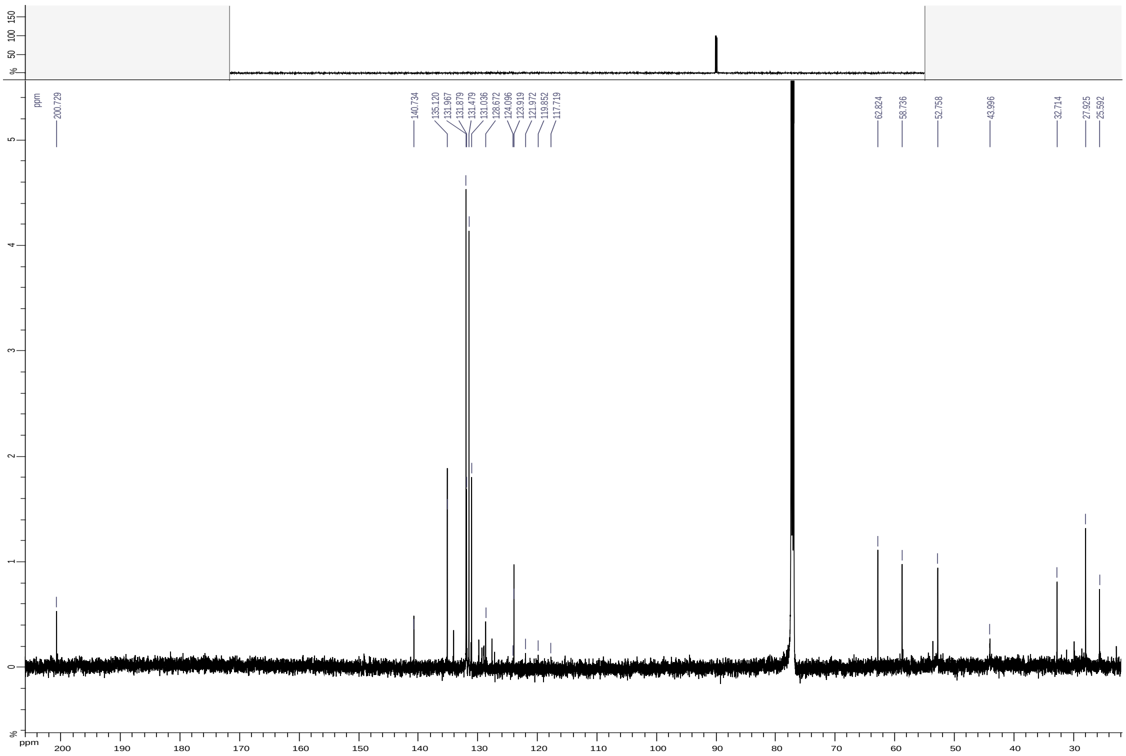

Diphenyl(4-(piperidine-4-carbonyl)phenyl)sulfonium trifluoromethanesulfonate (4b)

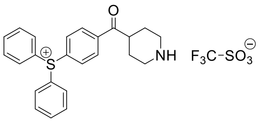

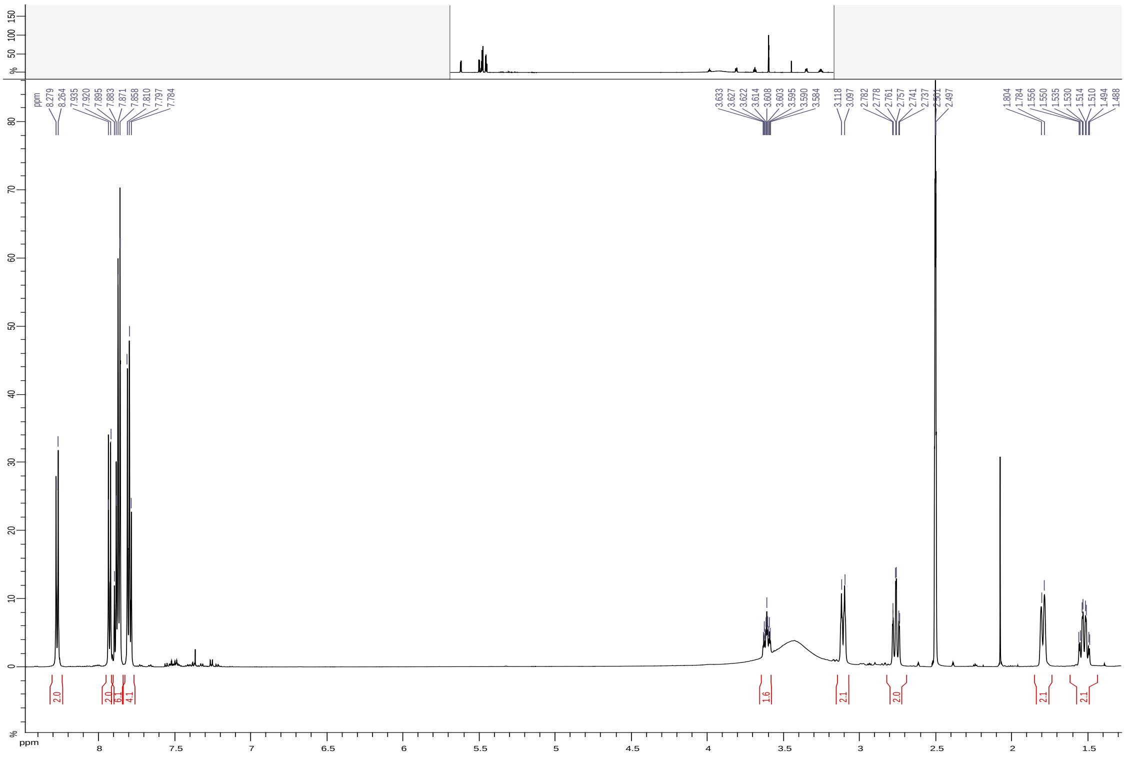

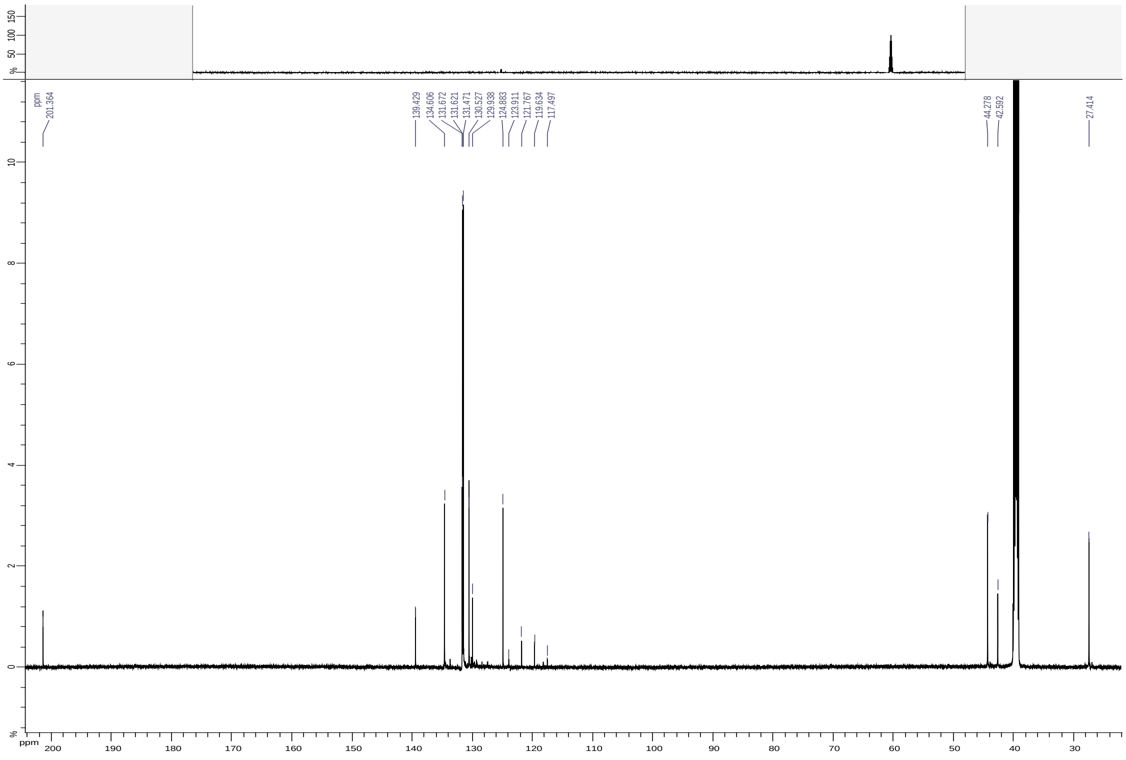

tert-Butyl 4-(hydroxy(phenyl)(4-(phenylthio)phenyl)methyl)piperidine-1-carboxylate (D)

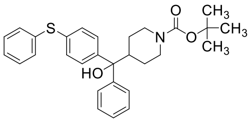

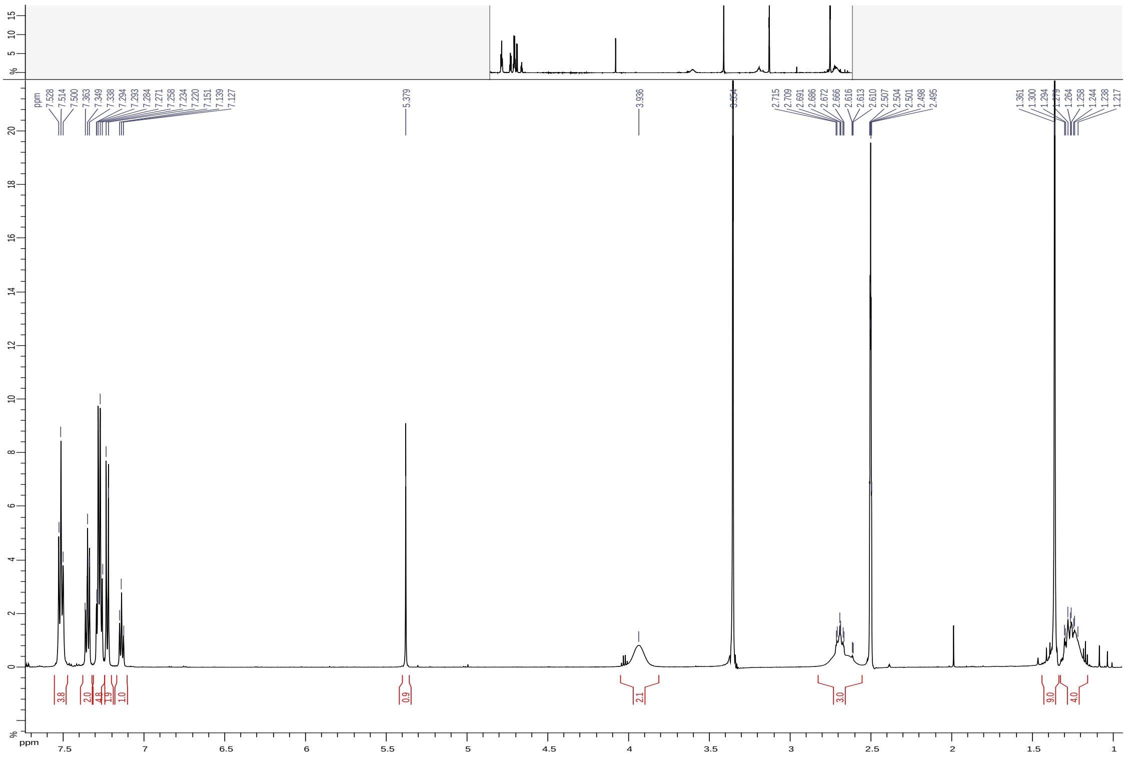

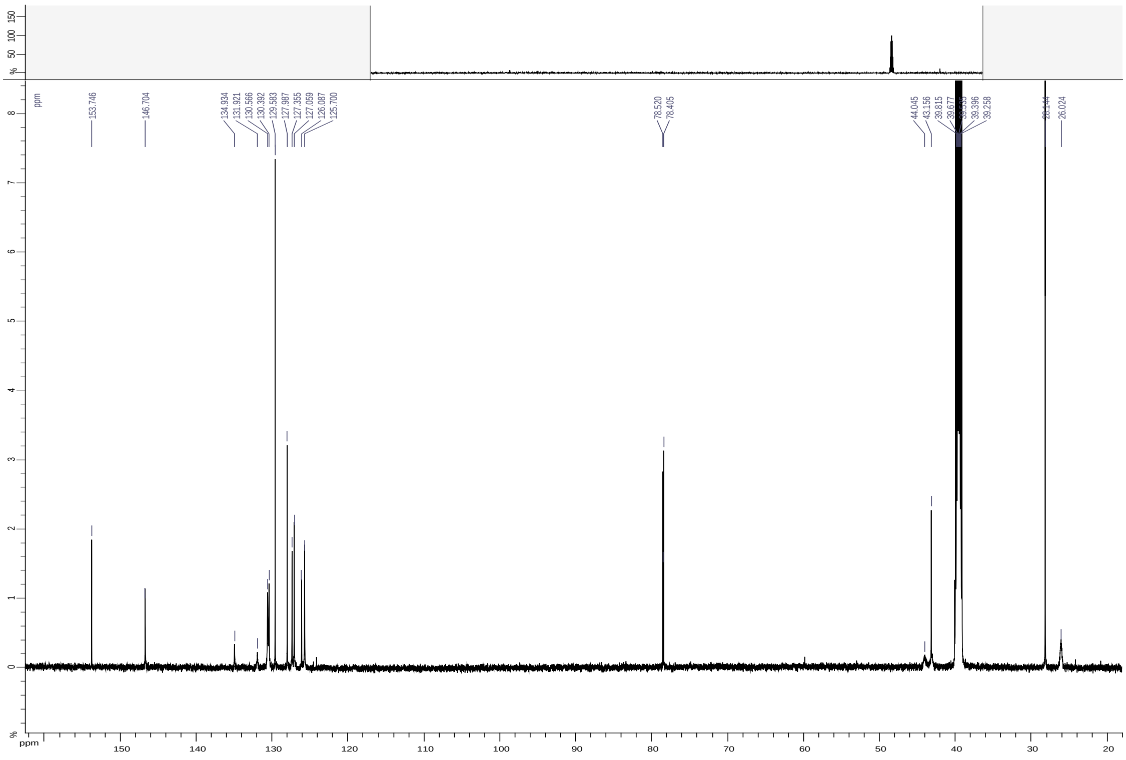

4-(Phenyl(4-(phenylthio)phenyl)methylene)piperidine (E)

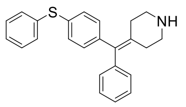

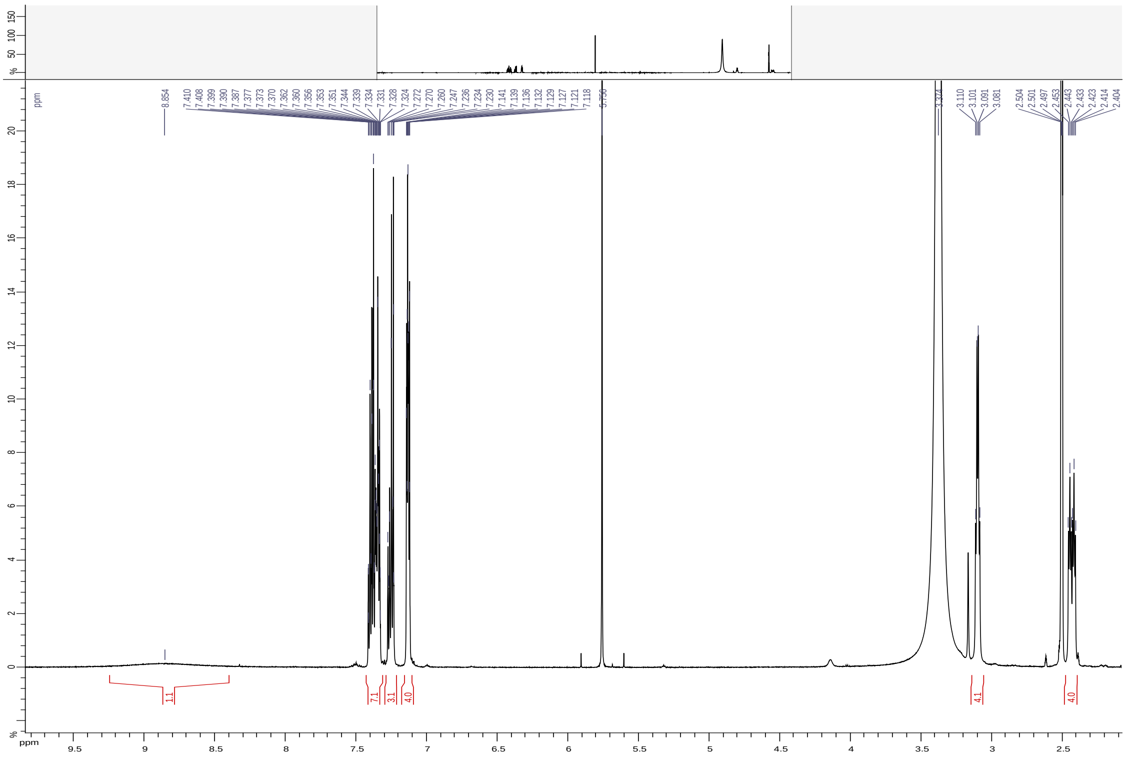

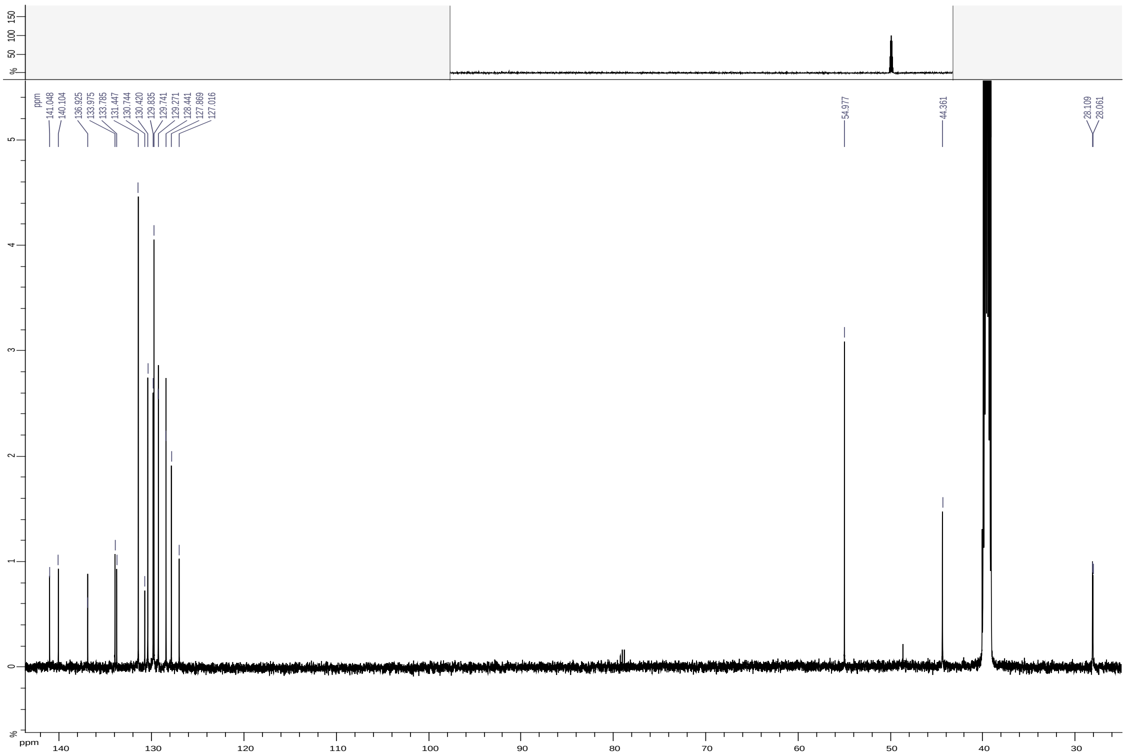

Ethyl 4-(4-(Phenyl(4-(phenylthio)phenyl)methylene)piperidin-1-yl)butanoate (8a)

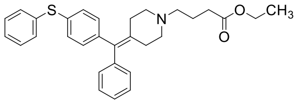

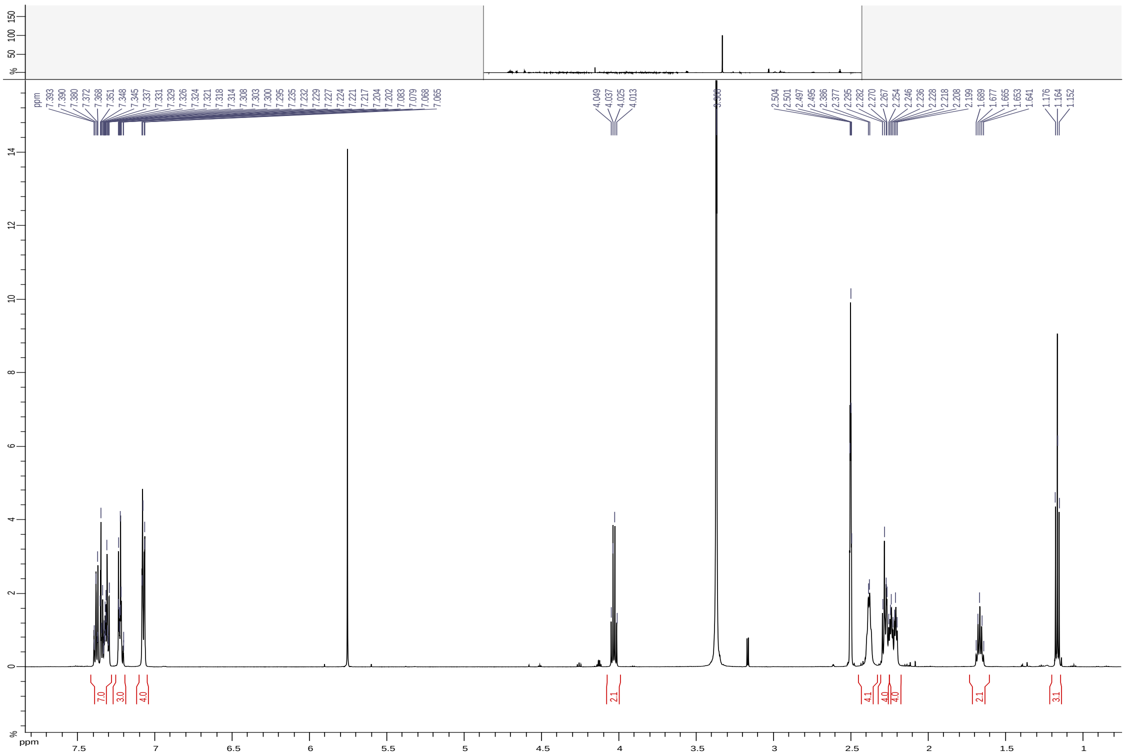

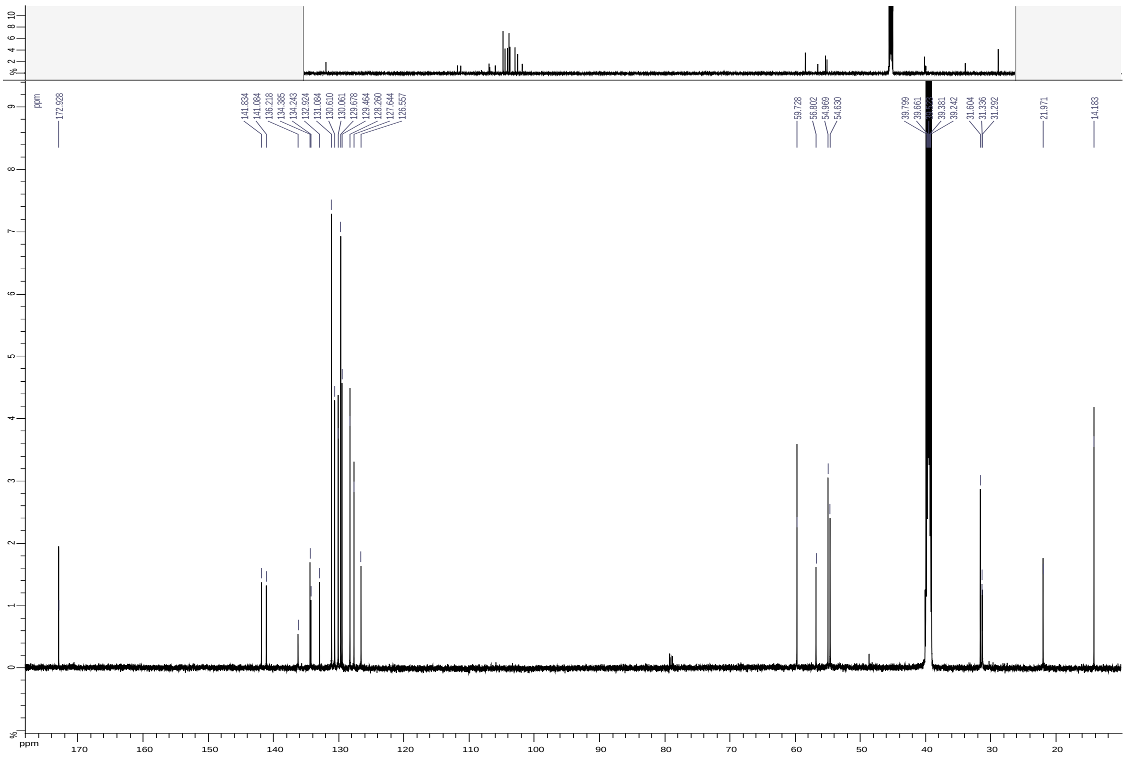

(4-((1-(4-Ethoxy-4-oxobutyl)piperidin-4-ylidene)(phenyl)methyl)phenyl)diphenyl-sulfonium trifluoromethanesulfonate (8b)

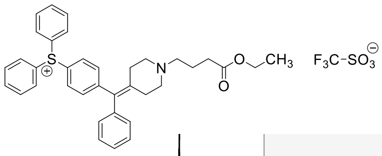

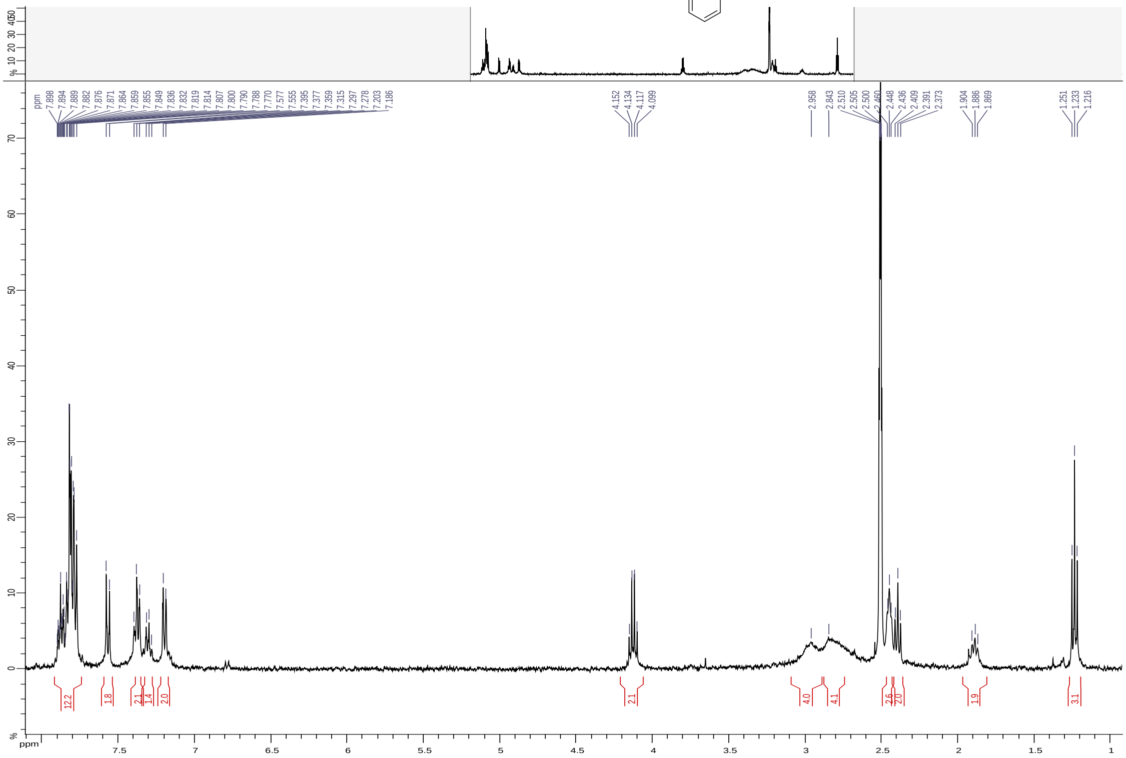

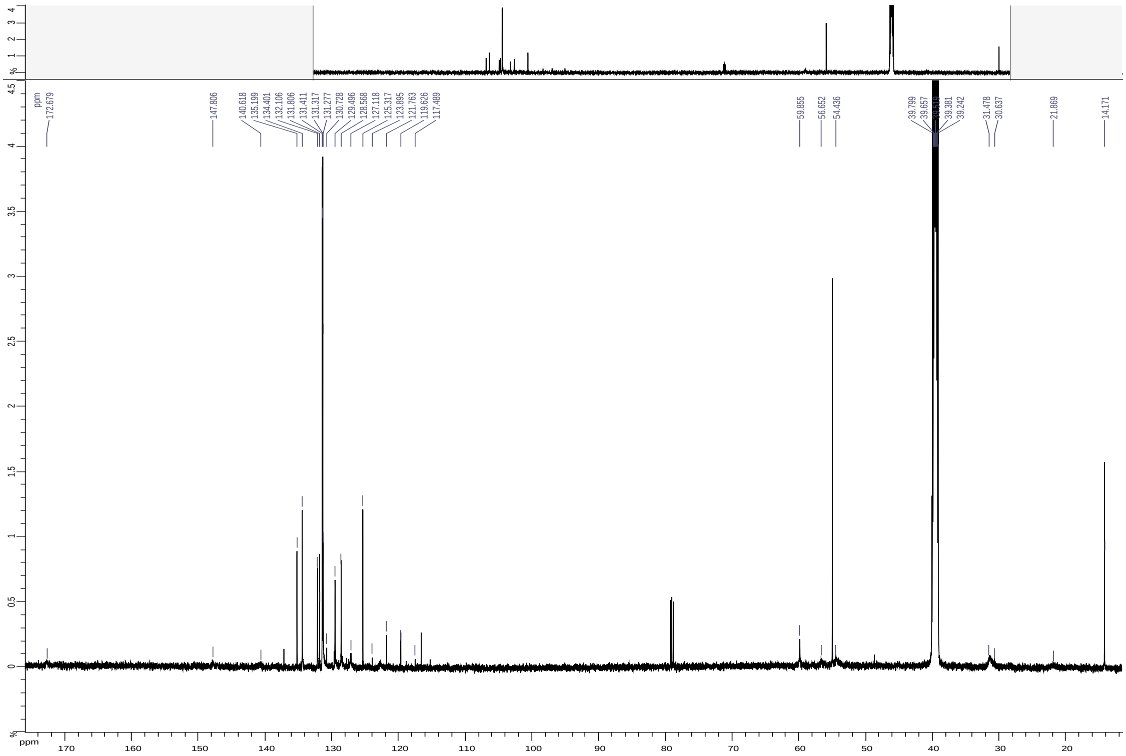

Phenyl(4-(phenylthio)phenyl)methanone (2a)

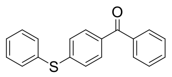

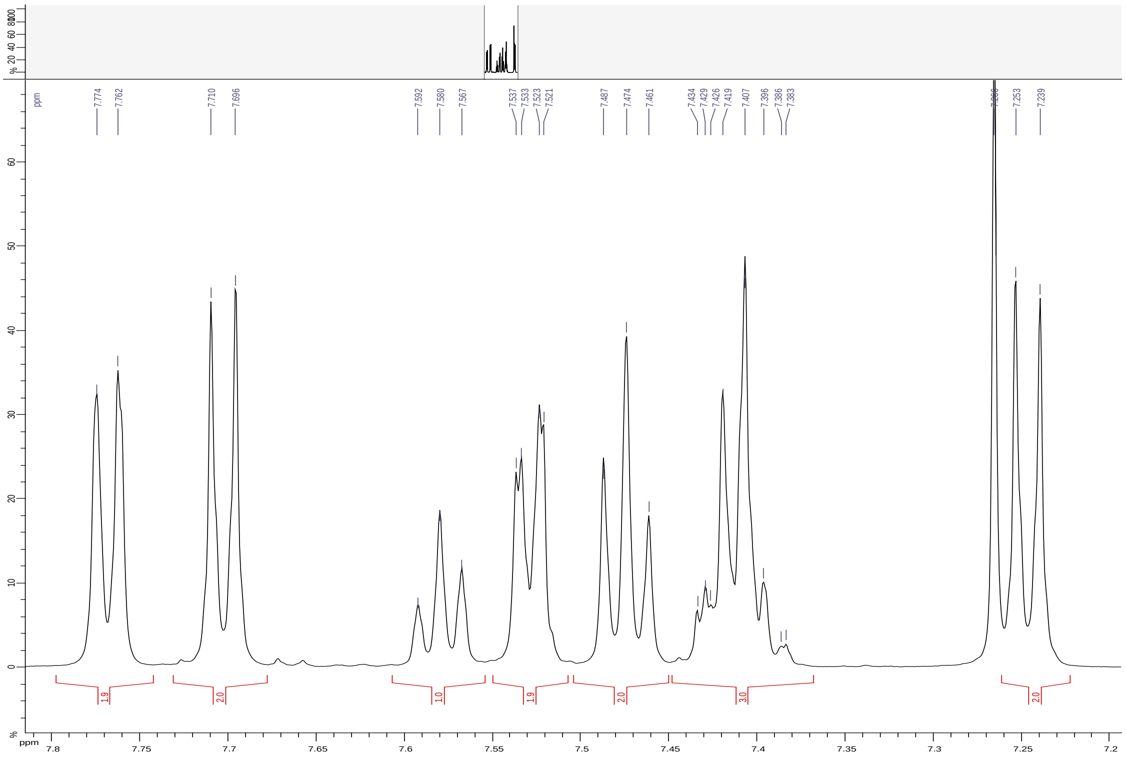

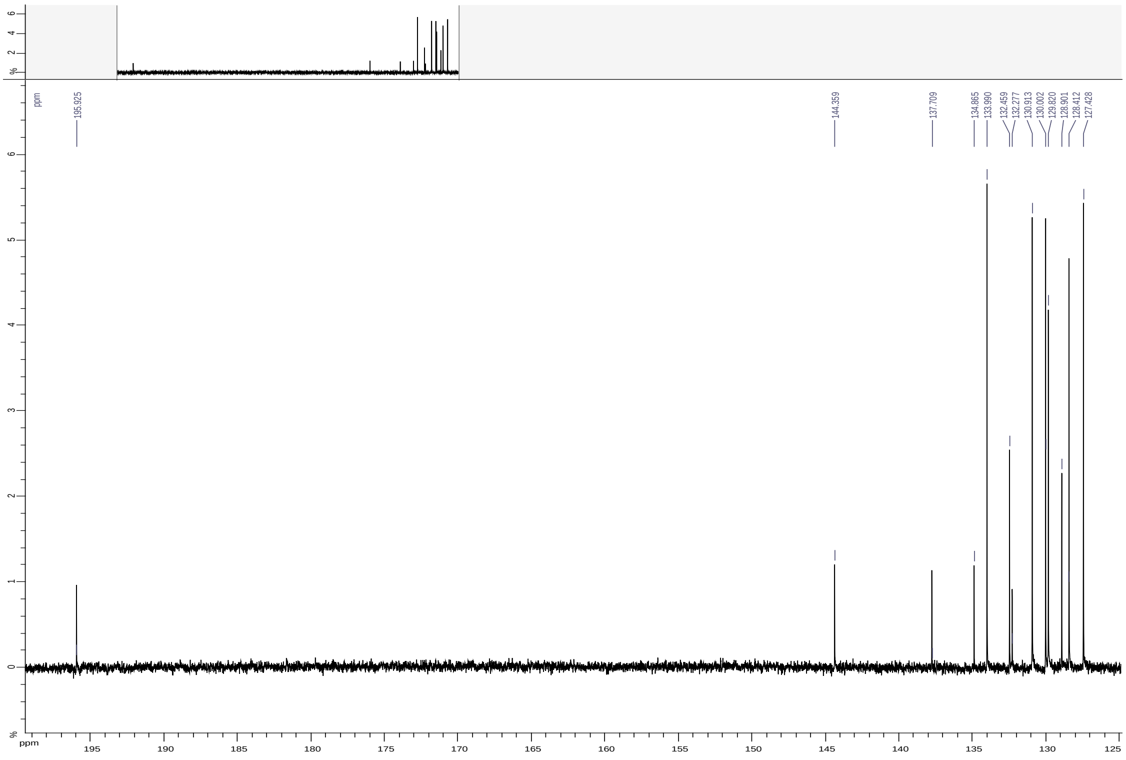

(4-Benzoylphenyl)diphenylsulfonium trifluoromethanesulfonate (2b)

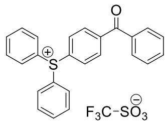

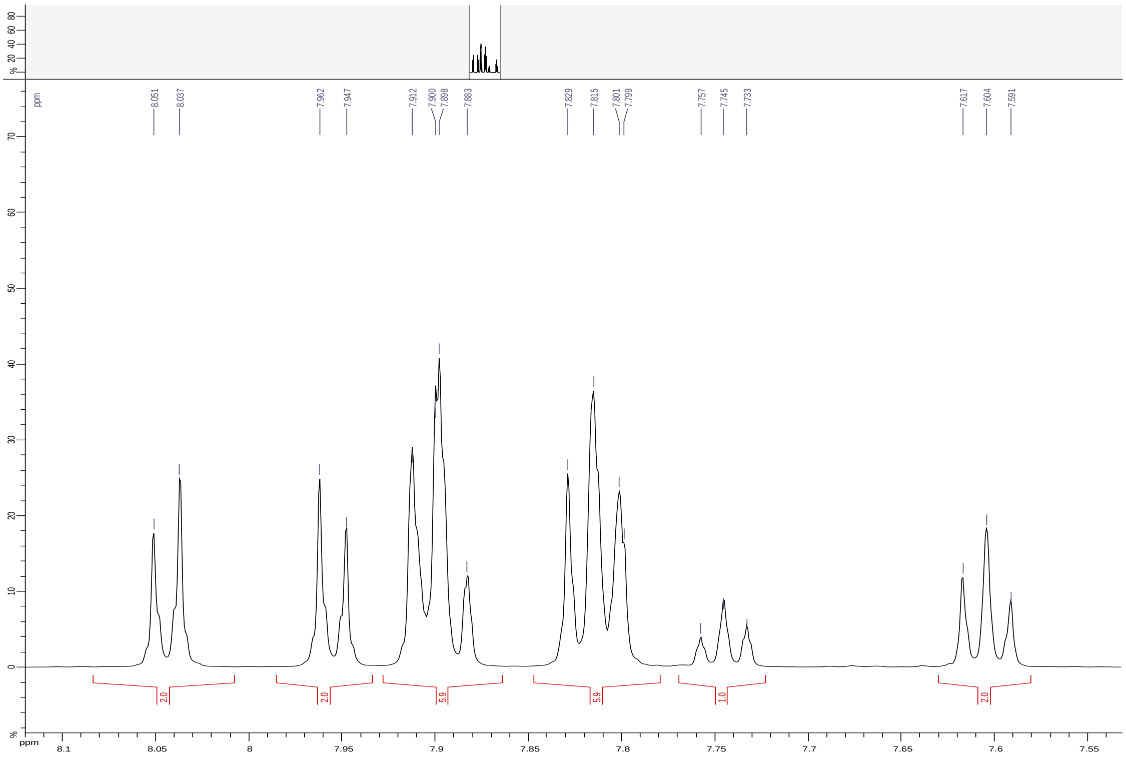

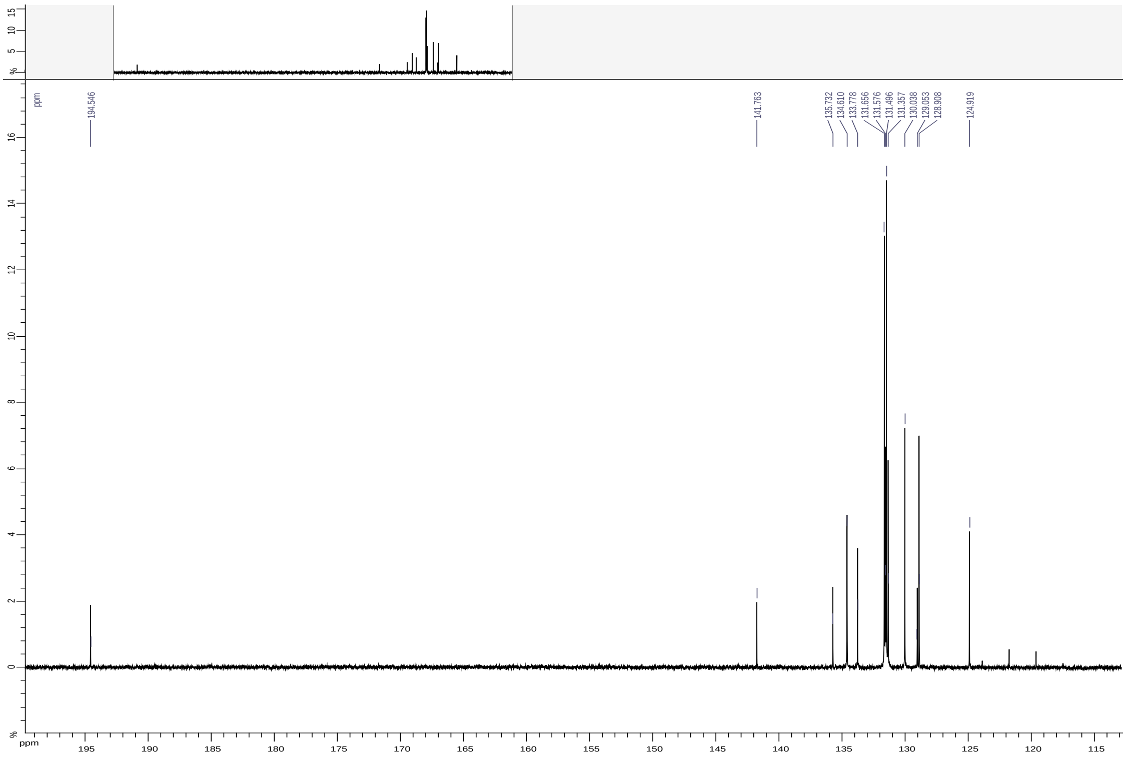

Methyl 2-((4-methoxyphenyl)thio)benzoate (5a)

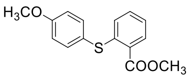

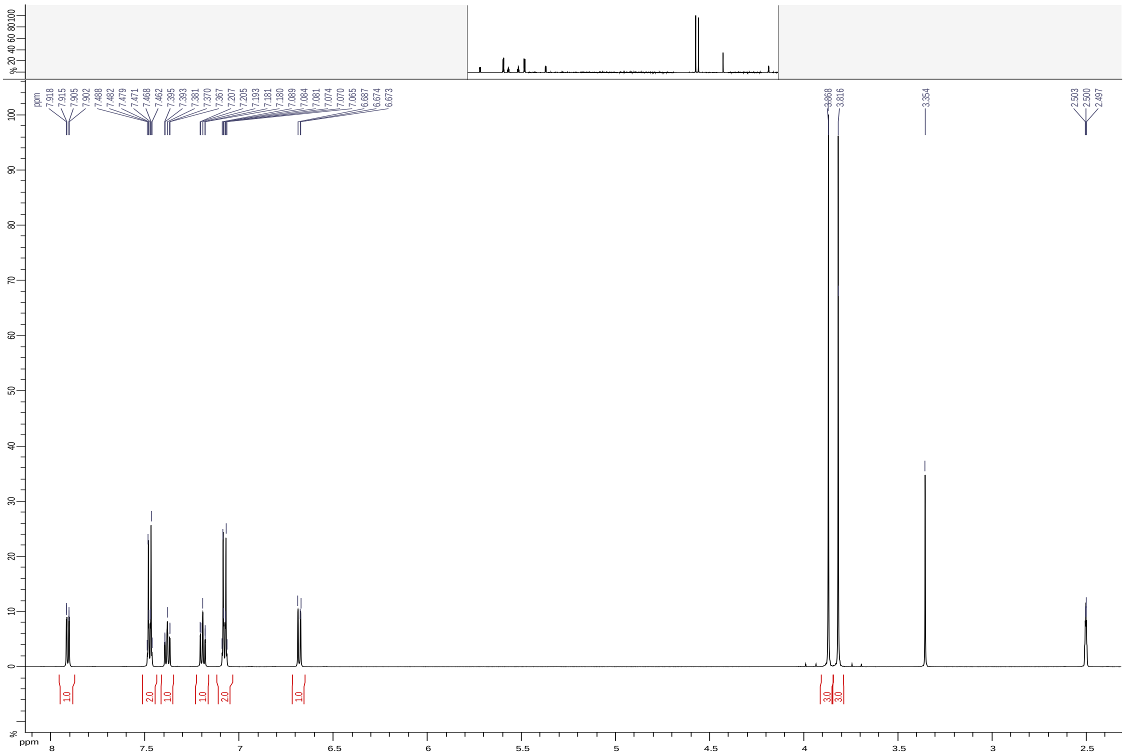

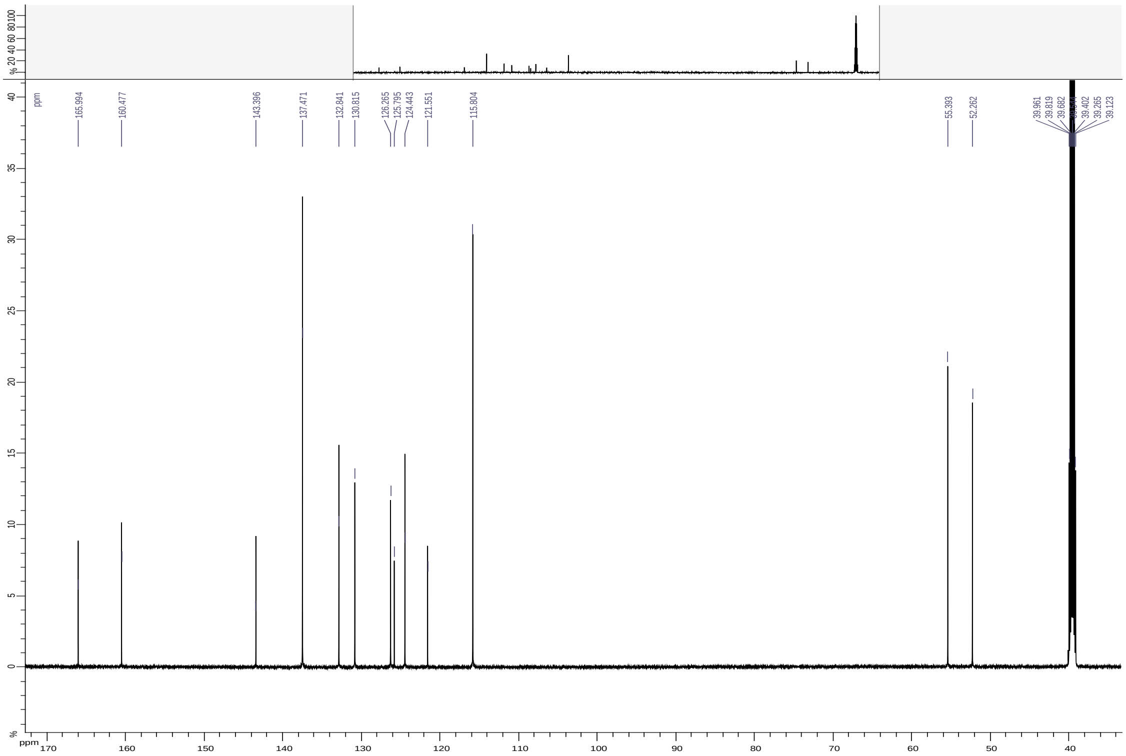

(2-(Methoxycarbonyl)phenyl)(4-methoxyphenyl)(phenyl)sulfonium trifluoromethane-sulfonate (5b)

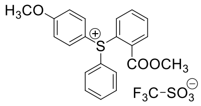

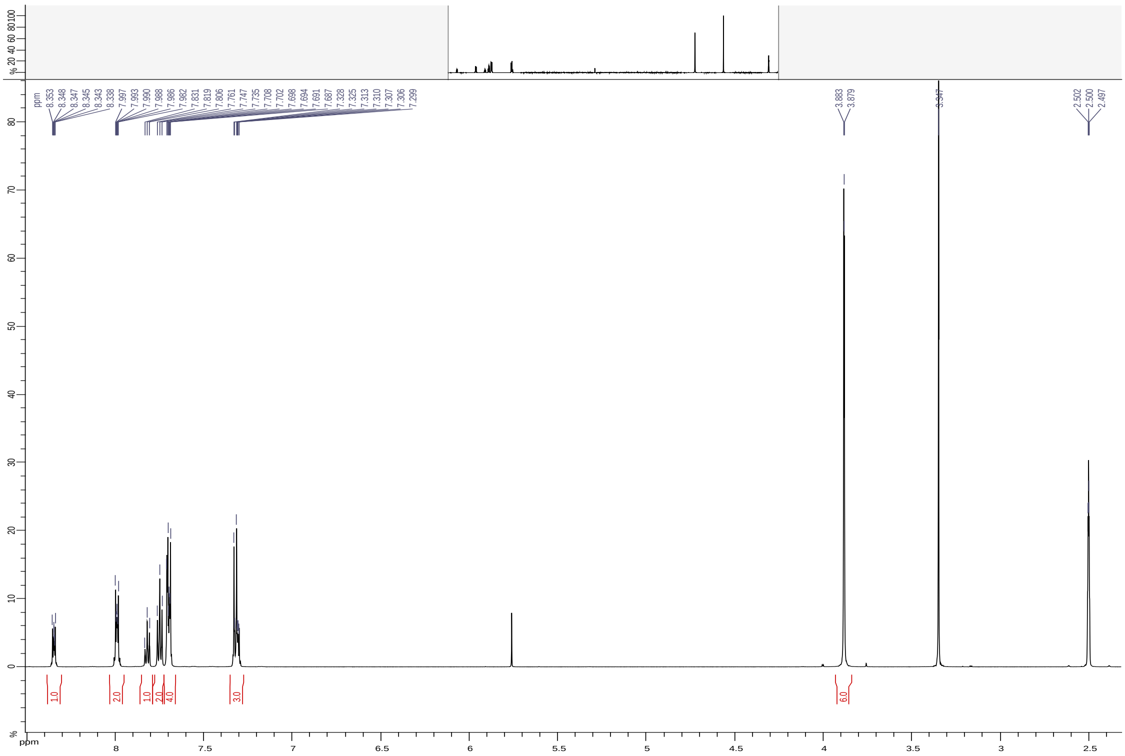

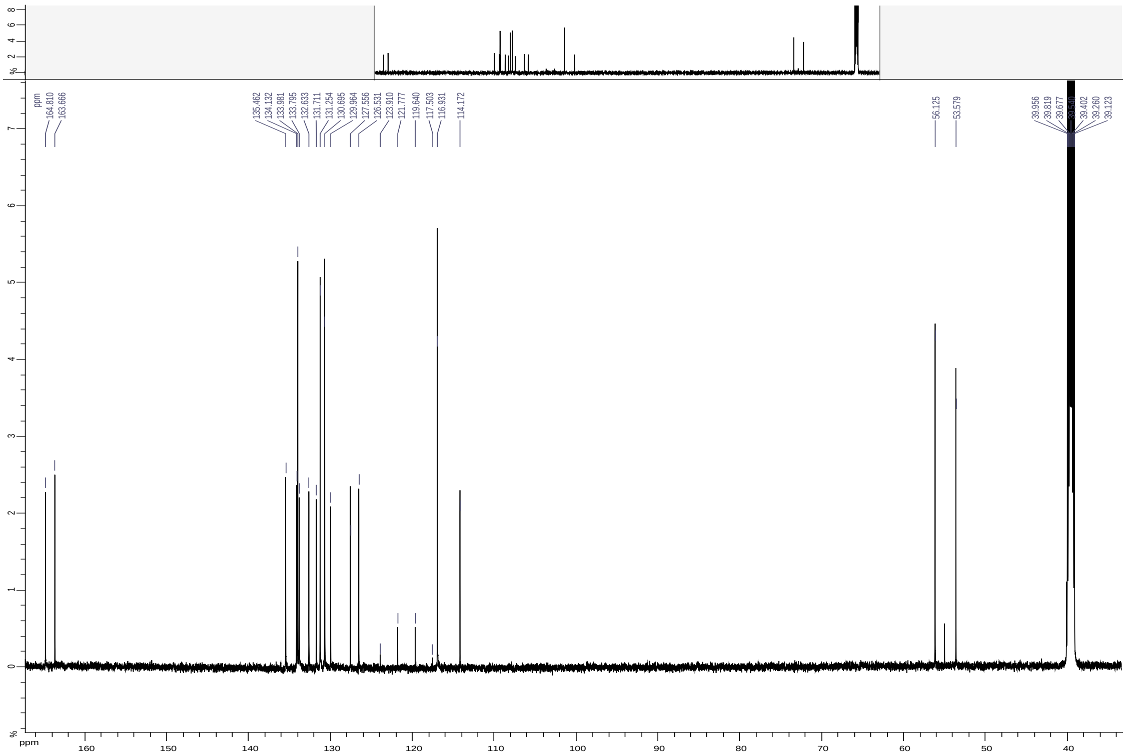

4,4'-Dimethoxy diphenyl sulfide (6a)

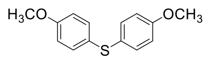

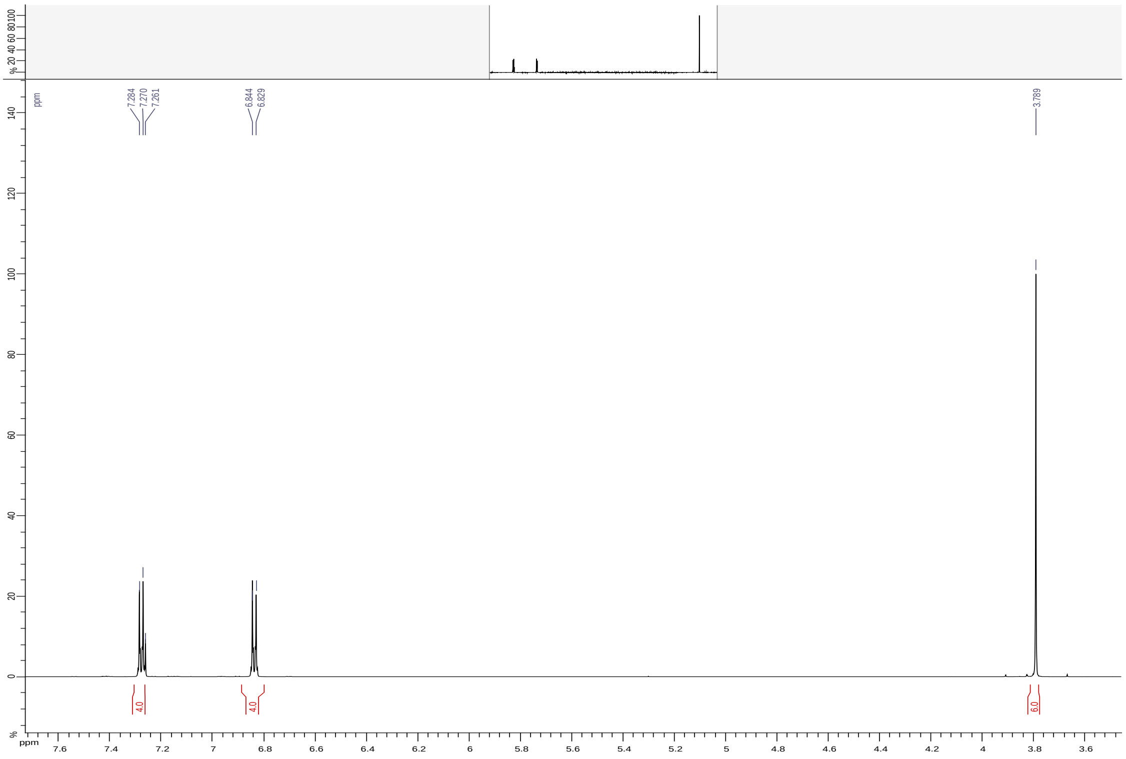

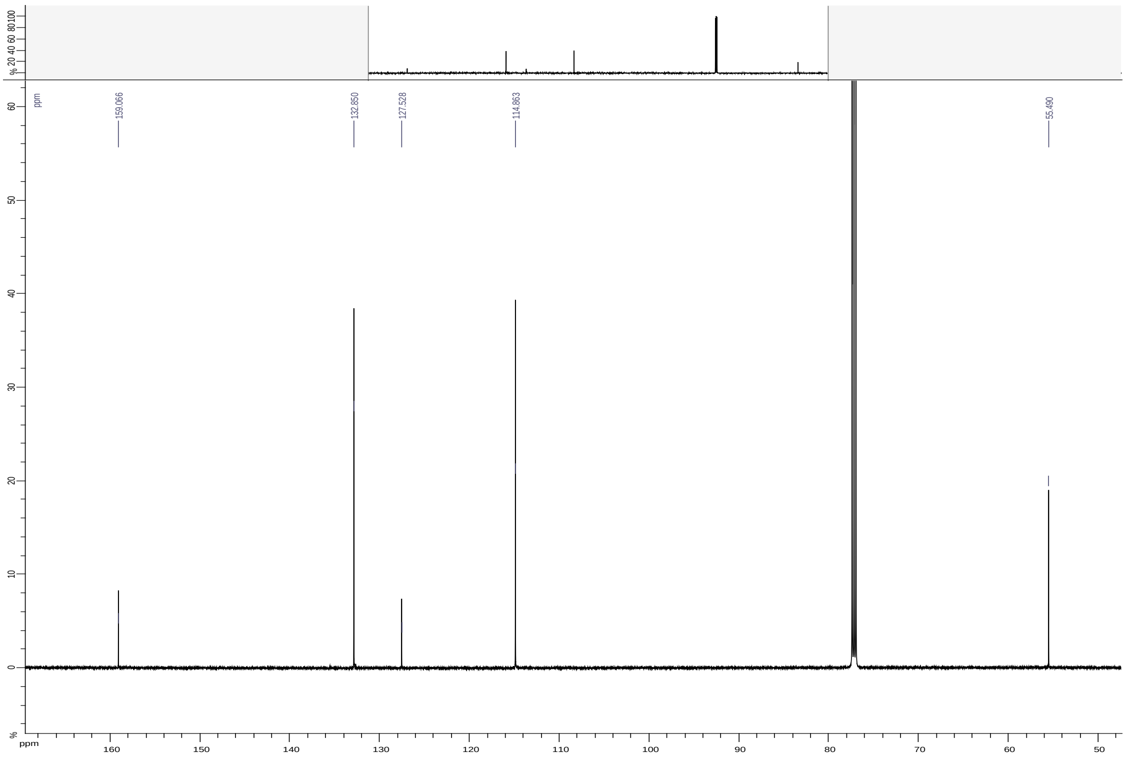

Bis(4-methoxyphenyl)(phenyl)sulfonium trifluoromethanesulfonate (6b)

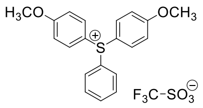

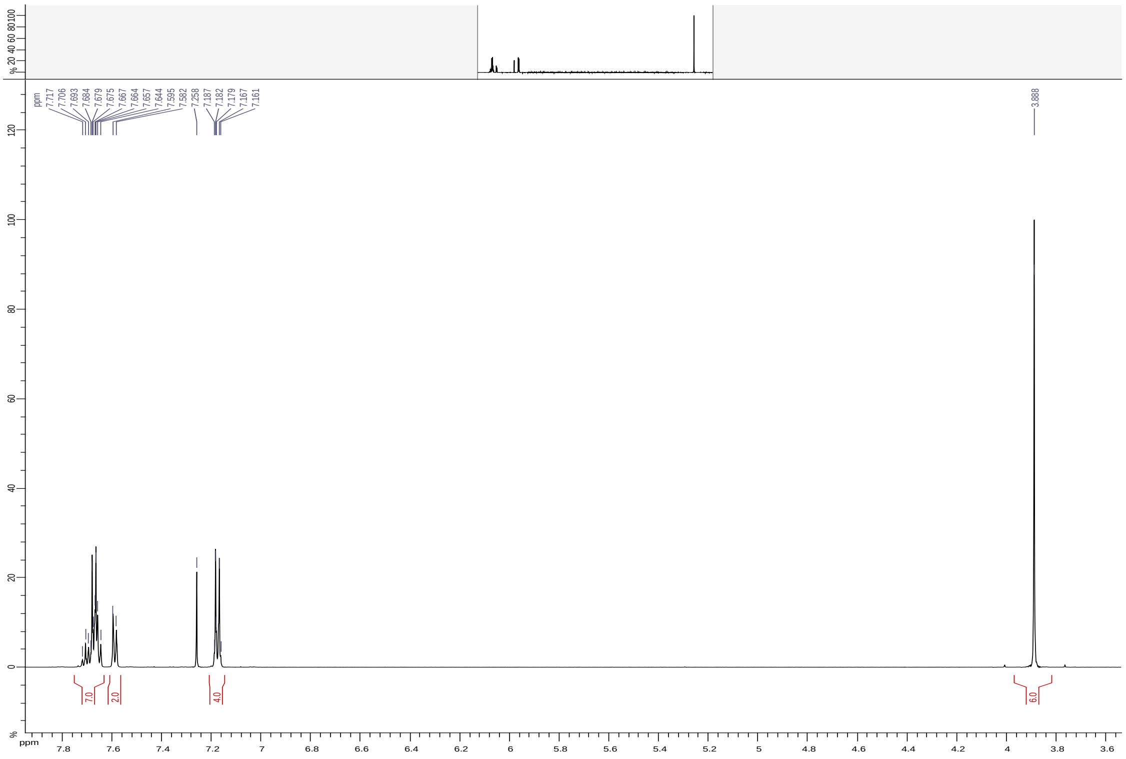

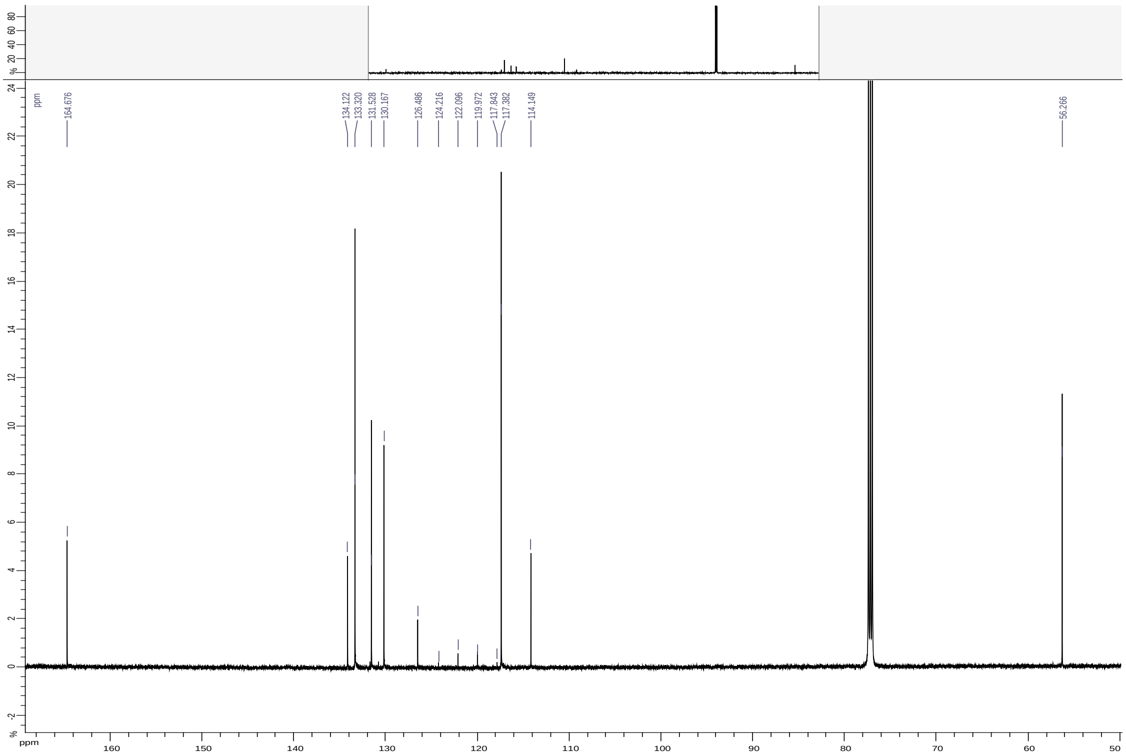

(2-Methoxyphenyl)(4-methoxyphenyl)sulfane (7a)

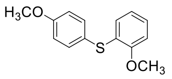

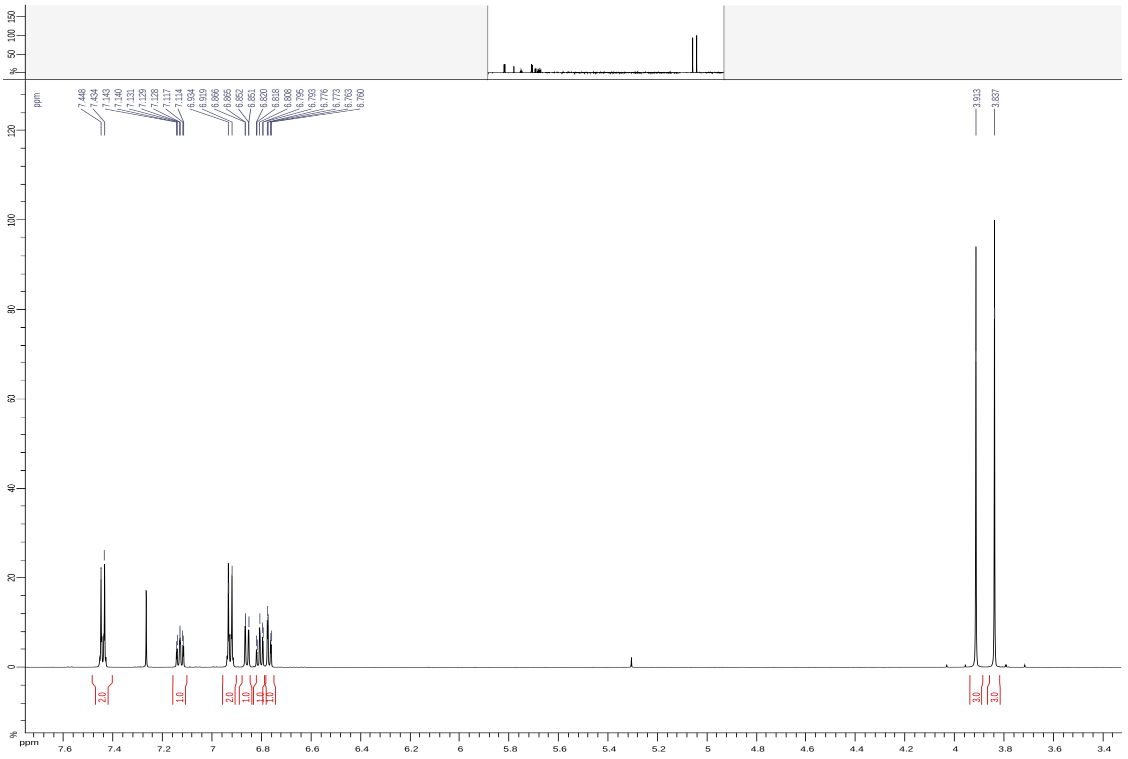

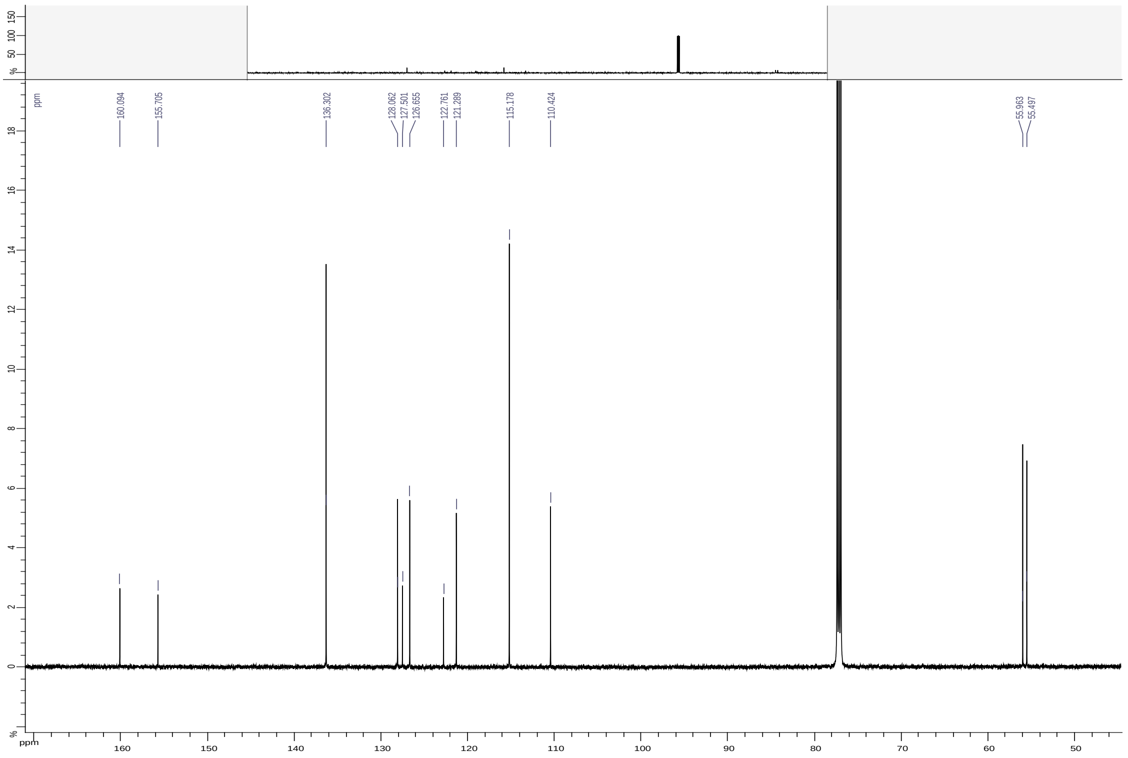

(2-Methoxyphenyl)(4-methoxyphenyl)(phenyl)sulfonium trifluoromethanesulfonate (7b)

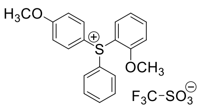

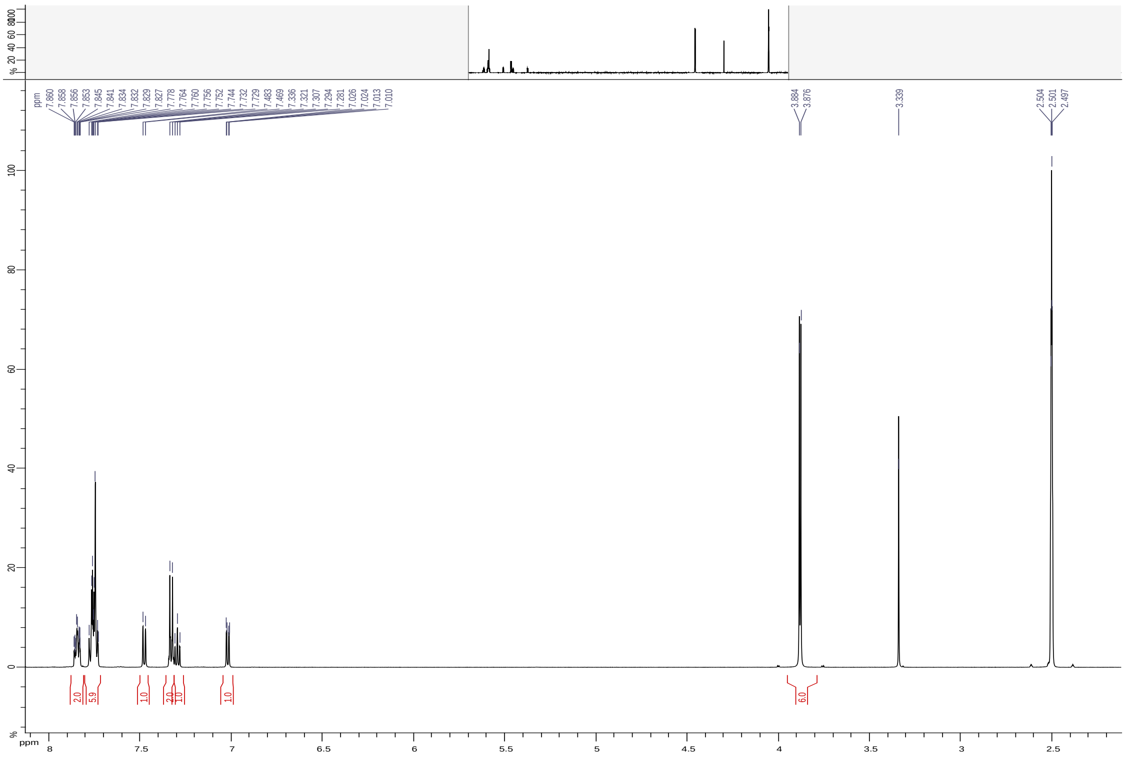

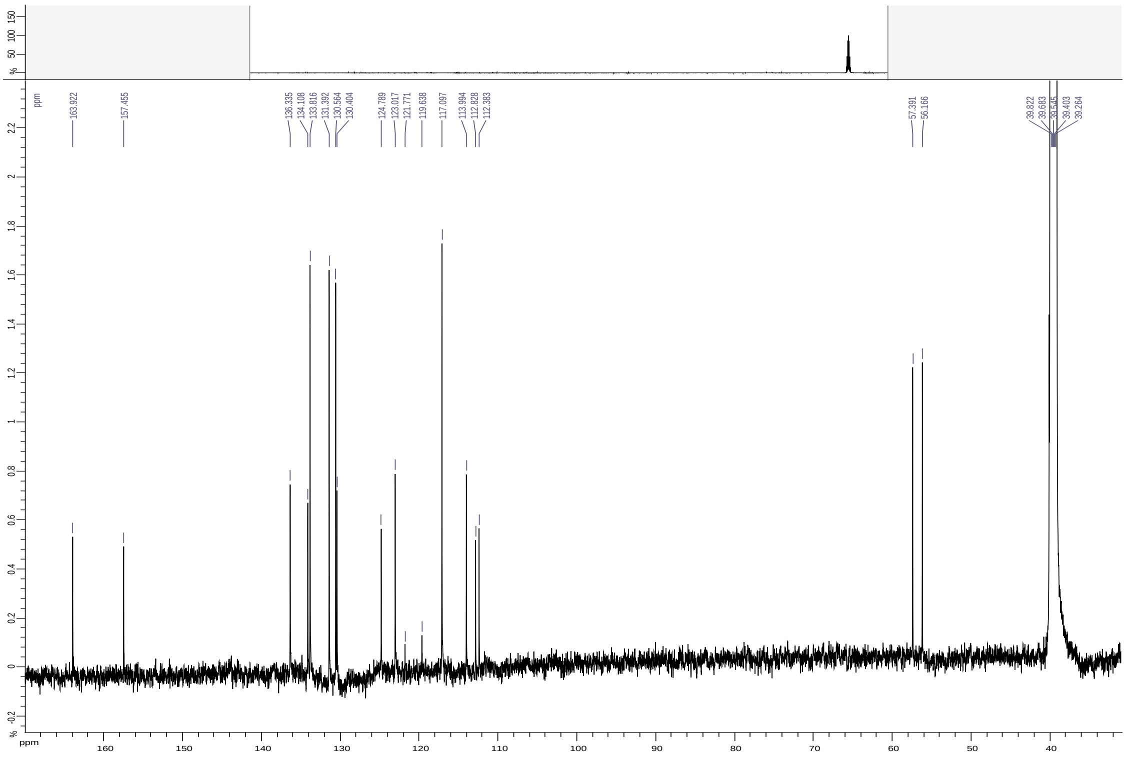

(4-Bromophenyl)(4-methoxyphenyl)sulfane (F)

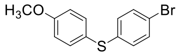

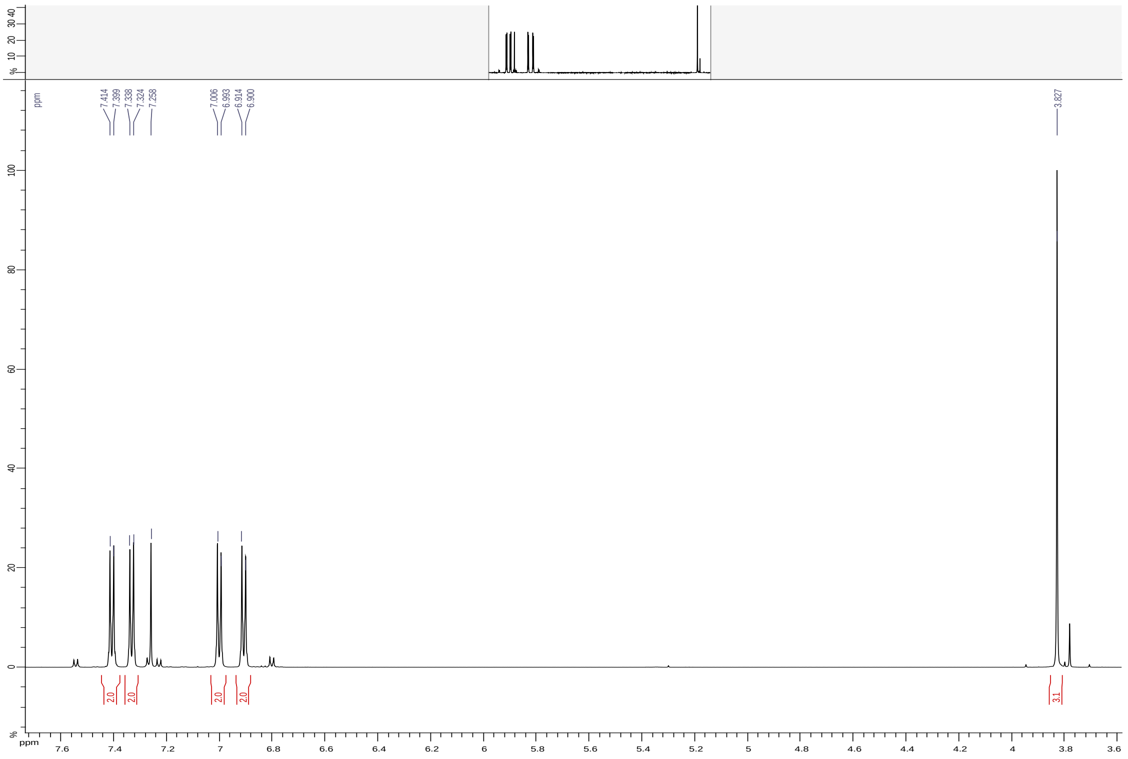

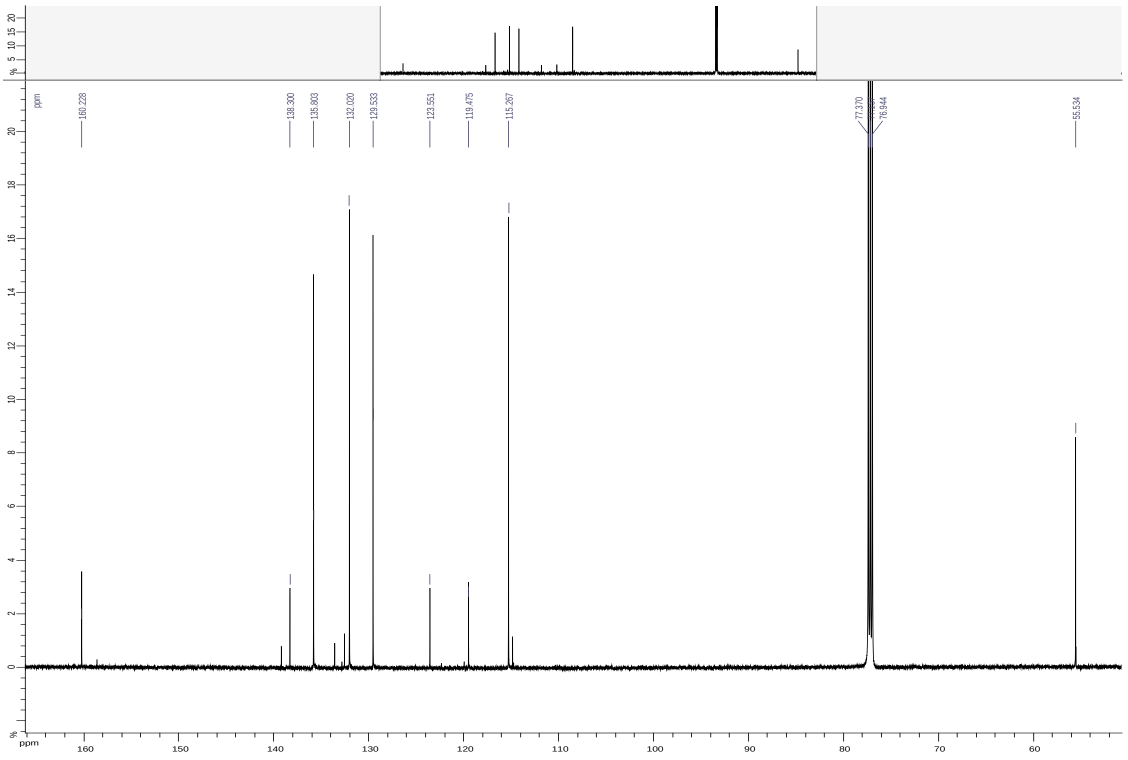

tert-Butyl 4-(4-((4-methoxyphenyl)thio)benzoyl)piperidine-1-carboxylate (G)

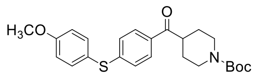

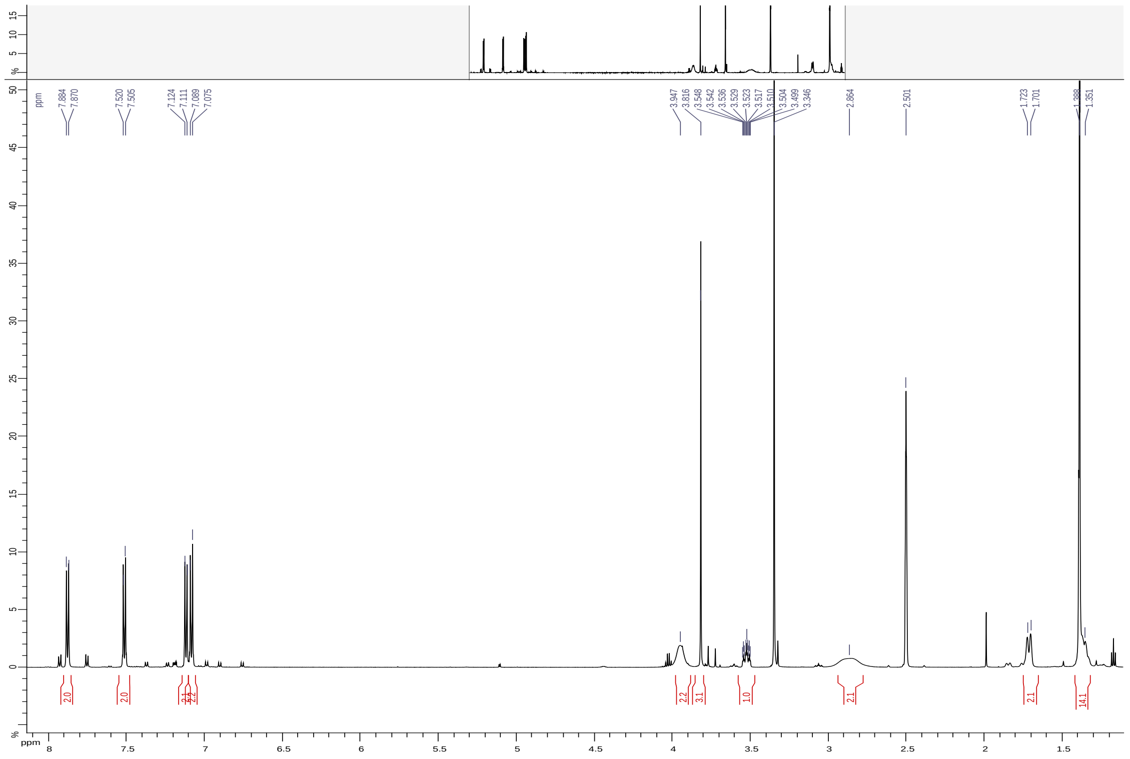

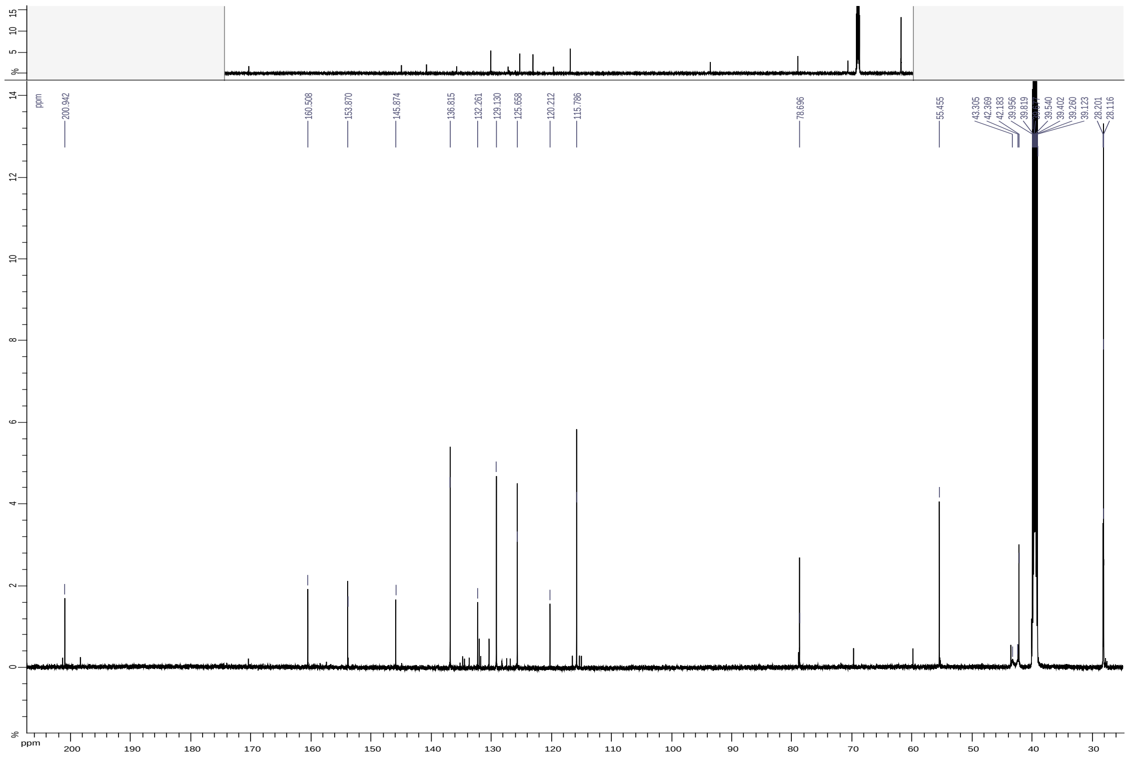

***tert*-Butyl 4-(hydroxy(4-((4-methoxyphenyl)thio)phenyl)(phenyl)methyl)piperidine-1-carboxylate (H)**

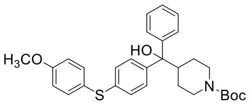

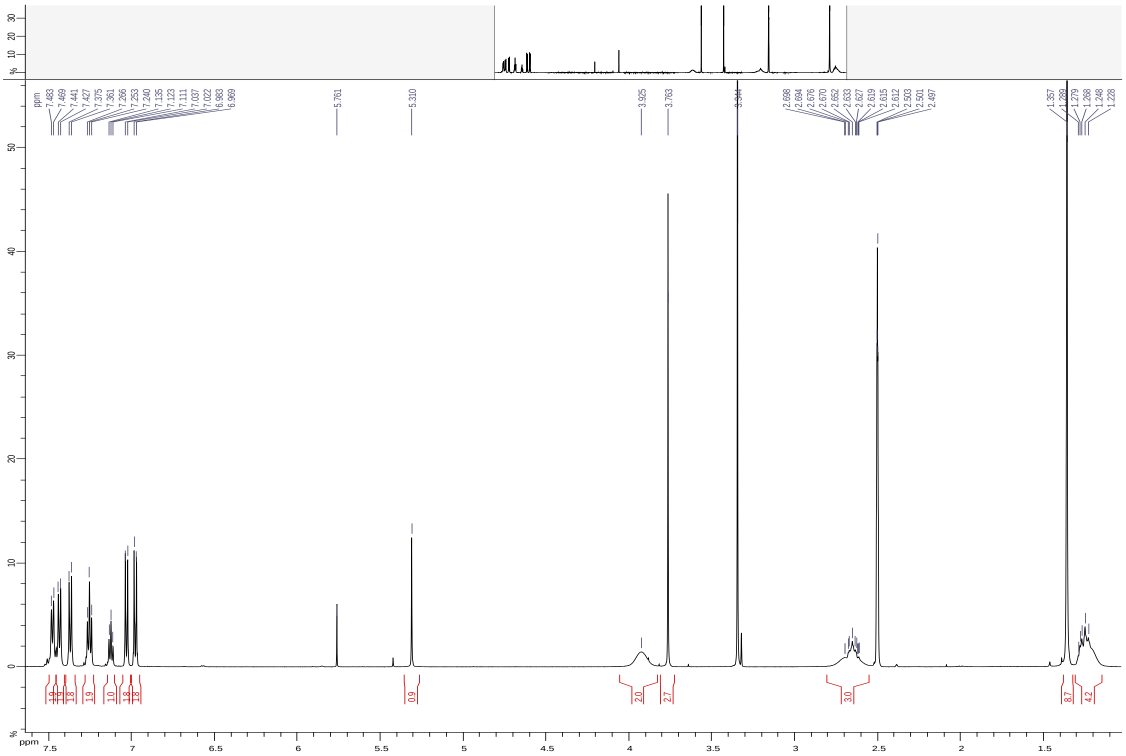

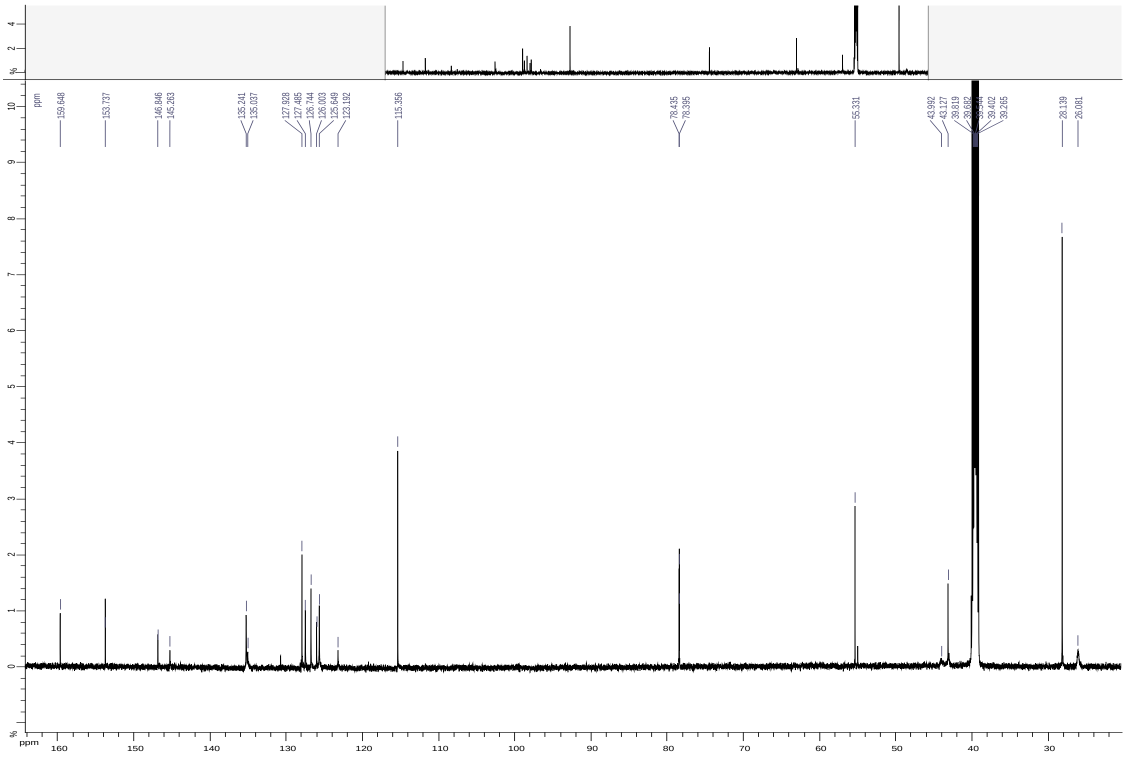

4-((4-((4-Methoxyphenyl)thio)phenyl)(phenyl)methylene)piperidine (I)

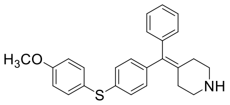

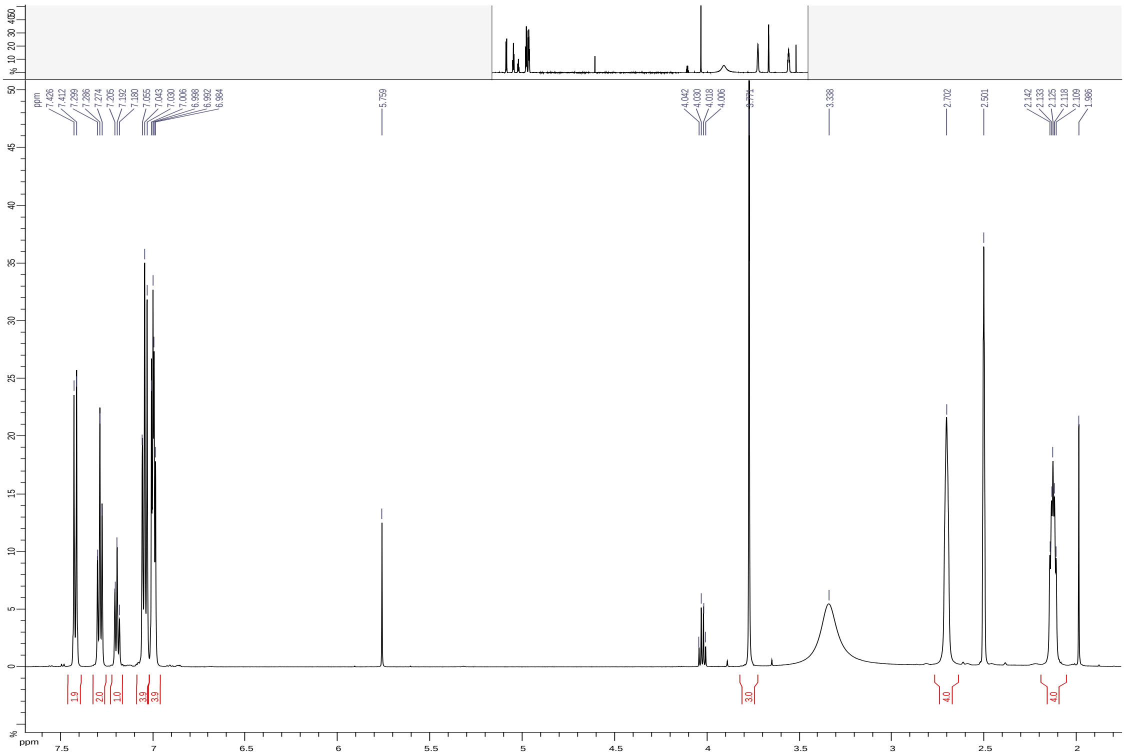

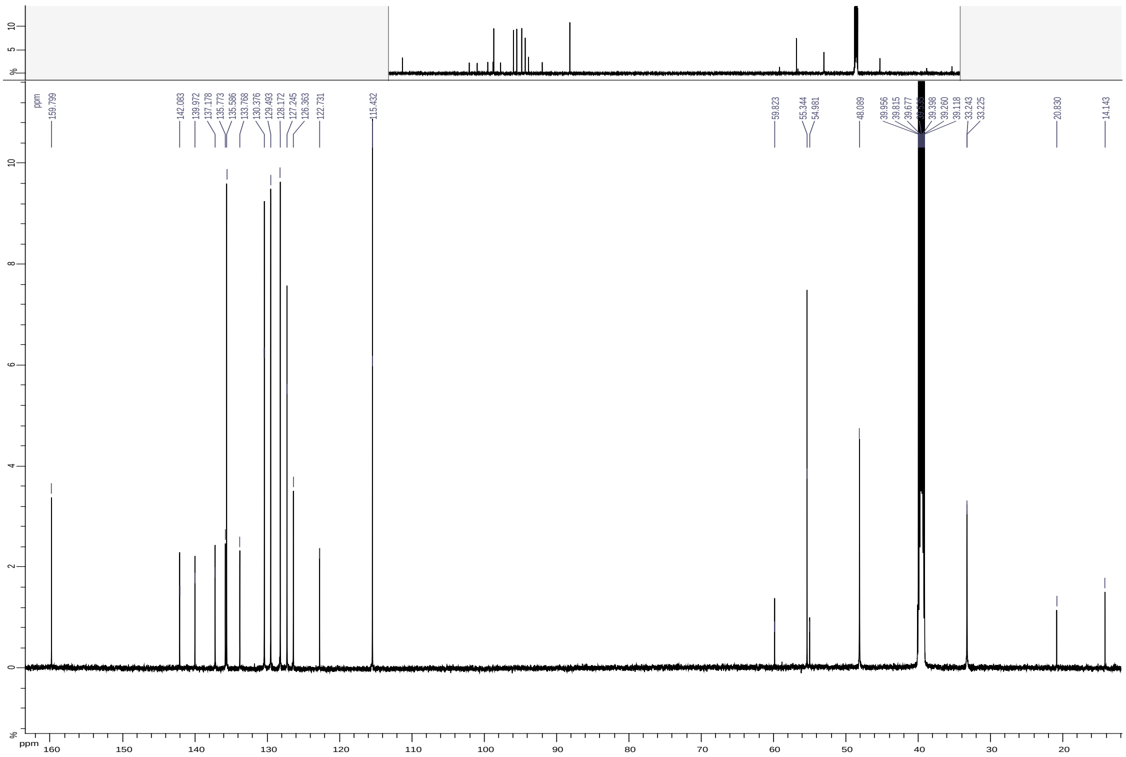

Ethyl 4-(4-((4-((4-methoxyphenyl)thio)phenyl)(phenyl)methylene)piperidin-1-yl)butanoate (9a)

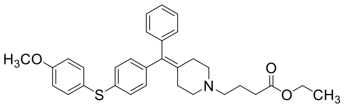

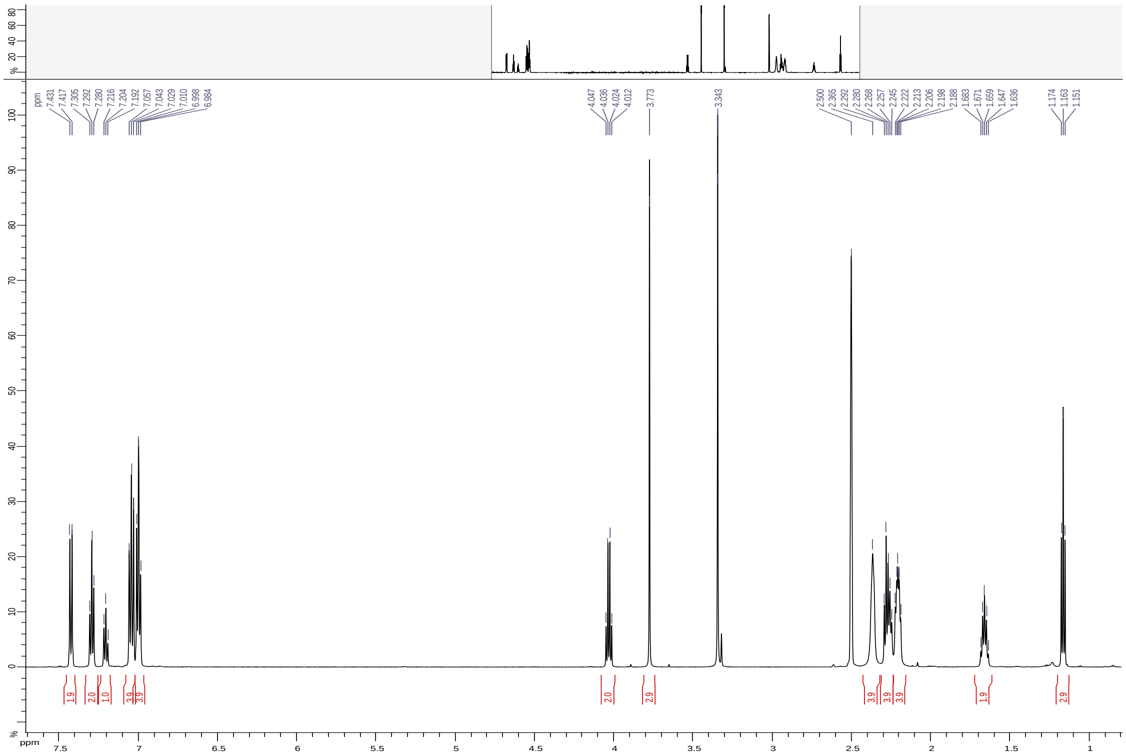

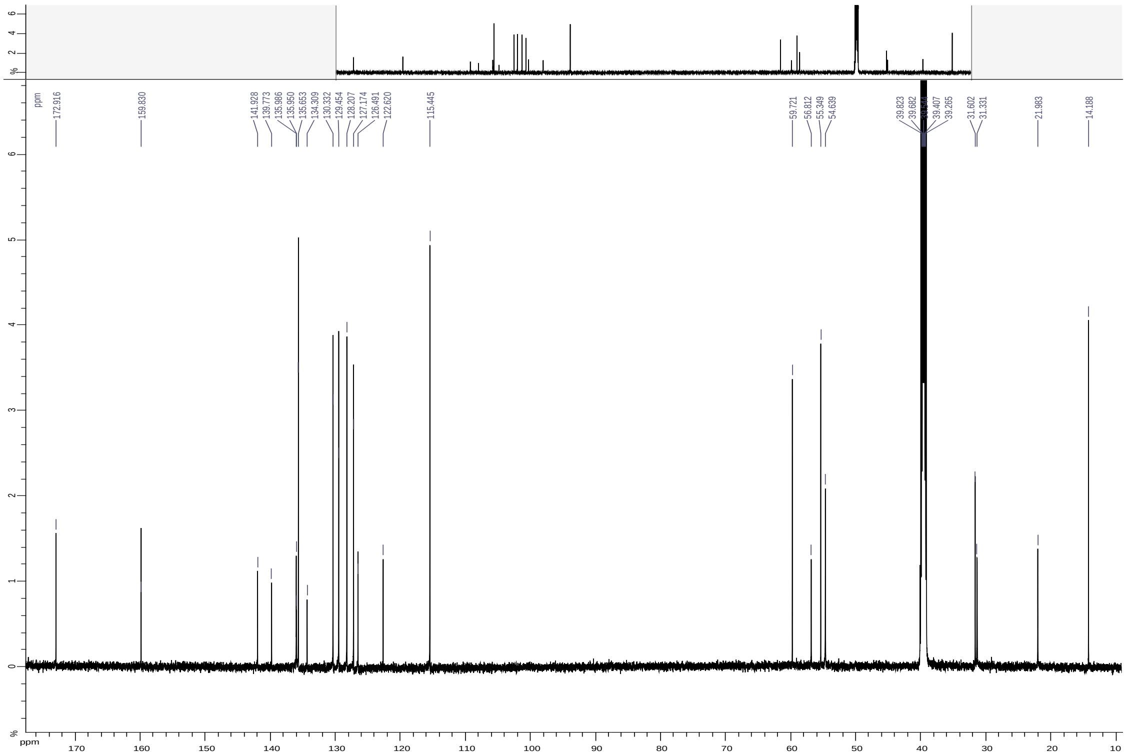

Bis(4-methoxyphenyl)iodonium trifluoromethanesulfonate (J)

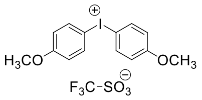

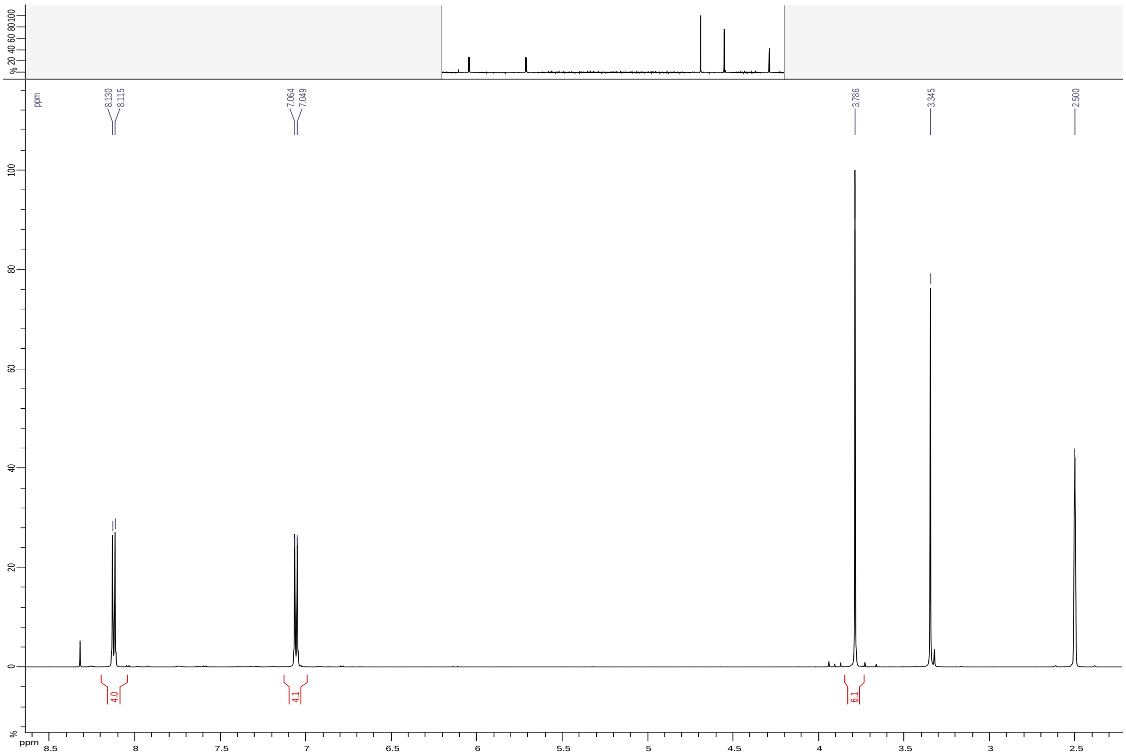

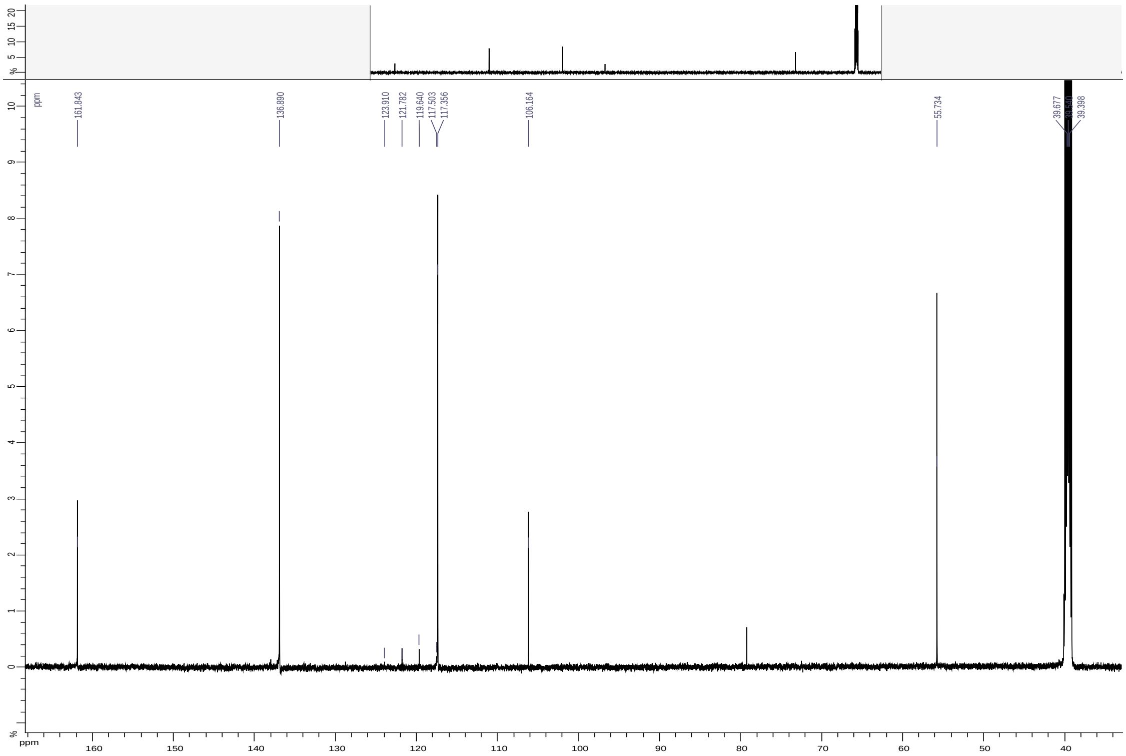

(4-((1-(4-Ethoxy-4-oxobutyl)piperidin-4-ylidene)(phenyl)methyl)phenyl)bis(4-methoxyphenyl)sulfonium trifluoromethanesulfonate (9b)

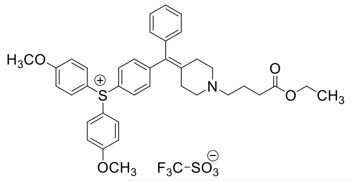

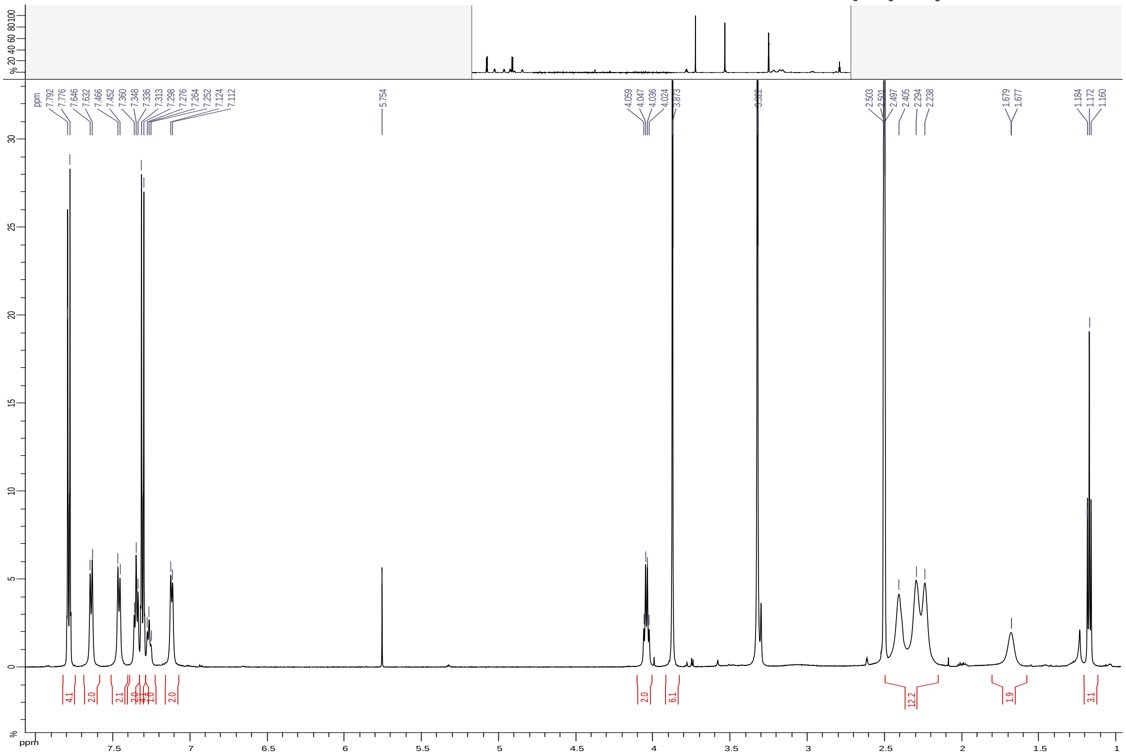

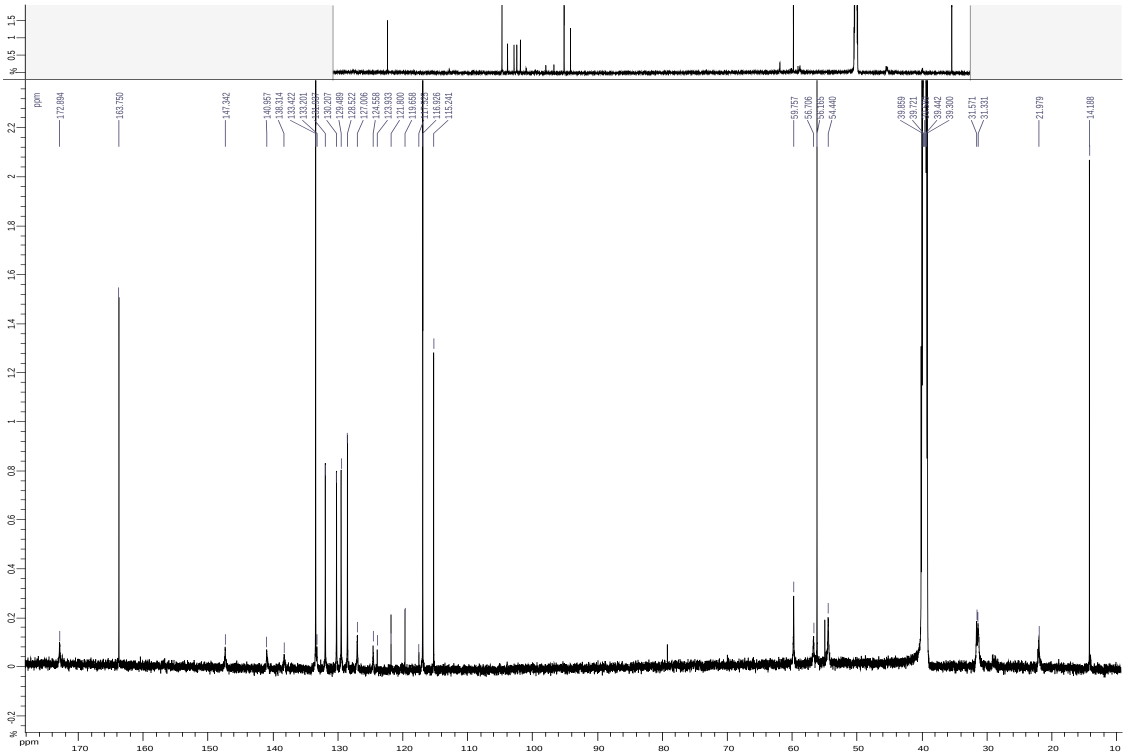

4-(4-((4-((4-methoxyphenyl)thio)phenyl)(phenyl)methylene)piperidin-1-yl)butyl acetate (10a)

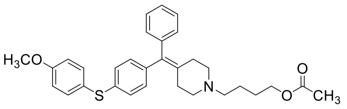

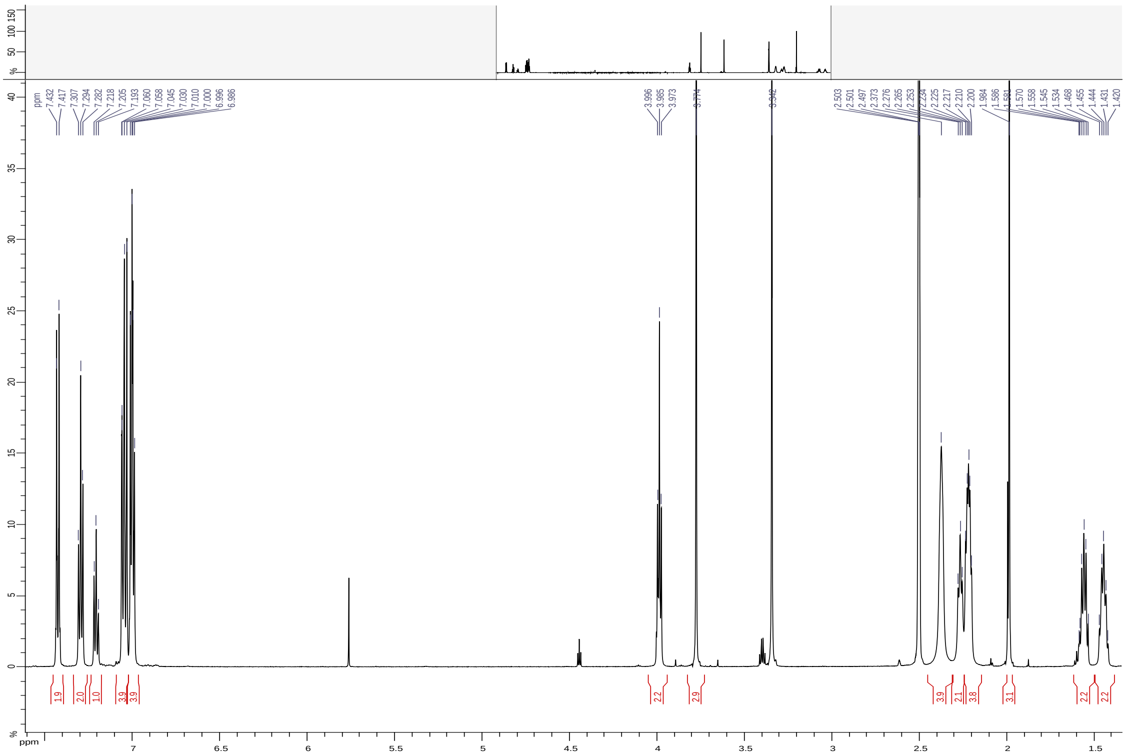

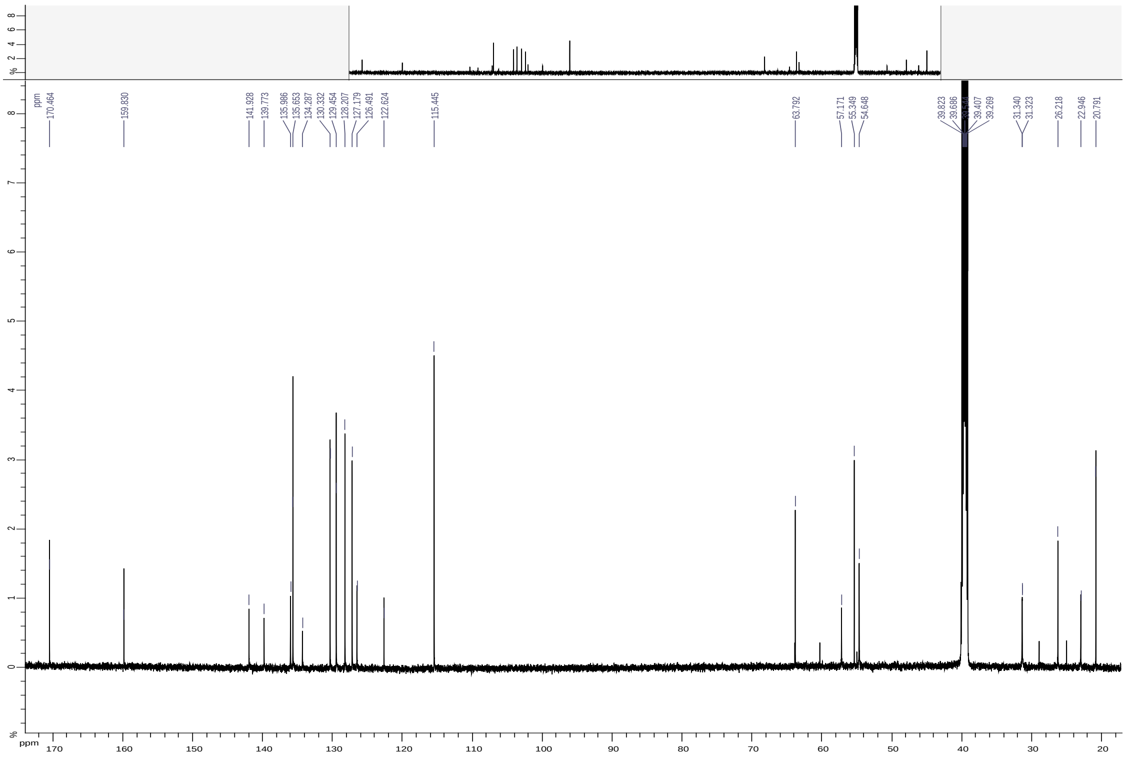

(4-((1-(4-Acetoxybutyl)piperidin-4-ylidene)(phenyl)methyl)phenyl)bis(4-methoxyphenyl)sulfonium trifluoromethanesulfonate (10b)

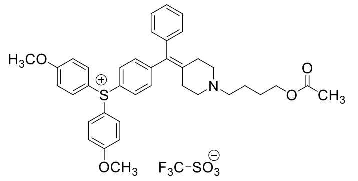

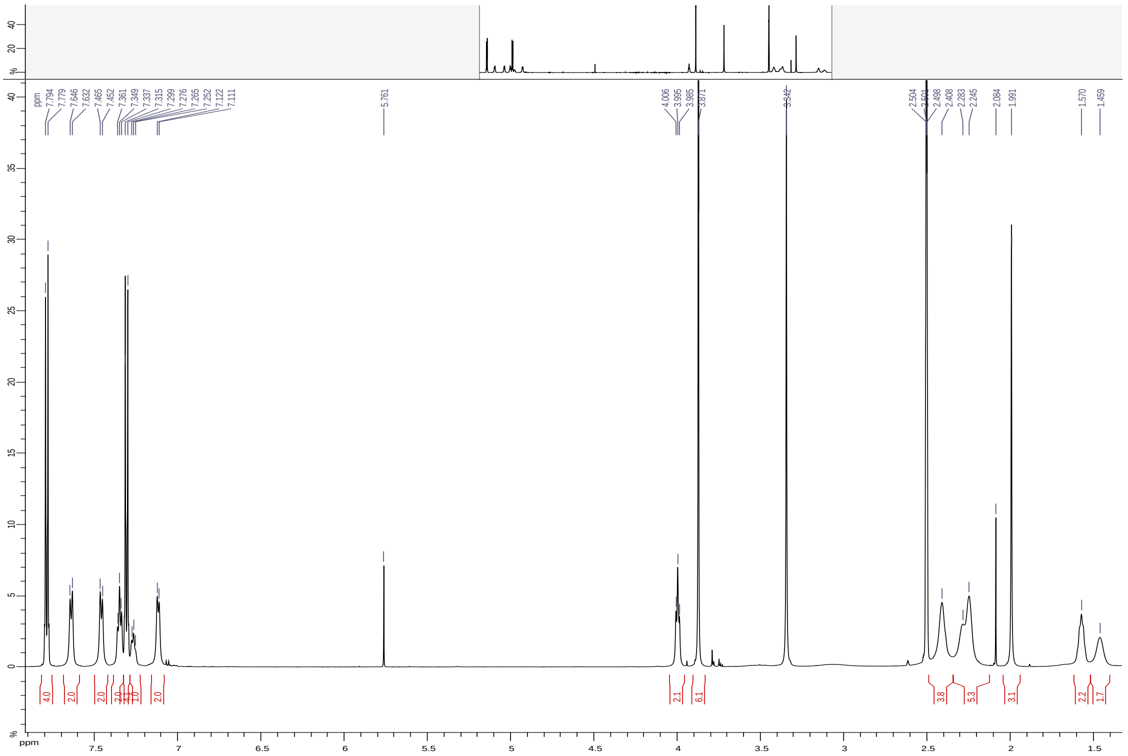

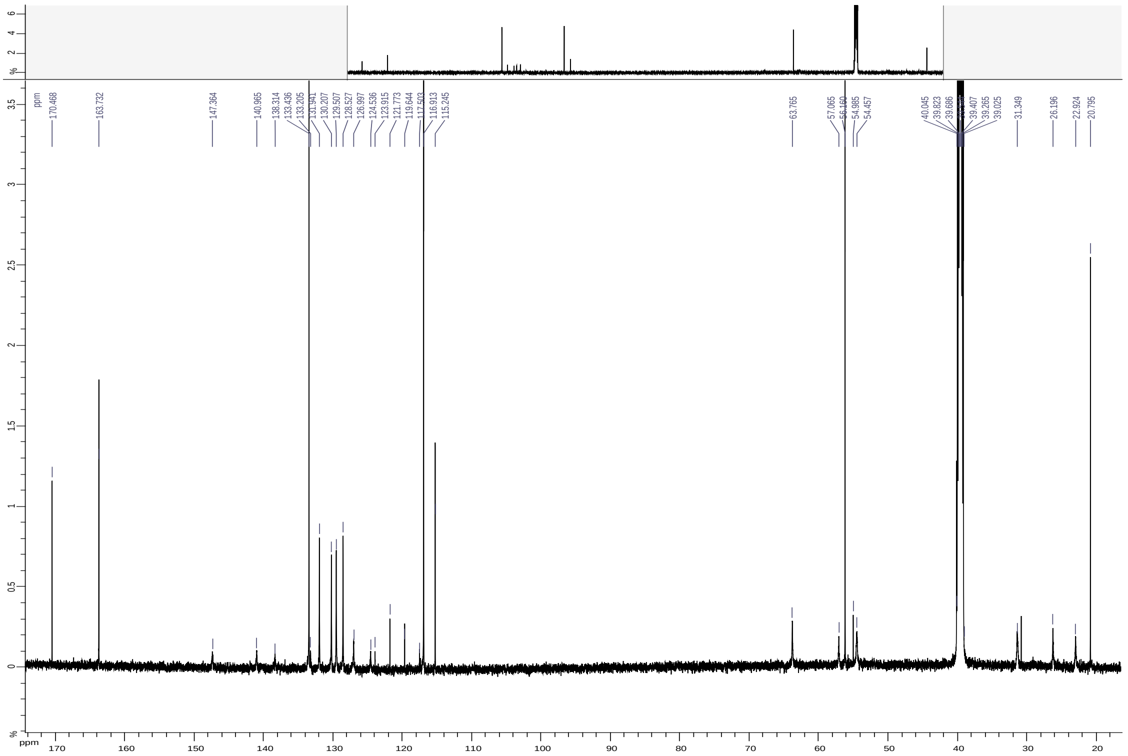

5-Bromo-2-(phenylthio)pyridine (K)

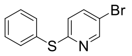

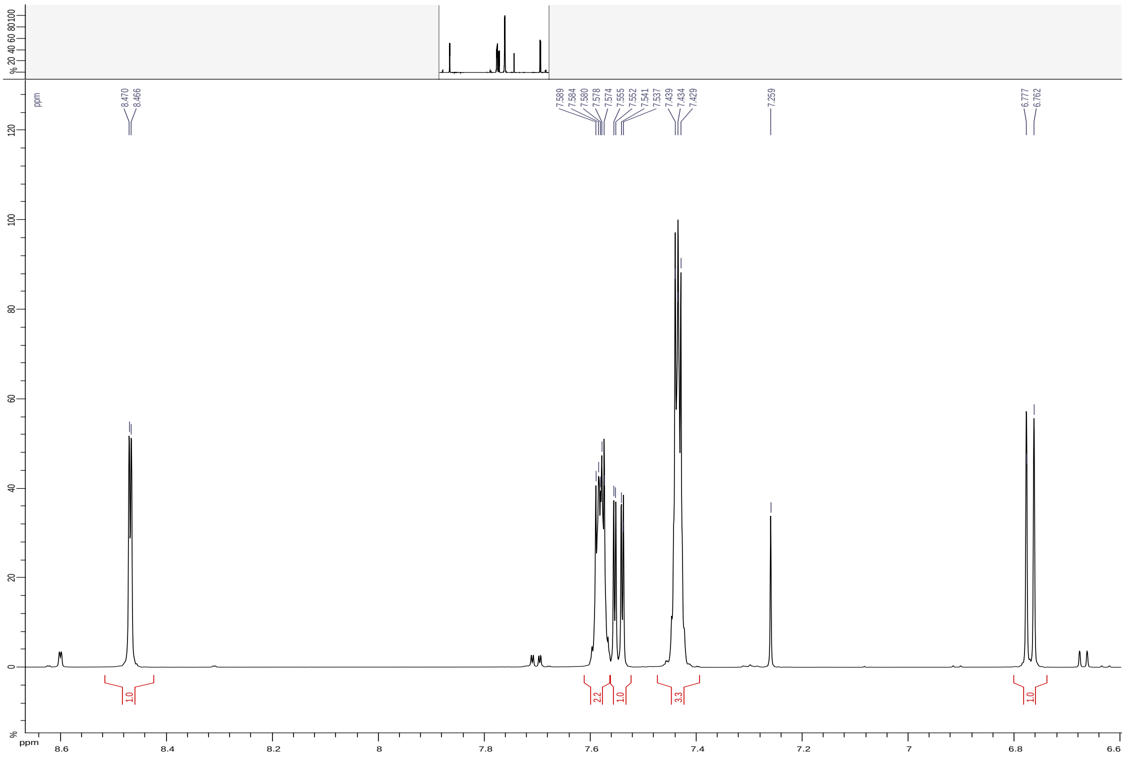

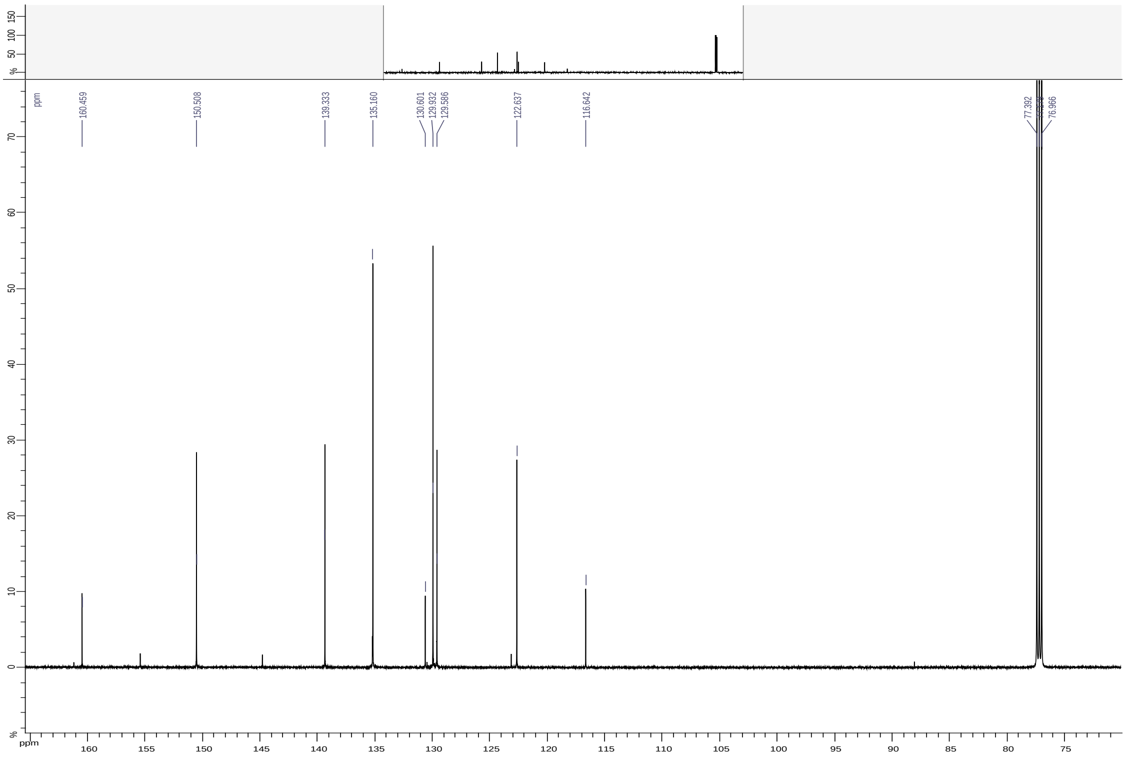

5-Phenyl-2-(phenylthio)pyridine (11a)

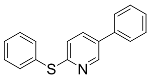

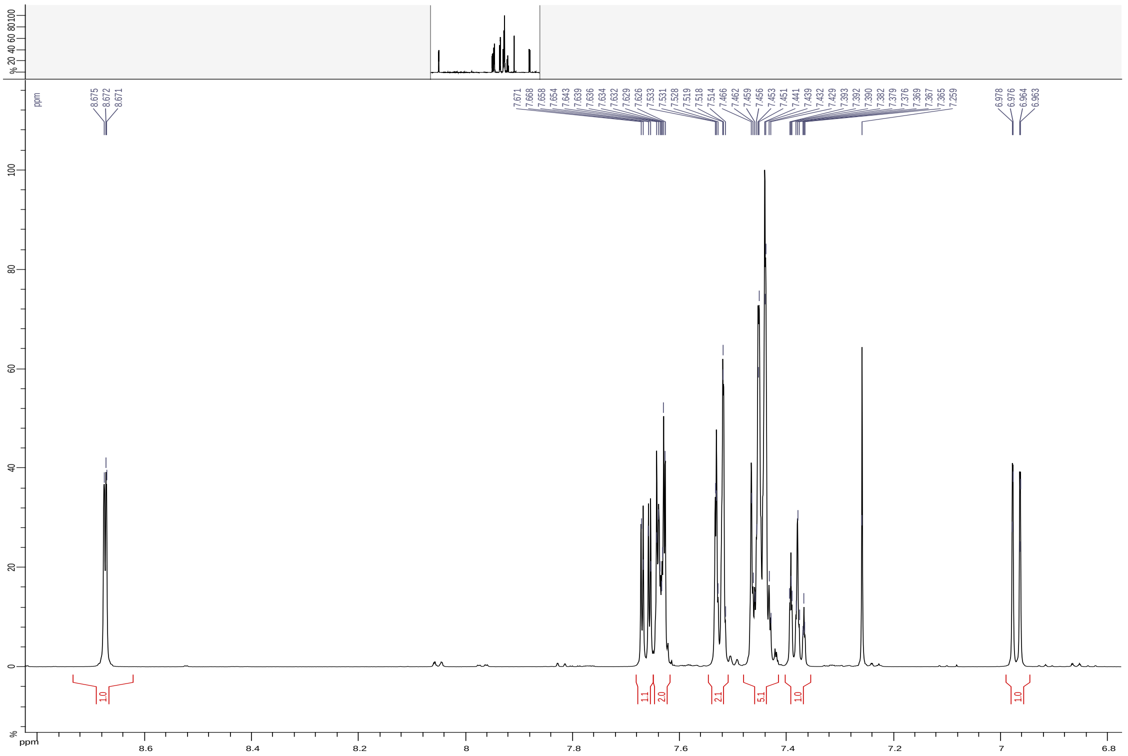

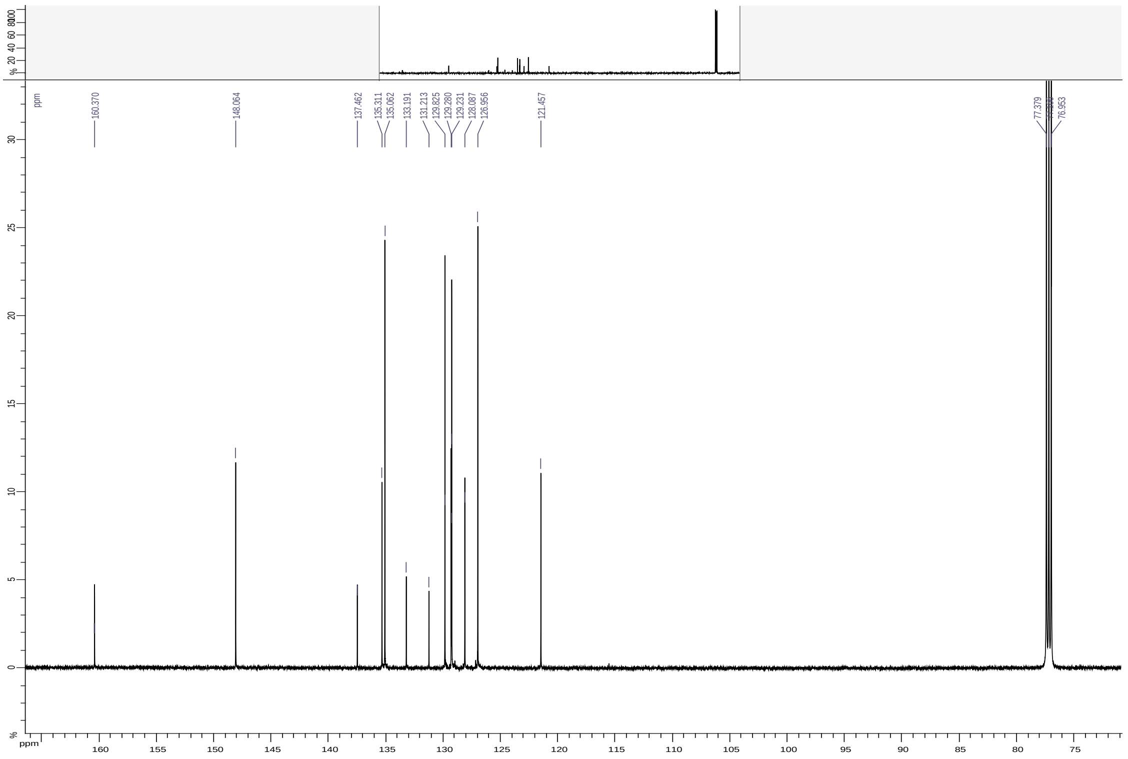

Diphenyl(5-phenylpyridin-2-yl)sulfonium trifluoromethanesulfonate (11b)

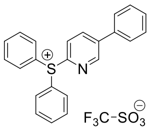

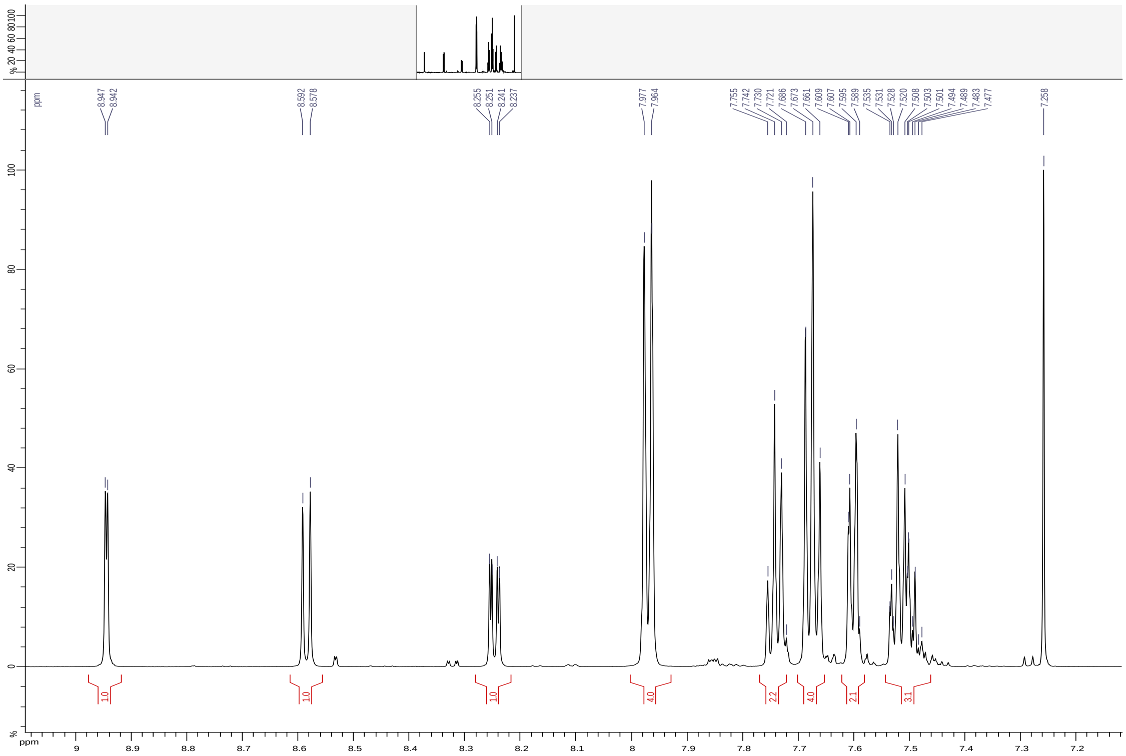

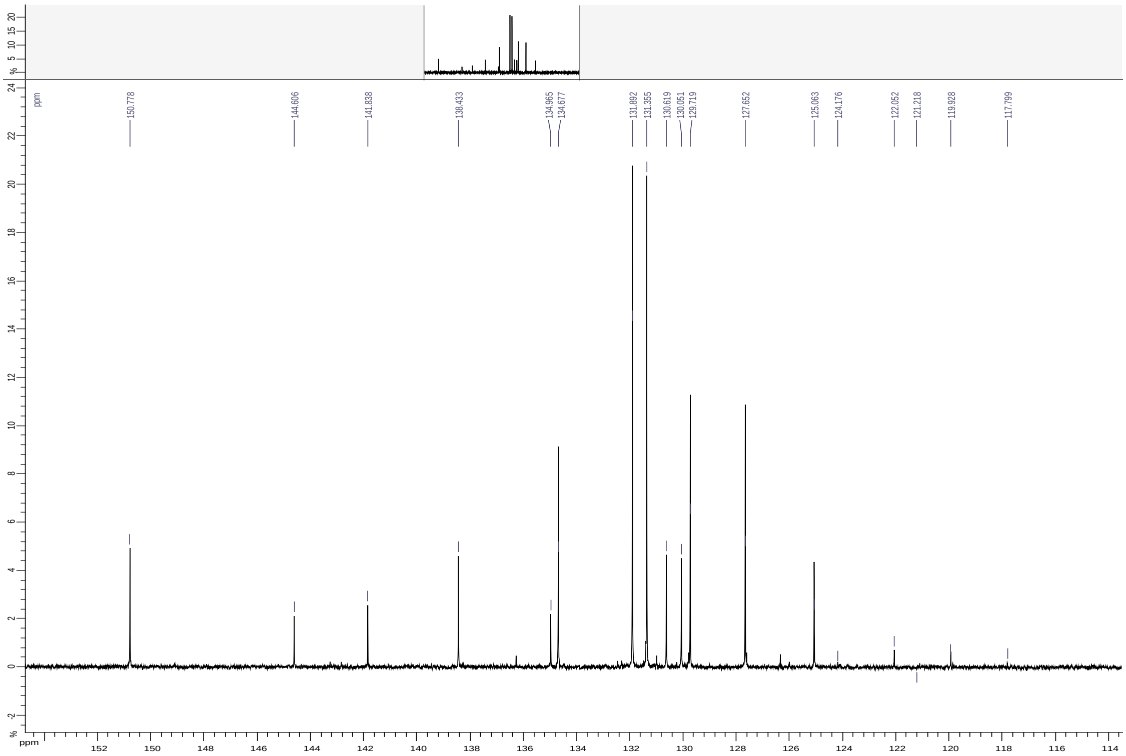

tert-Butyl 2-(((6-bromopyridin-3-yl)oxy)methyl)pyrrolidine-1-carboxylate (L)

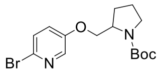

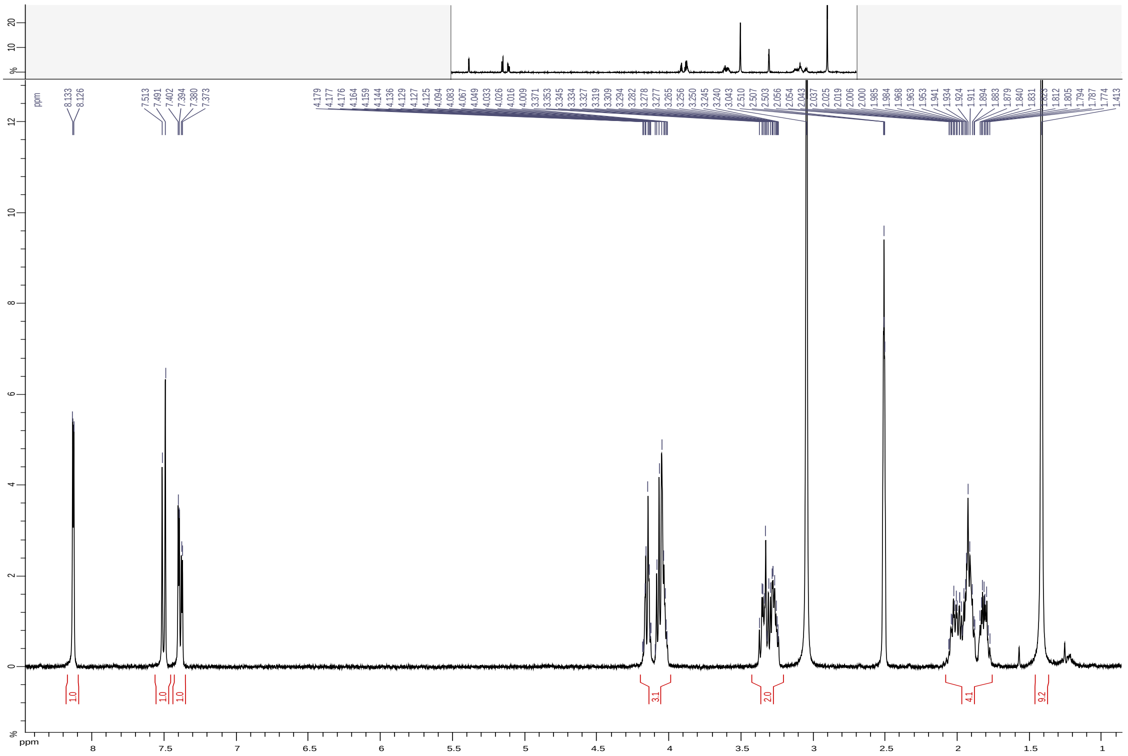

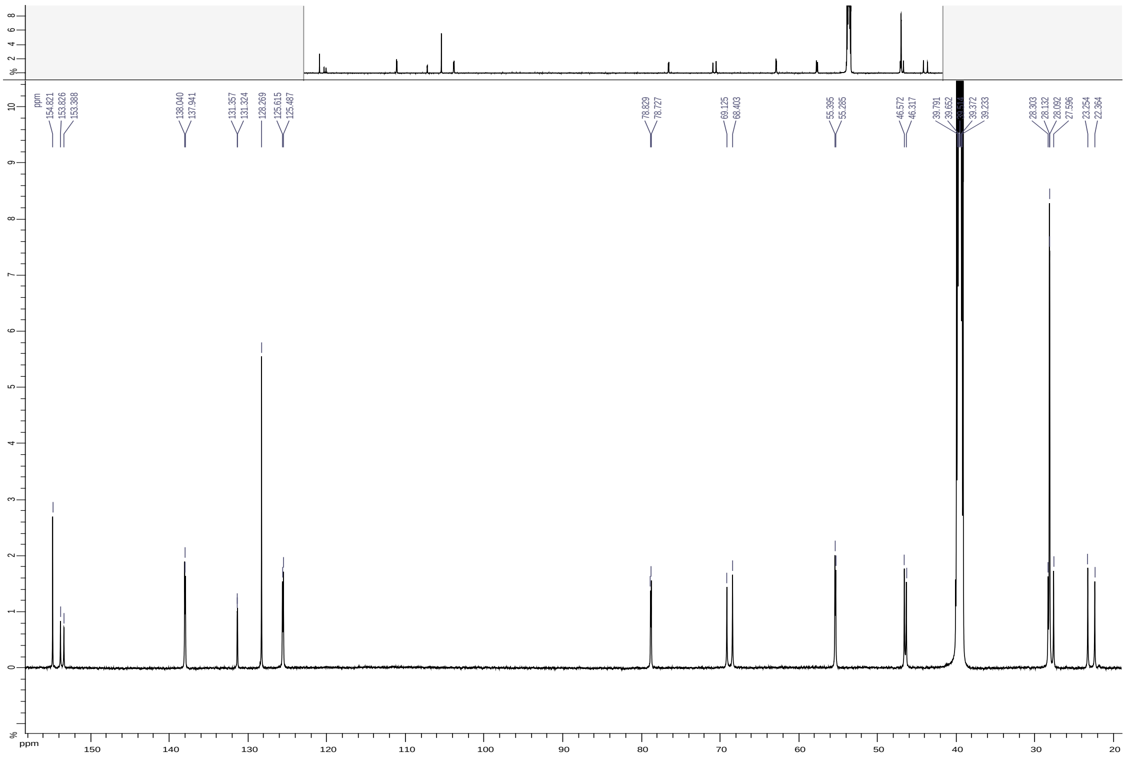

tert-Butyl 2-(((6-(phenylthio)pyridin-3-yl)oxy)methyl)pyrrolidine-1-carboxylate (12a)

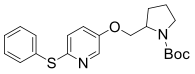

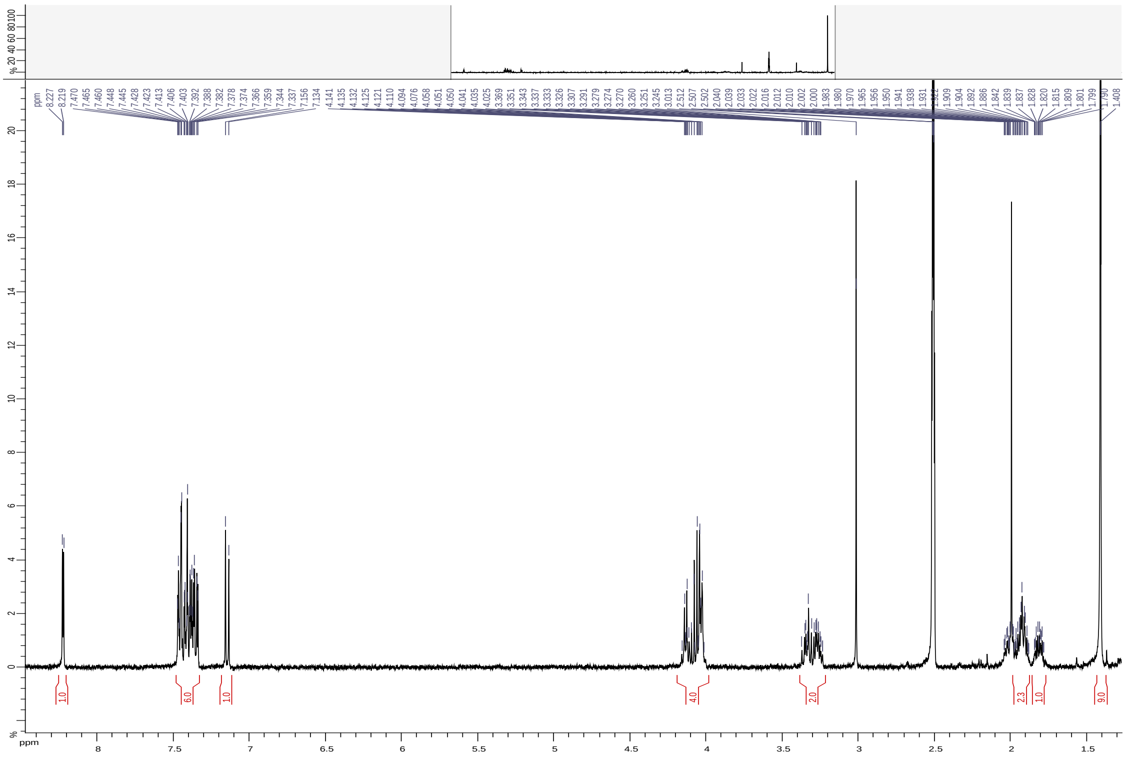

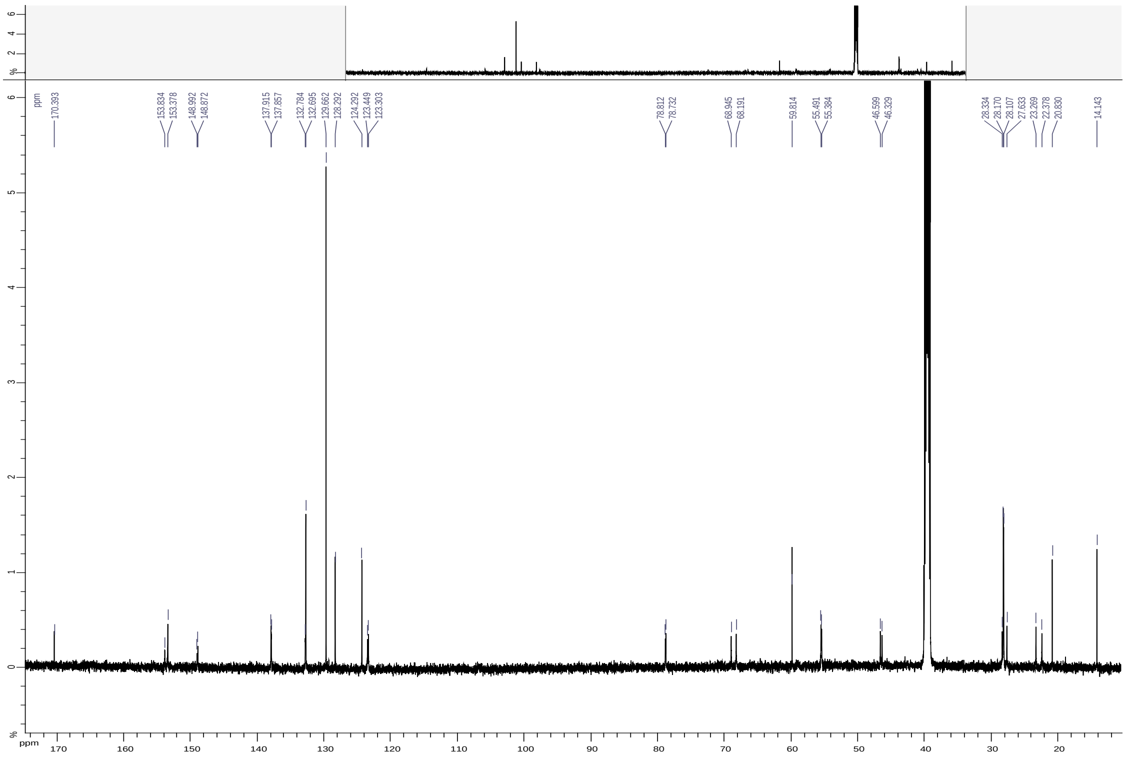

(5-((1-(*tert*-Butoxycarbonyl)pyrrolidin-2-yl)methoxy)pyridin-2-yl)diphenylsulfonium trifluoromethanesulfonate (12b)

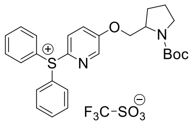

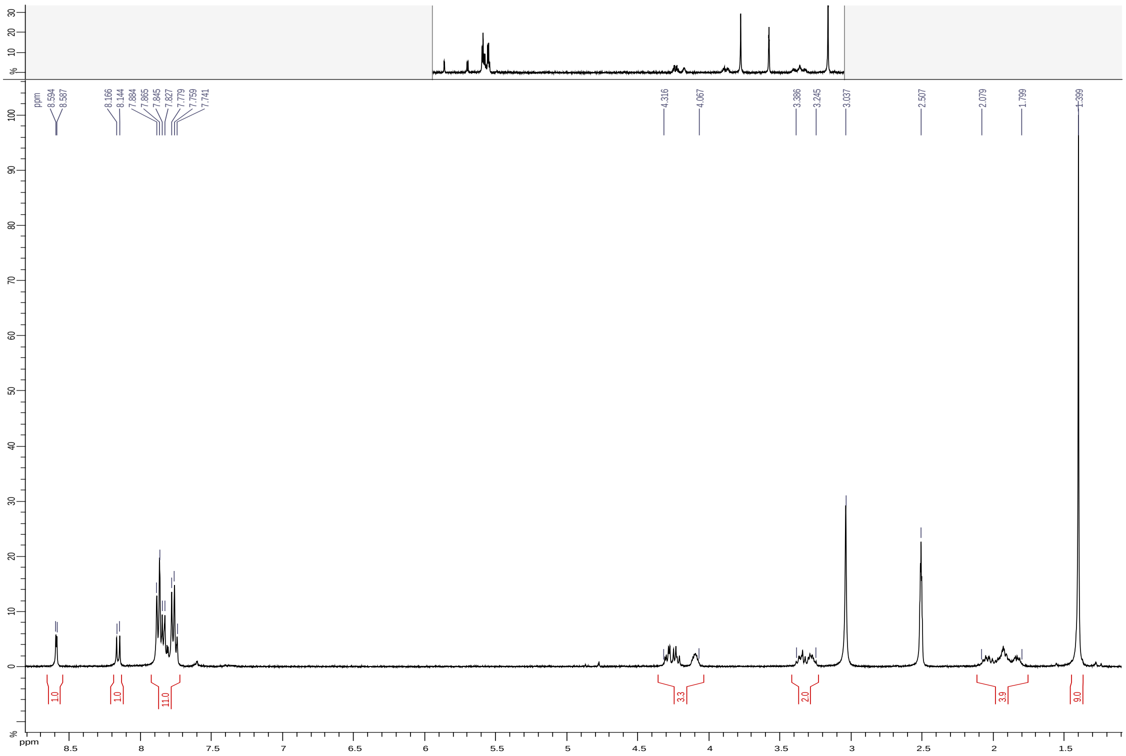

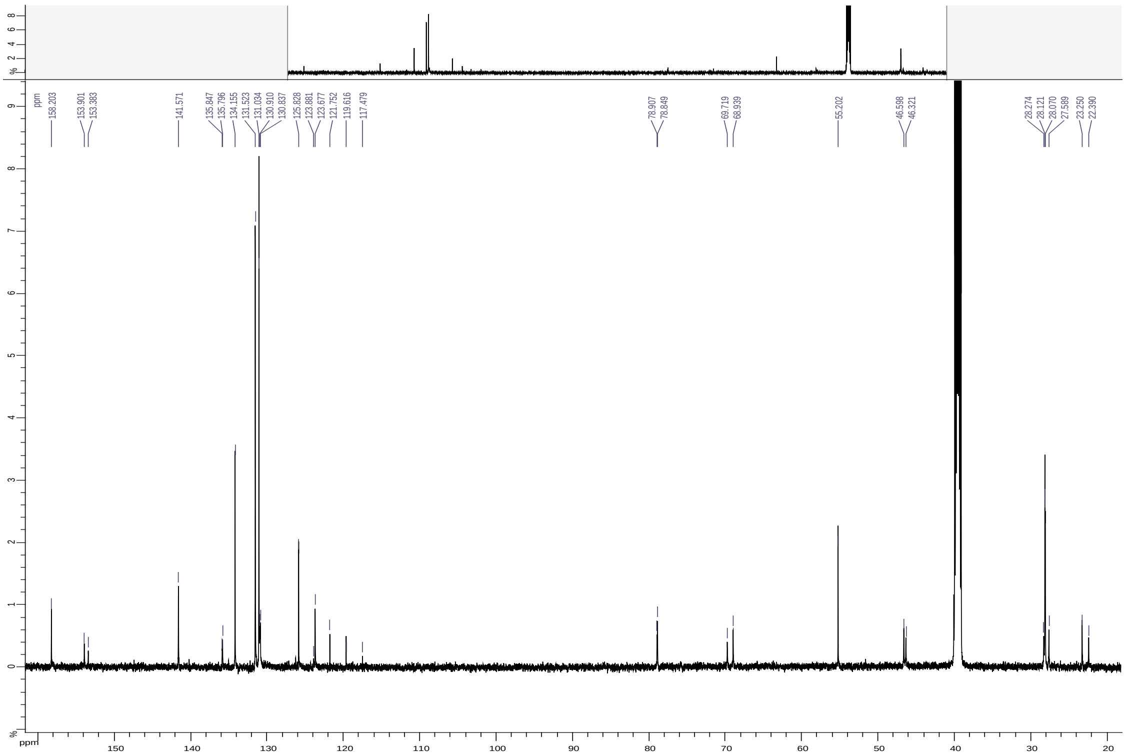

1-(2-Chloro-5-((4-methoxyphenyl)thio)phenyl)ethan-1-one (M)

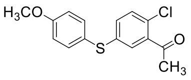

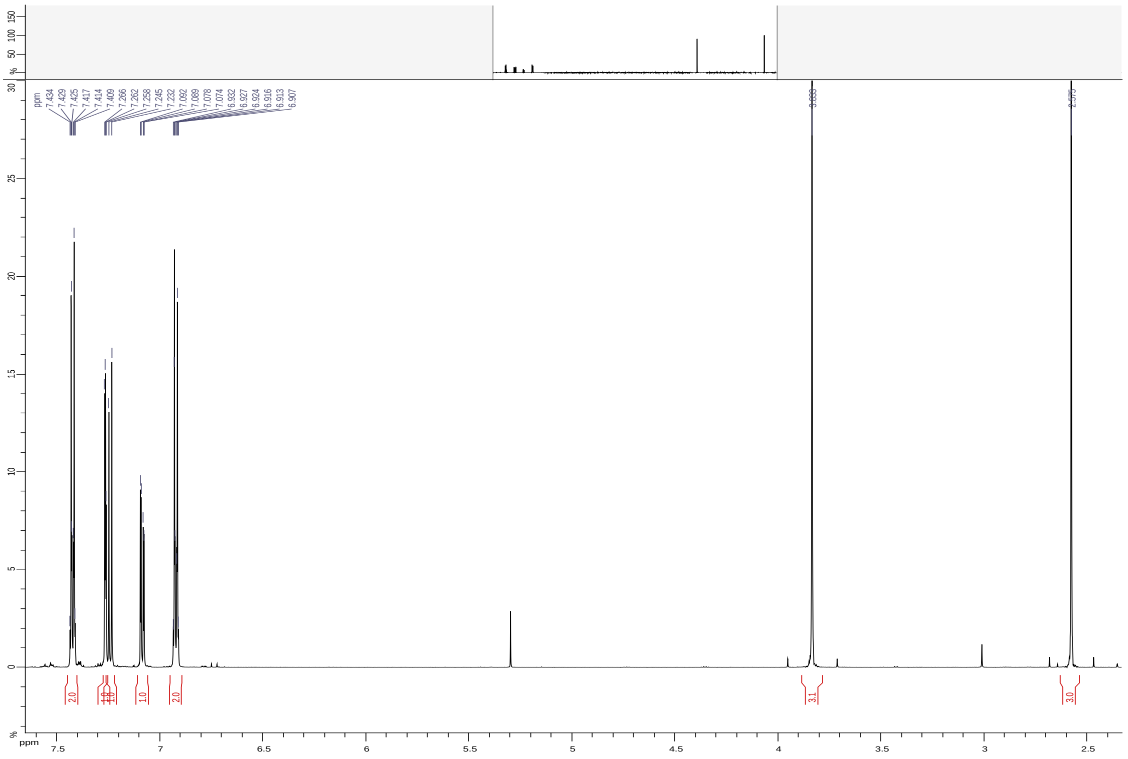

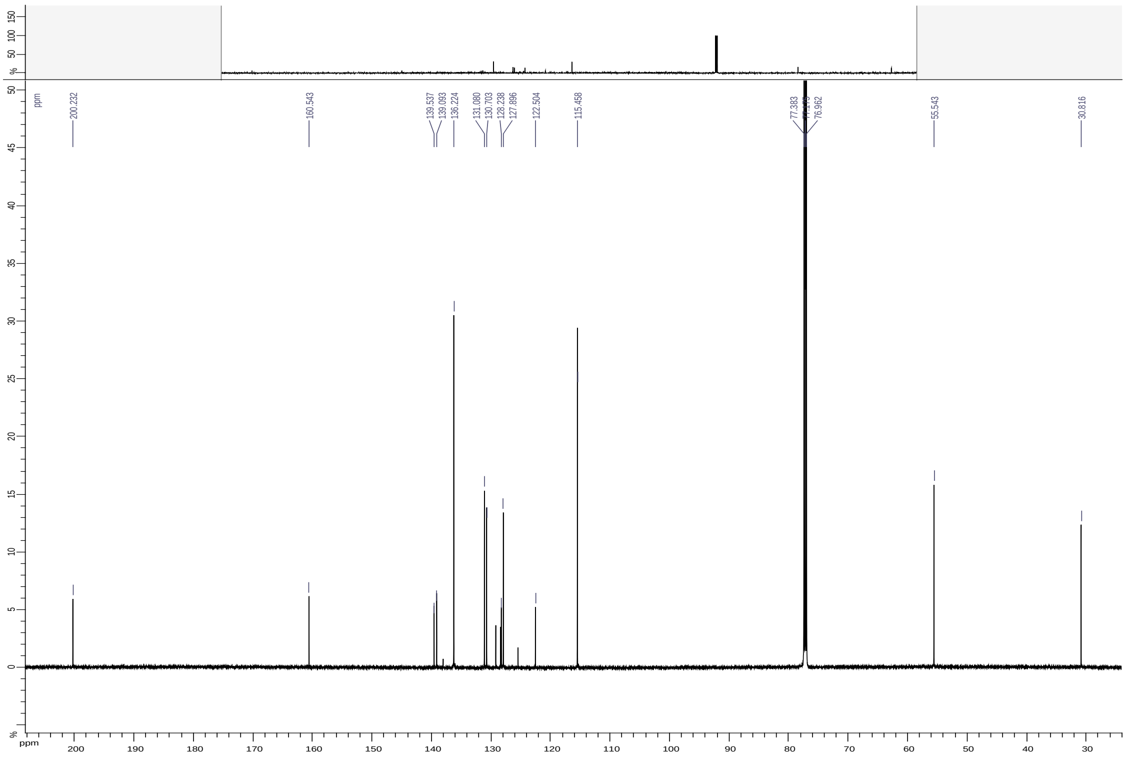

1-(2-Chloro-5-((4-methoxyphenyl)thio)phenyl)ethan-1-ol (N)

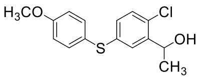

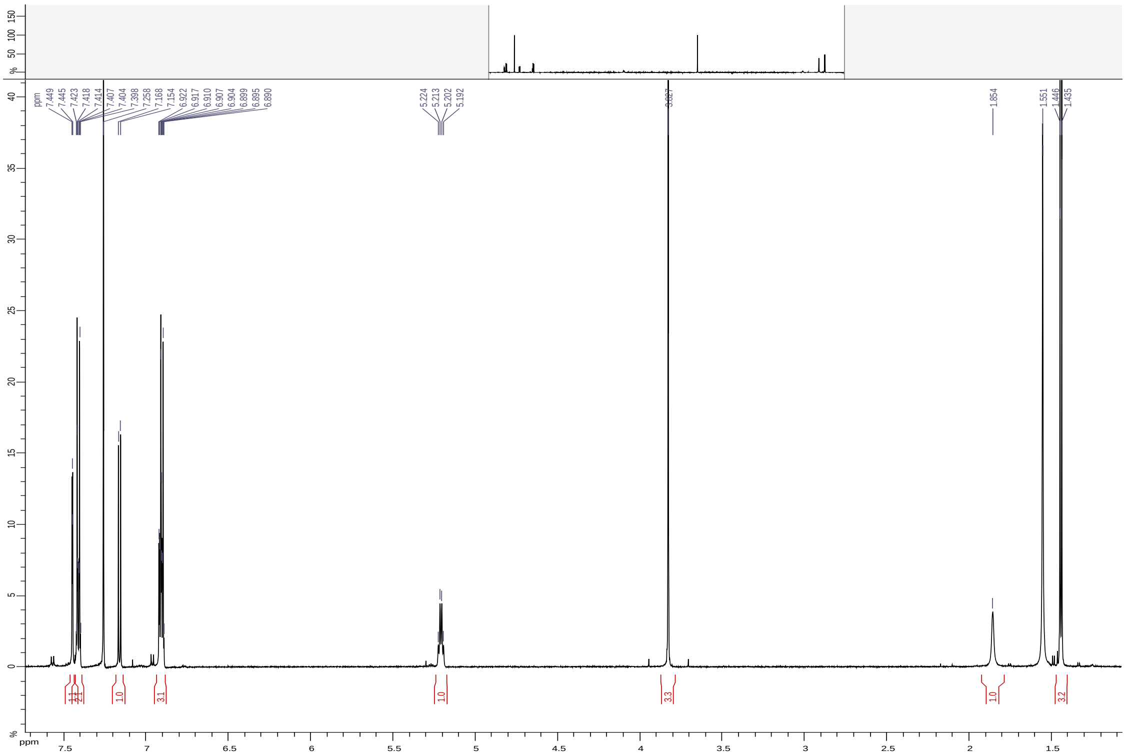

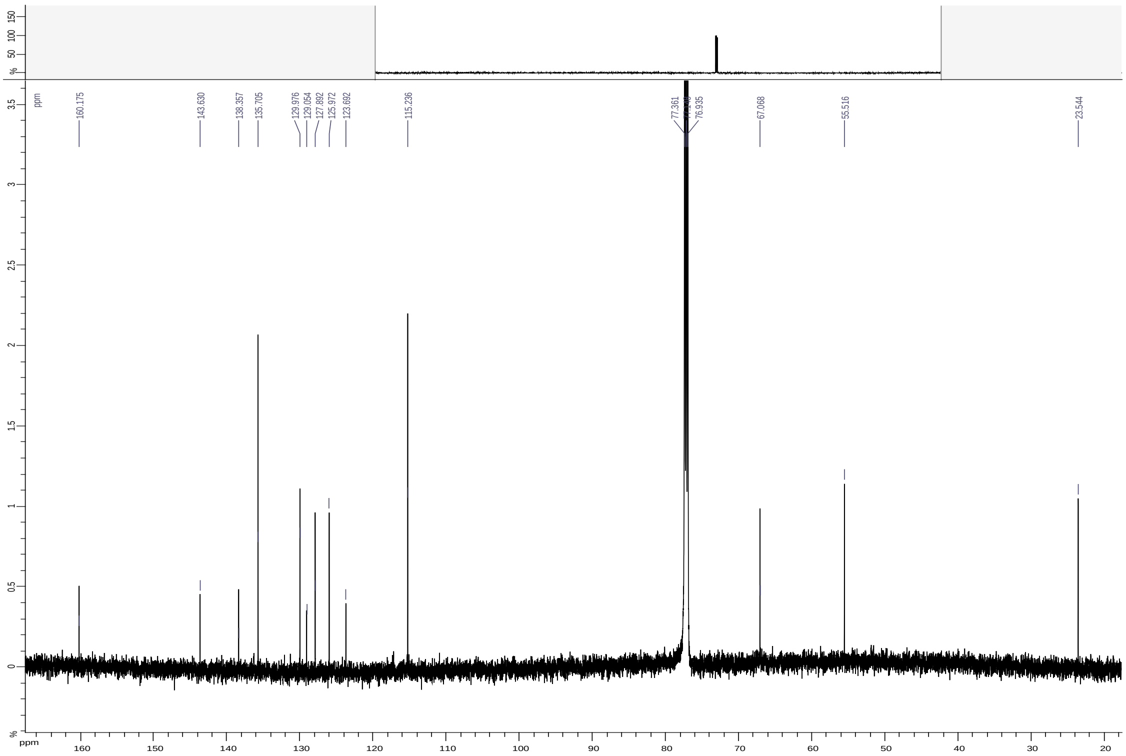

(3-(1-Bromoethyl)-4-chlorophenyl)(4-methoxyphenyl)sulfane (O)

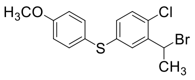

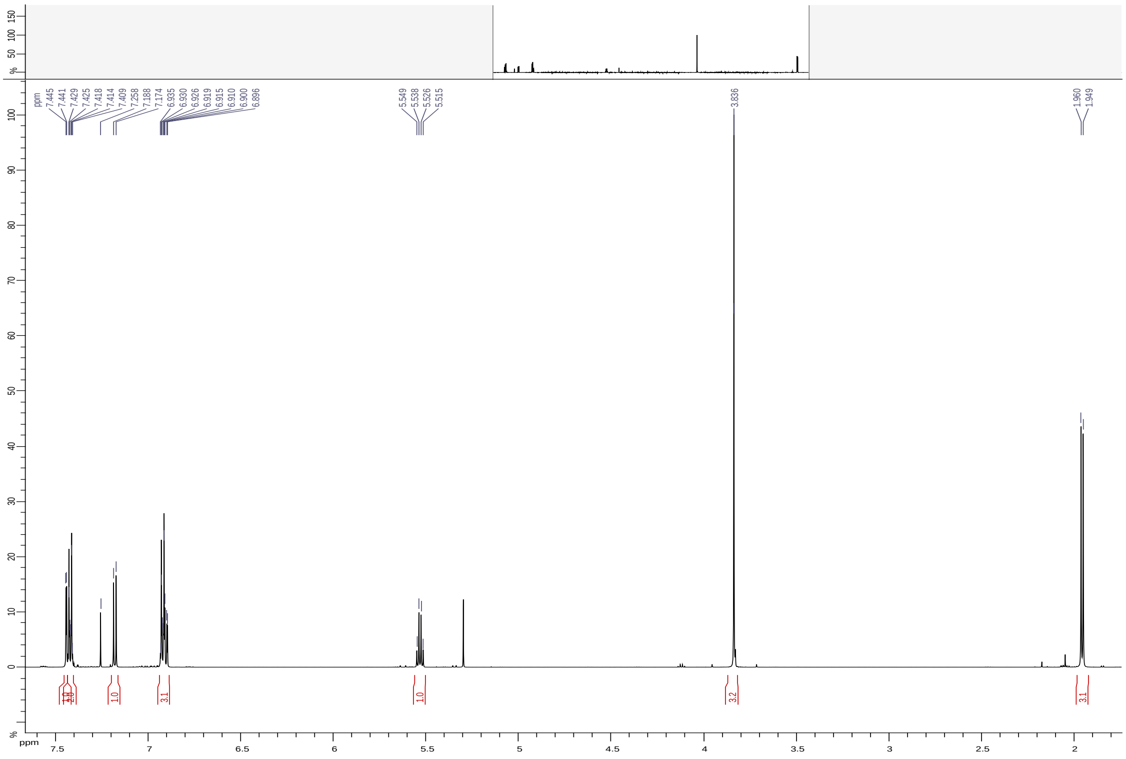

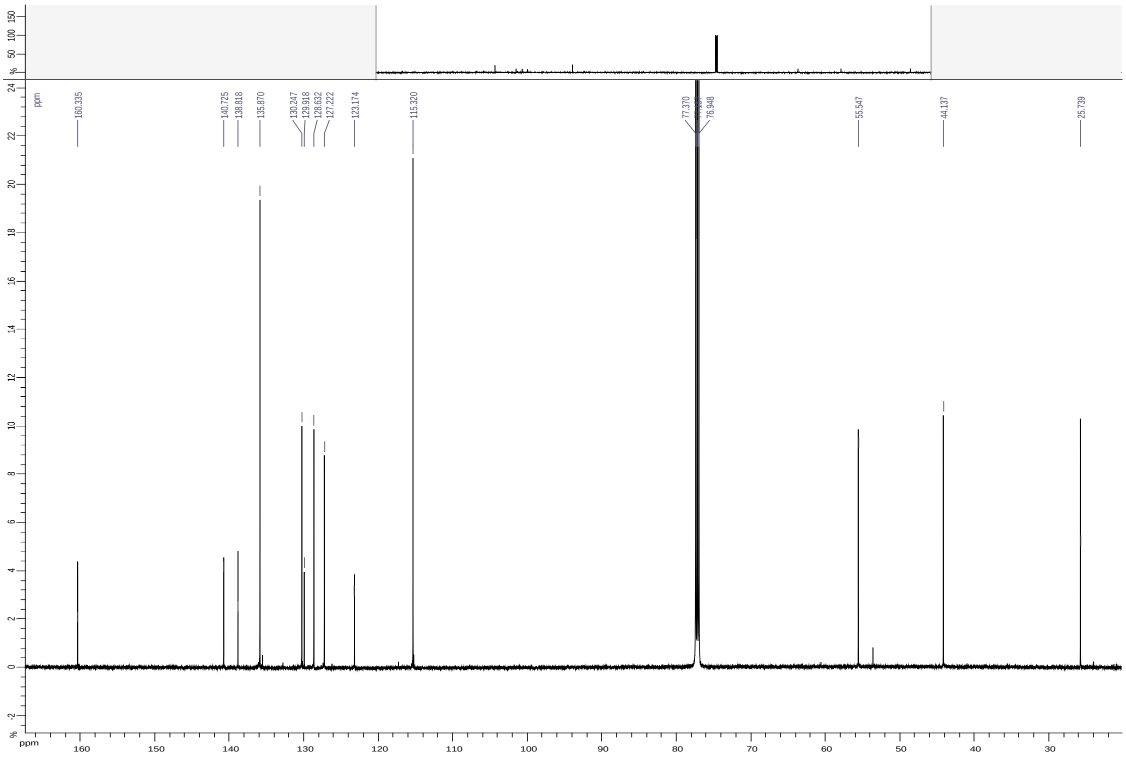

1-(1-(2-Chloro-5-((4-methoxyphenyl)thio)phenyl)ethyl)-1*H*-imidazole (13a)

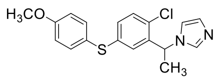

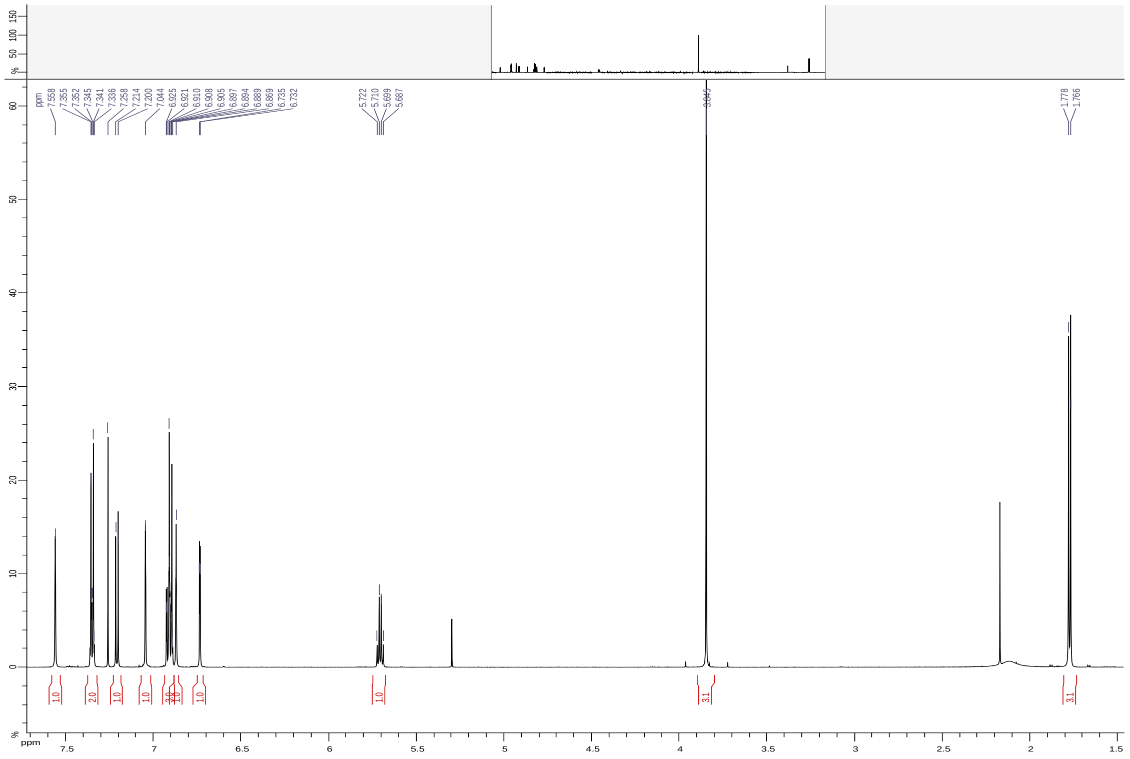

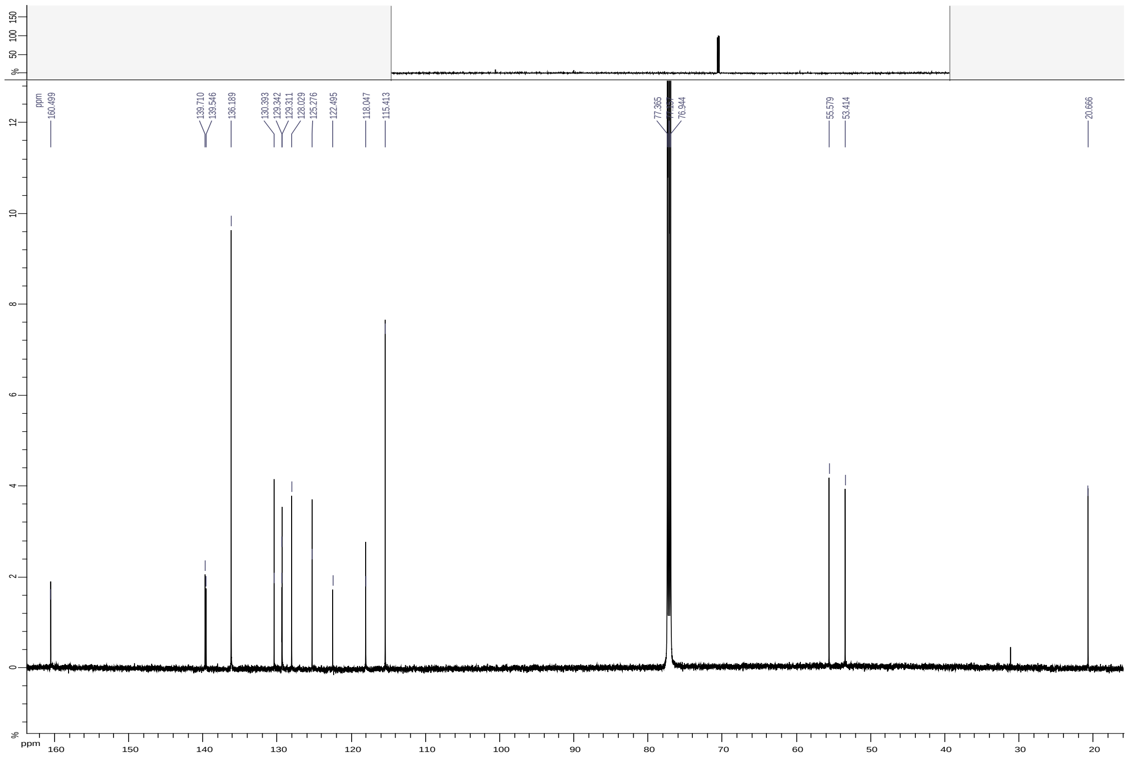

(3-(1-(1*H*-Imidazol-1-yl)ethyl)-4-chlorophenyl)bis(4-methoxyphenyl)sulfonium trifluoromethanesulfonate (13b)

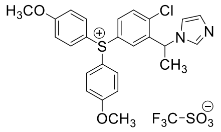

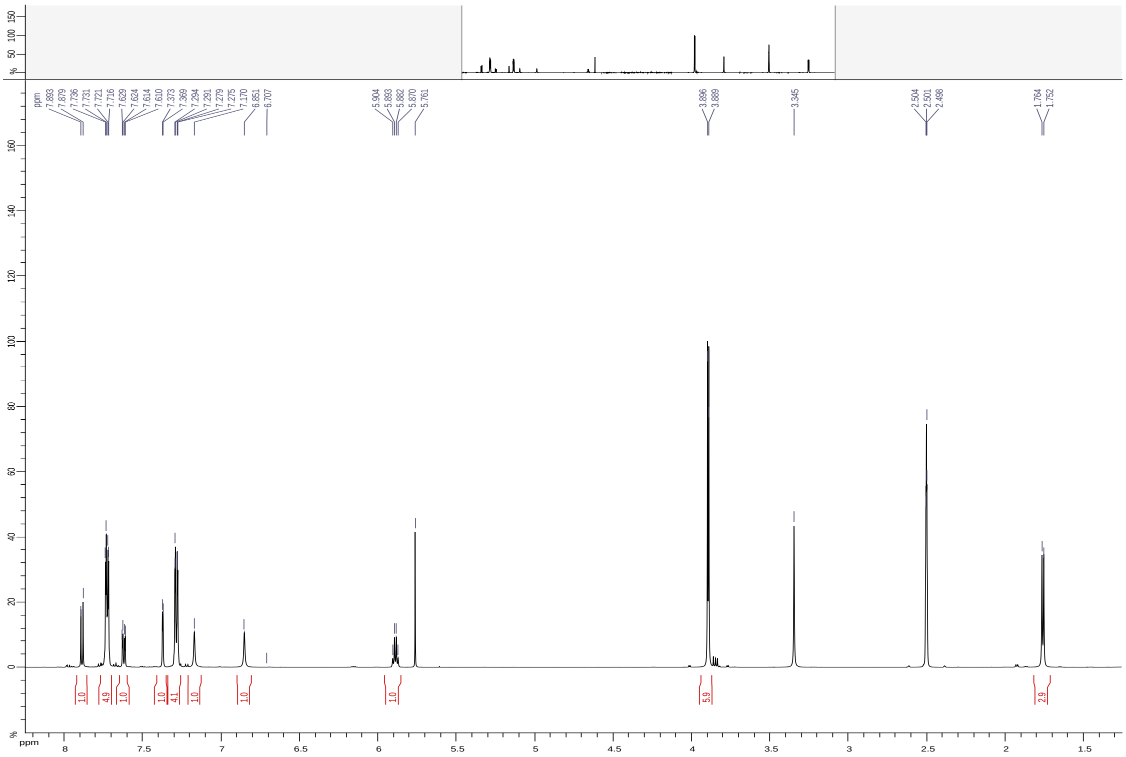

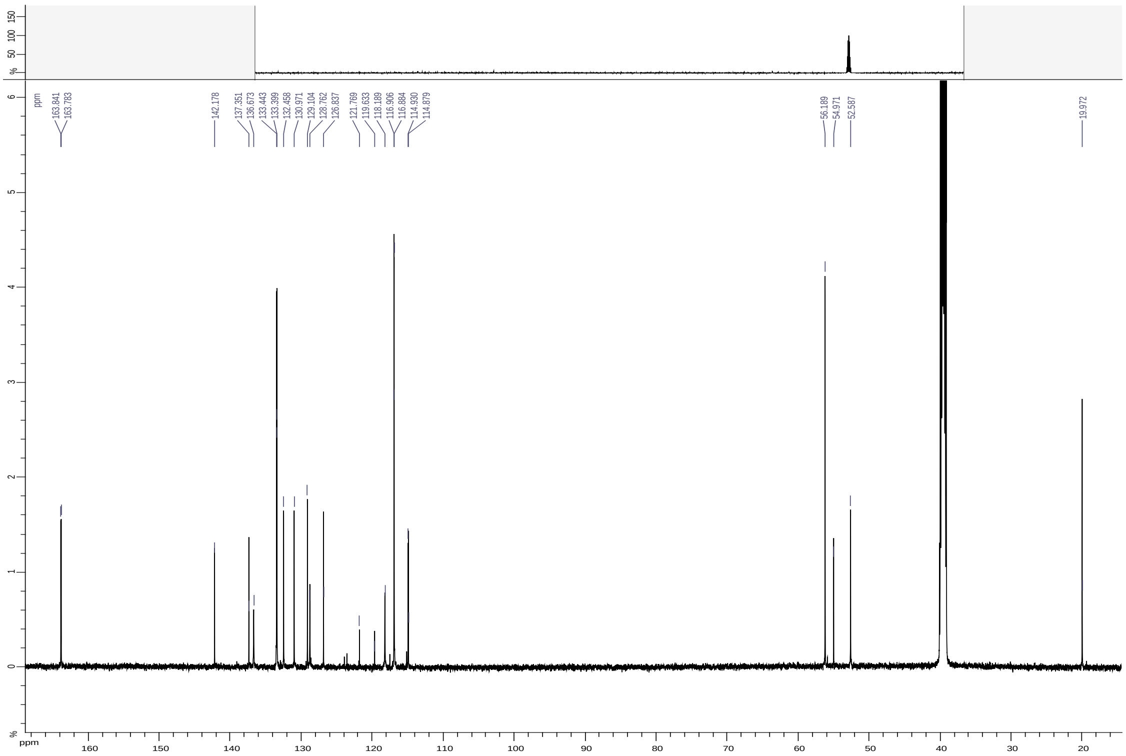

N-Pentylquinolin-4-amine (P)

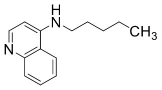

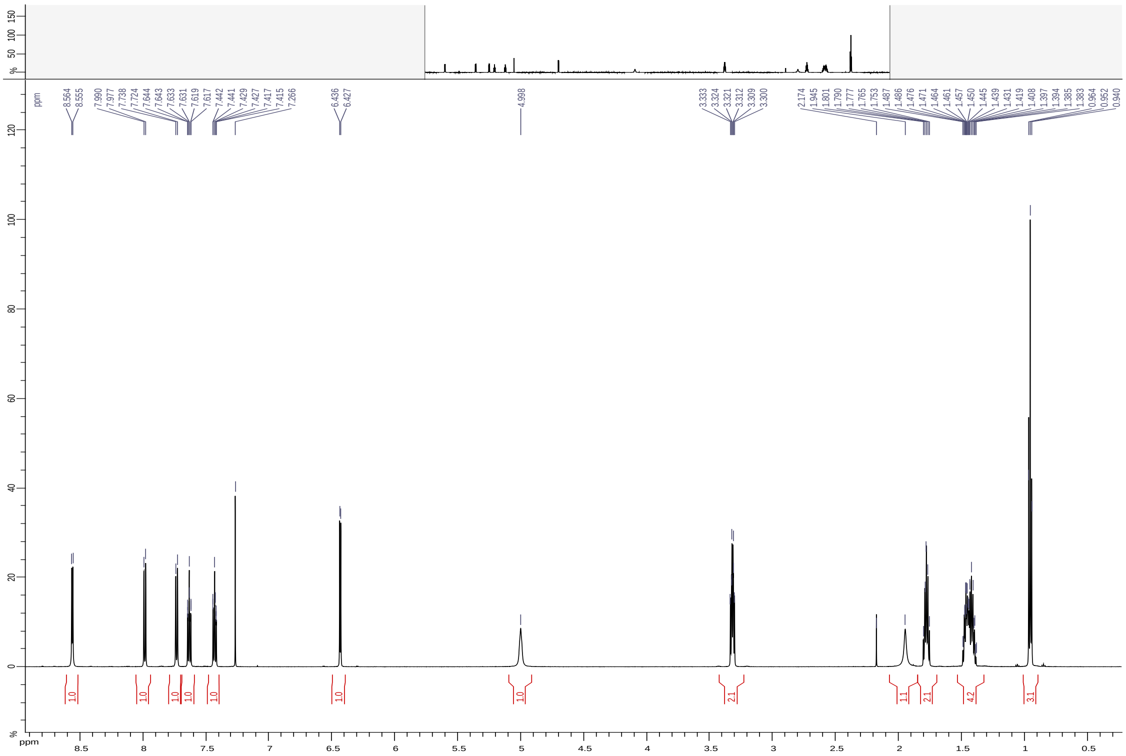

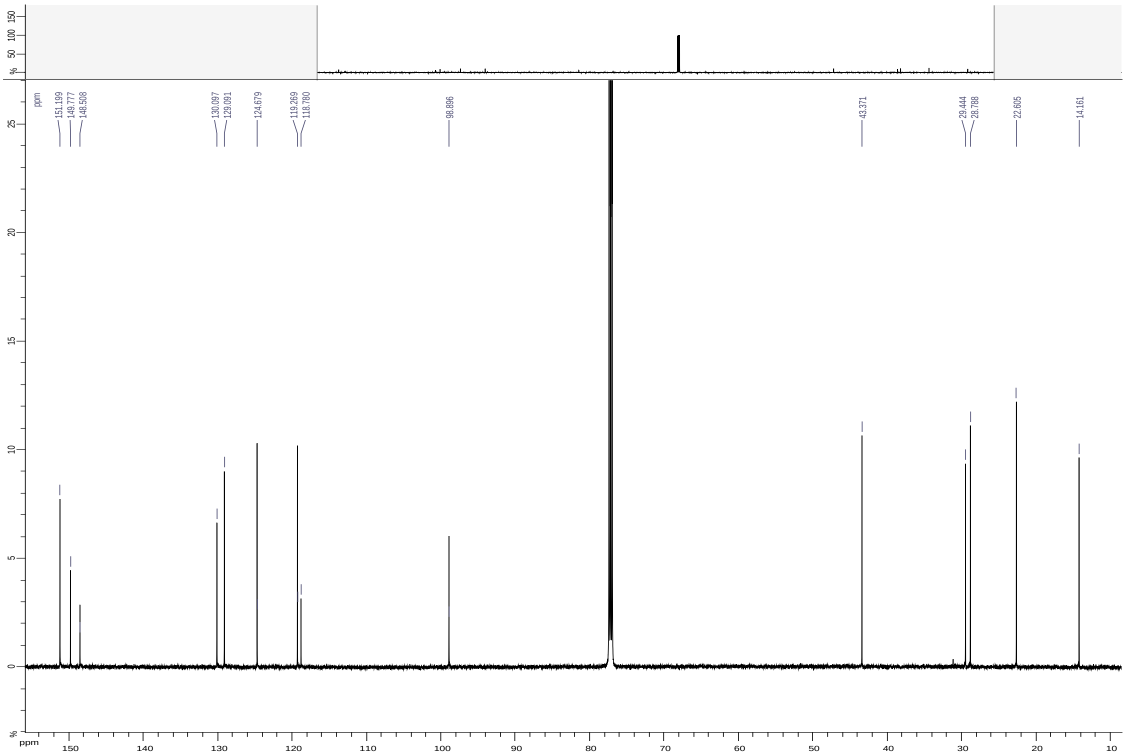

1-(3-Iodobenzyl)-*N*-pentylquinolin-4(1*H*)-imine (Q)

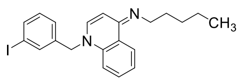

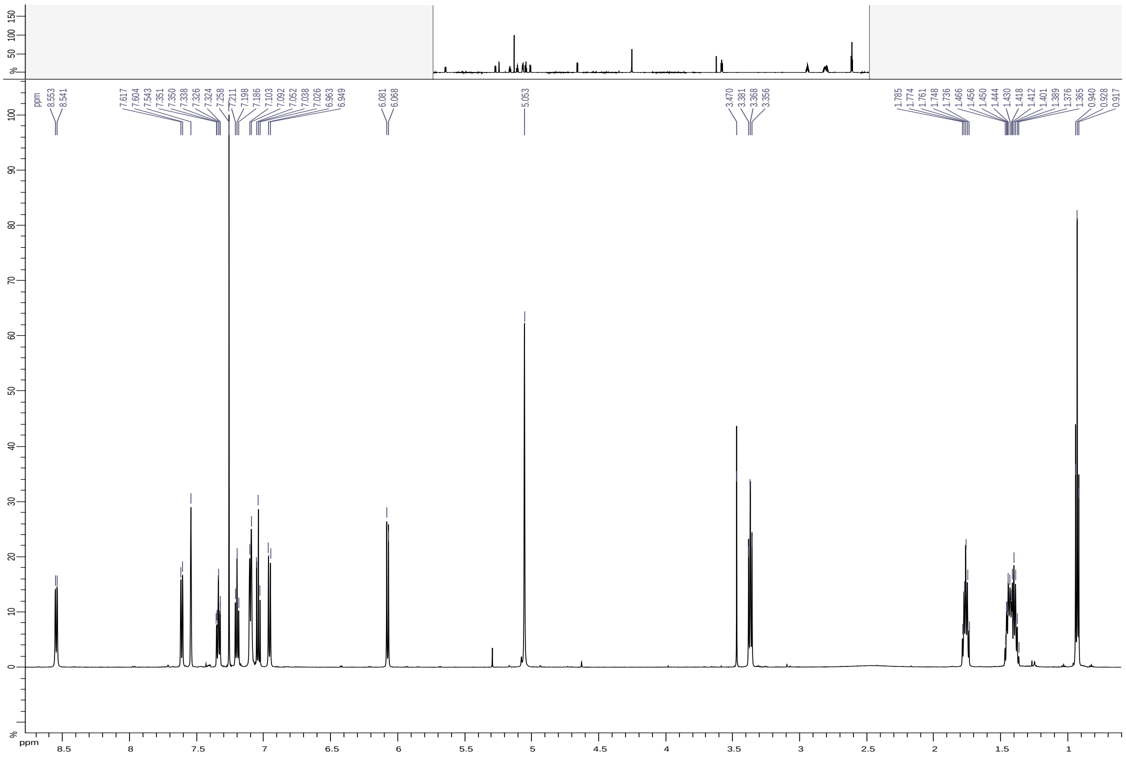

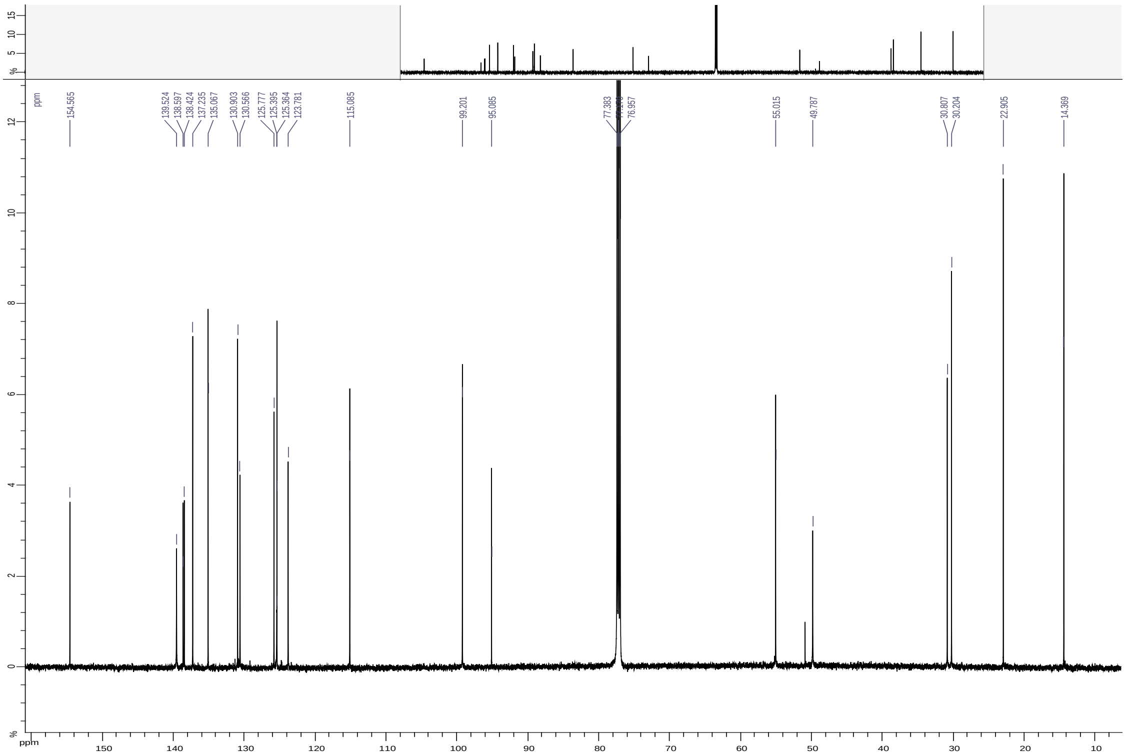

1-(3-((4-Methoxyphenyl)thio)benzyl)-*N*-pentylquinolin-4(1*H*)-imine (14a)

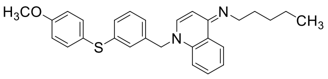

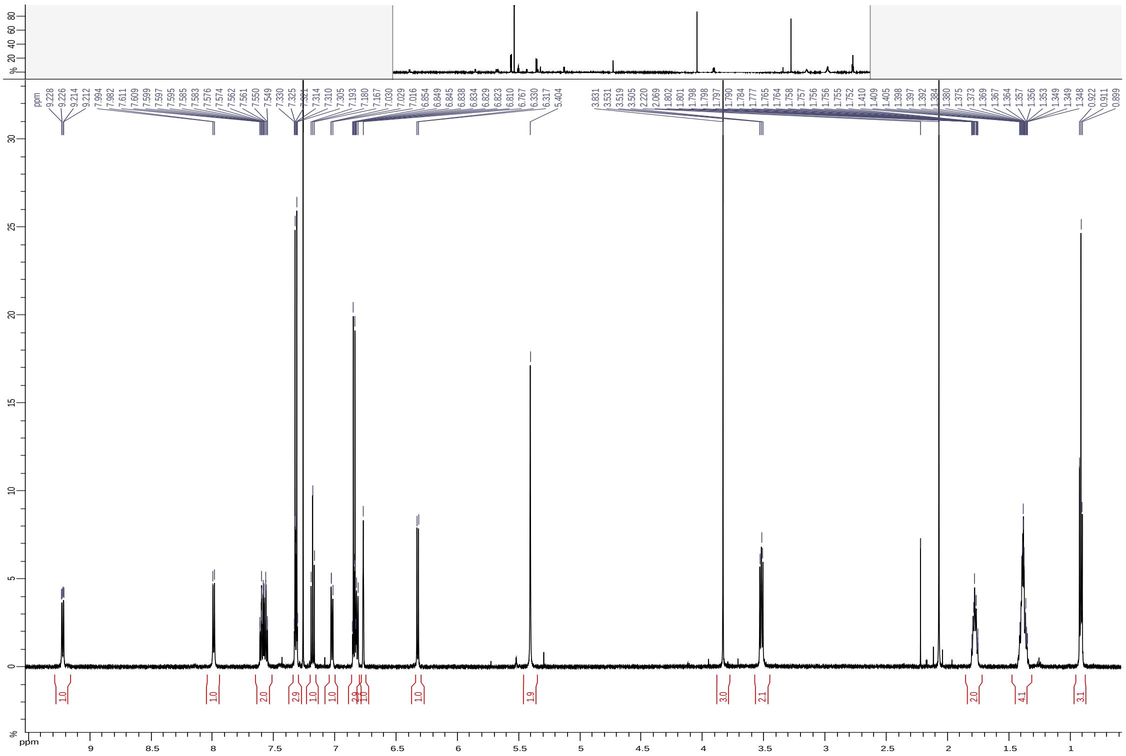

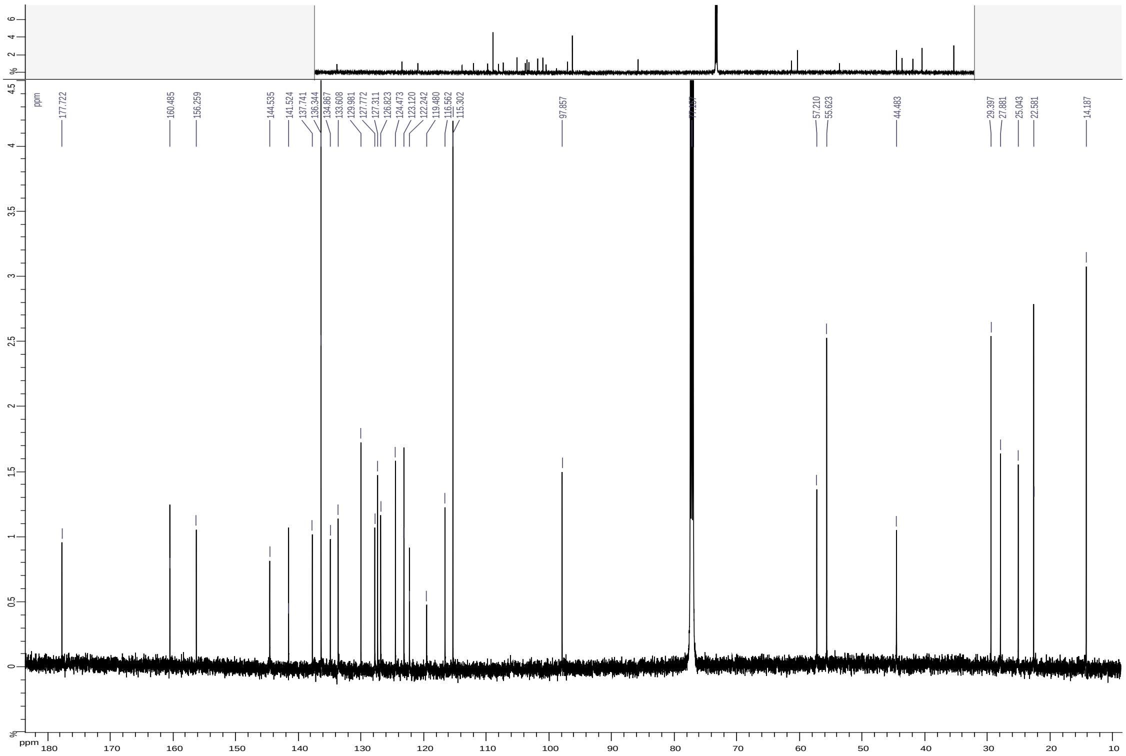

Bis(4-methoxyphenyl)(3-((4-(pentylimino)quinolin-1(4*H*)-yl)methyl)phenyl)sulfonium trifluoromethanesulfonate (14b)

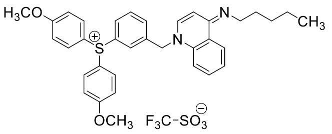

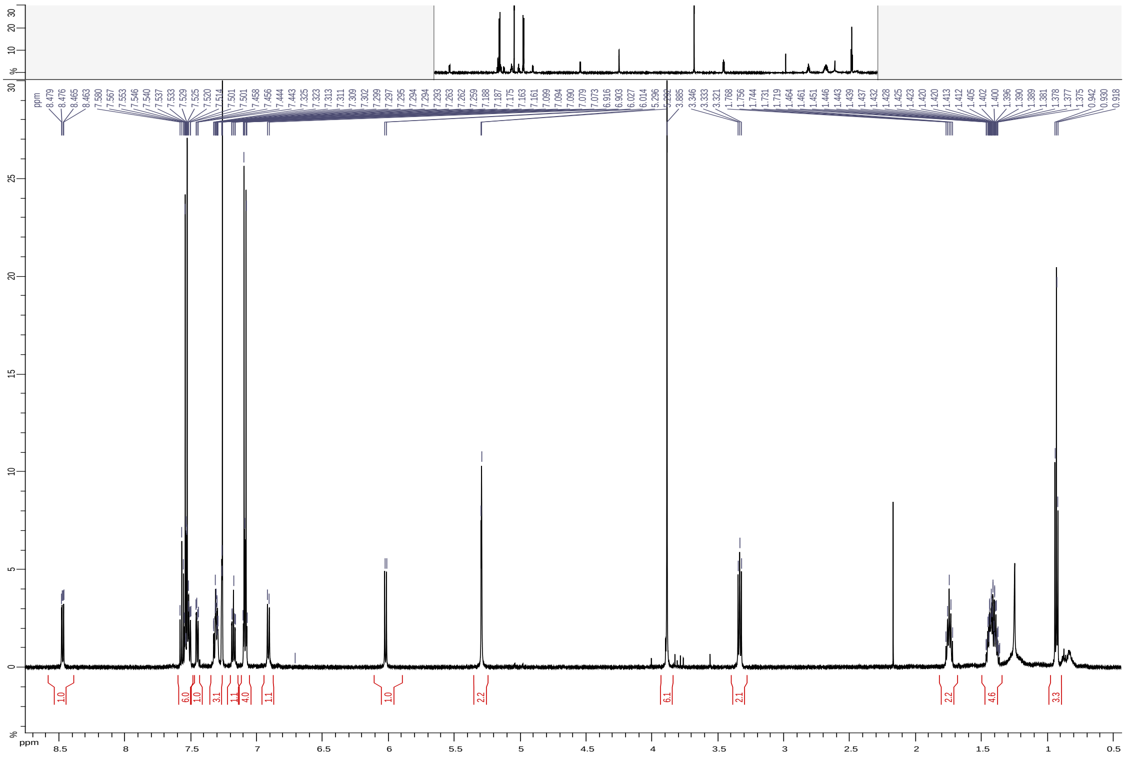

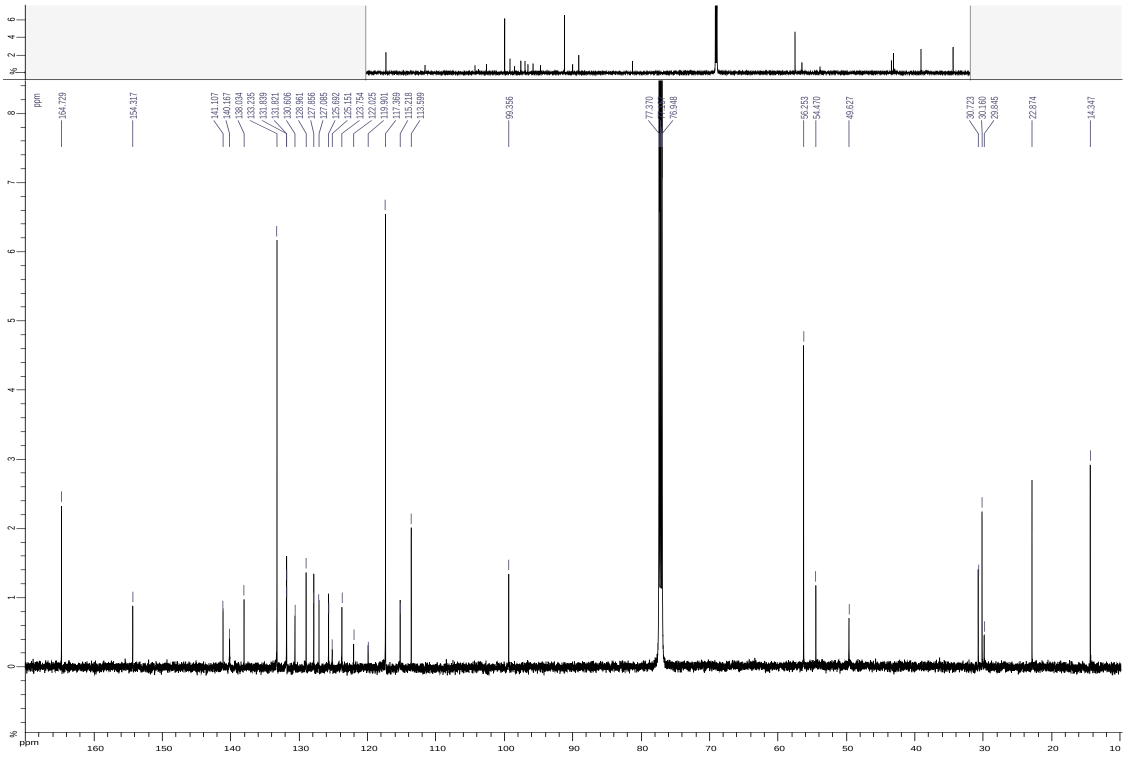

tert-Butyl 4-formylpiperidine-1-carboxylate (R)

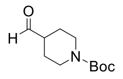

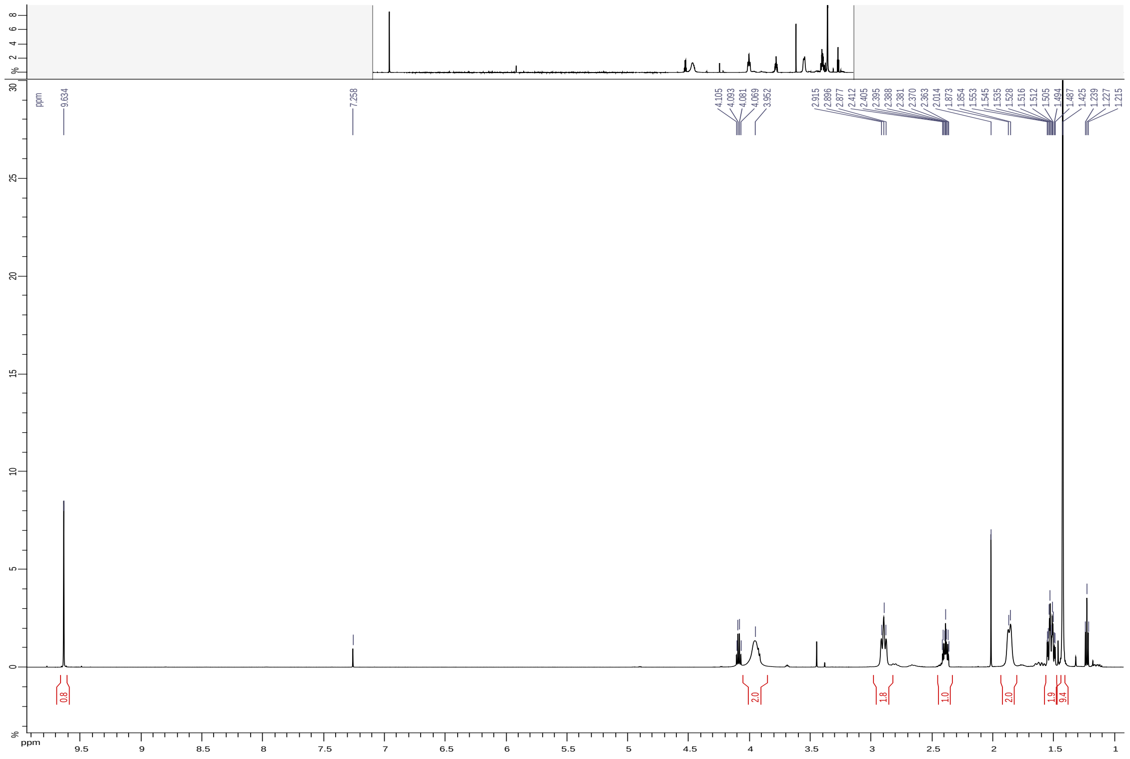

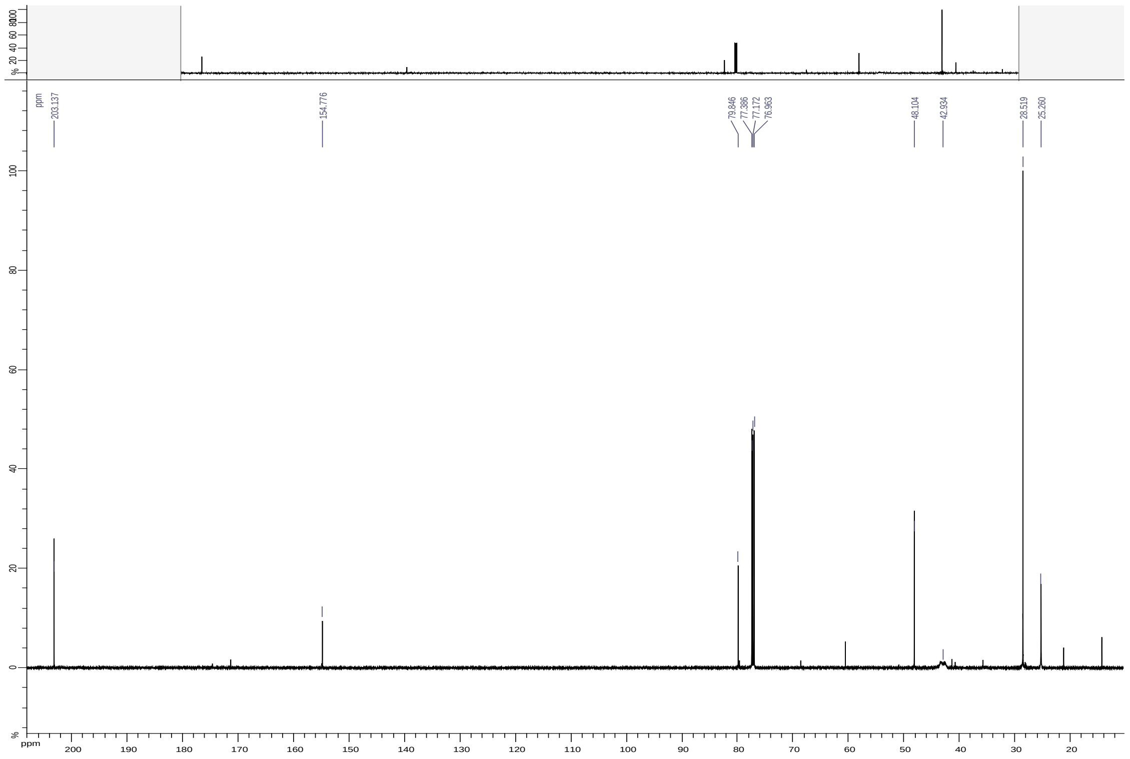

tert-Butyl 4-((4-fluorophenyl)(hydroxy)methyl)piperidine-1-carboxylate (S)

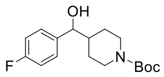

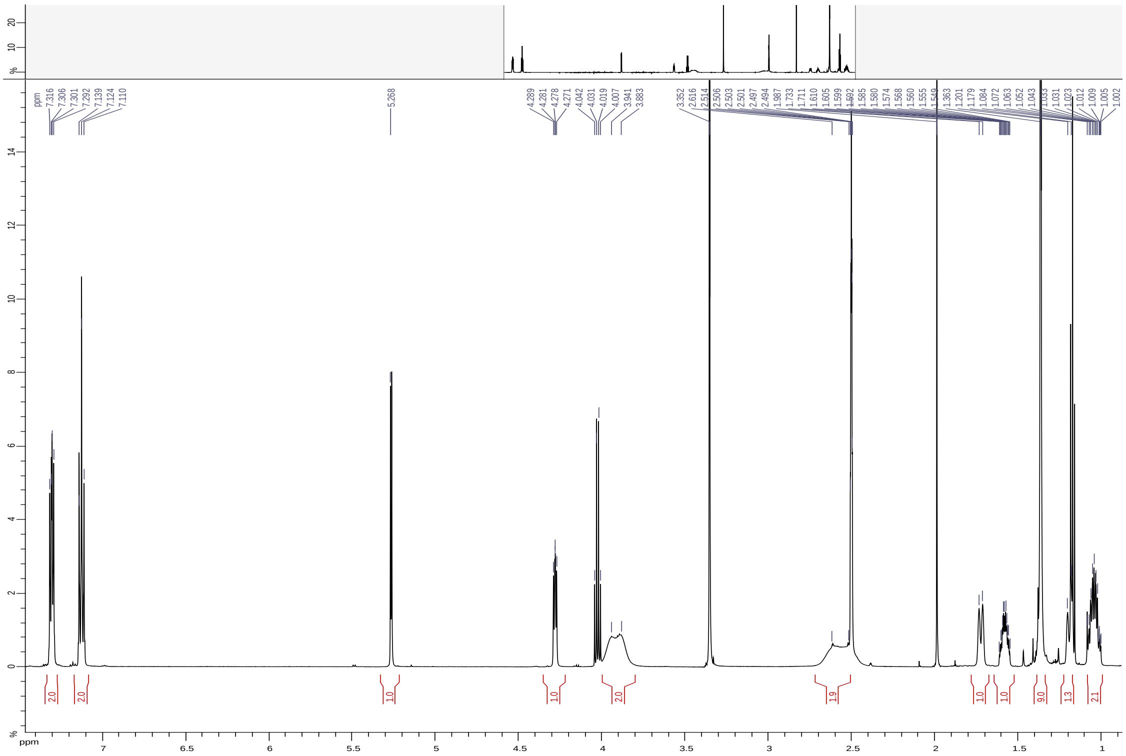

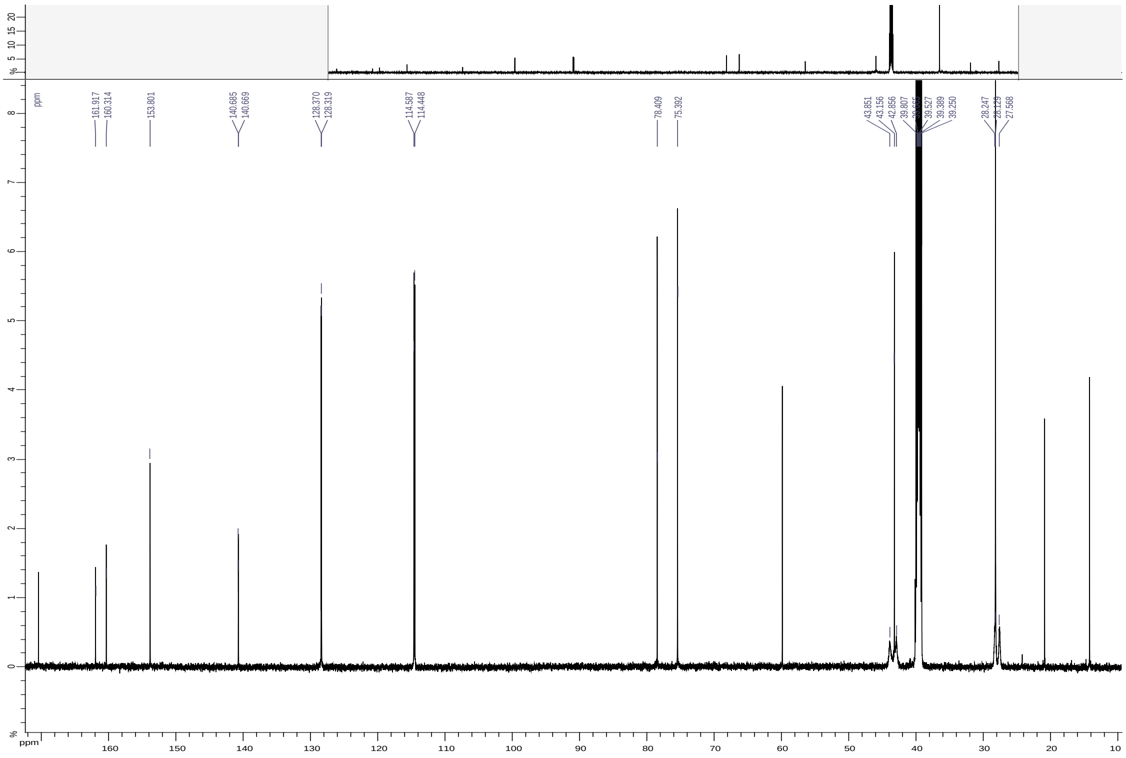

tert-Butyl 4-(4-fluorobenzoyl)piperidine-1-carboxylate (T)

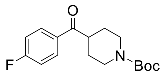

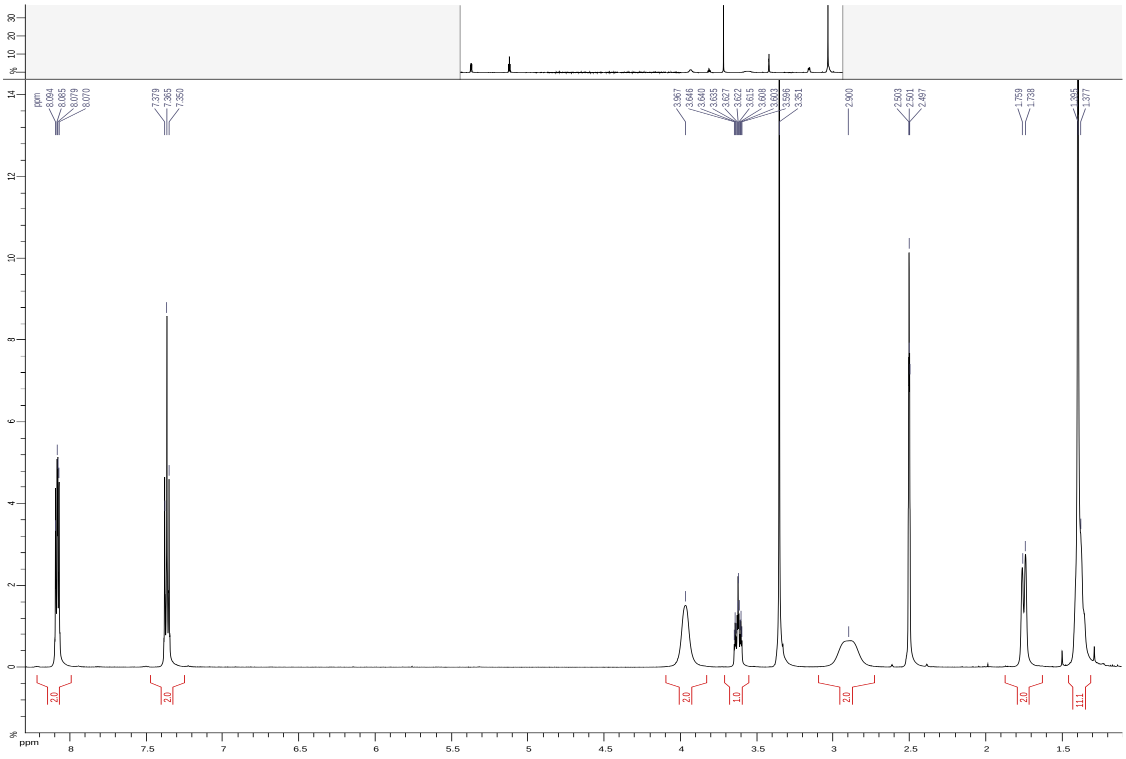

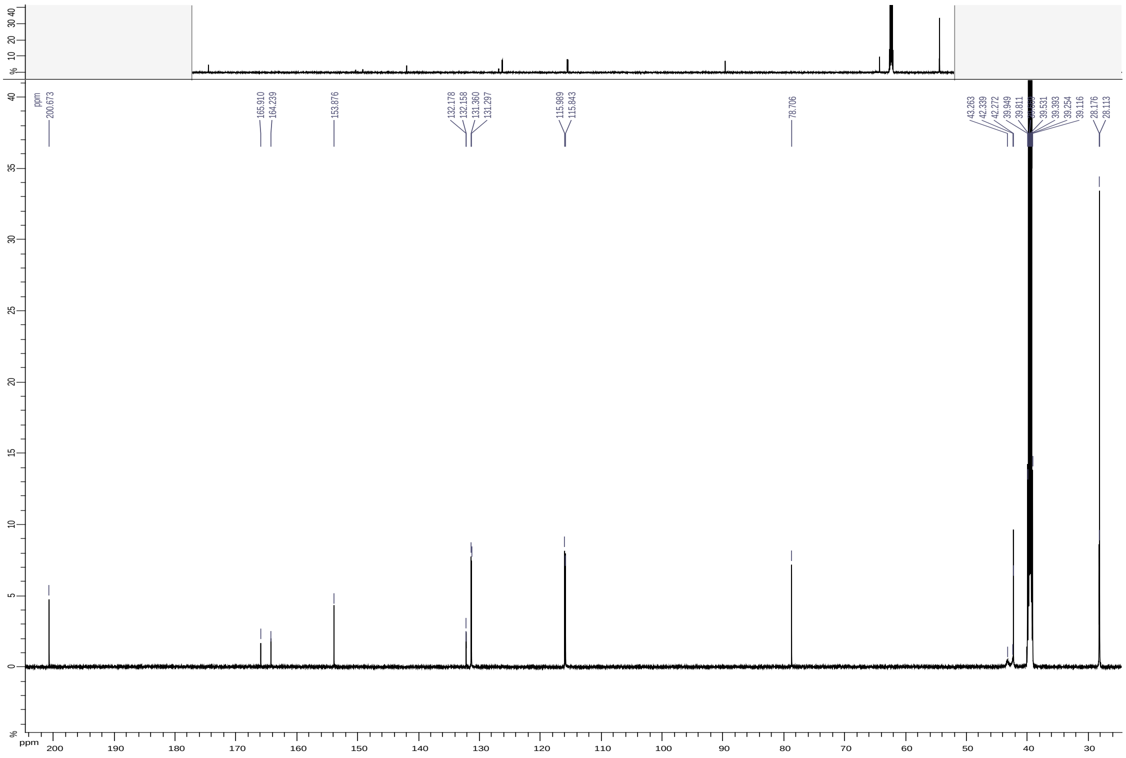

(4-Fluorophenyl)(piperidin-4-yl)methanone hydrochloride (4c)

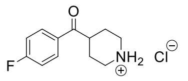

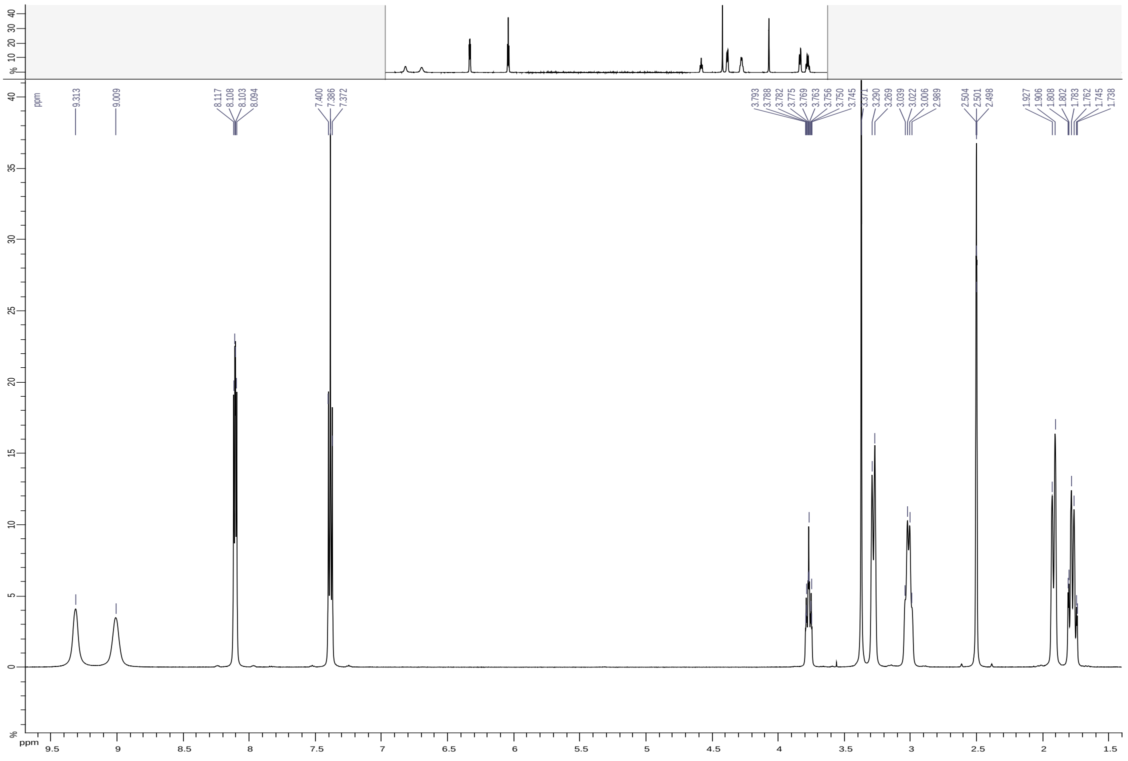

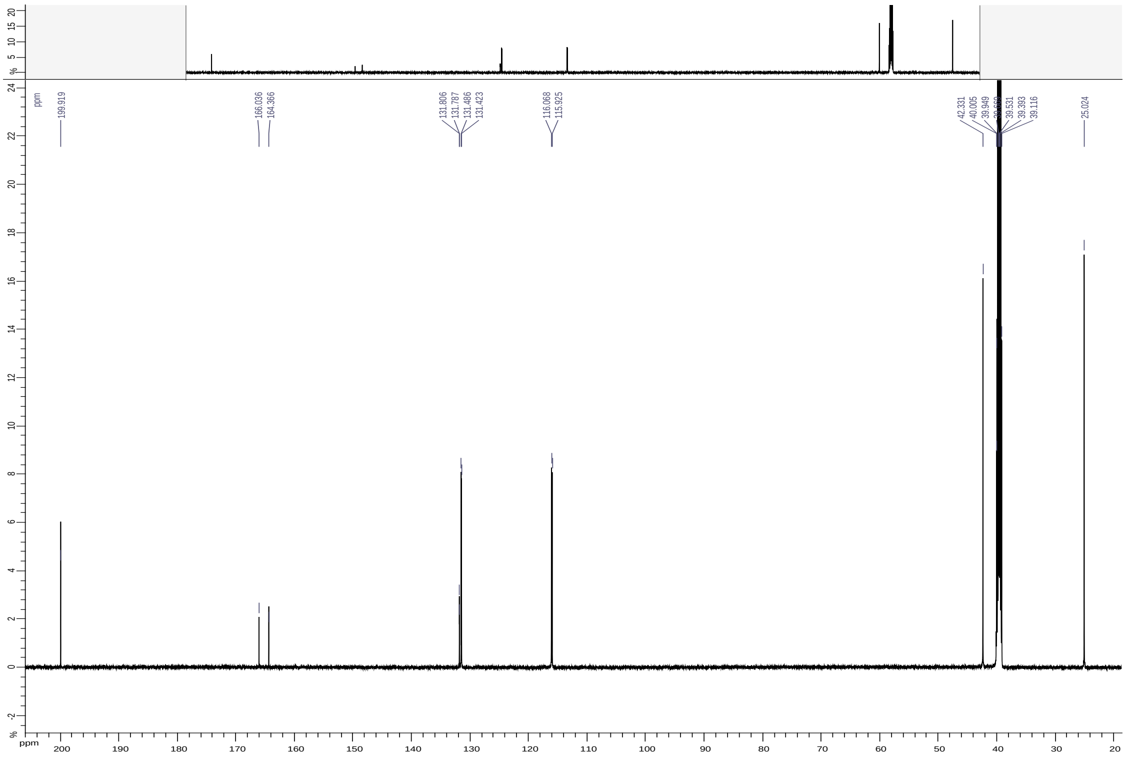

4-(4-(4-Fluorobenzoyl)piperidin-1-yl)butyl acetate (1c)

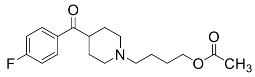

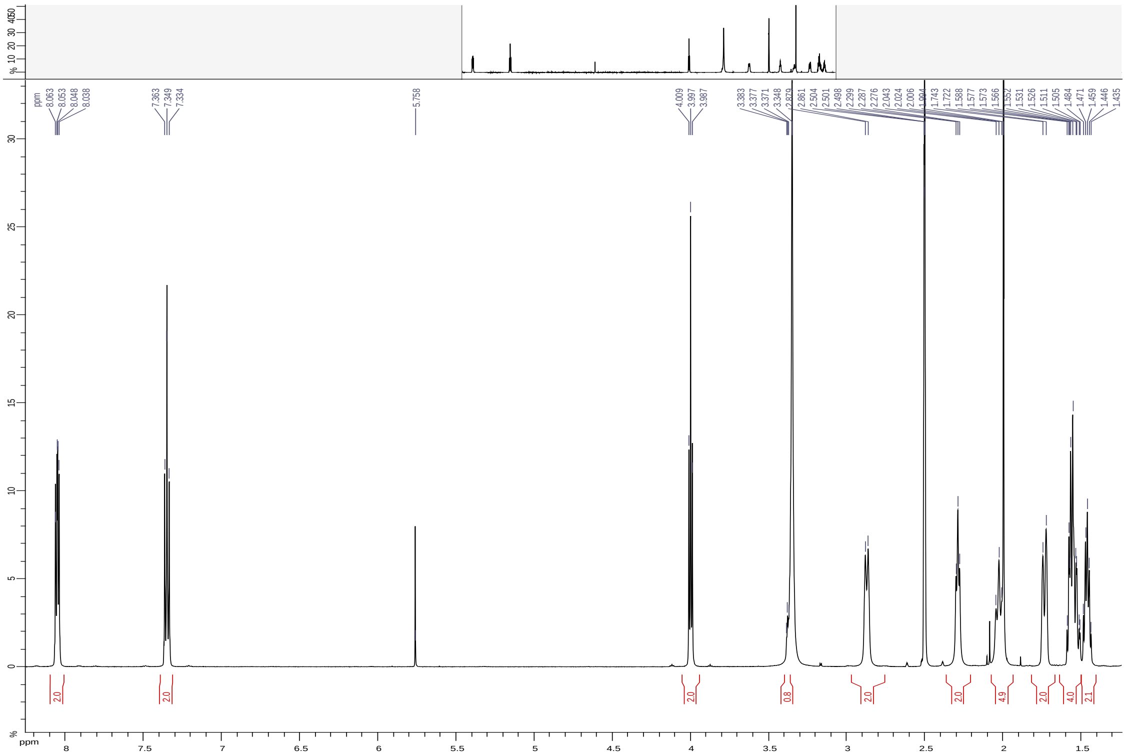

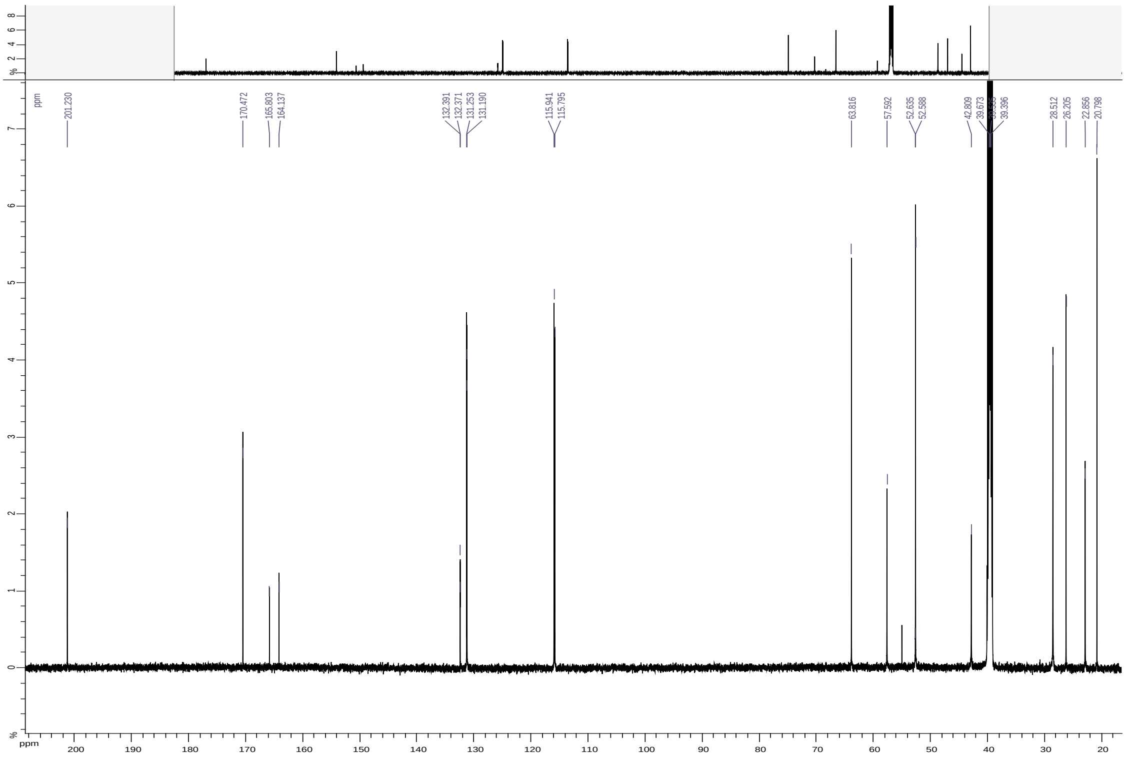

(4-Fluorophenyl)(1-(4-hydroxybutyl)piperidin-4-yl)methanone (3c)

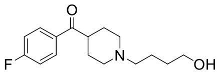

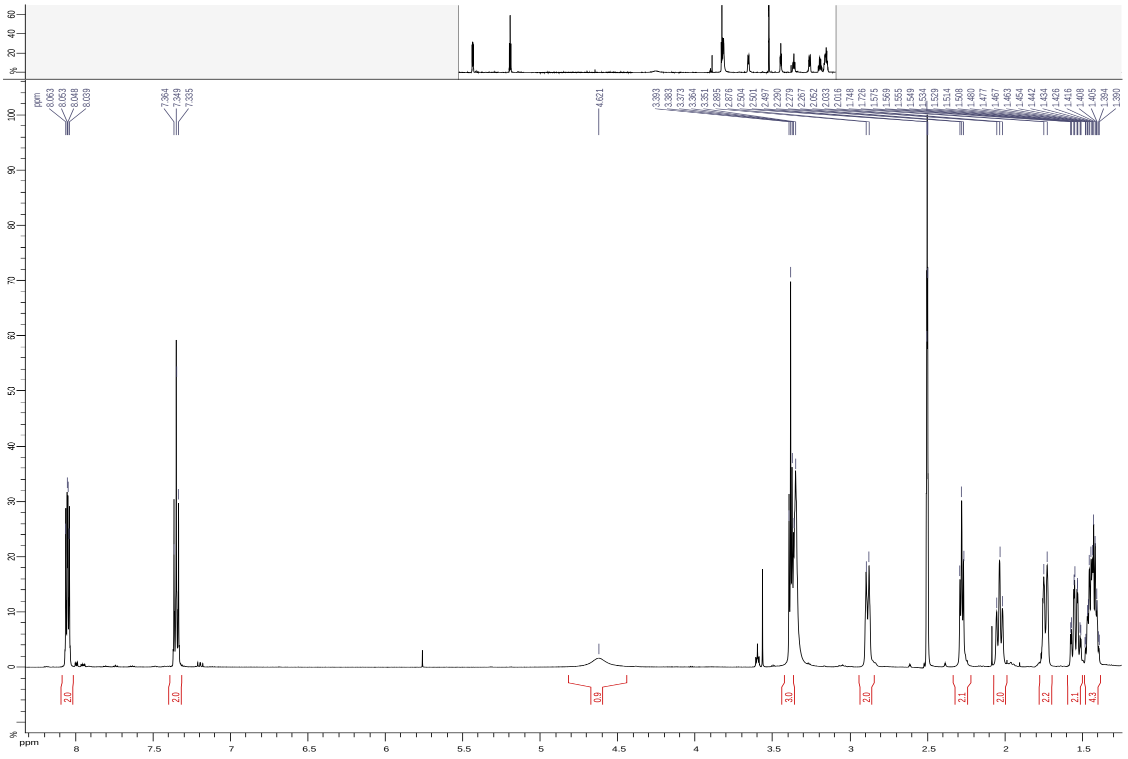

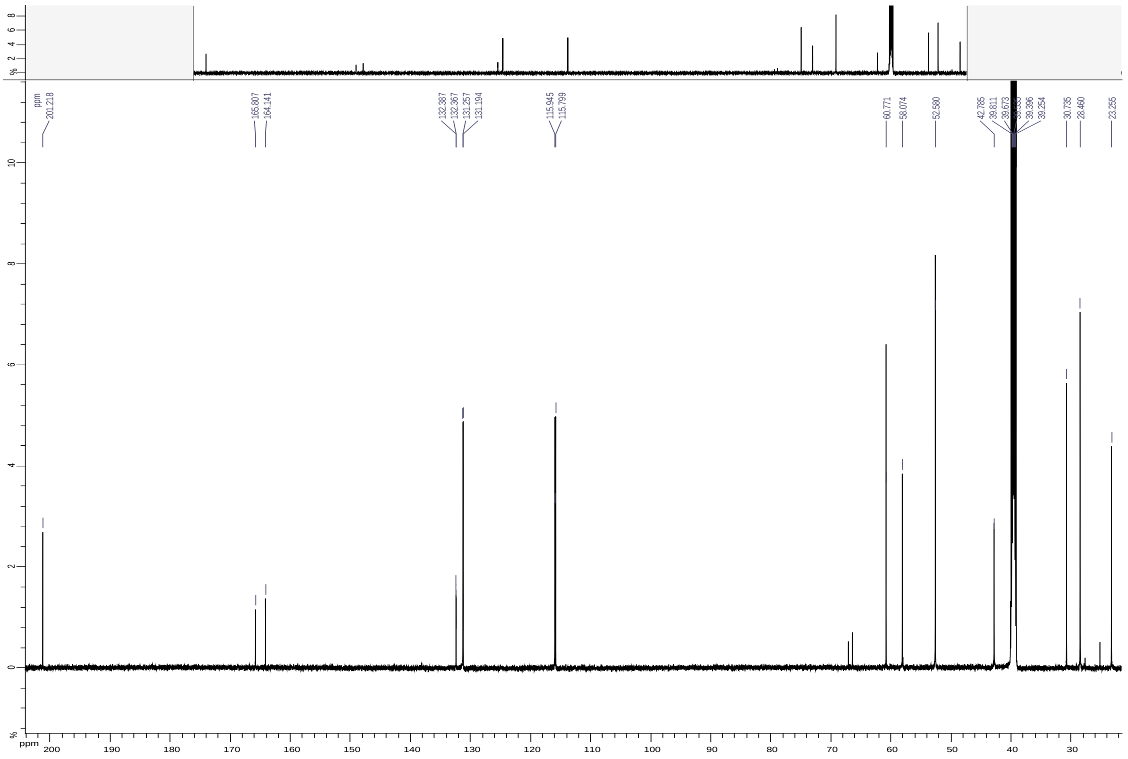

tert-Butyl 4-((4-fluorophenyl)(hydroxy)(phenyl)methyl)piperidine-1-carboxylate (U)

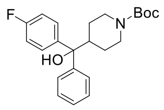

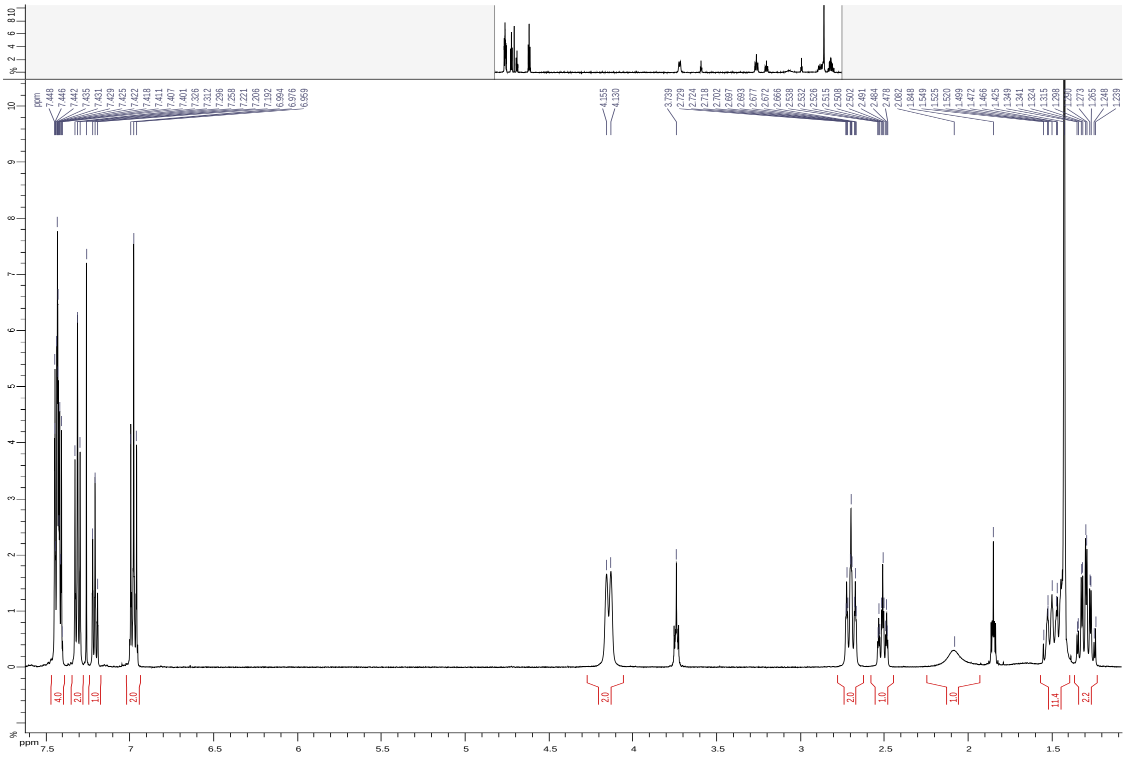

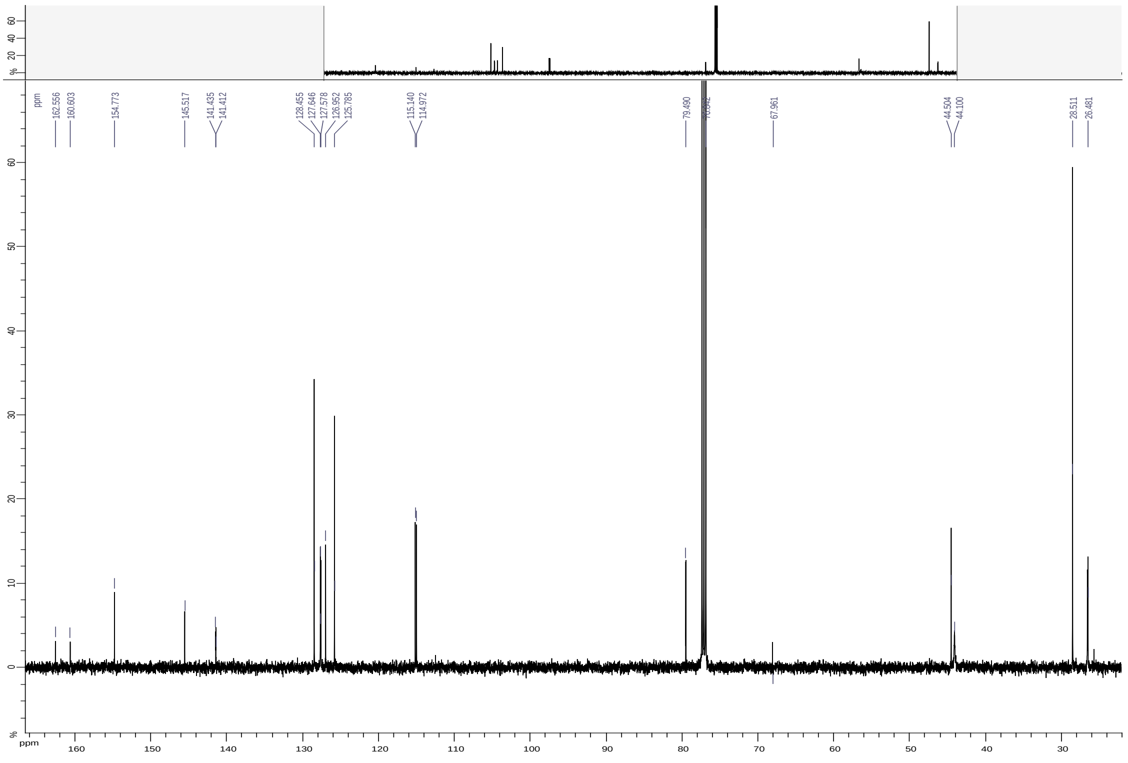

4-((4-Fluorophenyl)(phenyl)methylene)piperidine (V)

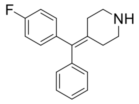

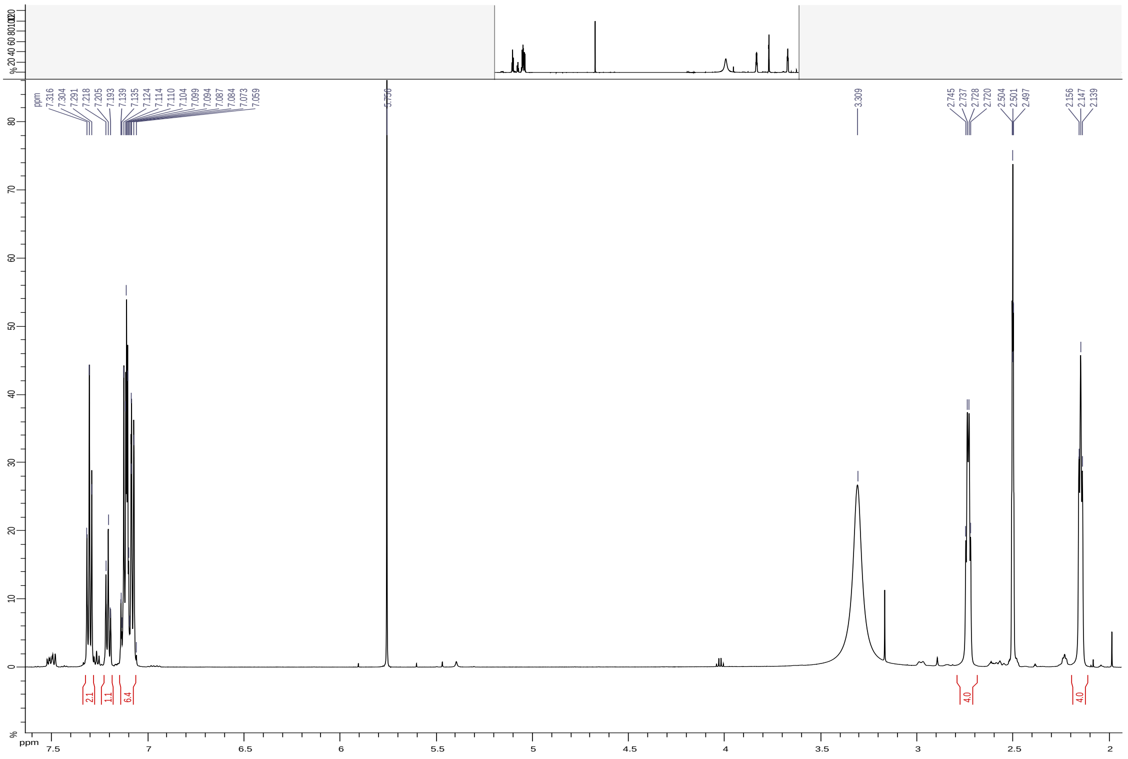

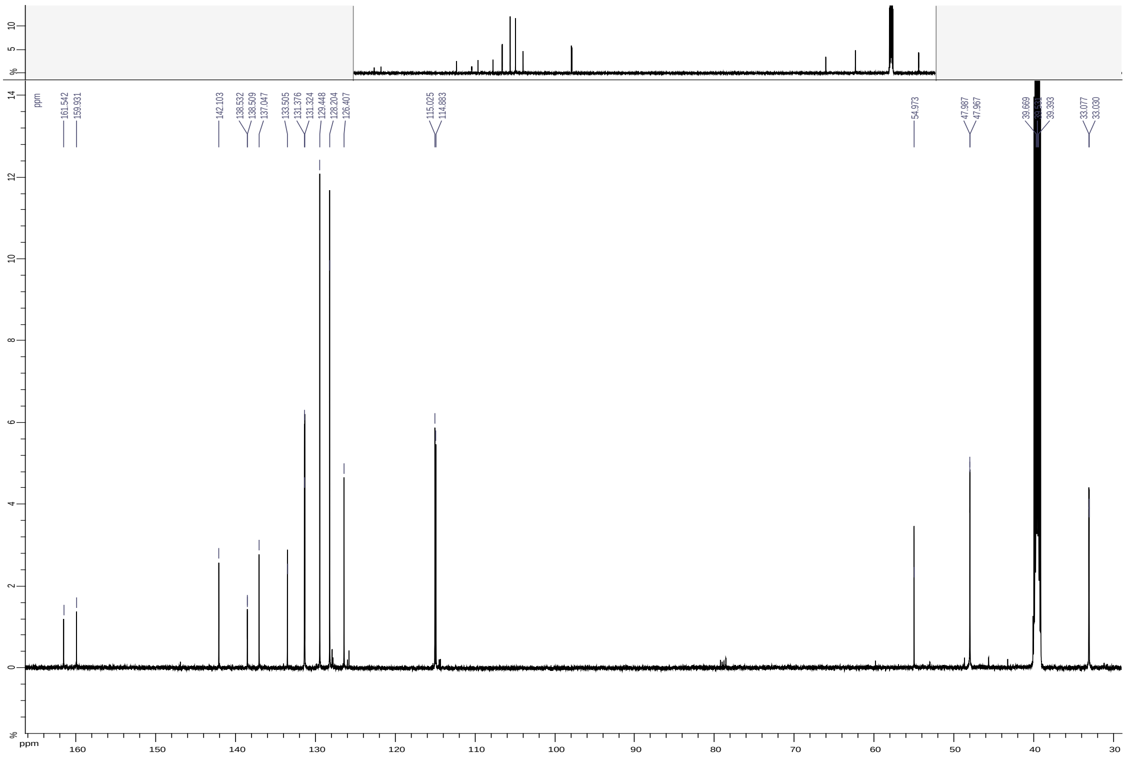

Ethyl 4-(4-((4-Fluorophenyl)(phenyl)methylene)piperidin-1-yl)butanoate (8c)

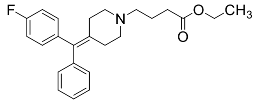

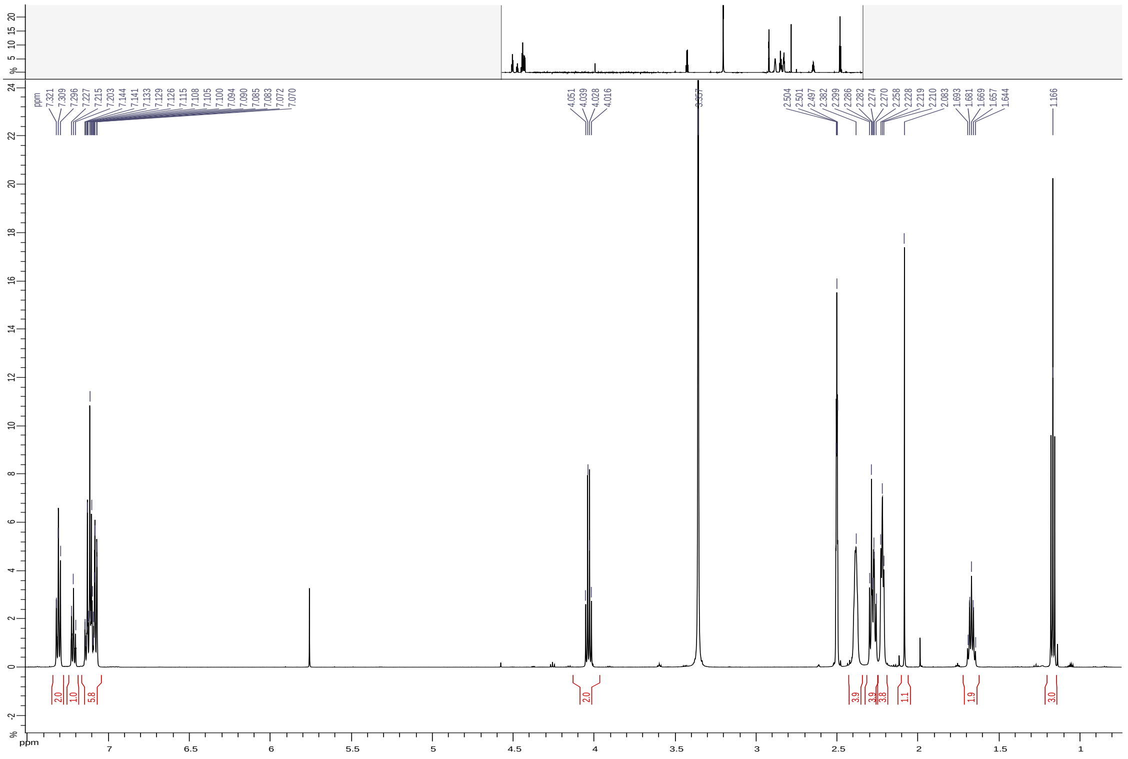

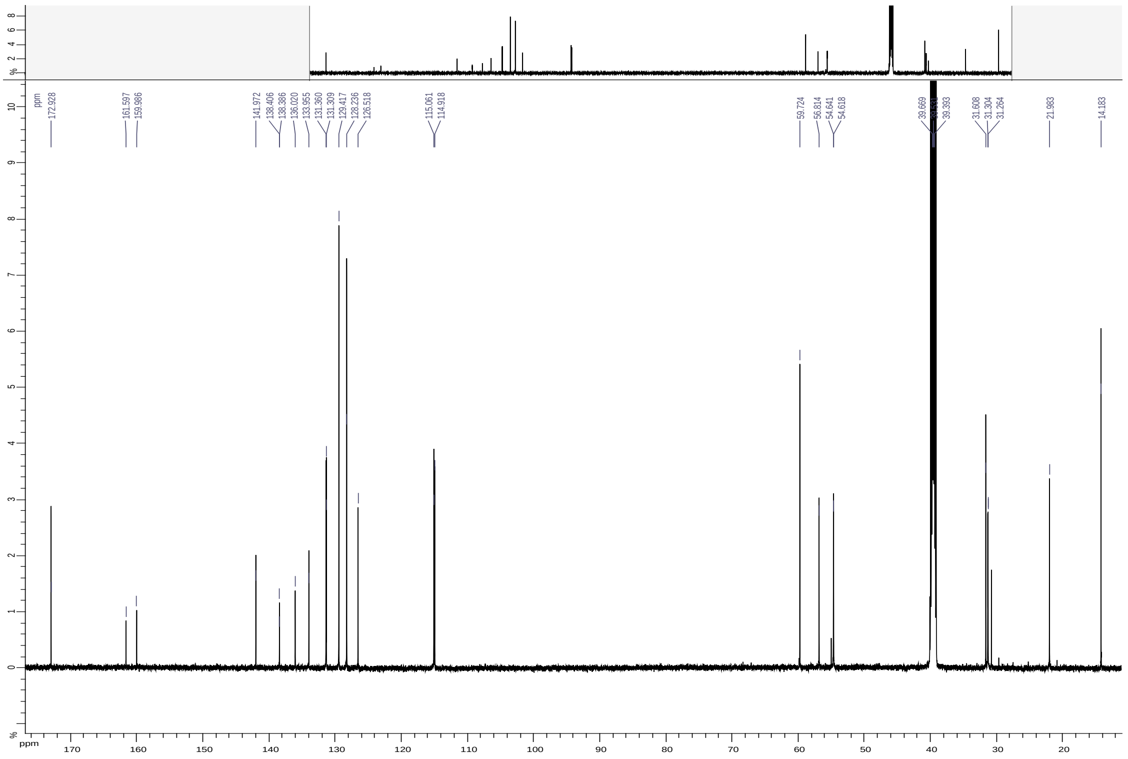

4-(4-((4-Fluorophenyl)(phenyl)methylene)piperidin-1-yl)butan-1-ol (10d)

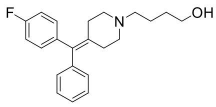

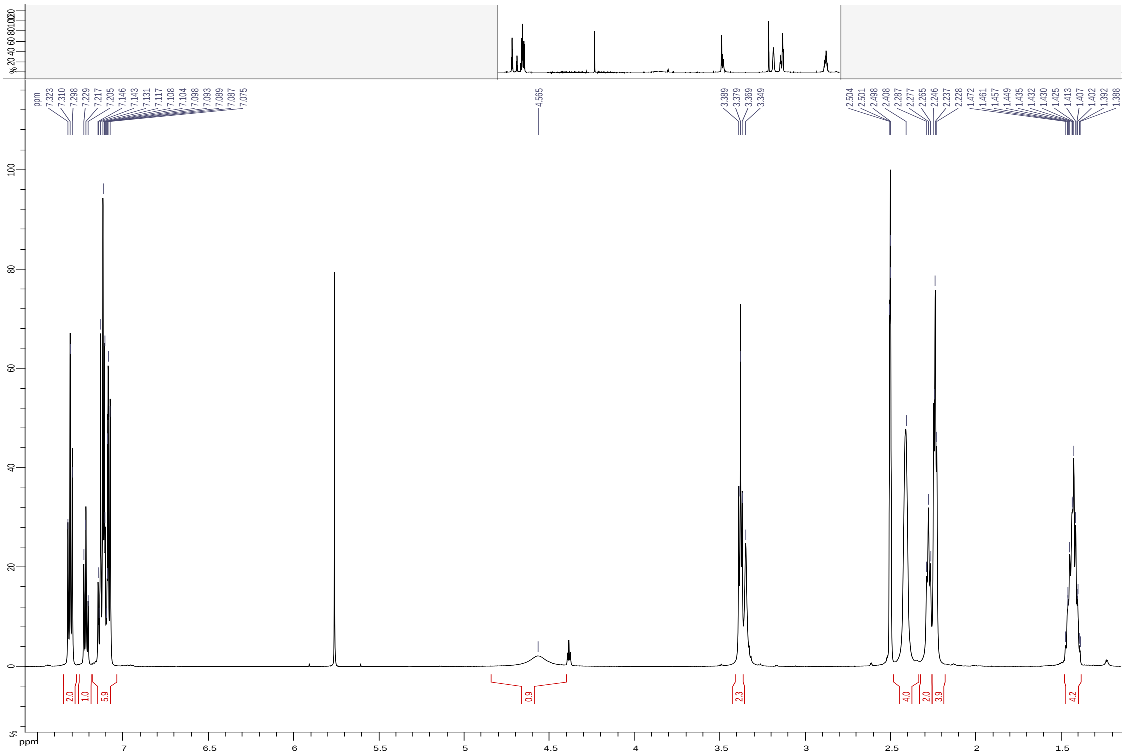

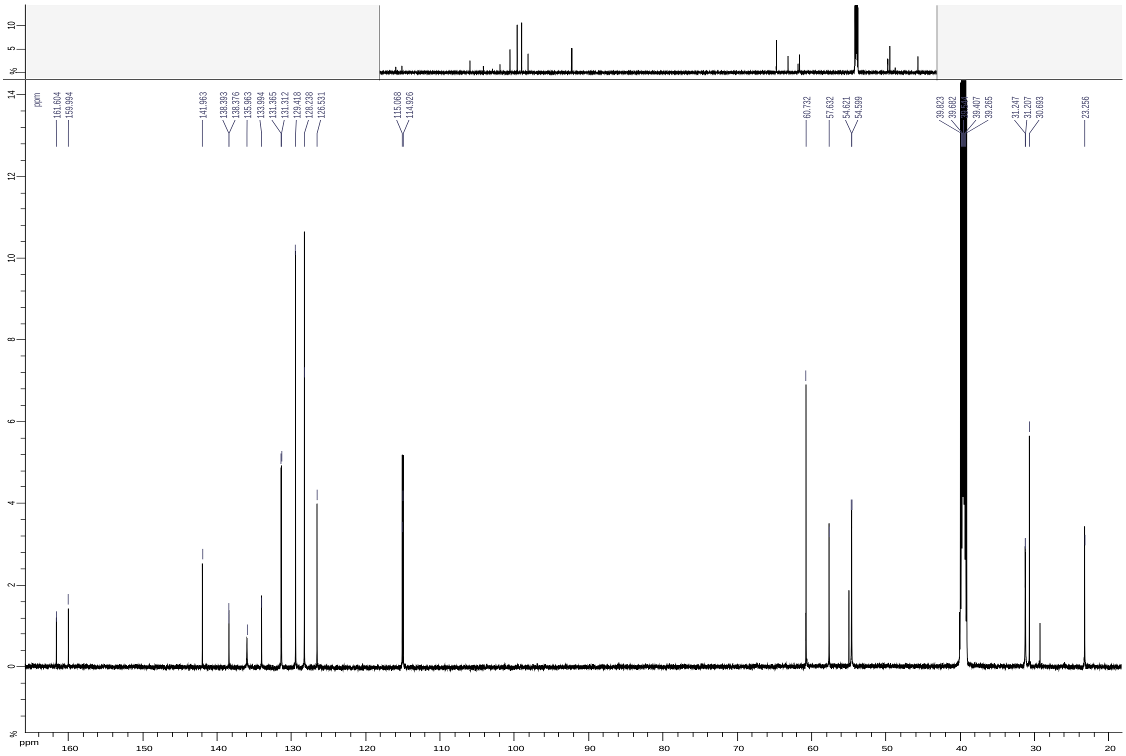

2-Fluoro-5-phenylpyridine (11c)

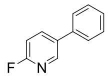

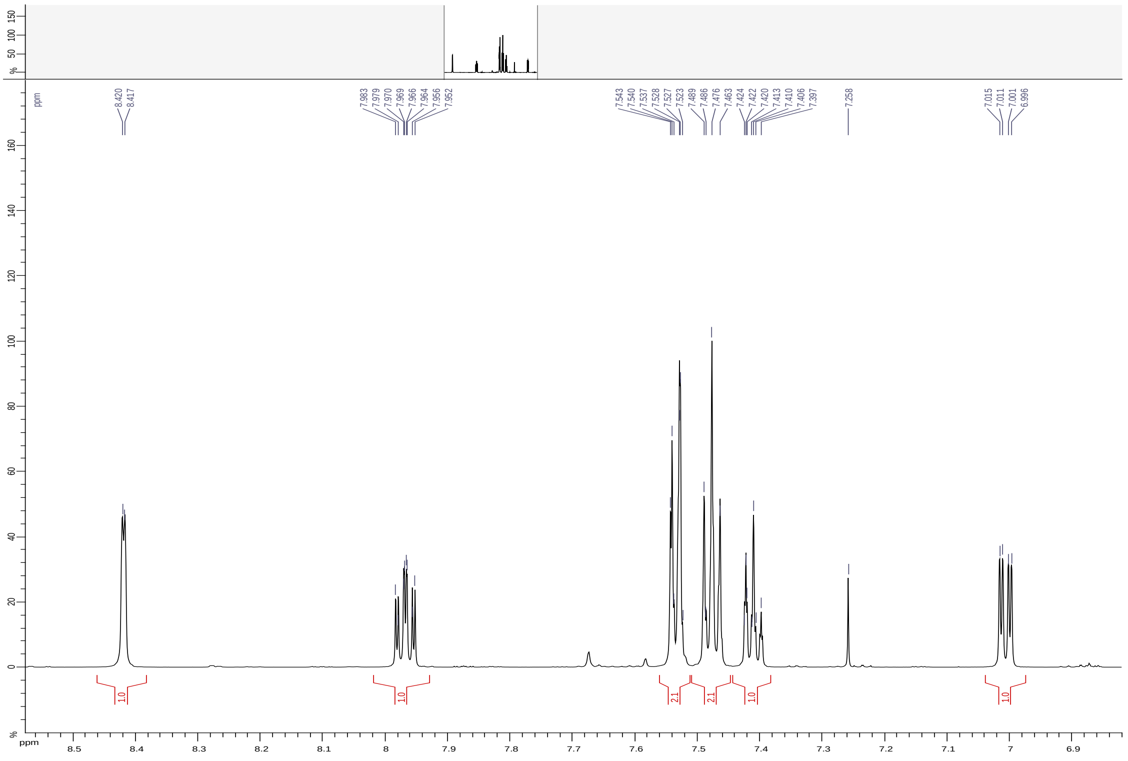

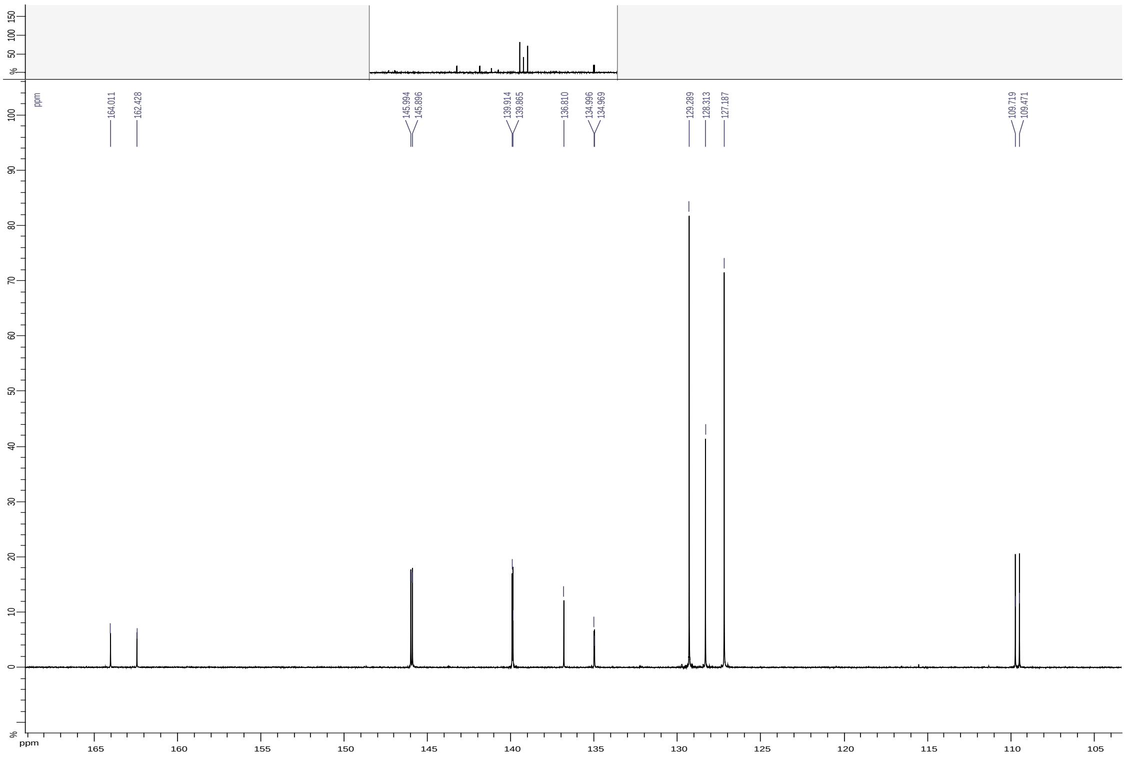

2-Fluoro-5-(pyrrolidin-2-ylmethoxy)pyridine (12d)

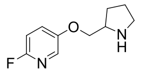

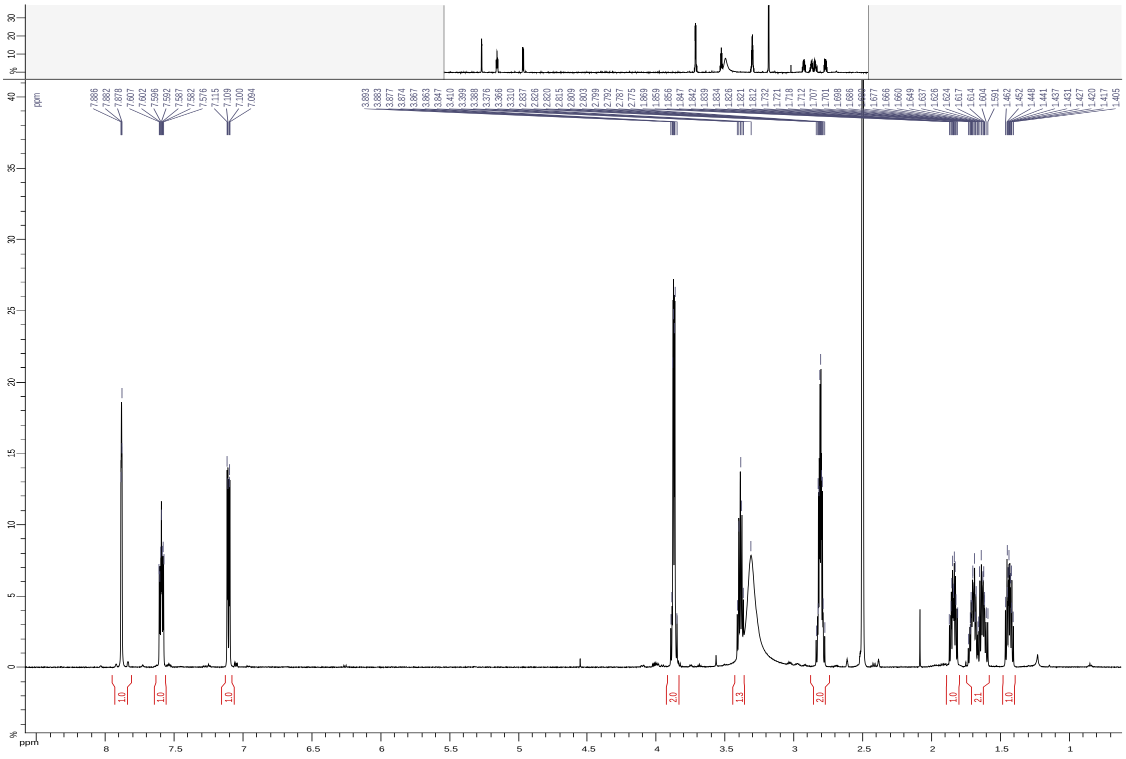

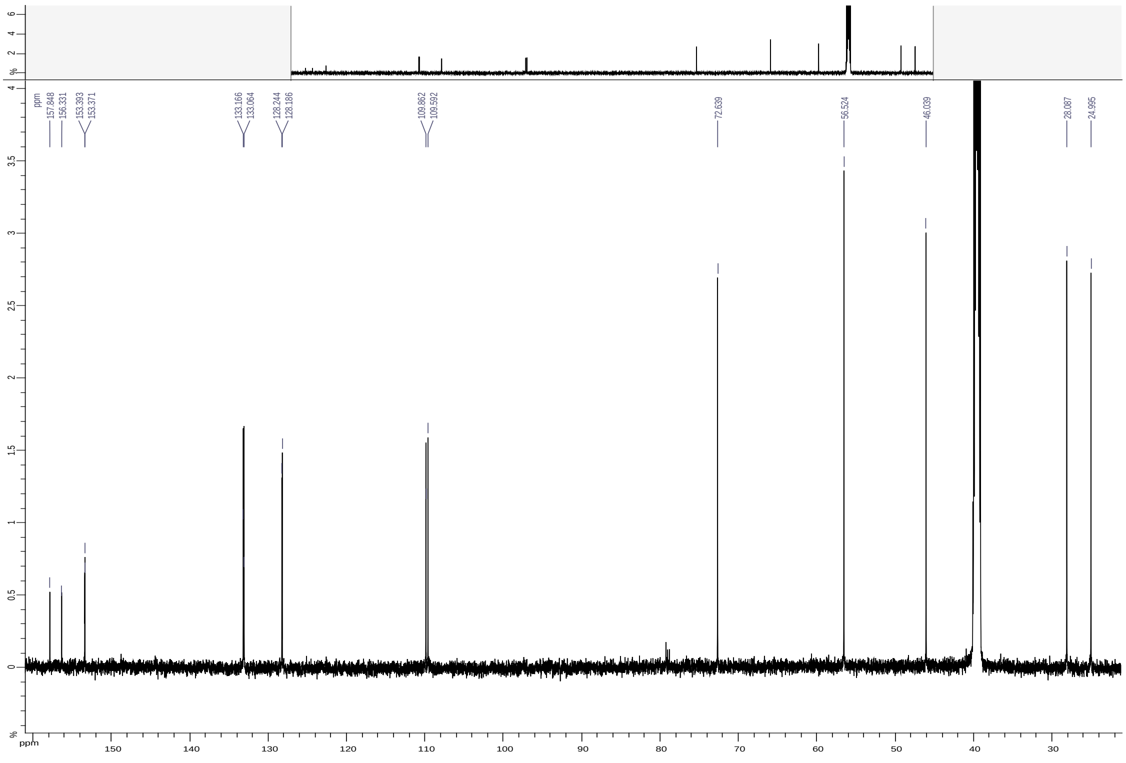

N-(1-(3-Fluorobenzyl)quinolin-4(1*H*)-ylidene)pentan-1-amine (14c)

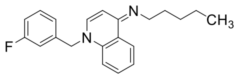

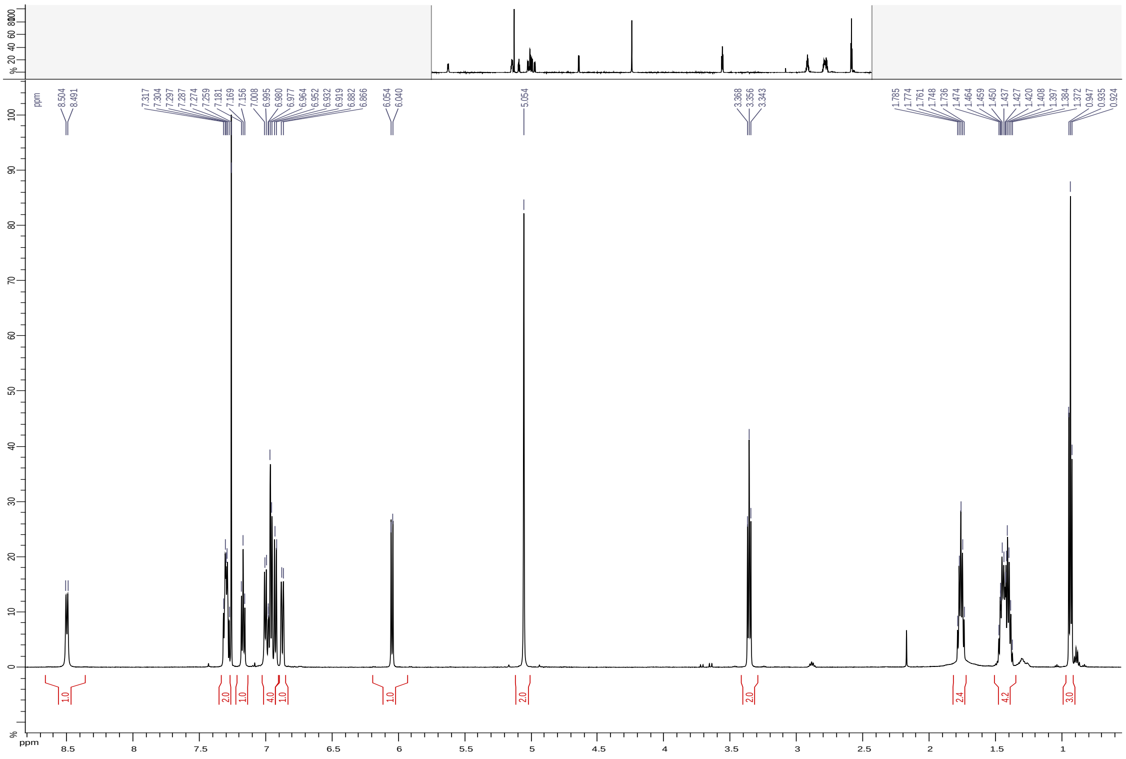

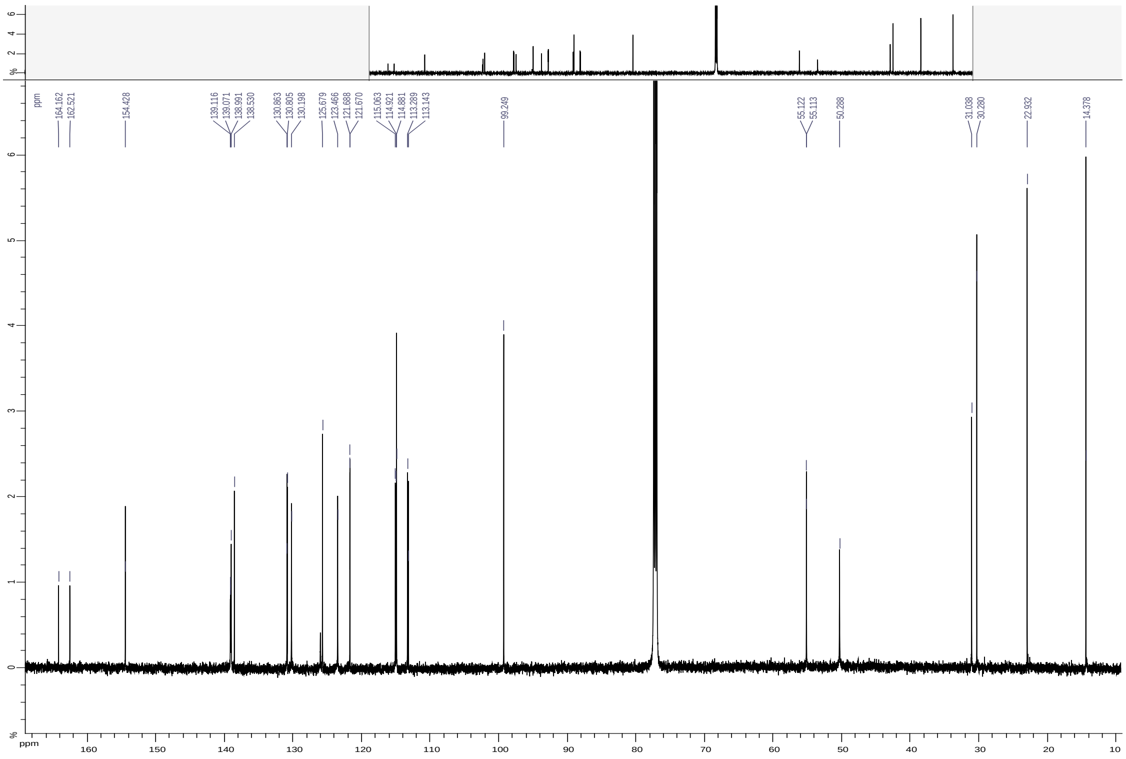

1-(2-Chloro-5-fluorophenyl)ethan-1-ol (W)

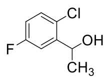

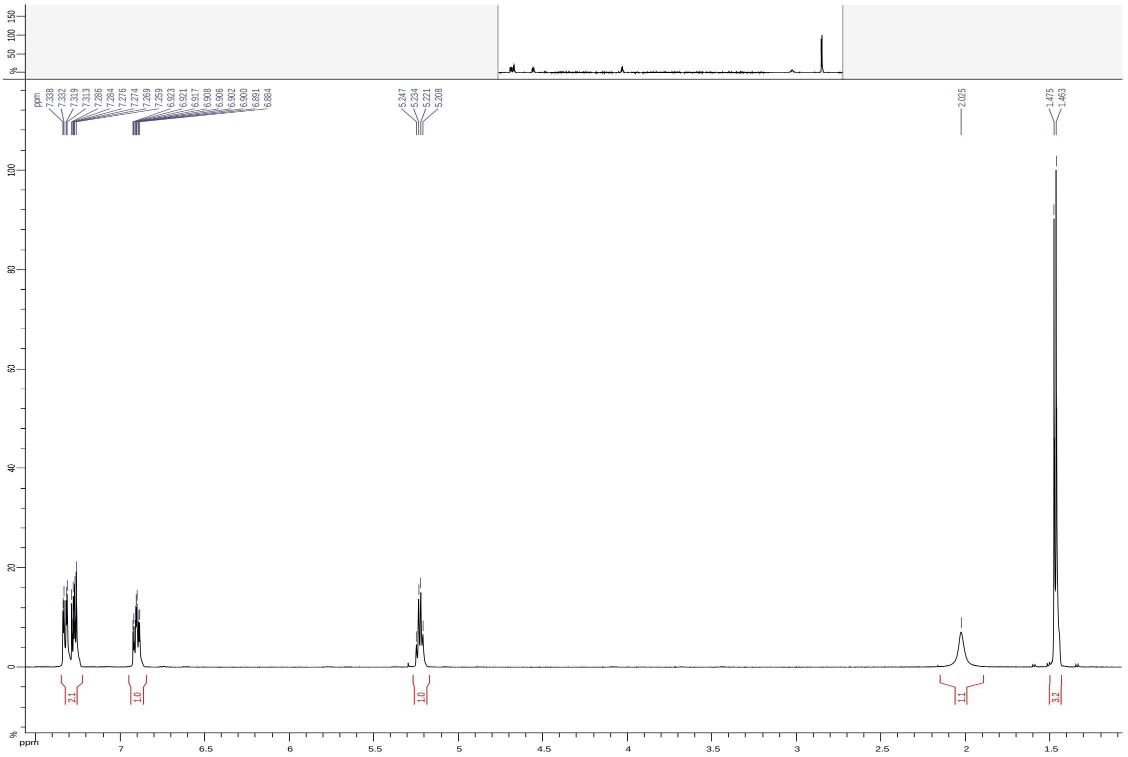

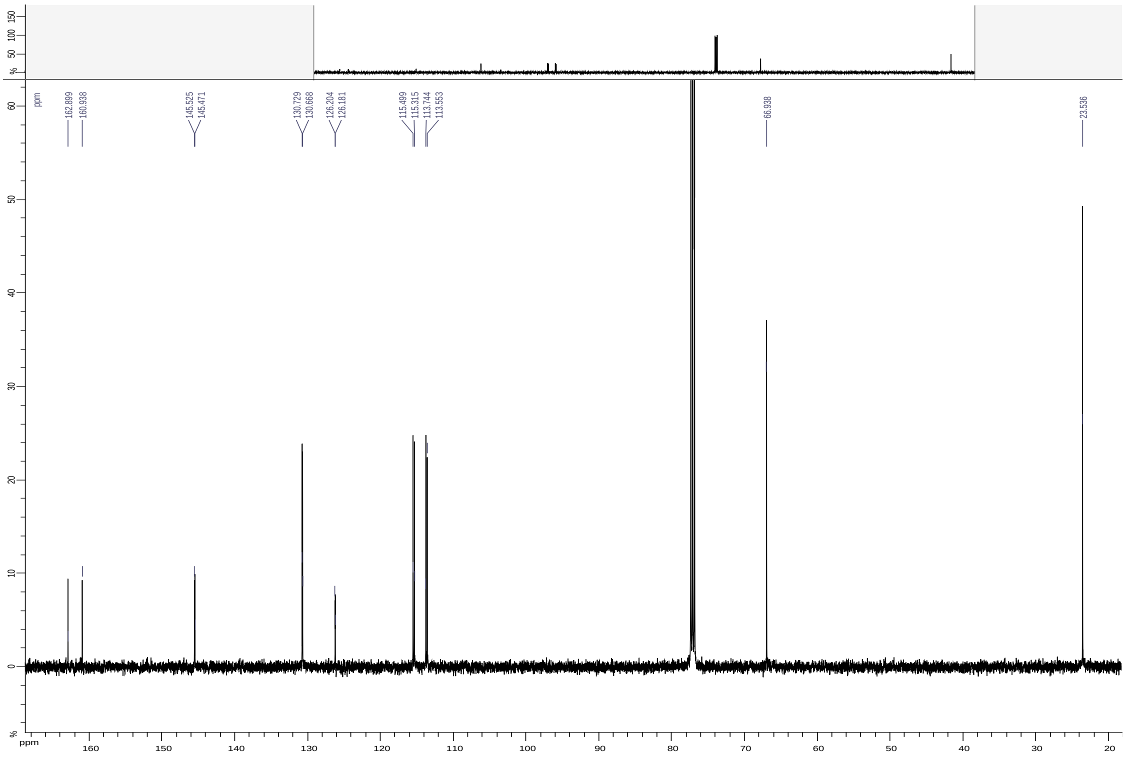

2-(1-Bromoethyl)-1-chloro-4-fluorobenzene (X)

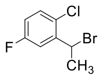

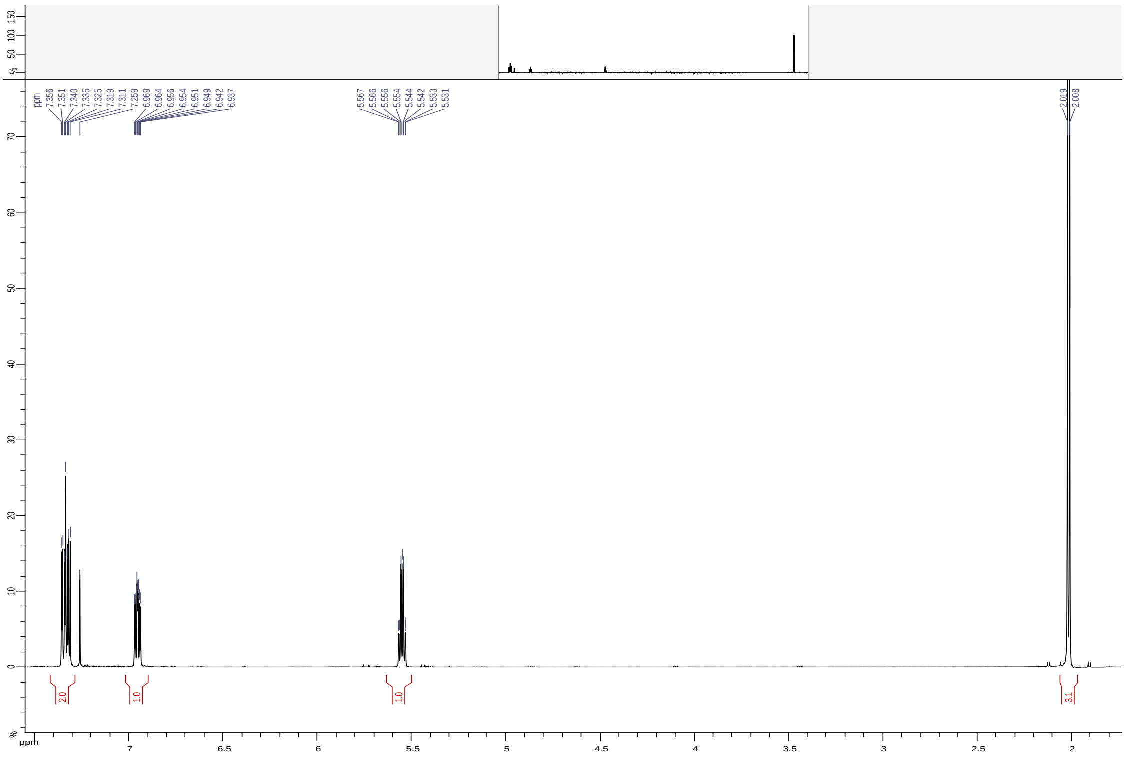

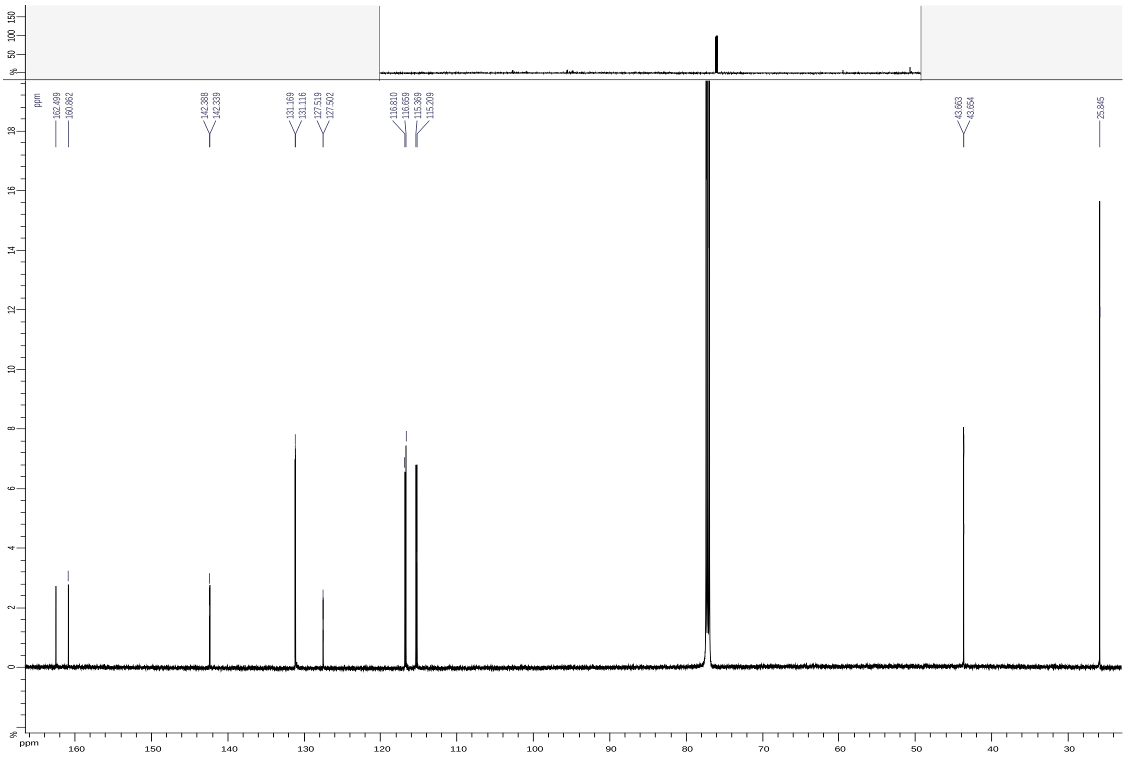

1-(1-(2-Chloro-5-fluorophenyl)ethyl)-1*H*-imidazole (13c)

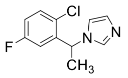

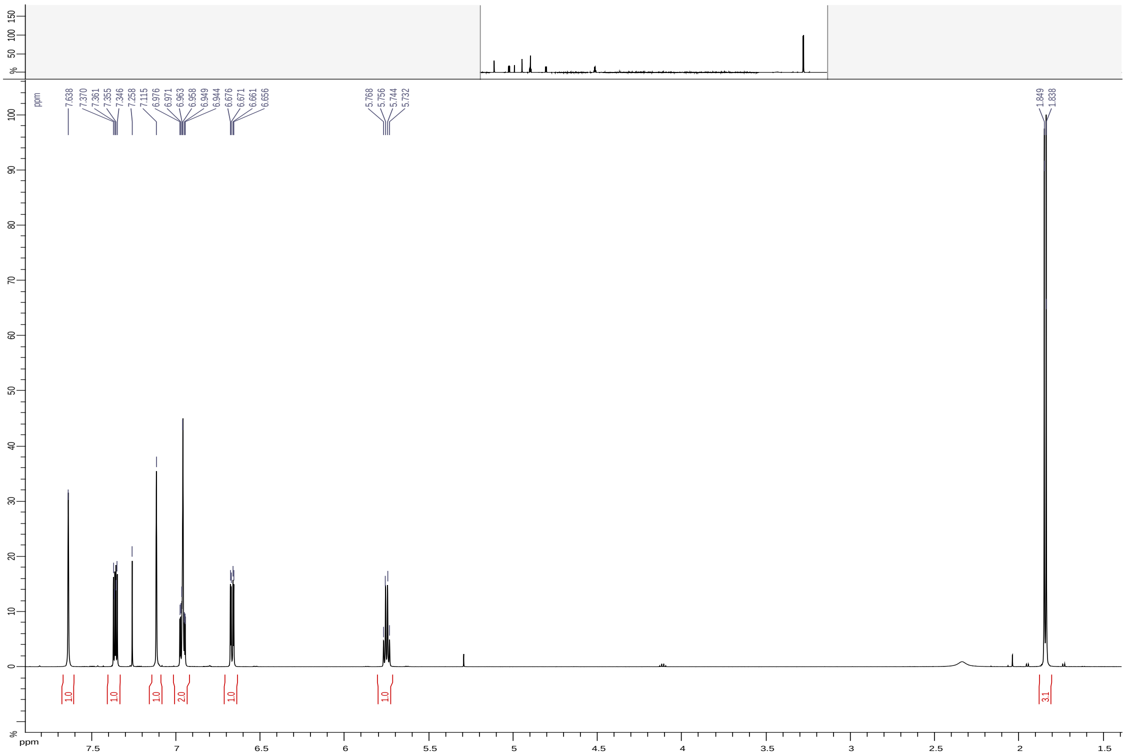

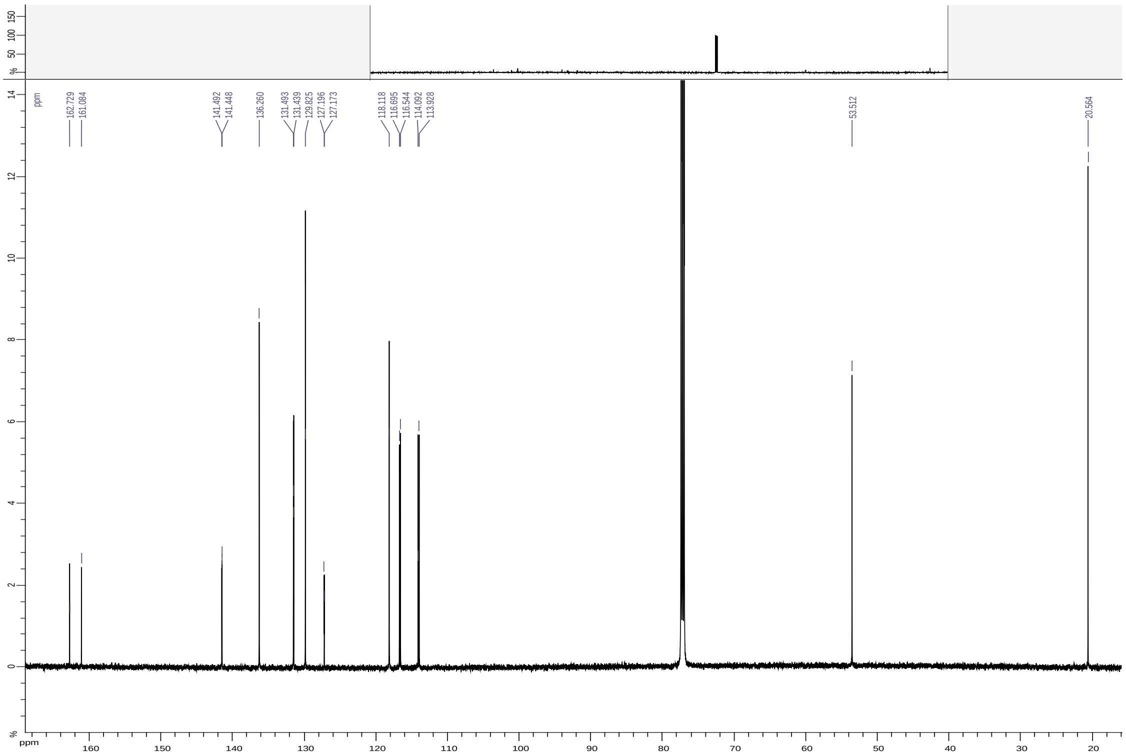

Supplement: Supplementary Information [file srep09941-s1.pdf]
